# Direct dehydrogenative alkyl Heck-couplings of vinylarenes with umpolung aldehydes catalyzed by nickel

**DOI:** 10.1038/s41467-019-08631-1

**Published:** 2019-02-12

**Authors:** Leiyang Lv, Dianhu Zhu, Chao-Jun Li

**Affiliations:** 1 Department of Chemistry and FRQNT Center for Green Chemistry and Catalysis, McGill University, 801 Sherbrooke Street West, Montreal, Quebec, H3A 0B8 Canada; 2 State Key Laboratory of Applied Organic Chemistry, Lanzhou University, 222 Tianshui Road, Lanzhou, Gansu 730000 China

## Abstract

Alkenes are fundamental functionalities in nature and highly useful intermediates in organic synthesis, medicinal chemistry and material sciences. Transition-metal-catalyzed Heck couplings with organic halides as electrophiles have been established as a powerful protocol for the synthesis of this valuable building block. However, the requirement of organic halides and the generation of stoichiometric hazardous halide wastes may cause significant sustainable concerns. The halide-free oxidative Heck alkenylations involving organometallics or arenes as the coupling partners provide a facile and alternative pathway. Nonetheless, stoichiometric amounts of extra oxidant are essential in most cases. Herein, we present a direct dehydrogenative alkyl Heck-coupling reaction under oxidant-free conditions, liberating hydrogen, nitrogen and water as the side products. Excellent regioselectivity is achieved via neighboring oxygen atom coordination. Broad substrate scope, great functional group (ketone, ester, phenol, free amine, amide etc) tolerance and modification of pharmaceutical candidates and biological molecules exemplified its generality and practicability.

## Abstract (web summary)

Heck coupling of alkenes with organic halides, arenes or organometallics poses sustainability issues due to hazardous waste formation and use of oxidants. Here, the authors show an oxidant-free alkyl Heck-coupling of vinylarenes with umpolung aldehydes, liberating H_2_, N_2_ and H_2_O as sole side products.

## Introduction

**Fig. 1:**
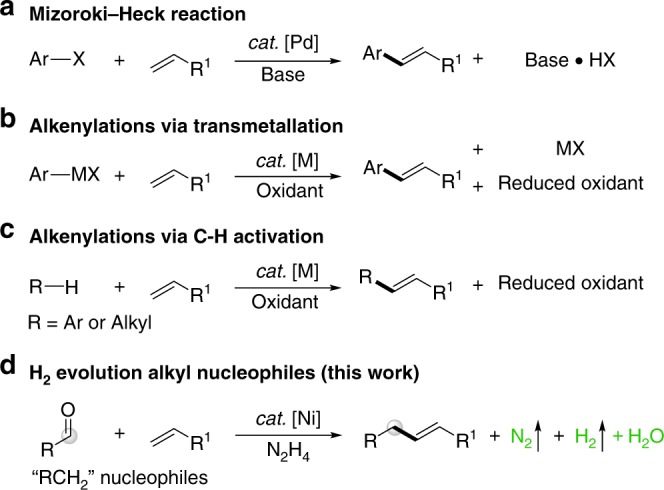
Strategies in the Heck coupling reaction. **a** Traditional Mizoroki-Heck coupling with hazardous organic halide; **b** Oxidative Heck coupling with pre-synthesized organometallic reagent; **c** Stoichiometric oxidant mediated Heck coupling via C–H bond activation; **d** Oxidant-free direct dehydrogenative alkyl Heck-coupling

Since its first discovery in the 1970s, the palladium-catalyzed Heck (or Mizoroki-Heck) reaction has emerged as the most powerful and straightfoward tool for the cross-couplings of alkenes and aryl (pseudo) halides (Fig. 1a)^1,2^. Despite its remarkable importance and widespread applications in organic synthesis^3–5^ over the past decades, this protocol suffers from the inherent disadvantage of the required pre-synthesizing the organic halides and accompanied formation of a stoichiometric amount of hazardous halide salt, which can cause significant environmental concerns. To persuit a more sustainable alkenylation process, the halide-free oxidative Heck coupling reactions via transmetallation or directed C–H activation have been developed^6–8^. Organometallic reagents are generally moisture/air sensitive and often not commercially available especially when bearing functional groups (Fig. 1b). Besides, preparation of these reagents requires stoichiometric quantities of metals, thus producing extra metal wastes. Alternatively, the alkenylations through direct cleavage of two C–H bonds represent an more environmentally benign and economically attractive strategy (Fig. 1c)^9–12^. As early as in 1967, Fujiwara and Moritani reported the cross-couplings of electron-rich arenes with activated alkenes^13^. Inspired by this precedent work, great achievements have been made in this field^14,15^. However, due to the inherent challenges of selectivity in C–H bond activation, the nucleophiles were mostly restricted to the (hetero) arenes with a directing group. Above all, the oxidative Heck reaction generally requires stoichiometric amounts of oxidant (such as metal salt, peroxide, benzoquinone, K_2_S_2_O_8_, O_2_, acetone^16^, or internal oxidant^17–19^ etc.) to regenerate the active metal species, thus often affording additional side products. Hence, the development of a dehydrogenative Heck-coupling reaction under oxidant-free conditions is promising and highly desirable^20,21^. In 2016, Jeganmohan and coworkers reported an attractive ruthenium-catalyzed oxidant-free *ortho* alkenylation of aromatic amides and anilides with acetic acid as an additive^22^. Two examples of electrocatalytic aromatic dehydrogenative Heck coupling of arenes were disclosed by Jutand and Lei groups^23,24^. Recently, Lei and coworkers also realized a direct dehydrogenative C–H alkenylation of electron-rich arenes with styrene derivatives via a photo-induced electron transfer process^25^.

In contrast, alkyl-Heck-type reactions were much more challenging and rarely explored until the last decade. Pioneered by Oshima^26^ and Fu’s seminal works^27^, effective strategies have been developed to facilitate this alkenylations with simple alkyl halides as electrophiles^28–44^. Notably, two elegant palladium-catalyzed cases involving alkyl C–H activation were disclosed by the groups of Yu^45^ and Sanford^46^, which employed *N*-arylamide or pyridine as a neighboring directing group with stoichiometric amounts of external oxidant. However, up to now, the oxidant-free, direct generation of H_2_ from alkyl Heck-type reaction still remains a highly formidable task.

To address these challenges and towards the goal of sustainable transformations, herein, we wish to report the example of direct dehydrogenative alkyl Heck-couplings of vinylarenes with umpolung aldehydes (Fig. 1d). Inspiration of this project stemsfrom our recent studies on hydrazone chemistry, in which umpolung aldehydes act as carbanion equivalents in the catalytic nucleophilic addition^47–52^ and cross-coupling reactions^53–56^. Highlighted features of this strategy are (a) no oxidant needed; (b) H_2_, N_2_ and H_2_O as innocuous side products; (c) naturally rich aldehydes as environmentally benign alkyl nucleophiles; (d) excellent regioselectivity achieved via vicinal oxygen atom chelation; (e) first-row abundant nickel as catalyst; (f) broad substrate scope and great functional group compatibility; and (g) modification of pharmaceutical candidates and biological molecules.

## Results

### Screening of reaction conditions

Our study commenced with a model reaction of styrene (**3a**) with hydrazone (**2a**) generated in situ from benzaldehyde (**1a**) with hydrazine monohydrate (Table 1) [Warning: hydrazine monohydrate is potentially hazardous and should be performed with appropriate personal protection]. We first examined Ni(cod)_2_ as a catalyst precursor and *N*,*N*-diisopropyl ethylamine as the base (DIPEA) for the Heck-type reaction. Among the various phosphine ligands evaluated (entries 1–6, see Supplementary Table 1 for details), only the sterically hindered, strong σ-donor bidentate alkyl phosphine ligand, 1,2-bis(dicyclohexyl phosphanyl)ethane (dcype), favored this transformation, and the product prop-1-ene-1,3-diyldibenzene **4aa** was obtained in 78% yield with 87:13 *E*:Z ratio (entry 6). The liberation of H_2_ gas was confirmed by gas chromatograph with a TCD detector. The operationally simple Ni(II) precatalysts were then tested (entries 7–10), only Ni(acac)_2_ afforded the target molecule in 10% yield. Gratifyingly, 91% yield of **4aa** was observed with more than 95:5 *E*:Z selectivity when 0.5 equivalent of NaI was added (entry 11). Notably, the efficiency of this transformation was almost not affected using twofold excess of benzaldehyde hydrazone **2a** (entry 12). Equimolar reaction of styrene **3a** and **2a** still delivered the desired product **4aa** in 58% yield (entry 14). When the dosage of Ni(cod)_2_ and dcype was decreased to 5 mol%, 78% yield of corresponding **4aa** was obtained (entry 15). Control experiments were also carried out to understand the role of each component. It is noteworthy that in absence of base, the reaction efficiency was only decreased slightly (entry 16). This result indicated that base was not essential for this transformation, but might partially assist the initial oxidative addition of nickel complex to N–H bond of hydrazone^57^. In absence of either the nickel catalyst or a ligand, the desired product **4aa** was not detected (entries 17 and 18).

**Table 1:** Optimization of the reaction conditions


Entry	Catalyst	Ligand	Additive	4aa (%)^a,b^
1	Ni(cod)_2_	PMe_3_	—	N.D.
2	Ni(cod)_2_	PPh_3_	—	N.D.
3	Ni(cod)_2_	PCy_3_	—	N.D.
4	Ni(cod)_2_	dmpe	—	N.D.
5	Ni(cod)_2_	dppe	—	N.D.
6	Ni(cod)_2_	dcype	—	78 (83:17)
7	NiCl_2_	dcype	—	Trace
8	NiBr_2_	dcype	—	Trace
9	NiBr_2_•glyme	dcype	—	Trace
10	Ni(acac)_2_	dcype	—	10 (78:22)
11	Ni(cod)_2_	dcype	NaI	91 (87)^c^ (>95:5)
12^d^	Ni(cod)_2_	dcype	NaI	85 (>95:5)
13^e^	Ni(cod)_2_	dcype	NaI	71 (>95:5)
14^f^	Ni(cod)_2_	dcype	NaI	58 (>95:5)
15^g^	Ni(cod)_2_	dcype	NaI	78 (>95:5)
16^h^	Ni(cod)_2_	dcype	NaI	69 (>95:5)
17	Ni(cod)_2_	—	NaI	N.D.
18	—	dcype	NaI	N.D.

Reaction conditions: **3a** (0.2 mmol), **1a** (0.6 mmol), N_2_H_4_•H_2_O (0.72 mmol), Ni(cod)_2_ (10 mol%), ligand (20 mol% for monodentate, 10 mol% for bidentate), DIPEA (0.4 mmol), NaI (0.1 mmol), THF (1.0 mL), 100 °C, 12 h under N_2_ unless otherwise noted

*N.D.* not detected

^a^NMR yields were determined by ^1^H NMR using mesitylene as an internal standard and based on **3a**

^b^The *E*:Z ratio in parenthesis was determined by ^1^H NMR analysis of the crude mixture

^c^The isolated yield in parenthesis

^d^**1a** (0.4 mmol), N_2_H_4_•H_2_O (0.48 mmol) instead

^e^**1a** (0.3 mmol), N_2_H_4_•H_2_O (0.36 mmol) instead

^f^**1a** (0.2 mmol), N_2_H_4_•H_2_O (0.24 mmol) instead

^g^Ni(cod)_2_ (5 mol%), ligand (5 mol%) instead

^h^Without adding DIPEA

### Scope and limitation of the reaction

**Fig. 2:**
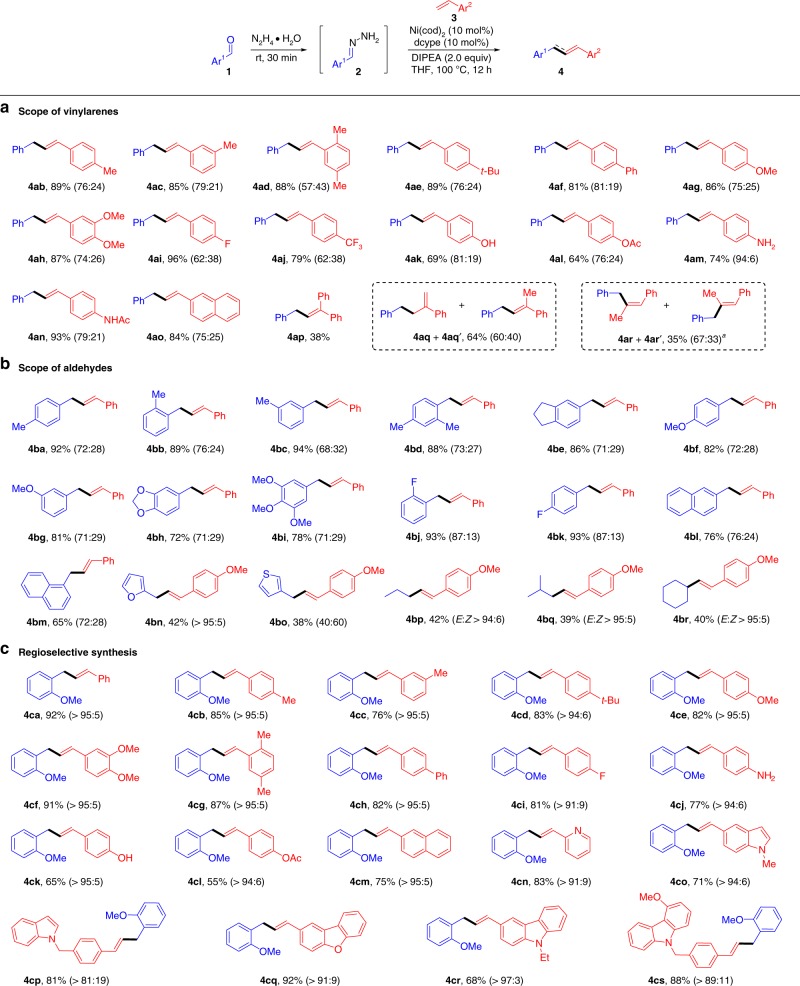
Scope of substrates. Reaction conditions: **3** (0.2 mmol), **1** (0.6 mmol), N_2_H_4_•H_2_O (0.72 mmol), Ni(cod)_2_ (10 mol%), dcype (10 mol%), DIPEA (0.4 mmol), NaI (0.1 mmol), THF (1.0 mL), 100 °C, 12 h under N_2_. Reported yields are the isolated ones (the ratio of allylic isomerization was in parentheses), the *E*:Z ratio was more than 20:1 unless otherwise noted. ^*a*^The Z:*E* ratio in parenthesis

With the optimized conditions identified, the substrate scope of olefins **3** was investigated (Fig. 2a). To our delight, an array of mono-substituted vinylarenes containing both electron-donating and electron-withdrawing groups were all proved to be competent substrates, delivering the desired products **4ab-ao** in 64–96% yields. The allylic isomerization of the product was attributed to the presence of Ni–H species in the reaction process. Trace amount of hydrogenation product of styrene was also detected by GC-MS. Various functional groups, including methyl (**4ab-ad**), *tert*-butyl (**4ae**), phenyl (**4af**), methoxyl (**4ag-ah**), fluoride (**4ai**), and trifluoromethyl (**4aj**), were accommodated under the optimal conditions. Remarkably, the alkenes with sensitive functional groups such as hydroxyl (**4ak**), ester (**4al**), amine (**4am**), and amide (**4an**), which are typically biased in the presence of organometallic reagents, could efficiently participated in this reaction. Steric hindrance on the double bond of vinylarene diminished the yield. For example, the target product **4ap** was obtained in 38% yield when 1,1-diphenylethylene was applied. Treatment of α-methylstyrene under the standard conditions gave the allylic isomers **4aq** and **4aq’** in combined 64% yield with 60:40 regioselectivity. With respect to β-methylstyrene, Z:*E* isomers (**4ar** and **4ar’**) were achieved in combined 35% yield. Unfortunately, vinylsilane, aliphatic and electron-deficient alkenes failed to give the corresponding products under the current catalytic system.

Next, we proceeded to examine the substrate scope of aldehydes **1** (Fig. 2b). In general, the electronic effects of the nucleophiles did not influence the efficiency of this transformation. Aldehydes bearing both electron-donating and electron-withdrawing substituents all reacted smoothly with styrene **3a** to afford the products **4ba-bm** in 65–94% yields. Hetero-aromatic aldehydes, such as furan-2-carbaldehyde and thiophene-3-carbaldehyde, also successfully gave the desired products (**4bn** and **4bo**) in moderate yields. To our delight, aliphatic aldehydes, such as propionaldehyde and iso-butyraldehyde, which are challenging substrates in the previous reports, were also applicable in this Heck-type reaction (**4bp** and **4bq**). Moreover, the attempt to apply ketone as a nucleophile in this transformation also proved feasible (**4br**).

It is worth noting that an excellent regioselectivity (>95:5) was observed when furan-2-carbaldehyde **1n** was examined (Fig. 2b, **4bn**). We therefore hypothesized that if the chelation or directing effect of the vicinal oxygen atom inhibited the allylic isomerization^58^. Thus, the phenyl aldehyde with an *ortho*-methoxyl group **1s** was selected as the electrophile candidate. As shown in Fig. 2c, a variety of vinylarenes attached with different functional groups were re-examined with 2-methoxybenzaldehyde **1s** under the optimal conditions, and excellent regioselectivity (mostly >95:5) of the desired products **4ca-cm** were obtained also with good yields. Furthermore, the olefins bearing heterocyclic skeletons including pyridine, dibenzo-furan, functionalized indole and carbazole, were all viable substrates, affording the corresponding products (**4cn-cs**) in 68–92% yields.

### Synthetic applications

**Fig. 3:**
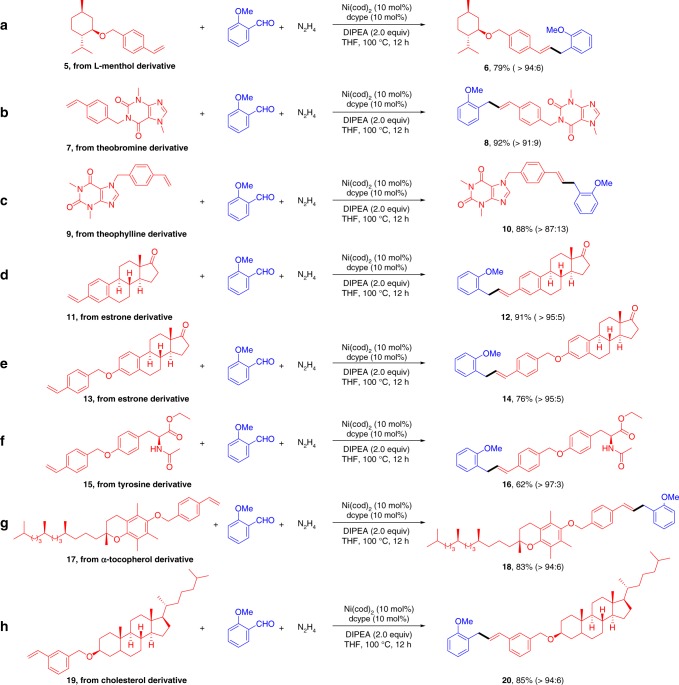
Functionalization of naturally and pharmaceutically important derivatives. **a** Regioselective Heck coupling of L-Menthol derivative; **b** Regioselective Heck coupling of Theobromine derivative; **c** Regioselective Heck coupling of Theophylline derivative; **d** Regioselective Heck coupling of vinyl Estrone derivative; **e** Regioselective Heck coupling of Estrone derivative; **f** Regioselective Heck coupling of Tyrosine derivative; **g** Regioselective Heck coupling of α-Tocopherol derivative; **h** Regioselective Heck coupling of Cholesterol derivative

The promising functional group tolerance and high-efficiency of this protocol enabled its application to the modification of pharmaceuticals and natural product derivatives (Fig. 3). For example, introducing *ortho*-methoxyl benzyl group into L-Menthol (**5**) was successfully achieved in excellent yield with good regioselectivity (>94:6). Alkaloids such as Theobromine (**7**) and Theophylline derivatives (**9**) reacted smoothly under current catalytic system. The estrone derivatives **11** and **13** were also tested as superior candidates, delivering the target molecules **12** and **14** with the ketone moiety untouched. In addition, the structural elaboration of tyrosine derivative (**15**) was readily accomplished with this nickel-catalyzed oxidant-free Heck-coupling strategy. Moreover, the cross-couplings of **1** **s** with α-Tocopherol and Cholesterol derivatives were investigated under the optimized conditions, and the expected benzylation products (**18** and **20**) were all obtained in good yields (>80%). These examples highlighted the wide applicablity and compatiblity of the method, and its enrichment of the tool box for the modification of complex bioactive molecules.

### Mechanistic investigation

**Fig. 4:**
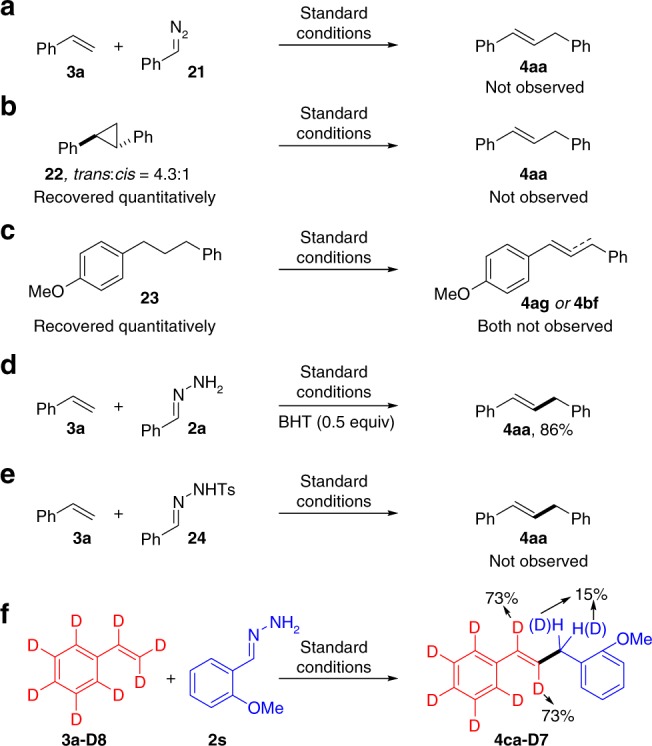
Mechanistic studies. **a** Reaction of styrene with phenyldiazomethane did not give the desired product; **b** Reaction of 1,2-diphenylcyclopropane under the standard conditions did not give the desired product; **c** Reaction of 1-methoxy-4-(3-phenylpropyl)benzene under the standard conditions did not give the desired product; **d** Radical scavenger, 2,6-di-*tert-*butyl-4-methylphenol (BHT) added and the reaction was almost unaffected; **e** *N*-Ts hydrazone used instead of simple hydrazone did not give the desired product; **f** H/D exchanges occurred in the isotope experiment

To gain preliminary insights into the reaction mechanism, several control experiments were subsequently carried out. Firstly, the reaction of styrene (**3a**) with phenyldiazomethane (**21**) did not afford the desired (*E*)-prop-1-ene-1,3-diyldibenzene (**4aa**) (Fig. 4a). When 1,2-diphenylcyclopropane (**22**) was applied under the standard conditions, the desired product **4aa** was also not observed (Fig. 4b). These two results ruled out the possibility that hydrazone played the role of carbene precursor or diazo species to undergo cyclopropanation and subsequent ring opening during the reaction course^59^. Secondly, when 1-methoxy-4-(3-phenylpropyl)benzene (**23**) was tested, neither **4ag** nor its regioisomer **4bf** was detected (Fig. 4c). This observation suggested the unlikely involvement of hydrazone addition to the vinylarene followed by dehydrogenation pathway. Thirdly, the efficiency of the model reaction was almost unaffected in the presence of a radical scavenger, 2,6-di-*tert*-butyl-4-methylphenol (BHT), indicating the unlikelihood of a radical mechanism in the current reaction (Fig. 4d). When *N*-Ts hydrazone **24** was used in the place of simple hydrazone **1a**, no desired product **4aa** was observed, which accounted for the totally diverse characters between the two different types of hydrazones (Fig. 4e)^60–62^. Finally, isotope experiment was carried out (Fig. 4f). H/D exchanges both in the double bond moiety (73% D) and benzylic position (15% D) revealed that iterative Ni-(H)D species addition/elimination steps existed during the reaction process^63,64^.

**Fig. 5:**
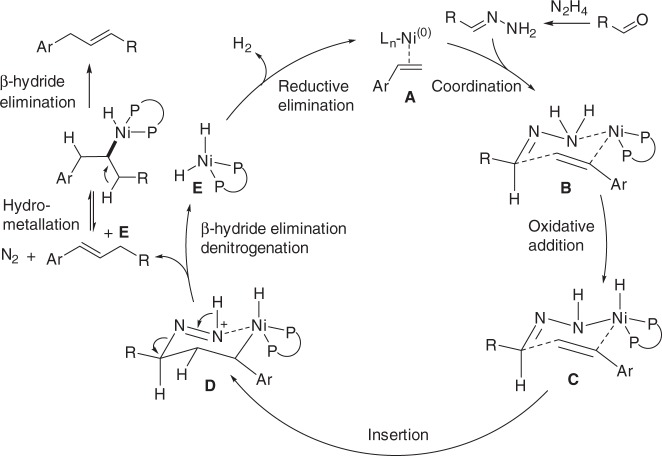
Reaction mechanism. Proposed pathway for the oxidant-free Heck-type reaction

Although, the exact mechanism still remained unclear at this moment, on the basis of literature reports^6,47–56^ and our findings, a plausible reaction pathway was proposed in Fig. 5. Initially, the active nickel(0) species, which potentially complexed to the olefin, coordinated with hydrazone to form a six-membered intermediate **B**. Then the nickel catalyst undergoes oxidative addition to the N–H bond likely with the assistance of DIPEA and delivers the key intermediate **C**. A similar N–H bond activation of simple hydrazone was also observed with a manganese pincer complex by Milstein and coworkers^57^. At this stage, intramolecular regioselective 1,2-insertion occurs to afford the complex **D**, which then undergoes concerted β-hydride elimination and releases N_2_ gas (Wolff-Kishner reductive denitrogenation) to deliver the desired product **4** and Ni–H species **E**. This metal-hydride releases H_2_ gas and regenerates the active Ni(0) catalyst to close the catalytic cycle. NaI as the additive enhances both the reactivity and E:Z selectivity, possibly through halide effects that assist the decomposition of intermediate **D** to produce thermodynamically more favored *trans*-alkene^65^. The allylic isomerization was due to the iterative Ni–H addition/elimination.

## Discussion

In summary, we have documented an efficient protocol for alkyl Heck-type reaction of vinylarenes with umpolung aldehydes under a simple nickel catalyst system. This strategy proceeds smoothly in absence of halides, organometallics or oxidant, liberating hydrogen, nitrogen, and water as innocuous side products. Excellent regioselectivity was achieved with the assistance of heteroatom chelation effect. Broad substrate scope, great functional group compatibility and modification of complex organic molecules containing olefin moieties made this methodology synthetically useful and valuable. Preliminary mechanistic studies revealed that the transformation did not proceed through carbene insertion, cyclopropane opening, nucleophilic addition/dehydrogenation or radical process. Detailed studies aimed at elucidating the mechanism and further applications of aldehydes as alkyl nucleophiles in chemical transformations are ongoing in our lab.

## Methods

### In situ preparation of hydrazone solution

A mixture of aldehydes **1** (0.6 mmol, 3.0 equiv) and hydrazine monohydrate (36 µL, 0.72 mmol, 64–65 wt%, 3.6 equiv) in THF (0.6 mL) solution was stirred for 30 min at room temperature in air. Before use, a small amount of anhydrous Na_2_SO_4_ and 4Ǻ MS was added.

### General procedure for dehydrogenative alkyl Heck-couplings

In a glovebox, a flame-dried reaction tube (10 cm^3^) equipped with a magnetic stir bar was charged with Ni(cod)_2_ (5.6 mg, 10 mol%), dcype (8.5 mg, 10 mol%) and THF (0.4 mL) before being sealed with a rubber septum. The reaction mixture was stirred at room temperature for 30 min. Then vinylarene **3** (0.2 mmol, 1.0 equiv), hydrazone solution **2** (0.6 mmol in 0.6 mL THF), DIPEA (0.4 mmol, 67 µL) and NaI (0.1 mmol, 15 mg) were added sequentially. After that, the reaction mixture was sealed with aluminum cap, moved out of glovebox and stirred at 100 °C for 12 h. After the mixture was cooled to rt, the resulting solution was directly filtered through a pad of silica by EtOAc (3.0 mL). The crude mixture was analyzed by GC-MS. The solvent was evaporated *in vacuo* to give the crude products. NMR yields were determined by ^1^H NMR using mesitylene as an internal standard. The residue was purified by preparative TLC (ethyl acetate/hexane) to give the pure product **4**.

### Notes

Use of the glovebox is not necessary unless to store and manipulate air-sensitive Ni(cod)_2_ catalyst. The other operations can be successfully performed outside the glovebox with standard Schlenk line procedure. One pot reaction of vinylarenes, aldehydes and hydrazine without preparation of hydrazones beforehand afforded only trace amount of desired products.

## Supplementary information

Supplementary Information

## Data Availability

The authors declare that the data supporting the findings of this study are available within the article and Supplementary Information file, or from the corresponding author upon reasonable request.
