# Supplementary material for: Direct dehydrogenative alkyl Heck-couplings of vinylarenes with umpolung aldehydes catalyzed by nickel
Source: Nat Commun. 2019 Feb 12;10:715. doi: 10.1038/s41467-019-08631-1 (PMC6372677; doi:10.1038/s41467-019-08631-1)
Supplement: Supplementary file 1 — Supplementary Information [file 41467_2019_8631_MOESM1_ESM.pdf]

Supporting Information

Direct Dehydrogenative Alkyl Heck-Couplings of Vinylarenes with Umpolung Aldehydes Catalyzed by Nickel

Lv et al

Supplementary Notes

^1H NMR spectra were recorded on Bruker 400 or 500 MHz spectrometer and the chemical shifts were reported in parts per million (δ) relative to internal solvent signal (7.28 ppm in $CDCl_3$). The peak patterns are indicated as follows: s, singlet; d, doublet; dd, doublet of doublet; t, triplet; q, quartet; m, multiplet. The coupling constants, *J*, are reported in Hertz (Hz). ^{13}C NMR spectra were obtained at 100 or 125 MHz and referenced to the internal solvent signal (central peak is 77.0 ppm in $CDCl_3$). $CDCl_3$ was used as the NMR solvent. Flash column chromatography was performed over silica gel 200-300. The reagent $Ni(cod)_2$ were weighed and handled in a glovebox. All reagents were purchased from Alfa, Acros, Aldrich, and TCI and used without further purification. The HRMS measurements were recorded on a TOF analyzer using an ESI or APCI source in the positive mode. [**Warning**: hydrazine monohydrate is potentially hazardous and should be performed with appropriate personal protection].

Supplementary Table 1 Optimization of the reaction conditions

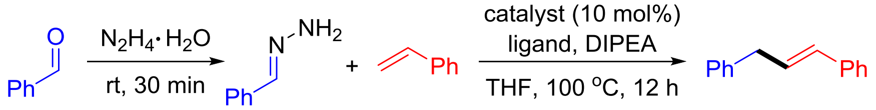

entry	catalyst	ligand	base	**4aa** (%)
1	Ni(cod)$_2$	PEt$_3$	DIPEA	N.D.
2	Ni(cod)$_2$	PBu$_3$	DIPEA	N.D.
3	Ni(cod)$_2$	dppf	DIPEA	N.D.
4	Ni(cod)$_2$	dmpe	DIPEA	N.D.
5	Ni(cod)$_2$	dppp	DIPEA	N.D.
6	Ni(cod)$_2$	Ruphos	DIPEA	trace
7	Ni(cod)$_2$	Davephos	DIPEA	N.D.
8	Ni(cod)$_2$	DPEphos	DIPEA	trace
9	Ni(cod)$_2$	X-phos	DIPEA	trace
10	Ni(cod)$_2$	Xanphos	DIPEA	N.D.
11	Ni(cod)$_2$	*rac*-BINAP	DIPEA	N.D.
12	Ni(cod)$_2$	cataCXium A	DIPEA	N.D.
13	Ni(cod)$_2$	SIPr·HCl	DIPEA	trace
14	Ni(cod)$_2$	IPr·HCl	DIPEA	trace
15	Ni(cod)$_2$	IPr·HCl	DIPEA	trace
16	Ni(cod)$_2$	bipy	DIPEA	trace
17	Ni(cod)$_2$	1,10-phenanthroline	DIPEA	N.D.
18	Ni(cod)$_2$	triphos	DIPEA	N.D.
19	Ni(cod)$_2$	dcype	^{t}BuOK	N.D.
20	Ni(cod)$_2$	dcype	K_3PO_4	N.D.
21	Ni(cod)$_2$	dcype	Cs_2CO_3	N.D.
22	Ni(cod)$_2$	dcype	DABCO	44 (79:21)
23	Ni(cod)$_2$	dcype	Et$_3$N	62 (77:23)
24	Ni(cod)$_2$	dcype	DBU	42 (82:18)
25	NiCl$_2$	dcype	DIPEA	trace
26	NiBr$_2$·diglyme	dcype	DIPEA	trace
27	Ni(acac)$_2$	dcype	DIPEA	10 (78:22)
28	Pd(OAc)$_2$	dcype	DIPEA	N.D.
29	CuCl$_2$	dcype	DIPEA	N.D.
30	CoCl$_2$	dcype	DIPEA	N.D.

Reaction conditions: **3a** (0.2 mmol), **1a** (0.6 mmol), N_2H_4•H_2O (0.72 mmol), Ni(cod)$_2$ (10 mol%), ligand (20 mol% for monodentate, 10 mol% for bidentate), base (0.4 mmol), THF (1.0 mL), 100 °C, 12 h under N_2 unless other noted. NMR yields were determined by ^{1}H NMR using mesitylene as an internal standard and based on **3a**. The *E*:Z ratio was determined by ^{1}H NMR analysis of the crude mixture. N.D.= not detected.

Supplementary Methods

Experimental procedures

In situ preparation of hydrazone solution **2**: a mixture of aldehydes **1** (0.6 mmol, 3.0 equiv) and hydrazine monohydrate (36 μL, 0.72 mmol, 64–65 wt%, 3.6 equiv) in THF (0.6 mL) solution was stirred for 30 min at room temperature in air. Before use, a small amount of anhydrous Na_2SO_4 and 4Å MS was added.

In a glovebox, a flame-dried reaction tube (10 cm^3) equipped with a magnetic stir bar was charged with Ni(cod)$_2$ (5.6 mg, 10 mol%), dcype (8.5 mg, 10 mol%) and THF (0.4 mL) before being sealed with a rubber septum. The reaction mixture was stirred at room temperature for 30 min. Then vinylarene **3** (0.2 mmol, 1.0 equiv), hydrazone solution **2** (0.6 mmol in 0.6 mL THF), DIPEA (0.4 mmol, 67 μL) and NaI (0.1 mmol, 15 mg) was added sequentially. After that, the reaction mixture was sealed with aluminum cap, moved out of glovebox, and stirred at 100 ℃ for 12 hour. After the mixture was cooled to room temperature, the resulting solution was directly filtered through a pad of silica by EtOAc (3.0 mL). The crude mixture was analyzed by GC-MS. The solvent was evaporated *in vacuo* to give the crude products. NMR yields were determined by ^{1}H NMR using mesitylene as an internal standard. The residue was purified by preparative TLC (ethyl acetate/petroleum ether) to give the pure product.

Data analyst

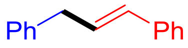

(*E*)-Prop-1-ene-1,3-diyldibenzene (4aa). (34 mg, 87%). Isolated by preparative TLC (hexane, R_f = 0.7); ^{1}H NMR (500 MHz, $CDCl_3$) δ 7.43-7.23 (m, 10H), 6.52 (d, J = 16.0 Hz, 1H), 6.45-6.38 (m, 1H), 3.61 (d, J = 7.0 Hz, 2H); ^{13}C NMR (125 MHz, $CDCl_3$) δ 140.2, 137.5, 131.1, 129.3, 128.7, 128.5, 127.1, 126.2, 126.1, 39.4; EI-MS (m/z): 194.2. The spectroscopic data for this product match the literature data.[1]

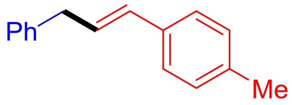

(*E*)-1-Methyl-4-(3-phenylprop-1-en-1-yl)benzene (4ab). (37 mg, 89%). Isolated by preparative TLC (hexane, R_f = 0.6); ^{1}H NMR (500 MHz, $CDCl_3$) δ 7.42-7.13 (m, 9H), 6.47 (d, J = 15.8 Hz, 1H), 6.40-6.32 (m, 1H), 3.58 (d, J = 6.8 Hz, 2H), 2.36 (s, 3H); ^{13}C NMR (125 MHz, $CDCl_3$) δ 140.3, 136.8, 134.7, 130.9, 129.2, 128.7, 128.5, 128.2, 126.1, 126.0, 39.4, 21.2; EI-MS (m/z): 208.1. The spectroscopic data for this product match the literature data.[2]

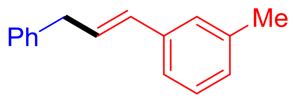

(*E*)-1-Methyl-3-(3-phenylprop-1-en-1-yl)benzene (4ac). (41 mg, 84%). Isolated by preparative TLC (hexane, R_f = 0.5); ^{1}H NMR (500 MHz, $CDCl_3$) δ 7.36-7.31 (m, 3H), 7.27-7.05 (m, 6H), 6.46 (d, J = 15.6 Hz, 1H), 6.42-6.35 (m, 1H), 3.58 (d, J = 6.6 Hz, 2H), 2.36 (s, 3H); ^{13}C NMR (125 MHz, $CDCl_3$) δ 140.3, 138.1, 137.4, 131.1, 129.0, 128.6, 128.5, 128.4, 127.9, 126.8, 126.1, 123.3, 39.4, 21.4; EI-MS (m/z): 208.1. The spectroscopic data for this product match the literature data.[2]

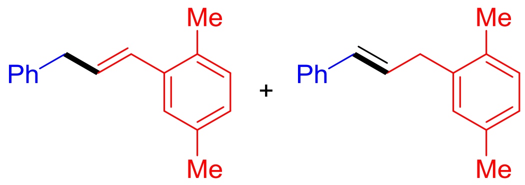

(*E*)-1,4-Dimethyl-2-(3-phenylprop-1-en-1-yl)benzene + 2-cinnamyl-1,4-dimethylbenzene (4ad). (combined 39.1 mg, 88%, 57:43). Isolated by preparative TLC (hexane, R_f = 0.5); **Major and minor**: ^{1}H NMR (500 MHz, $CDCl_3$) δ 7.39-7.22 (m, 6H), 7.11-7.05 (m, 1H), 7.01-6.97 (m, 1H), 6.69 (d, J = 15.6 Hz, 0.4H), 6.44-6.22 (m, 1.6H), 3.61 (d, J = 6.8 Hz, 1H), 3.53 (d, J = 5.3 Hz, 1H), 2.34 (s, 3.1H), 2.33 (s, 2.9H); **Major and minor**: ^{13}C NMR (125 MHz, $CDCl_3$) δ 140.4, 138.0, 137.6, 136.3, 135.5, 135.3, 133.2, 132.0, 130.8, 130.2, 130.1, 130.2, 129.1, 128.7, 128.5, 127.8, 127.1, 127.2, 126.2, 126.1, 39.7, 36.9, 21.0, 19.4, 19.0; EI-MS (m/z): 222.1. The spectroscopic data for this product match the literature data.[3]

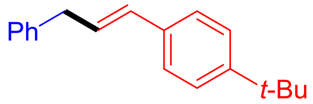

(*E*)-1-(*Tert*-butyl)-4-(3-phenylprop-1-en-1-yl)benzene (4ae). (44.5 mg, 89%). Isolated by preparative TLC (hexane, R_f = 0.6); ^{1}H NMR (500 MHz, $CDCl_3$) δ 7.38-7.23 (m, 9H), 6.49 (d, J = 15.8 Hz, 1H), 6.43-6.34 (m, 1H), 3.59 (d, J = 7.4 Hz, 2H), 1.36 (s, 9H); ^{13}C NMR (125 MHz, $CDCl_3$) δ 150.2, 140.4, 134.7, 130.9, 128.7, 128.5, 128.4, 126.2, 125.9, 125.5, 39.4, 34.5, 31.3; EI-MS (m/z): 250.2. The spectroscopic data for this product match the literature data.[2]

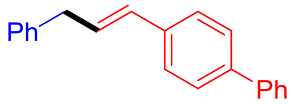

(*E*)-4-(3-Phenylprop-1-en-1-yl)-1,1'-biphenyl (4af). (43.8 mg, 81%). Isolated by preparative TLC (hexane, R_f = 0.5); ^{1}H NMR (500 MHz, $CDCl_3$) δ 7.65-7.58 (m, 5H), 7.49-7.46 (m, 3H), 7.40-7.35 (m, 4H), 7.33-7.30 (m, 2H), 6.55 (d, J = 15.8 Hz, 1H), 6.49-6.43 (m, 1H), 3.63 (d, J = 7.0 Hz, 2H); ^{13}C NMR (125 MHz, $CDCl_3$) δ 140.8, 140.2, 139.9, 136.6, 130.7, 129.5, 128.8, 128.7, 128.6, 127.3, 126.9, 126.6, 126.3, 39.5; EI-MS (m/z): 270.2. The spectroscopic data for this product match the literature data.[2]

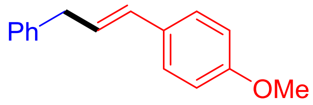

(*E*)-1-Methoxy-4-(3-phenylprop-1-en-1-yl)benzene (4ag). (38.5 mg, 86%). Isolated by preparative TLC (hexane: ethyl acetate = 50:1, R_f = 0.5); ^{1}H NMR (500 MHz, $CDCl_3$) δ 7.37-7.24 (m, 7H), 6.88 (d, J = 8.7 Hz, 2H), 6.45 (d, J = 15.6 Hz, 1H), 6.29-6.24 (m, 1H), 3.84 (s, 3H), 3.58 (d, J = 6.8 Hz, 2H); ^{13}C NMR (125 MHz, $CDCl_3$) δ 158.9, 140.5, 130.5, 130.4, 128.7, 128.6, 127.3, 127.1, 126.2, 113.9, 55.4, 39.4; EI-MS (m/z): 224.1. The spectroscopic data for this product match the literature data.[2]

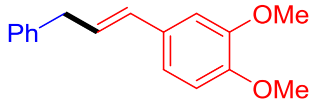

(*E*)-1,2-Dimethoxy-4-(3-phenylprop-1-en-1-yl)benzene (4ah). (44.2 mg, 87%). Isolated by preparative TLC (hexane: ethyl acetate = 20:1, R_f = 0.5); ^{1}H NMR (500 MHz, $CDCl_3$) δ 7.41-7.26 (m, 6H), 6.96-6.80 (m, 2H), 6.44 (d, J = 15.7 Hz, 1H), 6.30-6.24 (m, 1H), 3.91 (s, 3H), 3.90 (s, 3H), 3.58 (d, J = 6.8 Hz, 2H); ^{13}C NMR (125 MHz, $CDCl_3$) δ 149.0, 148.5, 140.4, 130.9, 130.7, 128.7, 128.5, 127.4, 126.2, 119.2, 111.2, 108.6, 56.0, 55.8, 39.4; EI-MS (m/z): 254.1. The spectroscopic data for this product match the literature data.[2]

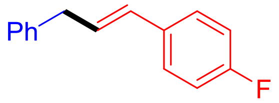

(*E*)-1-Fluoro-4-(3-phenylprop-1-en-1-yl)benzene (4ai). (40.7 mg, 96%). Isolated by preparative TLC (hexane, R_f = 0.6); ^{1}H NMR (500 MHz, $CDCl_3$) δ 7.38-7.33 (m, 4H), 7.30-7.24 (m, 3H), 7.06-7.01 (m, 2H), 6.46 (d, J = 15.8 Hz, 1H), 6.32 (dt, J = 15.8, 6.7 Hz, 1H), 3.59 (d, J = 6.7 Hz, 2H); ^{13}C NMR (125 MHz, $CDCl_3$) δ 162.0 (d, $J_{C\text{-}F}$ = 244.6 Hz), 140.0, 133.6 (d, $J_{C\text{-}F}$ = 3.1 Hz), 129.8, 129.0, 128.6, 128.5, 127.5 (d, $J_{C\text{-}F}$ = 7.8 Hz), 126.2, 115.3 (d, $J_{C\text{-}F}$ = 21.4 Hz), 39.3; ^{19}F NMR (470 MHz, $CDCl_3$) δ -115.3; EI-MS (m/z): 212.1. The spectroscopic data for this product match the literature data.[2]

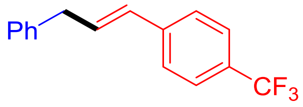

(*E*)-1-(3-Phenylprop-1-en-1-yl)-4-(trifluoromethyl)benzene (4aj). (41.4 mg, 79%). Isolated by preparative TLC (hexane, R_f = 0.6); ^{1}H NMR (500 MHz, $CDCl_3$) δ 7.56 (d, J = 8.2 Hz, 2H), 7.46 (d, J = 8.2 Hz, 2H), 7.39-7.25 (m, 5H), 6.52-6.46 (m, 2H), 3.60 (d, J = 4.0 Hz, 2H); ^{13}C NMR (125 MHz, $CDCl_3$) δ 140.9, 139.6, 132.1, 129.8, 128.9 (q, $J_{C\text{-}F}$ = 32.4 Hz), 128.7, 128.6, 126.4, 126.3, 125.5 (q, $J_{C\text{-}F}$ = 3.8 Hz), 124.3 (q, $J_{C\text{-}F}$ = 271.8 Hz), 39.4; ^{19}F NMR (470 MHz, $CDCl_3$) δ -62.5; EI-MS (m/z): 262.1. The spectroscopic data for this product match the literature data.[2]

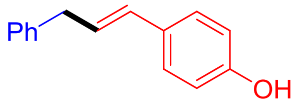

(*E*)-4-(3-phenylprop-1-en-1-yl)phenol (4ak). (29 mg, 69%). Isolated by preparative TLC (hexane: ethyl acetate = 3:1, R_f = 0.5); ^{1}H NMR (400 MHz, $CDCl_3$) δ 7.40-7.33 (m, 3H), 7.29-7.25 (m, 3H), 6.83-6.78 (m, 3H), 6.43 (d, J = 15.8 Hz, 1H), 6.24 (dt, J = 15.8, 6.8 Hz, 1H), 4.88 (br, 1H), 3.56 (d, J = 6.8 Hz, 2H); ^{13}C NMR (125 MHz, $CDCl_3$)

δ 154.7, 140.5, 130.4, 129.8, 128.7, 128.5, 127.5, 127.2, 126.1, 115.4, 39.3; EI-MS (m/z): 210.1. The spectroscopic data for this product match the literature data.[4]

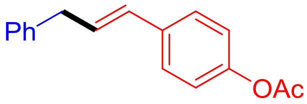

(*E*)-4-(3-Phenylprop-1-en-1-yl)phenyl acetate (4al). (32.3 mg, 64%). Isolated by preparative TLC (hexane:ethyl acetate = 20:1, R_f = 0.5); ^{1}H NMR (500 MHz, $CDCl_3$) δ 7.40-7.38 (m, 2H), 7.36-7.24 (m, 5H), 7.06-7.04 (m, 2H), 6.47 (d, J = 15.6 Hz, 1H), 6.40-6.31 (m, 1H), 3.57 (d, J = 6.7 Hz, 2H), 2.32 (s, 3H); ^{13}C NMR (125 MHz, $CDCl_3$) δ 169.5, 149.7, 140.1, 135.3, 130.1, 129.6, 129.5, 128.7, 128.5, 127.0, 126.1, 121.6, 39.3, 21.1; EI-MS (m/z): 252.1. The spectroscopic data for this product match the literature data.[5]

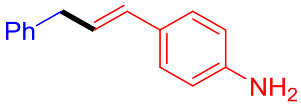

(*E*)-4-(3-Phenylprop-1-en-1-yl)aniline (4am). (31 mg, 74%). Isolated by preparative TLC (hexane:ethyl acetate = 3:1, R_f = 0.4); ^{1}H NMR (500 MHz, $CDCl_3$) δ 7.35-7.22 (m, 5H), 7.21 (d, J = 8.4 Hz, 2H), 6.65 (d, J = 8.4 Hz, 2H), 6.40 (d, J = 15.6 Hz, 1H), 6.19 (dt, J = 15.6, 6.8 Hz, 1H), 3.67 (br, 2H), 3.55 (d, J = 6.8 Hz, 2H); ^{13}C NMR (125 MHz, $CDCl_3$) δ 145.6, 140.7, 130.8, 128.7, 128.5, 128.3, 127.2, 126.0, 125.6, 115.2, 39.4; EI-MS (m/z): 209.1. The spectroscopic data for this product match the literature data.[6]

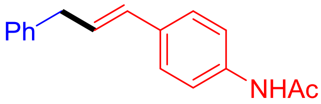

(*E*)-*N*-(4-(3-Phenylprop-1-en-1-yl)phenyl)acetamide (4an). (46.7 mg, 93%). Isolated by preparative TLC (dichloromethane: methanol = 30:1, R_f = 0.4); ^{1}H NMR (500 MHz, $CDCl_3$) δ 7.46 (d, J = 8.4 Hz, 2H), 7.42 (br, 1H), 7.38-7.30 (m, 4H), 7.27 (d, J = 8.4 Hz, 2H), 7.25-7.20 (m, 1H), 6.43 (d, J = 15.6 Hz, 1H), 6.38-6.28 (m, 1H), 3.56 (d, J = 6.8 Hz, 2H), 2.18 (s, 3H); ^{13}C NMR (125 MHz, $CDCl_3$) δ 168.3, 140.2, 136.9, 133.7, 130.4, 129.2, 128.7, 128.5, 126.7, 126.2, 119.9, 39.3, 24.6; EI-MS (m/z): 251.1. The spectroscopic data for this product match the literature data.[6]

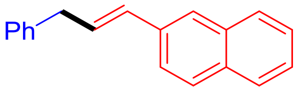

(*E*)-2-(3-Phenylprop-1-en-1-yl)naphthalene (4ao). (41 mg, 84%). Isolated by preparative TLC (hexane, R_f = 0.5); ^{1}H NMR (500 MHz, $CDCl_3$) δ 7.83-7.79 (m, 3H), 7.74 (s, 1H), 7.62 (dd, J = 8.6, 1.6 Hz, 1H), 7.50-7.25 (m, 7H), 6.66 (d, J = 15.7 Hz, 1H), 6.57-6.50 (m, 1H), 3.65 (d, J = 6.8 Hz, 2H); ^{13}C NMR (125 MHz, $CDCl_3$) δ 140.2, 135.0, 133.7, 132.8, 131.2, 129.8, 128.8, 128.6, 128.2, 127.9, 127.7, 126.3, 126.2, 125.8, 125.6, 123.6, 39.5; EI-MS (m/z): 244.1. The spectroscopic data for this product match the literature data.[2]

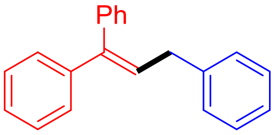

Prop-1-ene-1,1,3-triyltribenzene (4ap). (25.9 mg, 48%). Isolated by preparative TLC (hexane, R_f = 0.6); ^{1}H NMR (500 MHz, $CDCl_3$) δ 7.44-7.41 (m, 2H), 7.38-7.22 (m, 13H), 6.30 (t, J = 7.6 Hz, 1H), 3.51 (d, J = 7.6 Hz, 2H); ^{13}C NMR (125 MHz, $CDCl_3$) δ 142.5, 142.4, 141.0, 139.8, 129.9, 128.5, 128.4, 128.3, 128.1, 127.8, 127.4, 127.2, 127.1, 126.0, 35.9; EI-MS (m/z): 270.1. The spectroscopic data for this product match the literature data.[7]

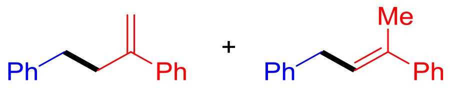

But-3-ene-1,3-diyldibenzene (4aq) and (*E*)-But-2-ene-1,3-diyldibenzene (4aq'). (combined 26.6 mg, 64%, 60:40). Isolated by preparative TLC (hexane, R_f = 0.7); **Major (4aq)**: ^{1}H NMR (500 MHz, $CDCl_3$) 7.52-7.23 (m, 10H), 5.36 (d, J = 0.9 Hz, 1H), 5.13 (d, J = 0.9 Hz, 1H), 2.91-2.81 (m, 4H); ^{13}C NMR (125 MHz, $CDCl_3$) δ 147.8, 141.9, 141.0, 128.4, 128.3, 127.4, 126.1, 125.8, 112.7, 37.2, 34.7. **Minor (4aq')**: ^{1}H NMR (500 MHz, $CDCl_3$) δ 7.52-7.25 (m, 10H), 6.04 (tq, J = 7.4, 1.3 Hz, 1H), 3.64 (d, J = 7.4 Hz, 2H), 2.21 (s, 3H); ^{13}C NMR (125 MHz, $CDCl_3$) δ 143.6, 141.1, 135.7, 128.4, 128.3, 126.7, 126.6, 125.9, 125.7, 34.9, 16.0; EI-MS (m/z): 208.1. The spectroscopic data for this product match the literature data.[6]

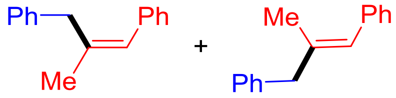

(*Z*)-(2-Methylprop-1-ene-1,3-diyl)dibenzene (4ar) and (*E*)-(2-Methylprop-1-ene-1,3-diyl)dibenzene (4ar'). (combined 14.6 mg, 35%, 67:33). Isolated by preparative TLC (hexane, R_f = 0.7); **Major**: ^{1}H NMR (500 MHz, $CDCl_3$) 7.35-7.30 (m, 6H), 7.25-7.21 (m, 4H), 6.55 (s, 1H), 3.64 (s, 2H), 1.84 (s, 3H); ^{13}C NMR (125 MHz, $CDCl_3$) δ 139.7, 138.2, 137.0, 128.6, 128.5, 128.4, 128.2, 127.4, 126.3, 126.0, 38.5, 24.0; **Minor**: ^{1}H NMR (500 MHz, $CDCl_3$) δ 7.35-7.30 (m, 6H), 7.25-7.21 (m, 4H), 6.41 (s, 1H), 3.51 (s, 2H), 1.83 (s, 3H); ^{13}C NMR (125 MHz, $CDCl_3$) δ 139.8, 138.4, 138.1, 129.0, 128.9, 128.3, 128.0, 126.8, 126.2, 126.1, 47.1, 17.6; EI-MS (m/z): 208.1. The spectroscopic data for this product match the literature data.[8]

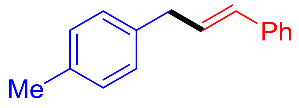

1-Cinnamyl-4-fluorobenzene (4ba). (38.3 mg, 92%). Isolated by preparative TLC (hexane, R_f = 0.7); ^{1}H NMR (500 MHz, $CDCl_3$) δ 7.43-7.13 (m, 9H), 6.48 (d, J = 15.8 Hz, 1H), 6.38 (dt, J = 15.8, 6.7 Hz, 1H), 3.55 (d, J = 6.7 Hz, 2H), 2.37 (s, 3H); ^{13}C NMR (125 MHz, $CDCl_3$) δ 137.5, 137.1, 135.7, 130.8, 129.5, 129.2, 128.5, 127.0, 126.1, 38.9, 21.0; EI-MS (m/z): 208.1. The spectroscopic data for this product match the literature data.[9]

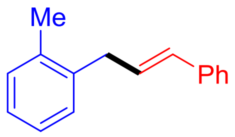

1-Cinnamyl-2-methylbenzene (4bb). (37.0 mg, 89%). Isolated by preparative TLC (hexane, R_f = 0.7); ^{1}H NMR (500 MHz, $CDCl_3$) δ 7.37 (d, J = 7.6 Hz, 2H), 7.32 (t, J = 7.6 Hz, 2H), 7.24-7.19 (m, 5H), 6.44-6.34 (m, 2H), 3.57 (d, J = 5.1 Hz, 2H), 2.37 (s, 3H); ^{13}C NMR (125 MHz, $CDCl_3$) δ 138.2, 137.5, 136.4, 131.0, 130.2, 129.2, 128.8,

128.7, 127.0, 126.4, 126.1, 126.0, 36.8, 19.4; EI-MS (m/z): 208.1. The spectroscopic data for this product match the literature data.[9]

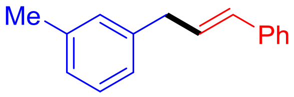

1-Cinnamyl-3-methylbenzene (4bc). (39.1 mg, 94%). Isolated by preparative TLC (hexane, R_f = 0.7); ^{1}H NMR (500 MHz, $CDCl_3$) δ 7.40 (dd, J = 7.6 Hz, 1.2 Hz, 2H), 7.33 (t, J = 7.6 Hz, 2H), 7.24 (t, J = 7.6 Hz, 3H), 7.10-7.06 (m, 2H), 6.50 (d, J = 15.8 Hz, 1H), 6.39 (dt, J = 16.0 Hz, 6.8 Hz, 1H), 3.56 (d, J = 6.8 Hz, 2H), 2.38 (s, 3H); ^{13}C NMR (125 MHz, $CDCl_3$) δ 140.1, 138.1, 137.1, 131.0, 129.4, 129.3, 128.5, 128.4, 127.1, 126.9, 126.1, 125.7, 39.3, 21.4; EI-MS (m/z): 208.1. The spectroscopic data for this product match the literature data.[9]

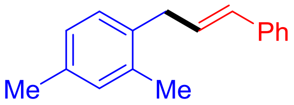

1-Cinnamyl-2,4-dimethylbenzene (4bd). (39.1 mg, 88%). Isolated by preparative TLC (hexane, R_f = 0.7); ^{1}H NMR (500 MHz, $CDCl_3$) δ 7.37 (d, J = 7.3 Hz, 2H), 7.31 (t, J = 7.3 Hz, 2H), 7.24-7.20 (m, 1H), 7.12 (d, J = 7.6 Hz, 1H), 7.03-6.99 (m, 2H), 6.43-6.36 (m, 2H), 3.53 (d, J = 5.2 Hz, 2H), 2.34 (s, 3H), 2.33 (s, 3H); ^{13}C NMR (125 MHz, $CDCl_3$) δ 137.6, 136.2, 135.8, 135.1, 131.0, 130.7, 129.2, 128.8, 128.4, 127.0, 126.7, 126.0, 36.5, 20.9, 19.3; EI-MS (m/z): 222.1. The spectroscopic data for this product match the literature data.[10]

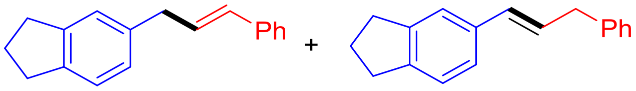

5-Cinnamyl-2,3-dihydro-1H-indene (4be) and . (*E*)-5-(3-phenylprop-1-en-1-yl)-2,3-dihydro-1H-indene (4be') (combined 40.3 mg, 86%, 71:29). Isolated by preparative TLC (hexane, R_f = 0.7); **Major and minor**: ^{1}H NMR (500 MHz, $CDCl_3$) δ 7.41-7.29 (m, 4H), 7.25-7.16 (m, 4H), 6.50 (d, J = 15.8 Hz, 1H), 6.39 (dt, J = 15.8, 6.6 Hz, 1H), 3.56 (d, J = 6.6 Hz, 2H), 2.94-2.90 (m, 4H), 2.13-2.09 (m, 2H); **Major and minor:** ^{13}C NMR (125 MHz, $CDCl_3$) δ 144.7, 144.6, 143.4, 142.1, 140.5, 138.0, 137.6, 135.6, 131.4, 130.7, 129.8, 128.7, 128.5, 127.0, 126.5, 126.1, 124.8, 124.3, 122.0, 121.9, 39.4, 39.3, 32.8, 32.7, 32.6, 32.5, 25.6, 25.5; EI-MS (m/z): 234.1. The spectroscopic data for this product match the literature data.[10]

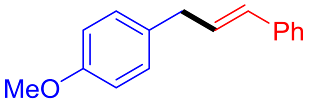

1-Cinnamyl-4-methoxybenzene (4bf). (36.7 mg, 82%). Isolated by preparative TLC (hexane: ethyl acetate = 40:1, R_f = 0.5); ^{1}H NMR (500 MHz, $CDCl_3$) δ 7.34 (d, J = 8.0 Hz, 2H), 7.29 (d, J = 8.0 Hz, 2H), 7.25-7.19 (m, 3H), 6.90 (d, J = 7.2 Hz, 2H), 6.47 (d, J = 15.8 Hz, 1H), 6.41-6.35 (m, 1H), 3.84 (s, 3H), 3.53 (d, J = 6.6 Hz, 2H); ^{13}C NMR (125 MHz, $CDCl_3$) δ 158.1, 137.6, 132.2, 130.8, 129.7, 129.6, 128.5, 127.2, 126.1, 113.9, 55.3, 38.5; EI-MS (m/z): 224.1. The spectroscopic data for this product match the literature data.[9]

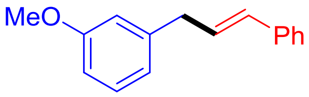

1-Cinnamyl-3-methoxybenzene (4bg). (36.3 mg, 81%). Isolated by preparative TLC (hexane: ethyl acetate =60:1, R_f = 0.5); ^{1}H NMR (500 MHz, $CDCl_3$) δ 7.41-7.24 (m, 6H), 7.01-6.81 (m, 3H), 6.50 (d, J = 15.8 Hz, 1H), 6.40 (dt, J = 15.8, 6.8 Hz, 1H), 3.84 (s, 3H), 3.57 (d, J = 6.8 Hz, 2H); ^{13}C NMR (125 MHz, $CDCl_3$) δ 159.8, 141.8, 137.5, 131.2, 129.5, 129.1, 128.5, 127.2, 126.2, 121.1, 114.4, 111.6, 55.2, 39.4; EI-MS (m/z): 224.1. The spectroscopic data for this product match the literature data.[11]

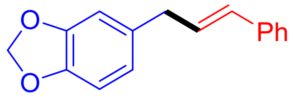

5-Cinnamylbenzo[d][1,3]dioxole (4bh). (34.3 mg, 72%). Isolated by preparative TLC (hexane: ethyl acetate = 20:1, R_f = 0.5); ^{1}H NMR (500 MHz, $CDCl_3$) δ 7.38 (d, J = 7.6 Hz, 2H), 7.32 (t, J = 7.6 Hz, 2H), 7.27-7.22 (m, 1H), 6.82-6.71 (m, 3H), 6.47 (d, J = 15.8 Hz, 1H), 6.35 (dt, J = 15.8, 6.7 Hz, 1H), 5.96 (s, 2H), 3.49 (d, J = 6.7 Hz, 2H); ^{13}C NMR (125 MHz, $CDCl_3$) δ 147.7, 145.9, 137.4, 133.9, 131.0, 129.3, 128.5, 127.1, 126.1, 121.4, 109.2, 108.2, 100.8, 39.0; EI-MS (m/z): 238.1. The spectroscopic data for this product match the literature data.[12]

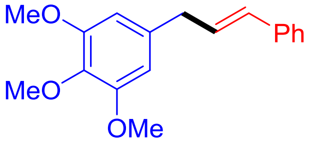

5-Cinnamyl-1,2,3-trimethoxybenzene (4bi). (44.3 mg, 78%). Isolated by preparative TLC (hexane: ethyl acetate = 8:1, R_f = 0.4); ^{1}H NMR (500 MHz, $CDCl_3$) δ 7.40 (d, J = 8.0 Hz, 2H), 7.34-7.31 (m, 2H), 7.25-7.21 (m, 1H), 6.50 (d, J = 15.6 Hz, 1H), 6.49 (m, 2H), 6.41-6.34 (m, 1H), 3.89 (s, 3H), 3.88 (s, 3H), 3.87 (s, 3H), 3.52 (d, J = 6.4 Hz, 2H); ^{13}C NMR (125 MHz, $CDCl_3$) δ 153.3, 137.4, 136.4, 135.9, 131.2, 129.0, 128.6, 127.2, 126.2, 105.6, 60.9, 56.1, 39.7; EI-MS (m/z): 284.1. The spectroscopic data for this product match the literature data.[13]

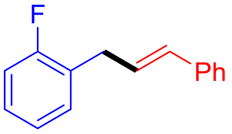

1-Cinnamyl-2-fluorobenzene (4bj). (39.4 mg, 93%). Isolated by preparative TLC (hexane, R_f = 0.7); ^{1}H NMR (500 MHz, $CDCl_3$) δ 7.42-7.22 (m, 7H), 7.15-7.04 (m, 2H), 6.51 (d, J = 15.8 Hz, 1H), 6.39 (dt, J = 15.8, 6.6 Hz, 1H), 3.62 (d, J = 6.6 Hz, 2H); ^{13}C NMR (125 MHz, $CDCl_3$) δ 161.0 (d, $J_{C\text{-}F}$ = 244.2 Hz), 137.3, 131.4, 130.6 (d, $J_{C\text{-}F}$ = 4.8 Hz), 128.5, 127.9 (d, $J_{C\text{-}F}$ = 8.4 Hz), 127.6, 127.2, 127.1 (d, $J_{C\text{-}F}$ = 16.7 Hz), 126.1, 32.2 (d, $J_{C\text{-}F}$ = 3.3 Hz), 124.1 (d, $J_{C\text{-}F}$ = 3.6 Hz), 115.3 (d, $J_{C\text{-}F}$ = 21.5 Hz), 32.3; ^{19}F NMR (470 MHz, $CDCl_3$) δ -118.4; EI-MS (m/z): 212.1. The spectroscopic data for this product match the literature data.[14]

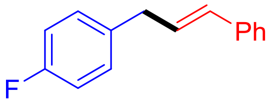

1-Cinnamyl-4-fluorobenzene (4bk). (40.7 mg, 96%). Isolated by preparative TLC (hexane, R_f = 0.6); ^{1}H NMR (500 MHz, $CDCl_3$) δ 7.41-7.22 (m, 7H), 7.06-7.01 (m, 2H), 6.48 (d, J = 15.6 Hz, 1H), 6.36 (dt, J = 15.6, 6.8 Hz,

1H), 3.56 (d, J = 6.8 Hz, 2H); ^{13}C NMR (125 MHz, $CDCl_3$) δ 161.5 (d, $J_{C\text{-}F}$ = 243.3 Hz), 137.4, 135.8 (d, $J_{C\text{-}F}$ = 3.9 Hz), 131.3, 130.0 (d, $J_{C\text{-}F}$ = 7.7 Hz), 129.0, 128.6, 127.3, 126.2, 115.3 (d, $J_{C\text{-}F}$ = 21.0 Hz), 38.5; ^{19}F NMR (470 MHz, $CDCl_3$) δ -117.2; EI-MS (m/z): 212.1. The spectroscopic data for this product match the literature data.[1]

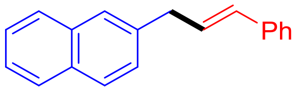

2-Cinnamylnaphthalene (4bl). (37.1 mg, 76%). Isolated by preparative TLC (hexane, R_f = 0.6); ^{1}H NMR (500 MHz, $CDCl_3$) δ 7.86-7.84 (m, 3H), 7.72 (s, 1H), 7.50-7.45 (m, 2H), 7.42-7.40 (m, 3H), 7.32 (t, J = 7.5 Hz, 2H), 7.24 (t, J = 7.3 Hz, 1H), 6.54 (d, J = 15.8 Hz, 1H), 6.47 (dt, J = 15.8, 6.2 Hz, 1H), 3.75 (d, J = 6.2 Hz, 2H); ^{13}C NMR (125 MHz, $CDCl_3$) δ 137.7, 137.5, 133.7, 132.2, 131.3, 129.1, 128.5, 128.0, 127.6, 127.5, 127.4, 127.1, 126.7, 126.2, 126.0, 125.3, 39.5; EI-MS (m/z): 244.1. The spectroscopic data for this product match the literature data.[12]

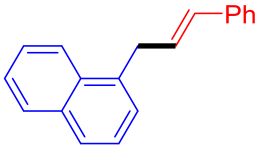

1-Cinnamylnaphthalene (4bm). (31.7 mg, 65%). Isolated by preparative TLC (hexane, R_f = 0.7); ^{1}H NMR (500 MHz, $CDCl_3$) δ 8.19-8.13 (m, 1H), 7.93-7.88 (m, 1H), 7.82-7.79 (m, 1H), 7.58-7.22 (m, 9H), 6.59-6.41 (m, 2H), 4.05 (d, J = 4.8 Hz, 2H); ^{13}C NMR (125 MHz, $CDCl_3$) δ 137.5, 136.3, 133.9, 132.1, 131.3, 128.9, 128.7, 128.5, 127.1, 126.4, 126.1, 125.9, 125.6, 125.5, 124.0, 36.5; EI-MS (m/z): 244.1. The spectroscopic data for this product match the literature data.[14]

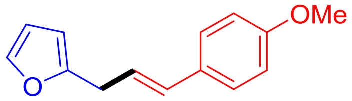

(*E*)-2-(3-(4-Methoxyphenyl)allyl)furan (4bn). (29.1 mg, 68%). Isolated by preparative TLC (hexane: ethyl acetate = 60:1, R_f = 0.6); ^{1}H NMR (500 MHz, $CDCl_3$) δ 7.37 (dd, J = 0.8, 1.8 Hz, 1H), 7.33 (d, J = 8.8 Hz, 2H), 6.87 (d, J = 8.8 Hz, 2H), 6.47 (d, J = 15.7 Hz, 1H), 6.33 (dd, J = 1.9, 3.1 Hz, 1H), 6.19 (dt, J = 15.7, 6.8 Hz, 1H), 6.09 (dd, J = 0.8, 3.1 Hz, 1H), 3.83 (s, 3H), 3.56 (d, J = 6.8 Hz, 2H); ^{13}C NMR (125 MHz, $CDCl_3$) δ 159.0, 154.2, 141.3, 131.4, 130.1, 127.3, 123.4, 113.9, 110.3, 105.5, 55.3, 31.8; EI-MS (m/z): 214.1. The spectroscopic data for this product match the literature data.[15]

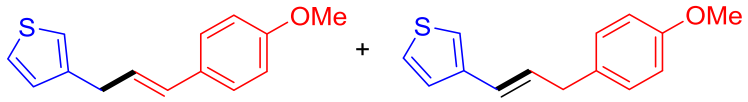

(*E*)-3-(3-(4-Methoxyphenyl)allyl)thiophene (4bn) and (*E*)-3-(3-(4-Methoxyphenyl)prop-1-en-1-yl) thiophene (4bn'). (combined 17.5 mg, 38%, 40:60). Isolated by preparative TLC (hexane: ethyl acetate = 60:1, R_f = 0.5); **Major and minor**: ^{1}H NMR (500 MHz, $CDCl_3$) δ 7.33-7.31 (m, 1H), 7.31-7.29 (m, 1H), 7.27-7.26 (m, 1H), 7.04-7.02 (m, 1H), 7.02-6.99 (m, 1H), 6.91-6.90 (m, 1H), 6.88-6.86 (m, 1H), 6.54 (d, J =11.4 Hz, 0.6H), 6.43 (d, J =15.8 Hz, 0.4H), 6.25 (dt, J =15.8, 6.8 Hz, 0.4H), 5.83 (dt, J =11.4, 7.4 Hz, 0.6H), 3.84 (s, 1.8 H), 3.83 (s, 1.2 H), 3.68 (d, J = 7.4 Hz, 1.2H), 3.56 (d, J = 6.8 Hz, 0.8H); **Major and minor**: ^{13}C NMR (125 MHz, $CDCl_3$) δ 158.9,

158.5, 141.1, 140.9, 130.4, 130.3, 129.9, 129.8, 129.5, 128.5, 128.4, 128.2, 127.2, 126.4, 125.6, 125.5, 120.8, 120.4, 114.0, 113.7, 55.3, 33.8, 29.5; EI-MS (m/z): 230.1. The spectroscopic data for this product match the literature data.[15,16]

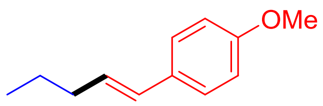

(*E*)-1-Methoxy-4-(pent-1-en-1-yl)benzene (4bp). (14.8 mg, 42%). Isolated by preparative TLC (hexane: ethyl acetate = 150:1, R_f = 0.6); ^{1}H NMR (500 MHz, $CDCl_3$) δ 7.30 (d, J = 8.8 Hz, 2H), 6.86 (d, J = 8.8 Hz, 2H), 6.34 (d, J =15.8 Hz, 1H), 6.10 (dt, J =15.8, 6.8 Hz, 1H), 3.82 (s, 3H), 2.19 (ddd, J =15.8, 7.0, 1.3 Hz, 2H), 1.50 (dq, J =14.5, 7.2 Hz, 2H), 0.97 (t, J =7.2 Hz, 3H); ^{13}C NMR (125 MHz, $CDCl_3$) δ 158.6, 130.8, 129.2, 128.9, 127.0, 113.9, 55.3, 35.1, 22.7, 13.7; EI-MS (m/z): 176.1. The spectroscopic data for this product match the literature data.[16]

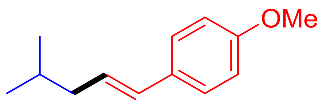

(*E*)-1-Methoxy-4-(4-methylpent-1-en-1-yl)benzene (4bq). (14.8 mg, 39%). Isolated by preparative TLC (hexane: ethyl acetate =100:1, R_f = 0.5); ^{1}H NMR (500 MHz, $CDCl_3$) δ 7.30 (t, J = 8.7 Hz, 2H), 6.86 (d, J = 8.7 Hz, 2H), 6.33 (d, J = 15.7 Hz, 1H), 6.10 (dt, J = 15.7, 7.2 Hz, 1H), 3.83 (s, 3H), 2.10 (td, J = 7.2, 1.3 Hz, 2H), 1.77-1.69 (m, 1H), 0.96 (d, J = 6.6 Hz, 6H); ^{13}C NMR (125 MHz, $CDCl_3$) δ 158.6, 130.8, 130.1, 127.7, 127.0, 113.9, 55.3, 42.4, 28.7, 22.4; EI-MS (m/z): 190.1. The spectroscopic data for this product match the literature data.[17]

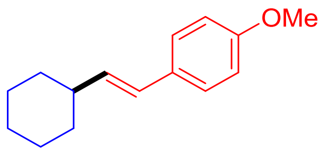

(*E*)-1-(2-Cyclohexylvinyl)-4-methoxybenzene (4br). (17.3 mg, 40%). Isolated by preparative TLC (hexane: ethyl acetate =80:1, R_f = 0.5); ^{1}H NMR (500 MHz, $CDCl_3$) δ 7.30 (d, J = 8.6 Hz, 2H), 6.86 (d, J = 8.6 Hz, 2H), 6.31 (d, J = 16.0 Hz, 1H), 6.06 (dd, J = 16.0, 7.0 Hz, 1H), 3.82 (s, 3H), 2.15-2.09 (m, 1H), 1.85-1.68 (m, 5H), 1.36-1.18 (m, 5H); ^{13}C NMR (125 MHz, $CDCl_3$) δ 158.6, 134.8, 130.9, 127.0, 126.5, 113.9, 55.3, 41.1, 33.1, 26.2, 26.1; EI-MS (m/z): 216.2. The spectroscopic data for this product match the literature data.[18]

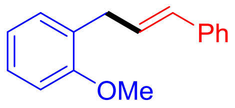

1-Cinnamyl-2-methoxybenzene (4ca). (41.2 mg, 92%). Isolated by preparative TLC (hexane: ethyl acetate = 50:1, R_f = 0.5); ^{1}H NMR (500 MHz, $CDCl_3$) δ 7.40-7.38 (m, 2H), 7.33-7.30 (m, 2H), 7.27-7.21 (m, 3H), 6.95 (td, J = 7.4, 1.0 Hz, 1H), 6.92 (d, J = 8.2 Hz, 1H), 6.48 (d, J = 16.0 Hz, 1H), 6.45-6.40 (m, 1H), 3.89 (s, 3H), 3.58 (d, J = 6.0 Hz, 2H); ^{13}C NMR (125 MHz, $CDCl_3$) δ 157.3, 137.8, 130.7, 130.0, 128.9, 128.7, 128.5, 127.4, 126.9, 126.1, 120.6, 110.4, 55.4, 33.4; EI-MS (m/z): 224.1. The spectroscopic data for this product match the literature data.[19]

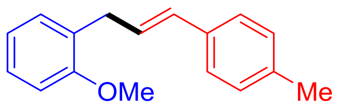

(*E*)-1-Methoxy-2-(3-(p-tolyl)allyl)benzene (4cb). (40.5 mg, 85%). Isolated by preparative TLC (hexane: ethyl acetate = 50:1, R_f = 0.5); ^{1}H NMR (500 MHz, $CDCl_3$) δ 7.31 (d, J = 8.0 Hz, 2H), 7.26-7.17 (m, 2H), 7.15 (d, J = 8.0 Hz, 2H), 6.97 (t, J = 7.2 Hz, 1H), 6.93 (d, J = 7.2 Hz, 1H), 6.47 (d, J = 15.8 Hz, 1H), 6.43-6.36 (m, 1H), 3.90 (s, 3H), 3.59 (d, J = 6.5 Hz, 2H), 2.38 (s, 3H); ^{13}C NMR (125 MHz, $CDCl_3$) δ 157.3, 136.6, 135.0, 130.6, 129.9, 129.2, 127.9, 127.4, 126.0, 120.6, 110.4, 55.4, 33.4, 21.2; HRMS (ESI) calcd for $C_{17}H_{19}O$ [M + H$^+$], 239.1430; found: 239.1426.

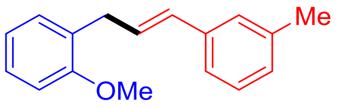

(*E*)-1-Methoxy-2-(3-(m-tolyl)allyl)benzene (4cc). (37.2 mg, 78%). Isolated by preparative TLC (hexane: ethyl acetate = 50:1, R_f = 0.5); ^{1}H NMR (500 MHz, $CDCl_3$) δ 7.27-7.20 (m, 5H), 7.06-7.04 (m, 1H), 6.95 (td, J = 7.4, 1.0 Hz, 1H), 6.92 (d, J = 8.2 Hz, 1H), 6.45 (d, J = 15.8 Hz, 1H), 6.44-6.37 (m, 1H), 3.89 (s, 3H), 3.58 (d, J = 5.6 Hz, 2H), 2.37 (s, 3H); ^{13}C NMR (125 MHz, $CDCl_3$) δ 157.3, 138.0, 137.7, 130.8, 129.9, 128.8, 128.7, 128.4, 127.7, 127.4, 126.8, 123.3, 120.6, 110.4, 55.4, 33.4, 21.4; HRMS (ESI) calcd for $C_{17}H_{19}O$ [M + H$^+$], 239.1430; found: 239.1431.

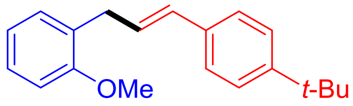

(*E*)-1-(3-(4-Fluorophenyl)allyl)-2-methoxybenzene (4cd). (46.5 mg, 83%). Isolated by preparative TLC (hexane: ethyl acetate = 50:1, R_f = 0.5); ^{1}H NMR (500 MHz, $CDCl_3$) δ 7.41-7.34 (m, 4H), 7.27-7.24 (m, 2H), 6.96 (td, J = 7.4, 1.0 Hz, 1H), 6.92 (d, J = 8.2 Hz, 1H), 6.48 (d, J = 15.8 Hz, 1H), 6.43-6.37 (m, 1H), 3.89 (s, 3H), 3.59 (d, J = 6.7 Hz, 2H), 1.36 (s, 9H); ^{13}C NMR (125 MHz, $CDCl_3$) δ 157.3, 149.9, 135.1, 130.5, 129.8, 128.9, 128.1, 127.4, 125.8, 125.4, 120.5, 110.4, 55.4, 34.5, 33.4, 31.4; HRMS (ESI) calcd for $C_{20}H_{25}O$ [M + H$^+$], 281.1900; found: 281.1902.

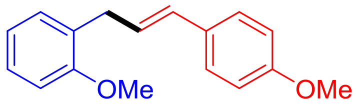

(*E*)-1-Methoxy-2-(3-(4-methoxyphenyl)allyl)benzene (4ce).[17] (41.7 mg, 82%). Isolated by preparative TLC (hexane: ethyl acetate = 40:1, R_f = 0.4); ^{1}H NMR (500 MHz, $CDCl_3$) δ 7.32 (d, J = 8.8 Hz, 2H), 7.26-7.20 (m, 2H), 6.94 (td, J = 7.4, 1.0 Hz, 1H), 6.91 (d, J = 8.0 Hz, 1H), 6.86 (d, J = 8.8 Hz, 2H), 6.41 (d, J = 15.8 Hz, 1H),

6.30-6.25 (m, 1H), 3.88 (s, 3H), 3.83 (s, 3H), 3.55 (d, J = 6.8 Hz, 2H); ^{13}C NMR (125 MHz, CDCl$_3$) δ 158.7, 157.3, 130.7, 130.1, 129.8, 129.6, 129.0, 127.3, 127.2, 126.7, 120.5, 113.9, 110.4, 55.4, 55.3, 33.3; EI-MS (m/z): 254.1.

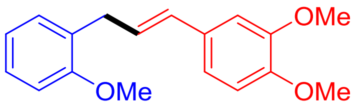

(*E*)-1,2-Dimethoxy-4-(3-(2-methoxyphenyl)prop-1-en-1-yl)benzene (4cf). (51.7 mg, 91%). Isolated by preparative TLC (hexane: ethyl acetate = 8:1, R_f = 0.4); ^{1}H NMR (500 MHz, CDCl$_3$) δ 7.27-7.23 (m, 2H), 7.00-6.89 (m, 4H), 6.84-6.81 (m, 1H), 6.41 (d, J = 15.8 Hz, 1H), 6.30-6.25 (m, 1H), 3.91 (s, 3H), 3.90 (s, 3H), 3.88 (s, 3H), 3.57 (d, J = 6.0 Hz, 2H); ^{13}C NMR (125 MHz, CDCl$_3$) δ 157.3, 149.0, 148.3, 131.0, 130.3, 129.9, 128.9, 127.4, 127.0, 120.5, 119.0, 111.1, 110.4, 108.7, 55.9, 55.8, 55.4, 33.3; HRMS (ESI) calcd for $C_{18}H_{21}O_3$ [M + H$^+$], 285.1485; found: 285.1481.

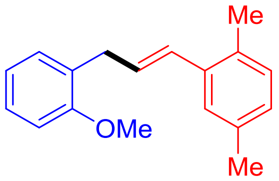

(*E*)-2-(3-(2-Methoxyphenyl)prop-1-en-1-yl)-1,4-dimethylbenzene (4cg). (43.9 mg, 87%). Isolated by preparative TLC (hexane: ethyl acetate = 50:1, R_f = 0.5); ^{1}H NMR (500 MHz, CDCl$_3$) δ 7.30-7.05 (m, 4H), 7.03-6.89 (m, 3H), 6.68 (d, J = 15.8 Hz, 1H), 6.39-6.25 (m, 1H), 3.90 (s, 3H), 3.61 (d, J = 6.8 Hz, 2H), 2.34 (s, 3H), 2.33 (s, 3H); ^{13}C NMR (125 MHz, CDCl$_3$) δ 157.3, 136.7, 135.3, 132.0, 130.1, 129.8, 129.7, 128.9, 128.8, 127.6, 127.4, 126.2, 120.6, 110.3, 55.4, 33.7, 21.0, 19.4; HRMS (ESI) calcd for $C_{18}H_{21}O$ [M + H$^+$], 253.1587; found: 253.1590.

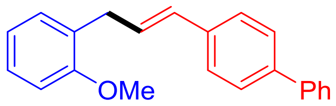

(*E*)-4-(3-(2-Methoxyphenyl)prop-1-en-1-yl)-1,1'-biphenyl (4ch). (49.2 mg, 82%). Isolated by preparative TLC (hexane: ethyl acetate = 50:1, R_f = 0.5); ^{1}H NMR (500 MHz, CDCl$_3$) δ 7.64-7.62 (m, 2H), 7.59-7.55 (m, 2H), 7.48-7.45 (m, 4H), 7.38-7.34 (m, 1H), 7.28-7.22 (m, 2H), 6.96 (td, J = 7.4, 1.0 Hz, 1H), 6.92 (d, J = 8.0 Hz, 1H), 6.52 (d, J = 15.8 Hz, 1H), 6.49-6.39 (m, 1H), 3.90 (s, 3H), 3.60 (d, J = 5.6 Hz, 2H); ^{13}C NMR (125 MHz, CDCl$_3$) δ 157.3, 140.9, 139.7, 136.9, 130.2, 129.9, 129.2, 128.8, 127.5, 127.2, 126.9, 126.5, 120.6, 110.4, 55.4, 33.5; HRMS (ESI) calcd for $C_{22}H_{21}O$ [M + H$^+$], 301.1587; found: 301.1585.

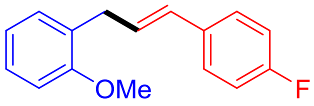

(*E*)-1-(3-(4-Fluorophenyl)allyl)-2-methoxybenzene (4ci). (39.7 mg, 81%). Isolated by preparative TLC (hexane: ethyl acetate = 50:1, R_f = 0.5); ^{1}H NMR (500 MHz, $CDCl_3$) δ 7.36-7.33 (m, 2H), 7.28-7.22 (m, 2H), 7.05-6.98 (m, 2H), 6.96 (td, J = 7.4, 1.0 Hz, 1H), 6.92 (d, J = 8.2 Hz, 1H), 6.43 (d, J = 15.8 Hz, 1H), 6.39-6.31 (m, 1H), 3.89 (s, 3H), 3.57 (d, J = 6.5 Hz, 2H); ^{13}C NMR (125 MHz, $CDCl_3$) δ 162.0 (d, $J_{C\text{-}F}$ = 245.8 Hz), 157.3, 134.0 (d, $J_{C\text{-}F}$ = 3.2 Hz), 129.9, 129.5, 128.7, 128.6, 127.6 (d, $J_{C\text{-}F}$ = 7.6 Hz), 127.5, 120.6, 115.3 (d, $J_{C\text{-}F}$ = 21.8 Hz), 110.4, 55.4, 33.4; ^{19}F NMR (470 MHz, $CDCl_3$) δ -115.7; HRMS (ESI) calcd for $C_{16}H_{16}FO$ [M + H$^+$], 243.1180; found: 243.1182.

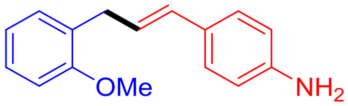

(*E*)-4-(3-(2-methoxyphenyl)prop-1-en-1-yl)aniline (4cj). (36.8 mg, 77%). Isolated by preparative TLC (hexane: ethyl acetate = 2:1, R_f = 0.5); ^{1}H NMR (500 MHz, $CDCl_3$) δ 7.26-7.19 (m, 4H), 6.94 (td, J = 7.4, 1.0 Hz, 1H), 6.91 (dd, J = 8.4, 0.8 Hz, 1H), 6.64 (d, J = 8.6 Hz, 2H), 6.38 (d, J = 15.8 Hz, 1H), 6.25-6.19 (m, 1H), 3.88 (s, 3H), 3.65 (br, 2H), 3.54 (d, J = 6.8 Hz, 2H); ^{13}C NMR (125 MHz, $CDCl_3$) δ 157.3, 145.4, 130.5, 129.8, 129.2, 128.7, 127.3, 127.2, 125.2, 120.5, 115.2, 110.4, 55.4, 33.3; HRMS (ESI) calcd for $C_{16}H_{18}NO$ [M + H$^+$], 240.1383; found: 240.1385.

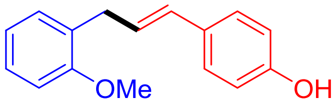

(*E*)-4-(3-(2-Methoxyphenyl)prop-1-en-1-yl)phenol (4ck). (25.5 mg, 53%). Isolated by preparative TLC (hexane: ethyl acetate = 5:1, R_f = 0.5); ^{1}H NMR (500 MHz, $CDCl_3$) δ 7.27-7.25 (m, 2H), 7.23-7.21 (m, 2H), 6.95-6.88 (m, 2H), 6.80-6.76 (m, 2H), 6.39 (d, J = 15.8 Hz, 1H), 6.28-6.22 (m, 1H), 4.85 (br, 1H), 3.87 (s, 3H), 3.54 (d, J = 6.7 Hz, 2H); ^{13}C NMR (125 MHz, $CDCl_3$) δ 157.3, 154.6, 130.8, 130.0, 129.8, 128.9, 127.4, 127.3, 126.8, 120.5, 115.3, 110.4, 55.4, 33.3; HRMS (ESI) calcd for $C_{16}H_{17}O_2$ [M + H$^+$], 241.1223; found: 241.1225.

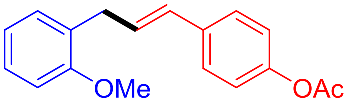

(*E*)-4-(3-(2-Methoxyphenyl)prop-1-en-1-yl)phenyl acetate (4cl). (21.4 mg, 38%). Isolated by preparative TLC (hexane: ethyl acetate = 10:1, R_f = 0.5); ^{1}H NMR (500 MHz, $CDCl_3$) δ 7.37 (d, J = 8.6 Hz, 2H), 7.24 (td, J = 7.8, 1.7 Hz, 1H), 7.21 (td, J = 7.4, 1.7 Hz, 1H), 7.02 (d, J = 8.6 Hz, 2H), 6.94 (td, J = 7.4, 1.0 Hz, 1H), 6.90 (d, J = 8.2 Hz, 1H), 6.43 (d, J = 15.8 Hz, 1H), 6.38-6.33 (m, 1H), 3.87 (s, 3H), 3.55 (d, J = 6.5 Hz, 2H), 2.31 (s, 3H); ^{13}C NMR (125 MHz, $CDCl_3$) δ 169.5, 157.3, 149.5, 135.6, 129.8, 129.7, 129.3, 128.6, 127.5, 127.0, 121.5, 120.6, 110.4, 55.4, 33.4, 21.1; HRMS (ESI) calcd for $C_{18}H_{19}O_3$ [M + H$^+$], 283.1329; found: 283.1333.

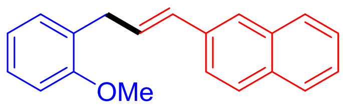

(*E*)-2-(3-(2-methoxyphenyl)prop-1-en-1-yl)naphthalene (4cm). (41.2 mg, 75%). Isolated by preparative TLC (hexane: ethyl acetate = 50:1, R_f = 0.5); ^{1}H NMR (500 MHz, $CDCl_3$) δ 7.86-7.82 (m, 2H), 7.80 (d, J = 7.6 Hz, 1H), 7.74 (s, 1H), 7.65 (dd, J = 8.6, 1.7 Hz, 1H), 7.51-7.44 (m, 2H), 7.30-7.27 (m, 2H), 6.99 (td, J = 7.6, 1.0 Hz, 1H), 6.95 (dd, J = 8.6, 1.0 Hz, 1H), 6.66 (d, J = 15.8 Hz, 1H), 6.61-6.55 (m, 1H), 3.92 (s, 3H), 3.67 (d, J = 6.6 Hz, 2H); ^{13}C NMR (125 MHz, $CDCl_3$) δ 157.4, 135.3, 133.7, 132.7, 130.8, 130.0, 129.5, 128.7, 128.0, 127.9, 127.7, 127.5, 126.1, 125.6, 125.5, 123.7, 120.6, 110.5, 55.5, 33.6; HRMS (ESI) calcd for $C_{20}H_{19}O$ [M + H$^+$], 275.1430; found: 275.1428.

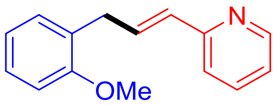

(*E*)-2-(3-(2-methoxyphenyl)prop-1-en-1-yl)pyridine (4cn). (37.2 mg, 83%). Isolated by preparative TLC (hexane: ethyl acetate = 5:1, R_f = 0.5); ^{1}H NMR (400 MHz, $CDCl_3$) δ 8.54 (d, J = 4.2 Hz, 1H), 7.60 (td, J = 7.8, 1.8 Hz, 1H), 7.28 (d, J = 7.8 Hz, 1H), 7.25-7.21 (m, 2H), 7.11-7.08 (m, 1H), 6.95-6.87 (m, 3H), 6.54 (dt, J = 15.8, 1.4 Hz, 1H), 3.87 (s, 3H), 3.62 (dd, J = 6.8, 0.8 Hz, 2H); ^{13}C NMR (125 MHz, $CDCl_3$) δ 157.3, 156.1, 149.3, 136.4, 134.0, 130.6, 130.1, 128.0, 127.6, 121.6, 121.0, 120.6, 110.4, 55.4, 33.4; HRMS (APCI) calcd for $C_{15}H_{16}ON$ [M + H+], 226.1226; found: 226.1229.

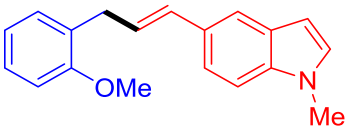

(*E*)-5-(3-(2-Methoxyphenyl)prop-1-en-1-yl)-1-methyl-1H-indole (4co). (39.4 mg, 71%). Isolated by preparative TLC (hexane: ethyl acetate = 20:1, R_f = 0.4); ^{1}H NMR (500 MHz, $CDCl_3$) δ 7.63 (s, 1H), 7.37 (dd, J = 8.6, 1.5 Hz, 1H), 7.30-7.24 (m, 3H), 7.04 (d, J = 3.0 Hz, 1H), 6.97 (td, J = 7.4, 0.9 Hz, 1H), 6.93 (d, J = 8.2 Hz, 1H), 6.62 (d, J = 15.8 Hz, 1H), 6.48 (dd, J = 3.0, 0.6 Hz, 1H), 6.38 (dt, J =15.8, 6.8 Hz, 1H), 3.91 (s, 3H), 3.80 (s, 3H), 3.62 (d, J = 6.8 Hz, 2H); ^{13}C NMR (125 MHz, $CDCl_3$) δ 157.4, 136.2, 131.9, 129.9, 129.5, 129.4, 129.1, 128.7, 127.3, 125.8, 120.6, 120.0, 118.9, 110.4, 109.2, 101.1, 55.5, 33.4, 32.9; HRMS (APCI) calcd for $C_{19}H_{20}ON$ [M + H$^+$], 278.1539; found: 278.1548.

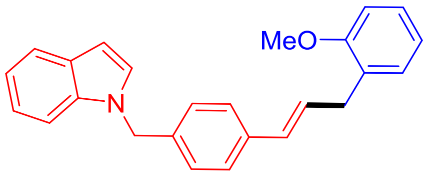

(*E*)-1-(4-(3-(2-methoxyphenyl)prop-1-en-1-yl)benzyl)-1H-indole (4cp). (57.1 mg, 81%). Isolated by preparative TLC (hexane: ethyl acetate = 10:1, R_f = 0.5); ^{1}H NMR (400 MHz, $CDCl_3$) δ 7.76-7.74 (m, 1H), 7.40-7.38 (m, 1H), 7.35 (d, J = 8.2 Hz, 2H), 7.32-7.26 (m, 3H), 7.23-7.18 (m, 2H), 7.10 (d, J = 8.2 Hz, 2H), 7.00 (td, J = 7.4, 1.0 Hz, 1H), 6.96 (d, J = 8.0 Hz, 1H), 6.65-6.63 (m, 1H), 6.49 (d, J = 15.8 Hz, 1H), 6.46-6.38 (m, 1H), 5.33 (s, 2H), 3.91 (s, 3H), 3.62 (d, J = 5.4 Hz, 2H); ^{13}C NMR (100 MHz, $CDCl_3$) δ 157.4, 137.3, 136.1, 130.2, 129.9, 129.3, 129.1, 128.8, 128.6, 128.3, 127.5, 127.1, 126.5, 121.7, 121.0, 120.6, 119.6, 110.5, 109.8, 101.7, 55.4, 49.9, 33.5; HRMS (APCI) calcd for $C_{25}H_{24}ON$ [M + H$^+$], 354.1852; found: 354.1856.

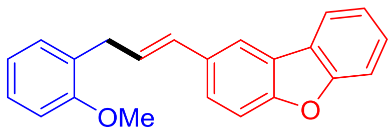

(*E*)-2-(3-(2-Methoxyphenyl)prop-1-en-1-yl)dibenzo[b,d]furan (4cq). (39.4 mg, 92%). Isolated by preparative TLC (hexane: ethyl acetate =60:1, R_f = 0.5); ^{1}H NMR (500 MHz, $CDCl_3$) δ 7.98-7.96 (m, 2H), 7.61-7.59 (m, 1H), 7.56-7.47 (m, 3H), 7.41-7.36 (m, 1H), 7.30-7.26 (m, 2H), 7.01-6.92 (m, 2H), 6.64 (d, J = 15.8 Hz, 1H), 6.51-6.46 (m, 1H), 3.92 (s, 3H), 3.65 (d, J = 6.8 Hz, 2H); ^{13}C NMR (125 MHz, $CDCl_3$) δ 157.4, 156.6, 155.5, 133.0, 130.5, 130.0, 128.8, 128.3, 127.5, 127.1, 125.6, 124.5, 124.3, 122.7, 120.7, 120.6, 118.0, 111.7, 111.5, 110.5, 55.5, 33.5; HRMS (APCI) calcd for $C_{22}H_{19}O_2$ [M + H$^+$], 315.1380; found: 315.1382.

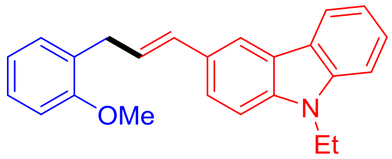

(*E*)-9-Ethyl-3-(3-(2-methoxyphenyl)prop-1-en-1-yl)-9H-carbazole (4cr). (46.4 mg, 68%). Isolated by preparative TLC (hexane: ethyl acetate = 10:1, R_f = 0.5); ^{1}H NMR (500 MHz, $CDCl_3$) δ 8.13-8.11 (m, 2H), 7.57 (dd, J = 8.4, 1.6 Hz, 1H), 7.49 (td, J = 7.6, 1.2 Hz, 1H), 7.42 (d, J = 8.2 Hz, 1H), 7.35 (d, J = 8.4 Hz, 1H), 7.32 (dd, J = 8.6, 1.6 Hz, 1H), 7.27-7.24 (m, 2H), 6.98 (td, J = 7.4, 0.9 Hz, 1H), 6.95 (d, J = 8.2 Hz, 1H), 6.68 (d, J = 15.8 Hz, 1H), 6.45 (dt, J = 15.8, 6.8 Hz, 1H), 4.38 (q, J = 7.2 Hz, 2H), 3.92 (s, 3H), 3.65 (d, J = 6.8 Hz, 2H), 1.46 (t, J = 7.2 Hz, 3H); ^{13}C NMR (125 MHz, $CDCl_3$) δ 157.4, 140.3, 139.3, 131.4, 129.9, 129.2, 129.1, 127.3, 126.1, 125.6, 124.1, 123.1, 123.0, 120.6, 120.5, 118.8, 118.1, 110.4, 108.5, 108.4, 55.5, 37.6, 33.5, 13.8; HRMS (APCI) calcd for $C_{24}H_{24}ON$ [M + H$^+$], 342.1852; found: 342.1862.

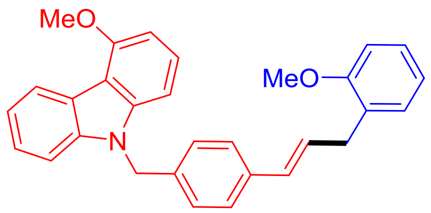

(*E*)-4-methoxy-9-(4-(3-(2-methoxyphenyl)prop-1-en-1-yl)benzyl)-9H-carbazole (4cs). (76.2 mg, 88%). Isolated by preparative TLC (hexane: ethyl acetate = 10:1, R_f = 0.6); ^{1}H NMR (400 MHz, $CDCl_3$) δ 8.46 (d, J = 7.6 Hz, 1H), 7.47-7.38 (m, 3H), 7.36-7.32 (m, 1H), 7.30-7.26 (m, 3H), 7.24-7.11 (m, 2H), 7.09 (d, J = 8.2 Hz, 2H), 6.96 (td, J = 7.4, 0.9 Hz, 1H), 6.91 (d, J = 8.0 Hz, 1H), 6.76 (d, J = 8.0 Hz, 1H), 6.43 (d, J = 15.8 Hz, 1H), 6.40-6.33 (m, 1H), 5.49 (s, 2H), 4.15 (s, 3H), 3.88 (s, 3H), 3.57 (d, J = 5.6 Hz, 2H); ^{13}C NMR (125 MHz, $CDCl_3$) δ 157.3, 156.4, 142.2, 139.9, 137.1, 135.9, 130.2, 129.9, 129.1, 128.6, 127.5, 126.7, 126.6, 126.5, 124.9, 123.2, 122.5, 120.6, 119.5, 112.2, 110.4, 108.4, 102.0, 100.4, 55.5, 55.4, 46.6, 33.4; HRMS (APCI) calcd for $C_{30}H_{28}O_2N$ [M + H^+], 434.2115; found: 434.2110.

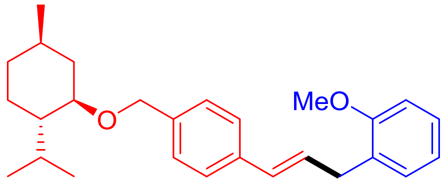

1-((*E*)-3-(4-((((1R,2S,5R)-2-Isopropyl-5-methylcyclohexyl)oxy)methyl)phenyl)allyl)-2-methoxybenzene (6). (0.1 mmol scale, 31 mg, 79%). Isolated by preparative TLC (hexane: ethyl acetate = 50:1, R_f = 0.5); ^{1}H NMR (500 MHz, $CDCl_3$) δ 7.35 (d, J = 8.2 Hz, 2H), 7.29 (d, J = 8.2 Hz, 2H), 7.27-7.22 (m, 2H), 6.94 (t, J = 7.1 Hz, 1H), 6.91 (d, J = 8.2 Hz, 1H), 6.45 (d, J = 15.8 Hz, 1H), 6.40 (td, J = 15.8, 6.0 Hz, 1H), 4.65 (d, J = 11.6 Hz, 1H), 4.40 (d, J = 11.6 Hz, 1H), 3.88 (s, 3H), 3.57 (d, J = 6.2 Hz, 2H), 3.23-3.16 (m, 1H), 2.35-2.30 (m, 1H), 2.25-2.20 (m, 1H), 1.70-1.63 (m, 2H), 1.42-1.29 (m, 2H), 1.03-0.85 (m, 9H), 0.74 (d, J = 7.0 Hz, 3H); ^{13}C NMR (125 MHz, $CDCl_3$) δ 157.3, 137.8, 137.0, 130.5, 129.9, 128.7, 128.1, 128.0, 127.4, 126.0, 120.6, 110.4, 78.6, 70.2, 55.4, 48.4, 40.3, 34.6, 33.4, 31.6, 25.5, 23.3, 22.4, 21.1, 16.1; HRMS (ESI) calcd for $C_{46}H_{67}O_3$ [M + H^+], 415.2608; found: 415.2603.

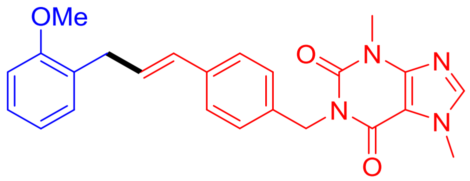

(*E*)-1-(4-(3-(2-Methoxyphenyl)prop-1-en-1-yl)benzyl)-3,7-dimethyl-3,7-dihydro-1H-purine-2,6-dione (8). (0.1 mmol scale, 38.3 mg, 92%). Isolated by preparative TLC (dichloromethane: methanol = 1:10, R_f = 0.3); ^{1}H NMR (500 MHz, $CDCl_3$) δ 7.50 (s, 1H), 7.44 (d, J = 8.2 Hz, 2H), 7.31 (d, J = 8.2 Hz, 2H), 7.24-7.17 (m, 2H),

6.93-6.86 (m, 2H), 6.41 (d, J = 15.8 Hz, 1H), 6.35 (td, J = 15.8, 6.4 Hz, 1H), 5.17 (s, 2H), 3.99 (s, 3H), 3.85 (s, 3H), 3.59 (s, 3H), 3.53 (d, J = 6.4 Hz, 2H); ^{13}C NMR (125 MHz, CDCl$_3$) δ 157.3, 155.3, 151.6, 148.9, 141.5, 137.2, 136.0, 130.4, 129.8, 129.1, 129.0, 128.7, 127.4, 126.1, 120.5, 110.3, 107.7, 55.4, 44.2, 33.6, 33.4, 29.8; HRMS (ESI) calcd for $C_{24}H_{25}O_3N_4$ [M + H$^+$], 417.1921; found: 417.1920.

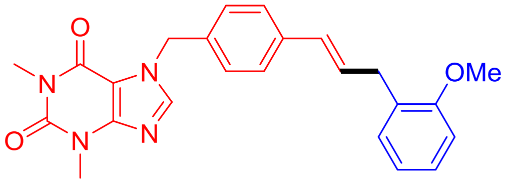

(*E*)-7-(4-(3-(2-methoxyphenyl)prop-1-en-1-yl)benzyl)-1,3-dimethyl-3,7-dihydro-1H-purine-2,6-dione (10). (37.2 mg, 88%). Isolated by preparative TLC (dichloromethane: methanol = 1:10, R$_f$ = 0.4); ^{1}H NMR (400 MHz, CDCl$_3$) δ 7.56 (s, 1H), 7.35 (d, J = 8.0 Hz, 2H), 7.26 (d, J = 8.0 Hz, 2H), 7.23-7.17 (m, 2H), 6.94-6.88 (m, 2H), 6.40 (s, 2H), 5.50-5.47 (m, 2H), 3.86 (m, 3H), 3.60 (s, 3H), 3.55 (s, 2H), 3.42 (s, 3H); ^{13}C NMR (125 MHz, CDCl$_3$) δ 157.3, 155.3, 151.7, 148.9, 140.8, 138.3, 133.6, 130.1, 129.8, 129.7, 128.4, 128.2, 127.5, 126.9, 126.7, 120.5, 110.4, 55.4, 50.1, 33.4, 29.8, 28.0; HRMS (APCI) calcd for $C_{24}H_{25}O_3N_4$ [M + H$^+$], 417.1921; found: 417.1916.

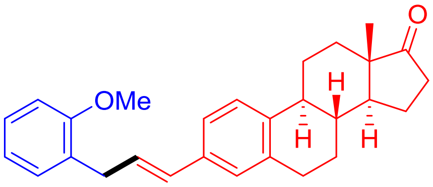

(8R,9S,13S,14S)-3-((*E*)-3-(2-Methoxyphenyl)prop-1-en-1-yl)-13-methyl-6,7,8,9,11,12,13,14,15,16-decahydro-17H-cyclopenta[a]phenanthren-17-one (12). (0.1 mmol scale, 36.4 mg, 91%). Isolated by preparative TLC (hexane: ethyl acetate =5:1, R$_f$ = 0.5); ^{1}H NMR (500 MHz, CDCl$_3$) δ 7.26-7.19 (m, 4H), 7.13 (s, 1H), 6.96-6.90 (m, 2H), 6.44-6.34 (m, 2H), 3.88 (s, 3H), 3.57 (d, J = 6.2 Hz, 2H), 2.94-2.91 (m, 2H), 2.57-2.51 (m, 1H), 2.46-2.43 (m, 1H), 2.33-2.30 (m, 1H), 2.21-2.14 (m, 1H), 2.11-1.98 (m, 3H), 1.70-1.57 (m, 3H), 1.55-1.45 (m, 3H), 0.94 (s, 3H); ^{13}C NMR (125 MHz, CDCl$_3$) δ 220.9, 157.3, 138.6, 136.5, 135.4, 130.4, 129.8, 128.8, 128.3, 127.4, 126.7, 125.5, 123.6, 120.5, 110.4, 55.4, 50.5, 48.0, 44.4, 38.2, 35.9, 33.4, 31.6, 29.4, 26.6, 25.8, 21.6, 13.9; HRMS (APCI) calcd for $C_{28}H_{33}O_2$ [M + H$^+$], 401.2475; found: 401.2484.

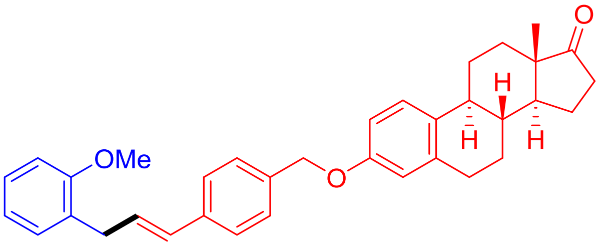

(8R,9S,13S,14S)-3-((4-((*E*)-3-(2-Methoxyphenyl)prop-1-en-1-yl)benzyl)oxy)-13-methyl-6,7,8,9,11,12,13,14,15,16-decahydro-17H-cyclopenta[a]phenanthren-17-one (14). (0.1 mmol scale, 38.5 mg, 76%). Isolated by preparative TLC (hexane: ethyl acetate =5:1, R_f = 0.5); ^{1}H NMR (500 MHz, $CDCl_3$) δ 7.44-7.35 (m, 4H), 7.26-7.21 (m, 3H), 6.96-6.89 (m, 2H), 6.84-6.74 (m, 2H), 6.48-6.38 (m, 2H), 5.04 (s, 2H), 3.88 (s, 3H), 3.57 (d, *J* = 5.7 Hz, 2H), 2.94-2.90 (m, 2H), 2.56-2.50 (m, 1H), 2.44-2.40 (m, 1H), 2.30-2.26 (m, 1H), 2.21-2.13 (m, 1H), 2.11-1.97 (m, 3H), 1.69-1.61 (m, 3H), 1.55-1.46 (m, 3H), 0.94 (s, 3H); ^{13}C NMR (125 MHz, $CDCl_3$) δ 221.0, 157.3, 156.9, 137.8, 137.5, 135.8, 132.3, 130.3, 129.9, 129.2, 128.6, 127.6, 127.5, 126.3, 126.2, 120.6, 115.0, 112.5, 110.4, 69.8, 55.4, 50.4, 48.0, 44.0, 38.4, 35.9, 33.4, 31.6, 29.7, 26.6, 25.9, 21.6, 13.9; HRMS (APCI) calcd for $C_{35}H_{39}O_3$ [M + H$^+$], 507.2907; found: 507.2894.

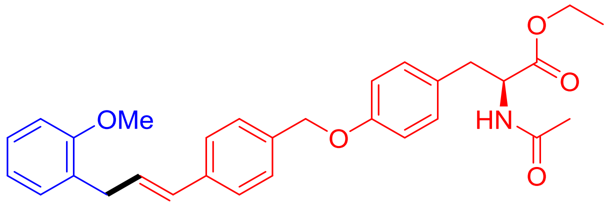

Ethyl (*S*,*E*)-2-acetamido-3-(4-((4-(3-(2-methoxyphenyl)prop-1-en-1-yl)benzyl)oxy)phenyl)propanoate (16). (0.1 mmol scale, 30.2 mg, 62%). Isolated by preparative TLC (hexane: ethyl acetate = 1:1, R_f = 0.4); ^{1}H NMR (500 MHz, $CDCl_3$) δ 7.38 (d, *J* = 8.2 Hz, 2H), 7.35 (d, *J* = 8.2 Hz, 2H), 7.26-7.20 (m, 2H), 7.05-7.02 (m, 2H), 6.95-6.89 (m, 4H), 6.48-6.38 (m, 2H), 5.94 (d, *J* = 7.4 Hz, 1H), 5.02 (s, 2H), 4.87-4.82 (m, 1H), 4.22-4.17 (m, 2H), 3.87 (s, 3H), 3.57 (d, *J* = 5.6 Hz, 2H), 3.12-3.05 (m, 2H), 2.01 (s, 3H), 1.27 (t, *J* = 7.2 Hz, 3H); ^{13}C NMR (125 MHz, $CDCl_3$) δ 171.7, 169.6, 157.9, 157.3, 137.6, 135.5, 130.3, 130.2, 129.9, 129.3, 129.0, 128.6, 128.1, 127.7, 127.5, 126.3, 120.6, 115.0, 110.4, 69.9, 61.5, 55.4, 53.3, 37.1, 33.4, 23.2, 14.2; HRMS (APCI) calcd for $C_{30}H_{34}O_5N$ [M + H$^+$], 488.2432; found: 488.2437.

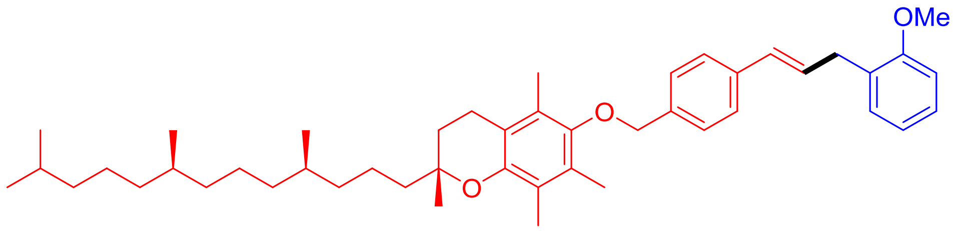

(R)-6-((4-((*E*)-3-(2-Methoxyphenyl)prop-1-en-1-yl)benzyl)oxy)-2,5,7,8-tetramethyl-2-((4R,8R)-4,8,12-trimethyltridecyl)chromane (18). (0.1 mmol scale, 55.3 mg, 83%). Isolated by preparative TLC (hexane: ethyl acetate = 50:1, R_f = 0.5); ^{1}H NMR (500 MHz, $CDCl_3$) δ 7.47 (d, *J* = 8.2 Hz, 2H), 7.44 (d, *J* = 8.2 Hz, 2H), 7.29-7.26 (m, 2H), 6.98 (td, *J* = 7.6, 0.8 Hz, 1H), 6.94 (d, *J* = 8.2 Hz, 1H), 6.52 (d, *J* = 15.8 Hz, 1H), 6.46 (td, *J* = 15.8, 6.0 Hz, 1H), 4.73 (s, 2H), 3.91 (s, 3H), 3.62 (d, *J* = 6.0 Hz, 2H), 2.65 (t, *J* = 6.6 Hz, 2H), 2.27 (s, 3H), 2.22 (s, 3H), 2.17 (s, 3H),

1.92-1.80 (m, 2H), 1.66-1.55 (m, 3H), 1.52-1.44 (m, 4H), 1.38-1.28 (m, 11H), 1.23-1.11 (m, 6H), 0.95-1.91 (m, 12H); ^{13}C NMR (125 MHz, $CDCl_3$) δ 157.4, 148.2, 147.9, 137.4, 136.7, 130.5, 129.9, 129.0, 128.7, 127.9, 127.5, 126.2, 126.0, 122.9, 120.6, 117.6, 110.4, 74.8, 74.6, 55.4, 40.1, 39.4, 37.5, 37.4, 33.5, 32.8, 32.7, 31.4, 28.0, 24.9, 24.5, 24.0, 22.8, 22.7, 21.1, 20.7, 19.8, 19.7, 13.0, 12.1, 11.9; HRMS (APCI) calcd for $C_{46}H_{67}O_3$ [M + H^+], 667.5085; found: 667.5104.

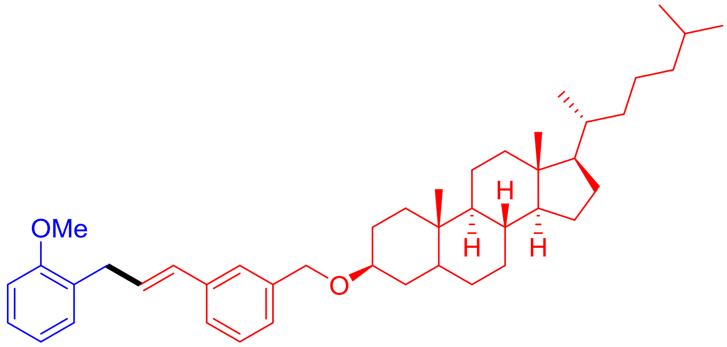

(3S,8R,9S,10S,13R,14S,17R)-3-((3-((*E*)-3-(2-Methoxyphenyl)prop-1-en-1-yl)benzyl)oxy)-10,13-dimethyl-17-((R)-6-methylheptan-2-yl)hexadecahydro-1H-cyclopenta[a]phenanthrene (20). (0.1 mmol scale, 53 mg, 85%). Isolated by preparative TLC (hexane: ethyl acetate = 50:1, R_f = 0.4); ^{1}H NMR (500 MHz, $CDCl_3$) δ 7.34 (d, J = 8.2 Hz, 2H), 7.28 (d, J = 8.2 Hz, 2H), 7.26-7.21 (m, 2H), 6.94 (td, J = 7.4, 1.0 Hz, 1H), 6.90 (d, J = 8.0 Hz, 1H), 6.45 (d, J = 15.8 Hz, 1H), 6.39 (td, J = 15.8, 6.2 Hz, 1H), 4.57-4.51 (m, 2H), 3.88 (s, 3H), 3.56 (d, J = 6.2 Hz, 2H), 3.36-3.30 (m, 1H), 2.00-1.97 (m, 1H), 1.92-1.90 (m, 1H), 1.85-1.81 (m, 1H), 1.76-1.66 (m, 3H), 1.59-1.45 (m, 4H), 1.39-1.25 (m, 10H), 1.20-0.96 (m, 10H), 0.93 (d, J = 6.4 Hz, 3H), 0.90 (d, J = 2.2 Hz, 3H), 0.89 (d, J = 2.2 Hz, 3H), 0.83 (s, 3H), 0.68 (s, 3H), 0.65-0.60 (m, 1H); ^{13}C NMR (125 MHz, $CDCl_3$) δ 157.3, 137.9, 137.0, 130.5, 129.9, 128.7, 127.7, 127.4, 126.0, 120.5, 110.4, 77.8, 69.5, 56.5, 56.3, 55.4, 54.5, 44.9, 42.6, 40.1, 39.5, 37.0, 36.2, 35.8, 35.5, 34.9, 33.4, 32.2, 28.9, 28.3, 28.0, 24.2, 23.9, 22.8, 22.6, 21.3, 18.7, 12.3, 12.1; HRMS (APCI) calcd for $C_{44}H_{65}O_2$ [M + H^+], 625.4979; found: 625.4982.

Mechanistic study

The phenyldiazomethane **21** was prepared following a literature procedure.[20] To a solution of benzaldehyde **1a** (0.6 mmol) in THF (2.0 mL) was added hydrazine monohydrate (36 μL, 0.72 mmol, 64–65 wt%). The resulting mixture was stirred for 30 min at room temperature and then cooled to 0 °C. Activated MnO_2 (2.4 mmol) and $MgSO_4$ (2.4 mmol) were added. The reaction mixture was stirred for 2 h at 0 °C and 1 h at room temperature, then filtered through celite and used immediately without further purification. When phenyldiazomethane was used instead of benzaldehyde hydrazone under the standard conditions, the desired product **4aa** was not observed (see Supplementary Figure 1a). This result excluded the participation of carbene or diazo compound species.

The 1,2-diphenylcyclopropane **22** was prepared following a literature procedure.[21] A dry Schlenk was charged with styrene (2.0 mmol), K_2CO_3 (1.5 mmol), *N*-Tosyl phenylhydrazone (1.0 mmol) and dioxane (5.0 mL) under nitrogen atmosphere. The reaction mixture was heated at 110 ^oC, stirred and refluxed for 6 h. After the mixture was cooled to room temperature, diluted with dichloromethane (10.0 mL) and filtered through celite. The solvent was evaporated in vacuo and the residue was purified by flash column chromatography on silica gel with hexane to give the product **22** (*trans*:*cis* = 4.3:1). ^1H NMR (500 MHz, $CDCl_3$) δ 7.34-7.31 (m, 4H), 7.23-7.20 (m, 2H), 7.18-7.16 (m, 4H), 7.13-7.10 (m, 0.92H), 7.08-7.05 (m, 0.46H), 6.98-6.96 (m, 0.92H), 2.51 (dd, *J* = 8.6, 6.4 Hz, 0.46H), 2.20 (dd, *J* = 7.4, 6.0 Hz, 2H), 1.50-1.46 (m, 2.46H). The spectroscopic data for this substrate match the literature data.[21] When 1,2-diphenylcyclopropane **22** was used instead of benzaldehyde hydrazone and styrene under the standard conditions, the desired product **4aa** was not observed (see Supplementary Figure 1b). This result ruled out the pathway that ring opening of cyclopropane to give the desired product.

The 1-methoxy-4-(3-phenylpropyl)benzene **23** was prepared following a literature procedure.[22] In a glovebox, a flame-dried reaction tube equipped with a magnetic stir bar was charged with $Ni(cod)_2$ (11.2 mg, 10 mol%), PMe_3 (17 µL, 40 mol%) and 1,4-dioxane (2.0 mL) before being sealed with a rubber septum and taken out of the glove box. The reaction mixture was stirred at room temperature for 30 min. Then 4-methoxyphenyl triflate (0.2 mmol), (3-bromopropyl)benzene (0.6 mmol), hydrazine solution (1 M in THF, 0.4 mmol, 400 µL) and K_3PO_4 (1.2 mmol) were added sequentially. After that, the reaction mixture was sealed with aluminum cap, moved out of glovebox and stirred at 110 ℃ for 12 h. After the mixture was cooled to room temperature, the resulting solution was directly filtered through a pad of silica and washed with EtOAc (5.0 mL). The solvent was evaporated *in vacuo* to give the crude product. The residue was purified by flash column chromatography on silica gel (ethyl acetate/petroleum ether) to give the pure substrate **23**. ^1H NMR (500 MHz, $CDCl_3$) δ 7.34-7.30 (m, 2H), 7.23-7.20 (m, 3H), 7.14 (d, *J* = 8.6 Hz, 2H), 6.87 (d, *J* = 8.6 Hz, 2H), 3.83 (s, 3H), 2.68 (t, *J* = 7.6 Hz, 2H), 2.64 (t, *J* = 7.6 Hz, 2H), 2.00-1.94 (m, 2H). The spectroscopic data for this product match the literature data.[22] When **23** was used under the standard conditions, the desired product **4aa** was not observed (see Supplementary Figure 1c). This result ruled out the hydrazone addition to the vinylarene and followed by dehydrogenation pathway.

When deuterated styrene (**3a**-D8) was reacted with hydrazone under the optimized conditions, the H/D exchanges both in the double bond moiety (73% D) and benzylic position (15% D) were observed in the corresponding product **4a**-D7. ^1H NMR (500 MHz, $CDCl_3$) δ 7.27-7.22 (m, 2H), 6.95 (td, *J* = 7.4, 1.0 Hz, 1H), 6.92 (d, *J* = 8.2 Hz, 1H), 6.50-6.46 (m, 0.27H), 6.44-6.37 (m, 0.27H), 3.89 (s, 3H), 3.59-3.58 (m, 1.7H); EI-MS (m/z): 231.2. This outcome revealed that there were iterative Ni-(H)D species addition/elimination steps during the reaction process (see Supplementary Figure 1f)

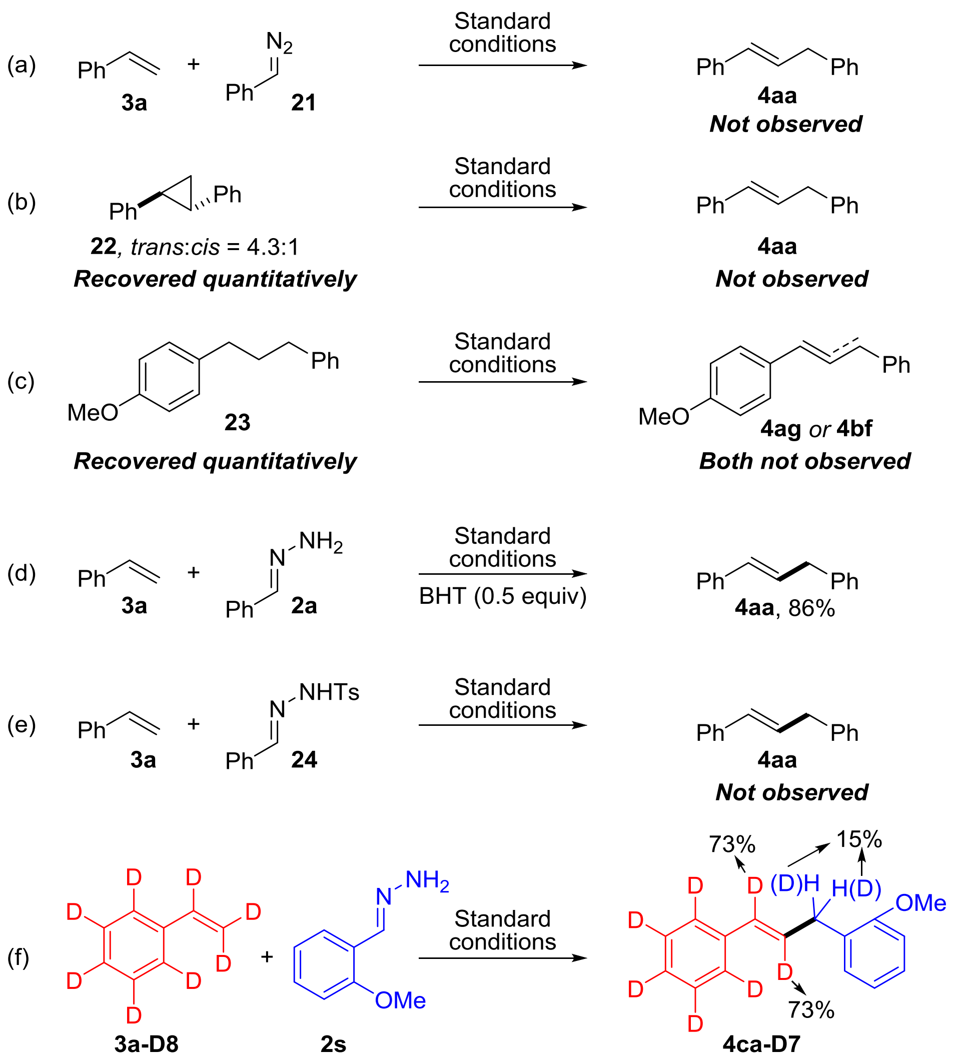

Supplementary Figure 1 Mechanistic studies. **a** Reaction of styrene with phenyldiazomethane did not give the desired product; **b** Reaction of 1,2-diphenylcyclopropane under the standard conditions did not give the desired product; **c** Reaction of 1-methoxy-4-(3-phenylpropyl)benzene under the standard conditions did not give the desired product; **d** Radical scavenger, 2,6-di-*tert*-butyl-4-methylphenol (BHT) added and the reaction was almost unaffected; **e** *N*-Ts hydrazone used instead of simple hydrazone did not give the desired product; **f** H/D exchanges occurred in the isotope experiment

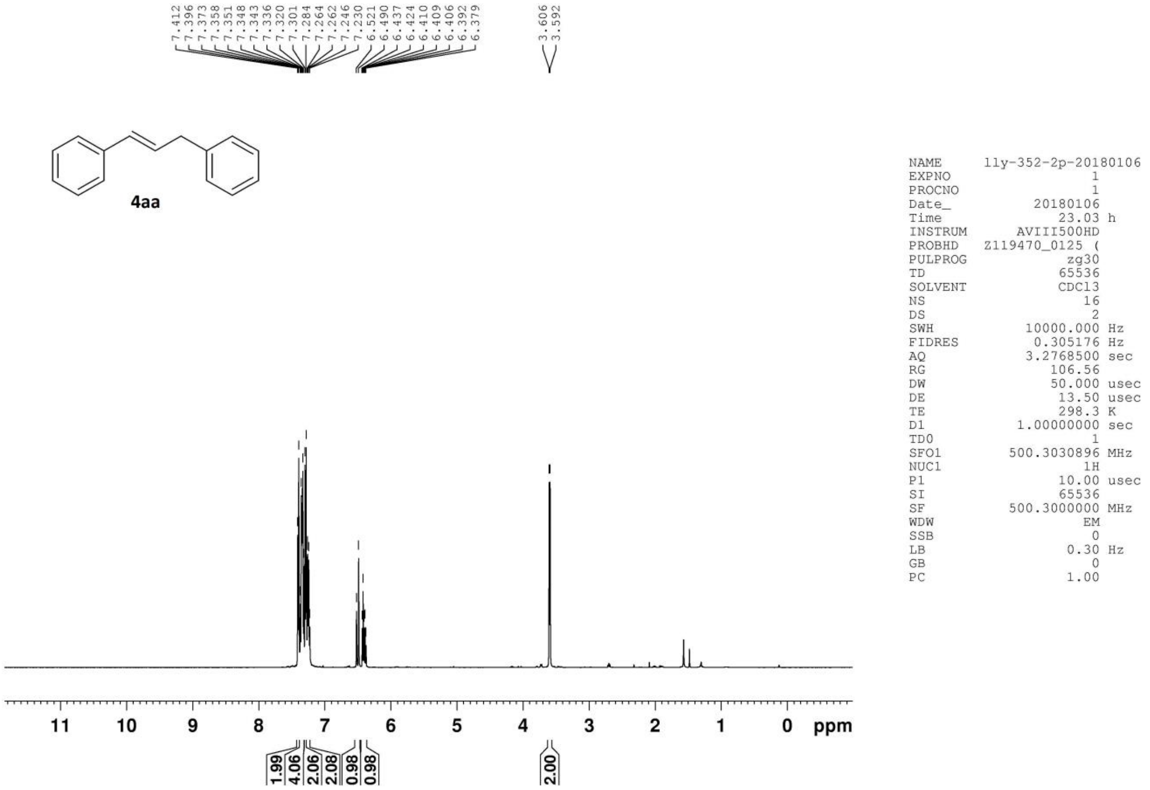

Supplementary Figure 2. ^{1}H NMR spectra for compound **4aa**

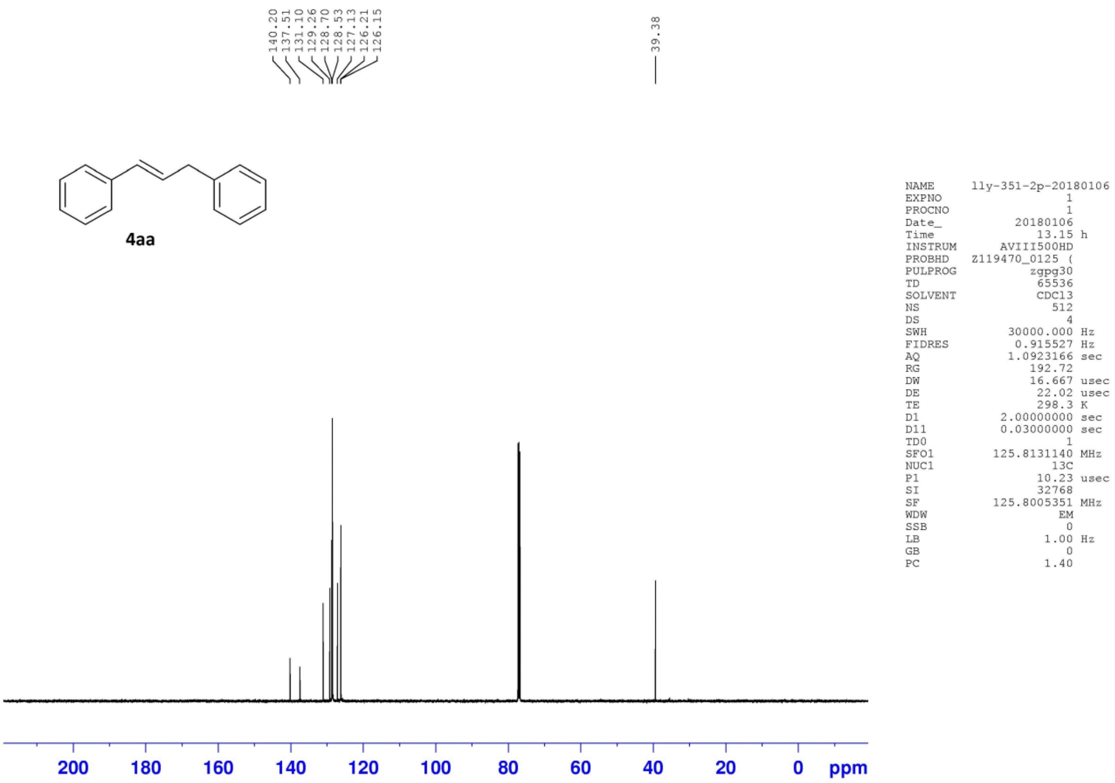

Supplementary Figure 3. ^{13}C NMR spectra for compound **4aa**

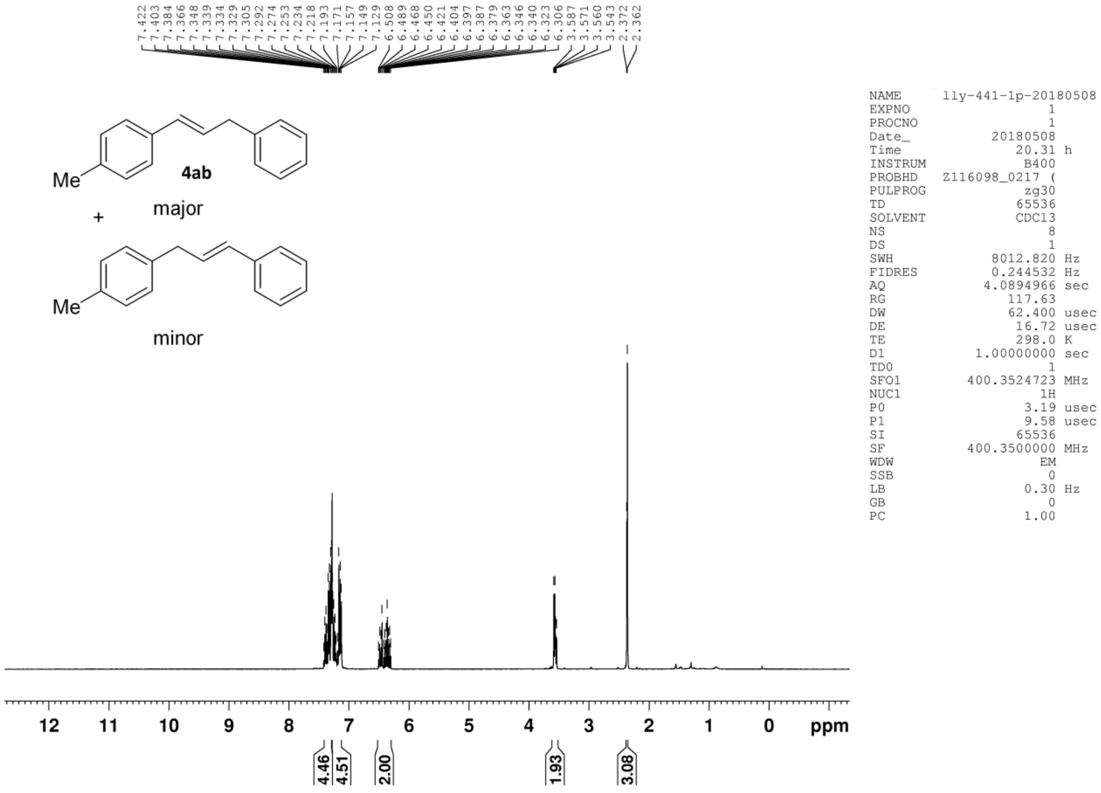

Supplementary Figure 4. ^{1}H NMR spectra for compound **4ab**

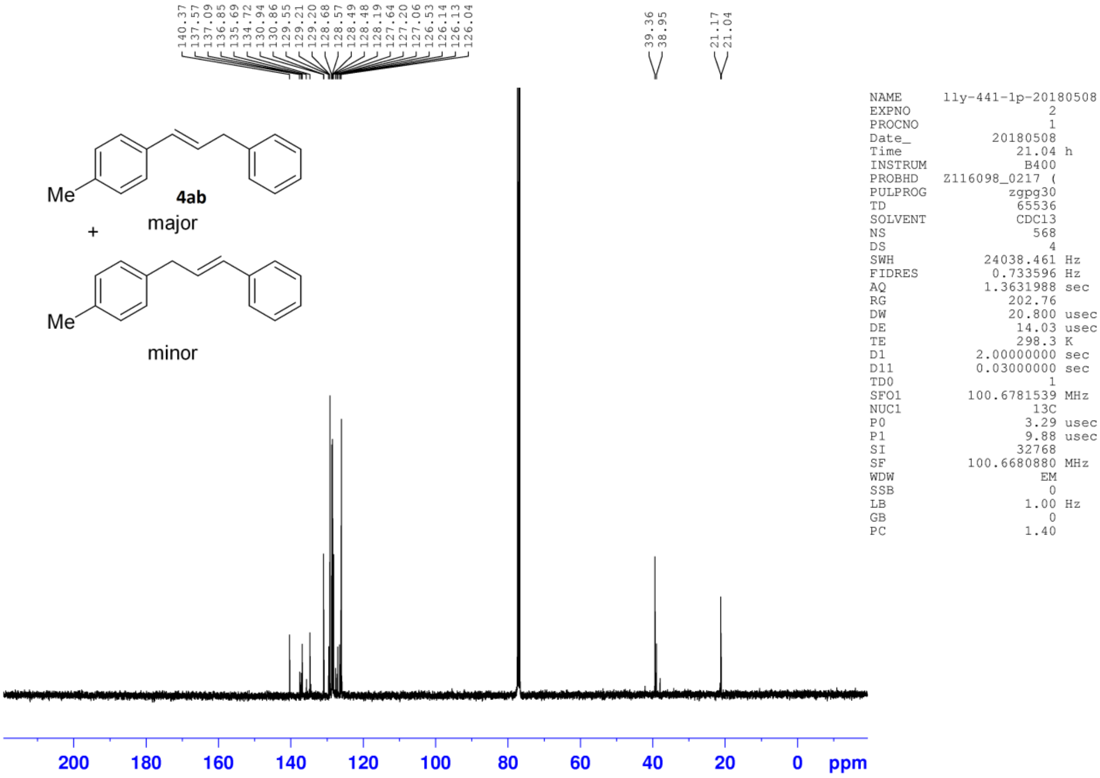

Supplementary Figure 5. ^{13}C NMR spectra for compound **4ab**

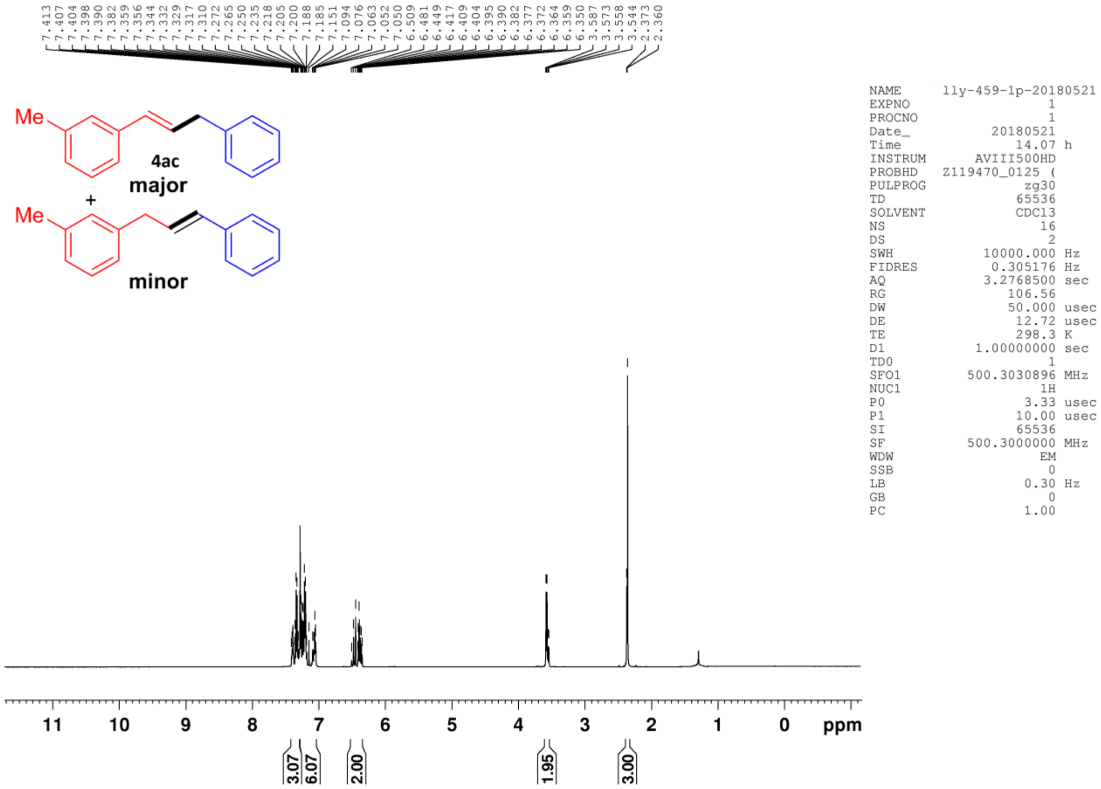

Supplementary Figure 6. ^{1}H NMR spectra for compound **4ac**

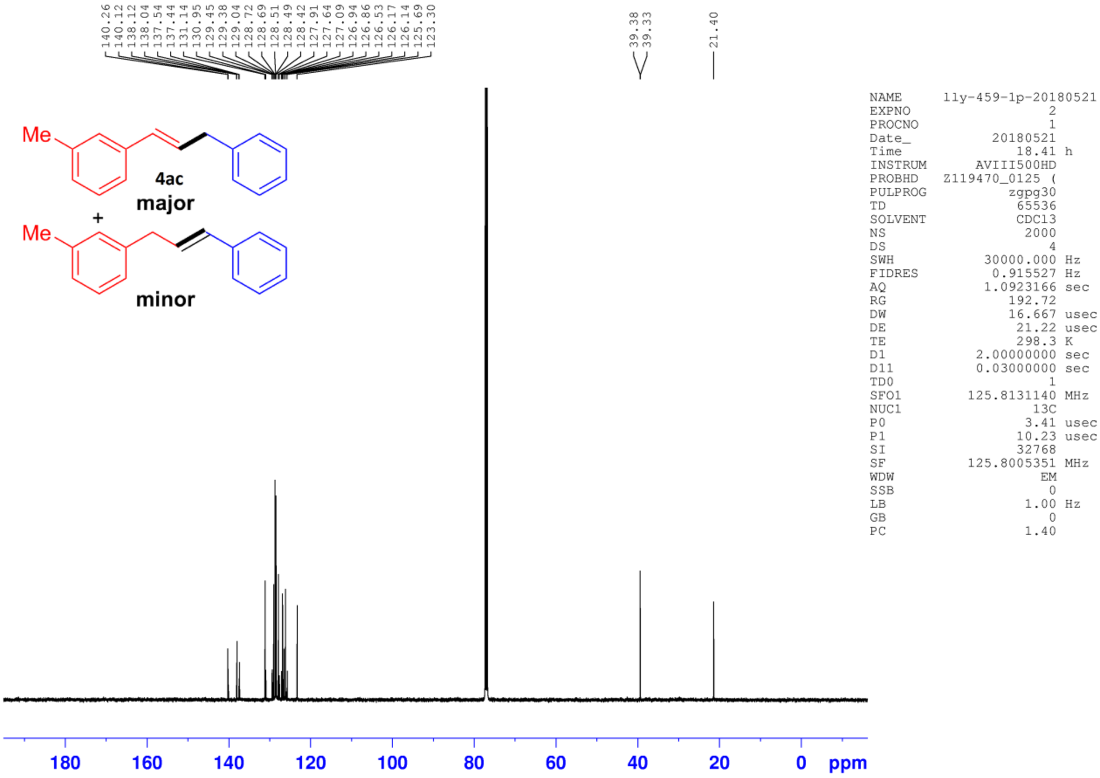

Supplementary Figure 7. ^{13}C NMR spectra for compound **4ac**

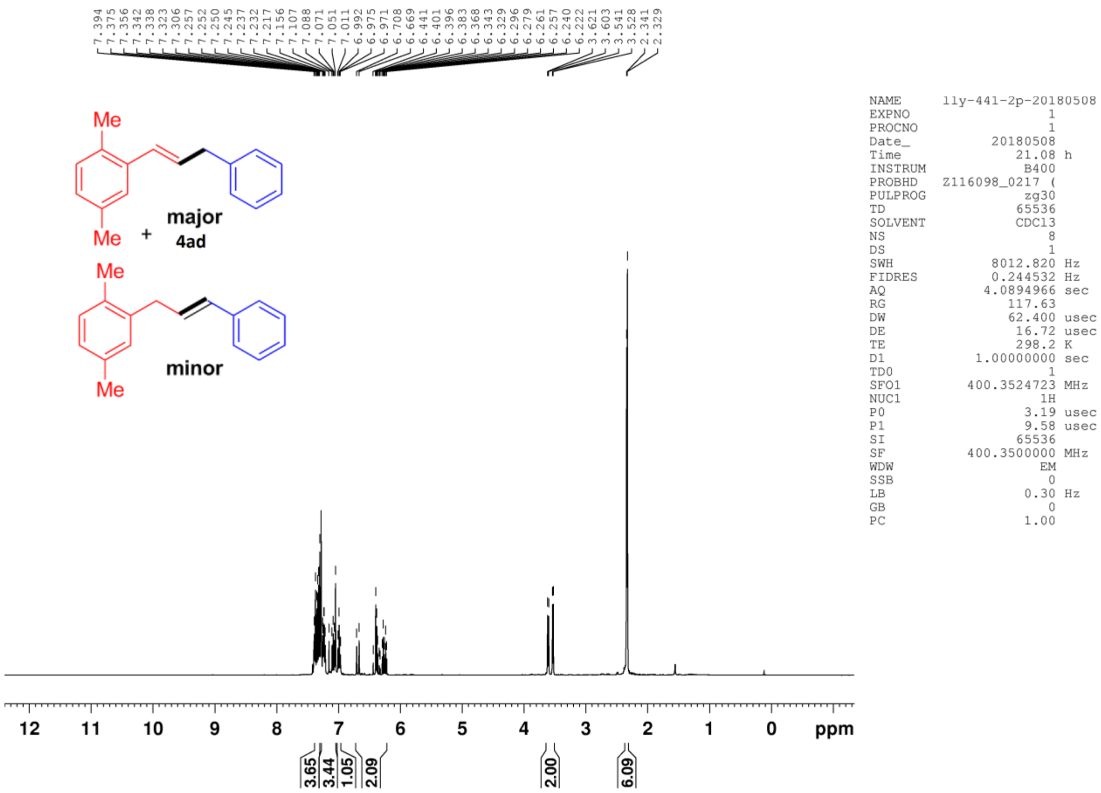

Supplementary Figure 8. ^{1}H NMR spectra for compound **4ad**

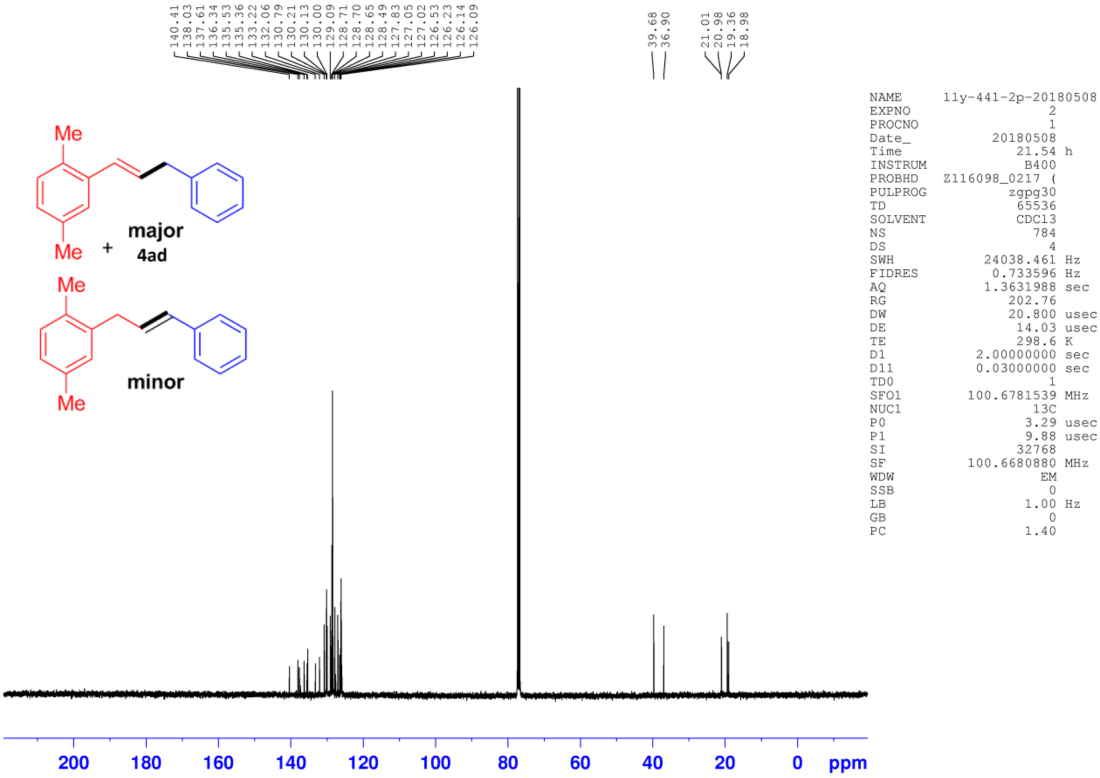

Supplementary Figure 9. ^{13}C NMR spectra for compound **4ad**

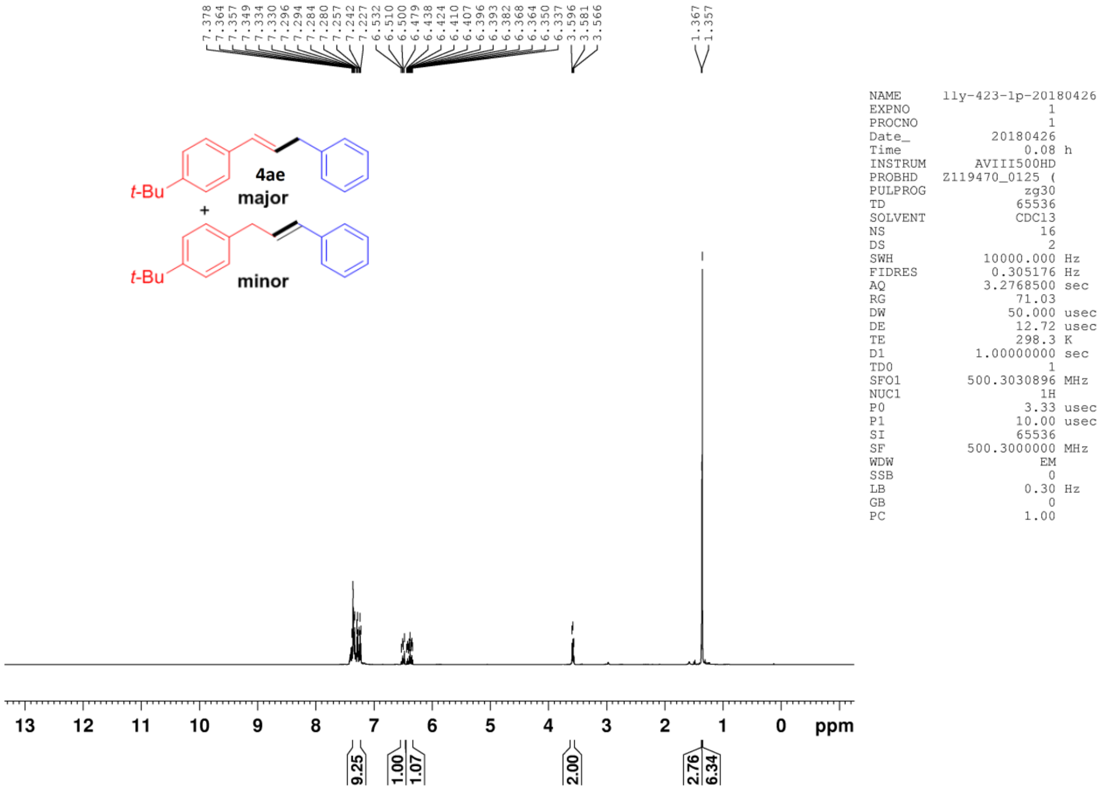

Supplementary Figure 10. ^{1}H NMR spectra for compound **4ae**

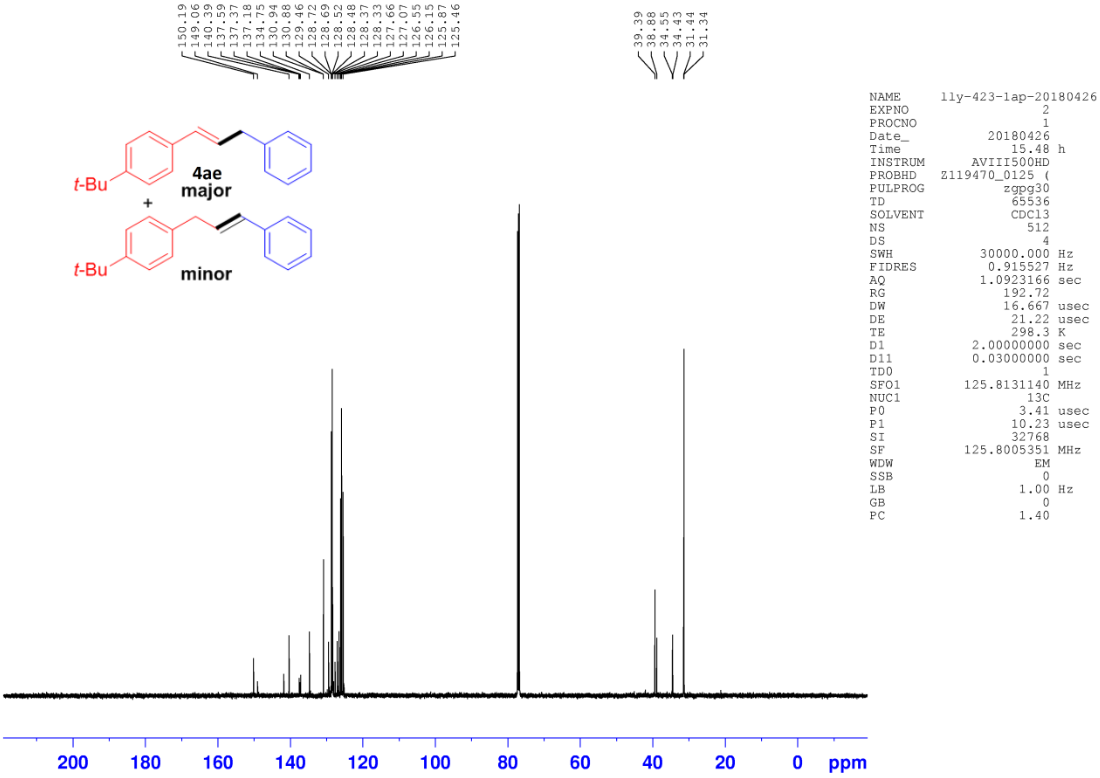

Supplementary Figure 11. ^{13}C NMR spectra for compound **4ae**

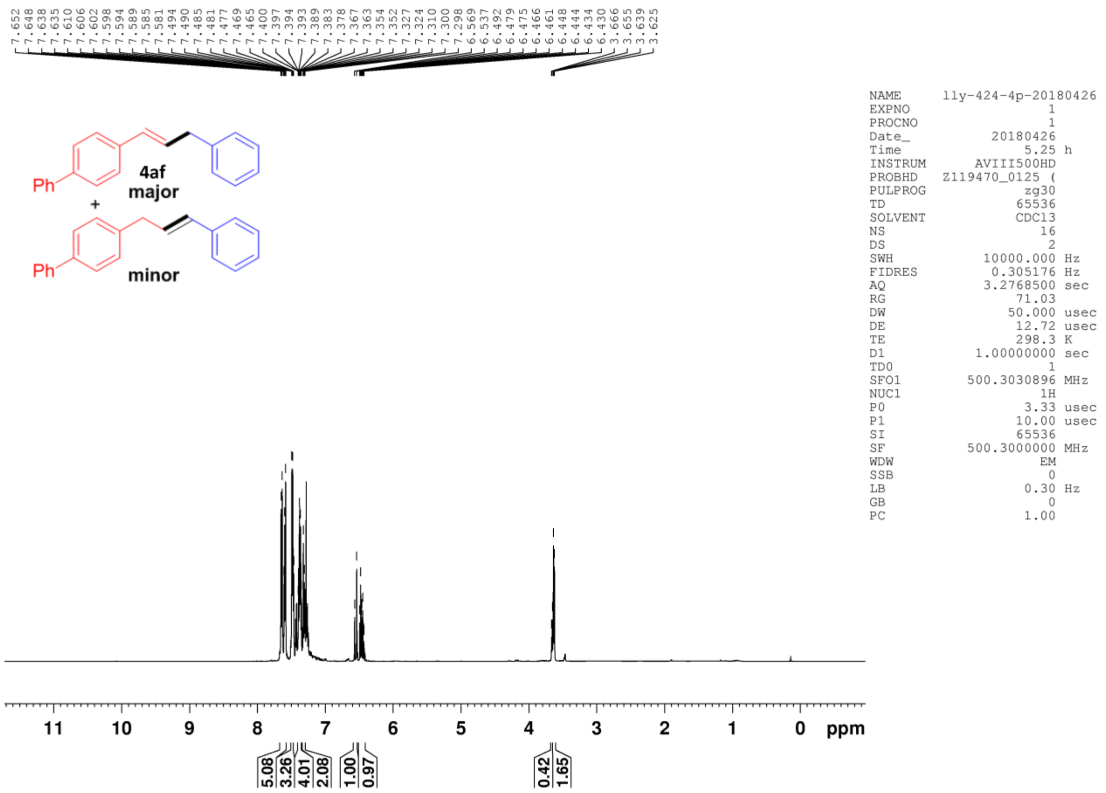

Supplementary Figure 12. ^{1}H NMR spectra for compound **4af**

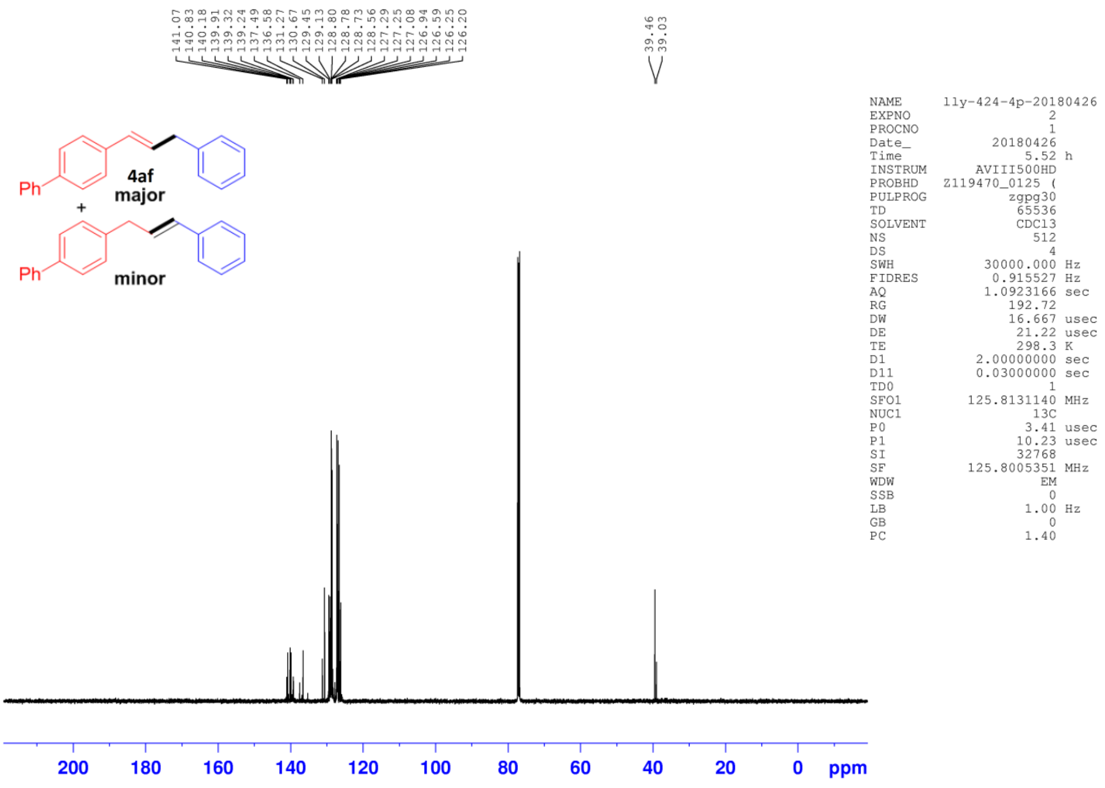

Supplementary Figure 13. ^{13}C NMR spectra for compound **4af**

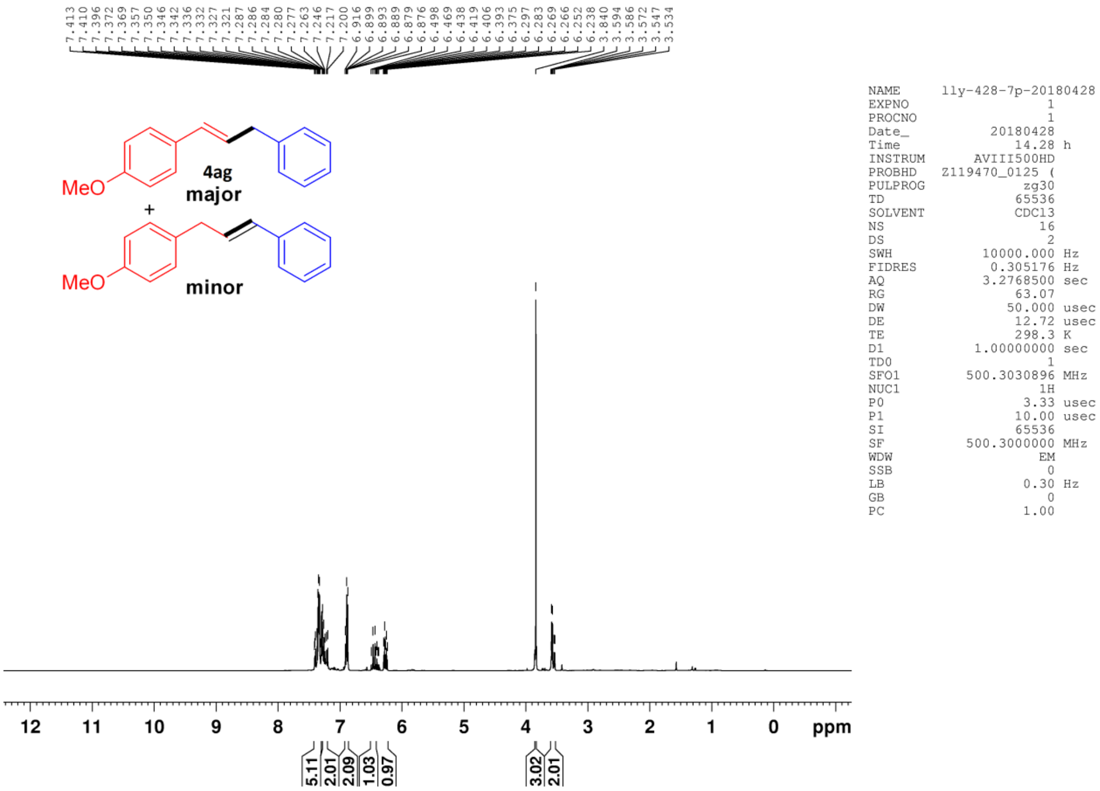

Supplementary Figure 14. ^{1}H NMR spectra for compound **4ag**

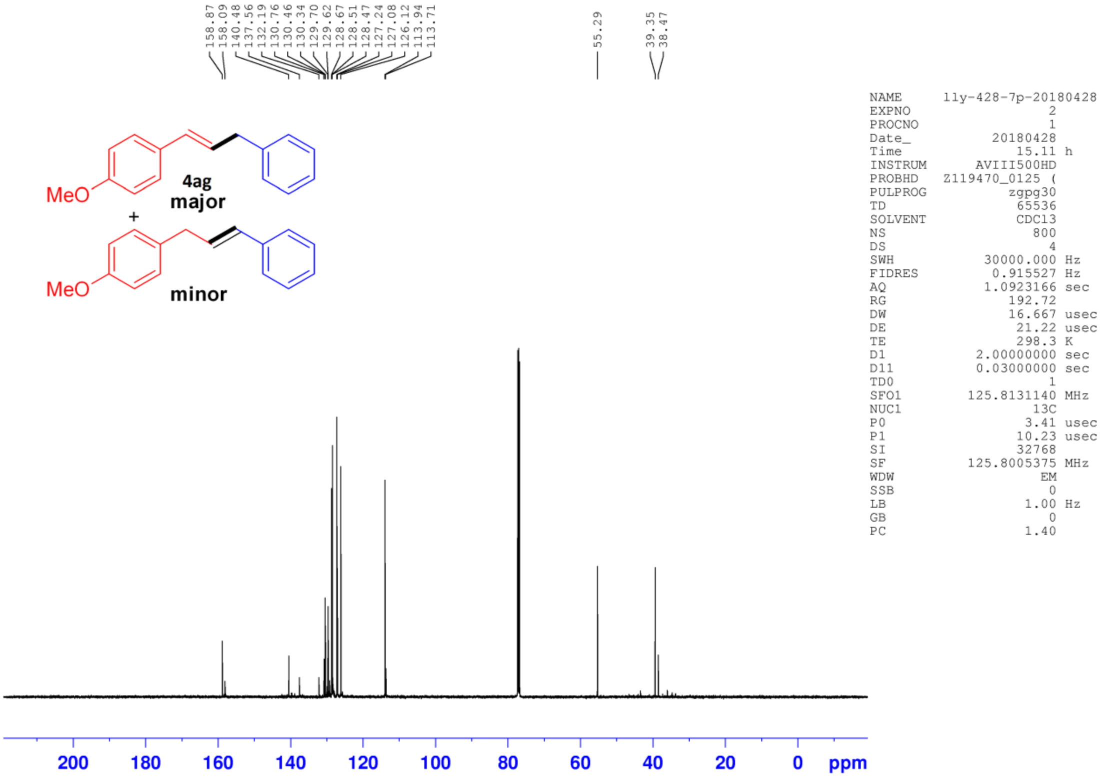

Supplementary Figure 15. ^{13}C NMR spectra for compound **4ag**

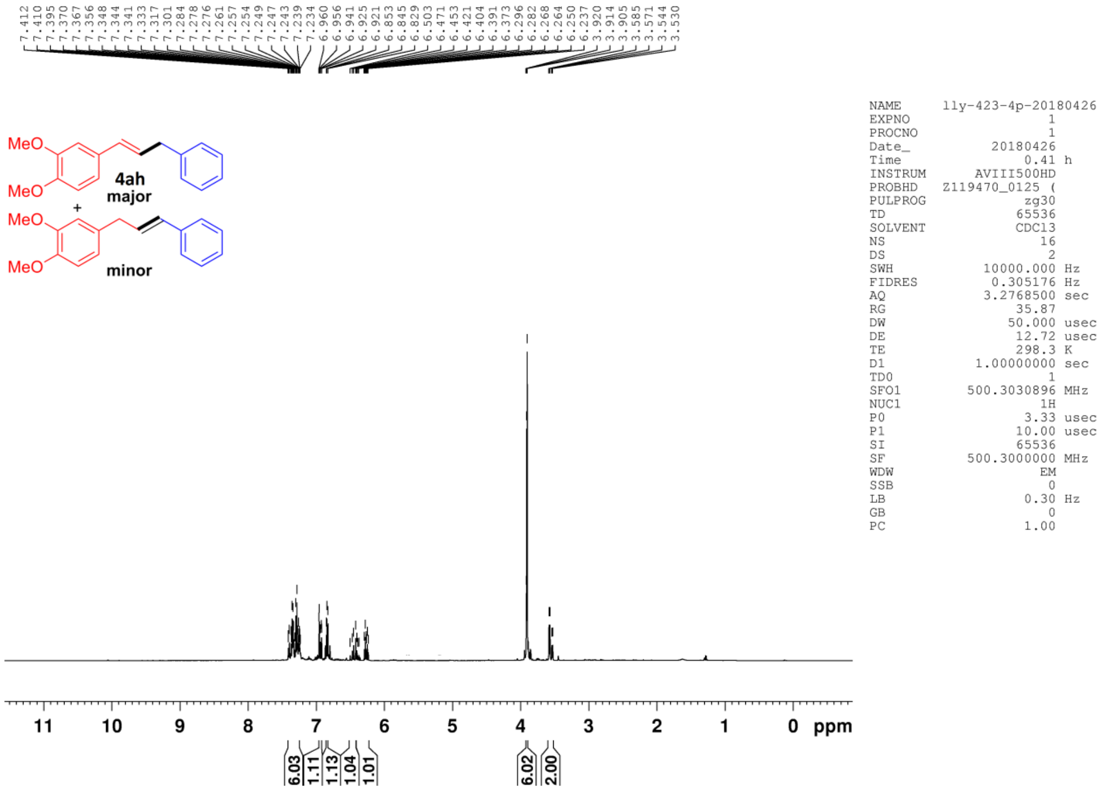

Supplementary Figure 16. ^{1}H NMR spectra for compound **4ah**

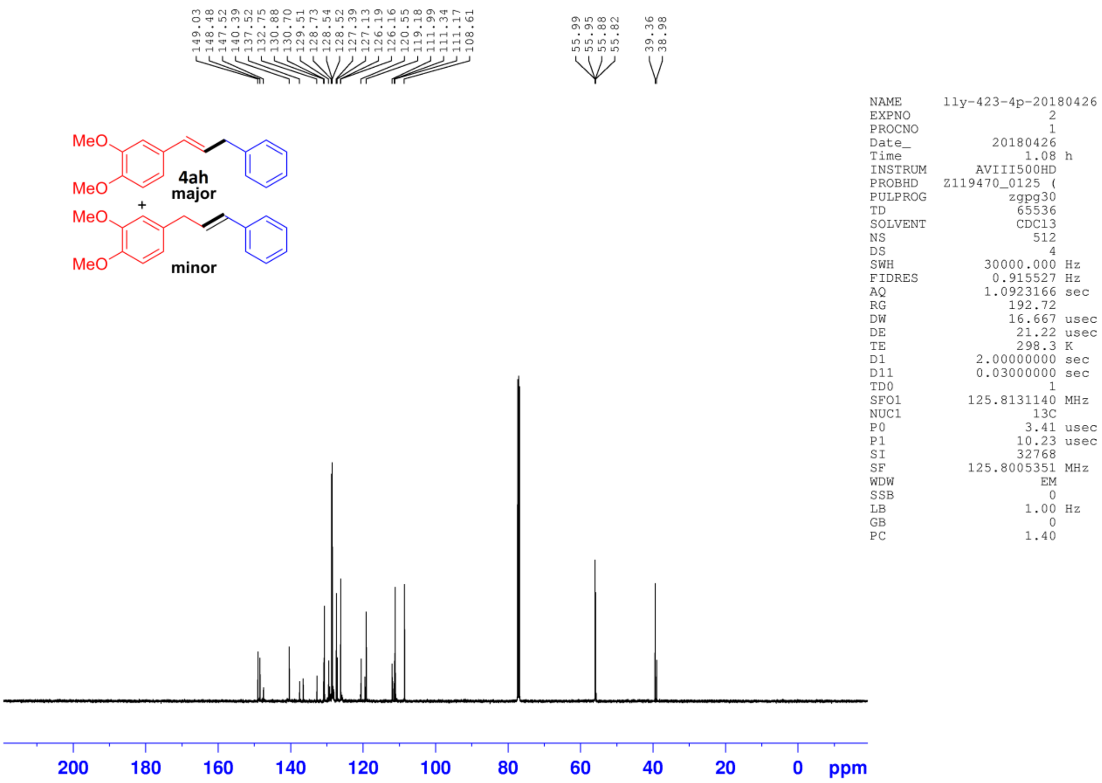

Supplementary Figure 17. ^{13}C NMR spectra for compound **4ah**

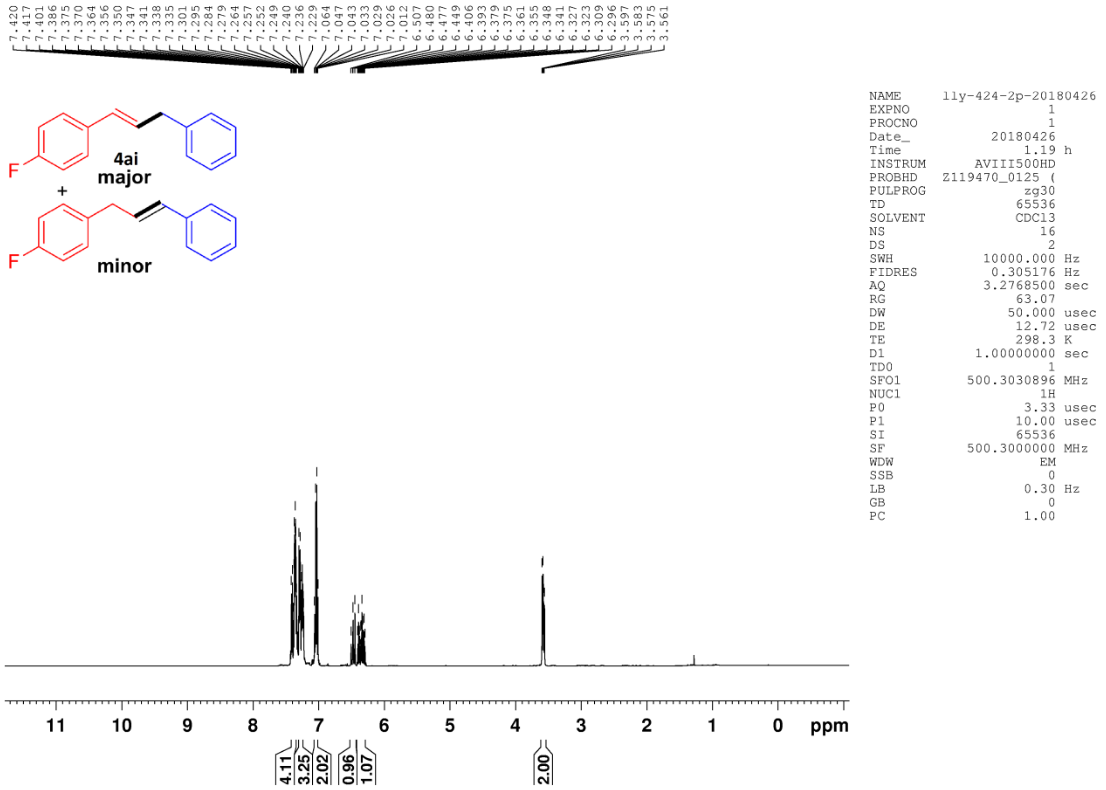

Supplementary Figure 18. ^{1}H NMR spectra for compound **4ai**

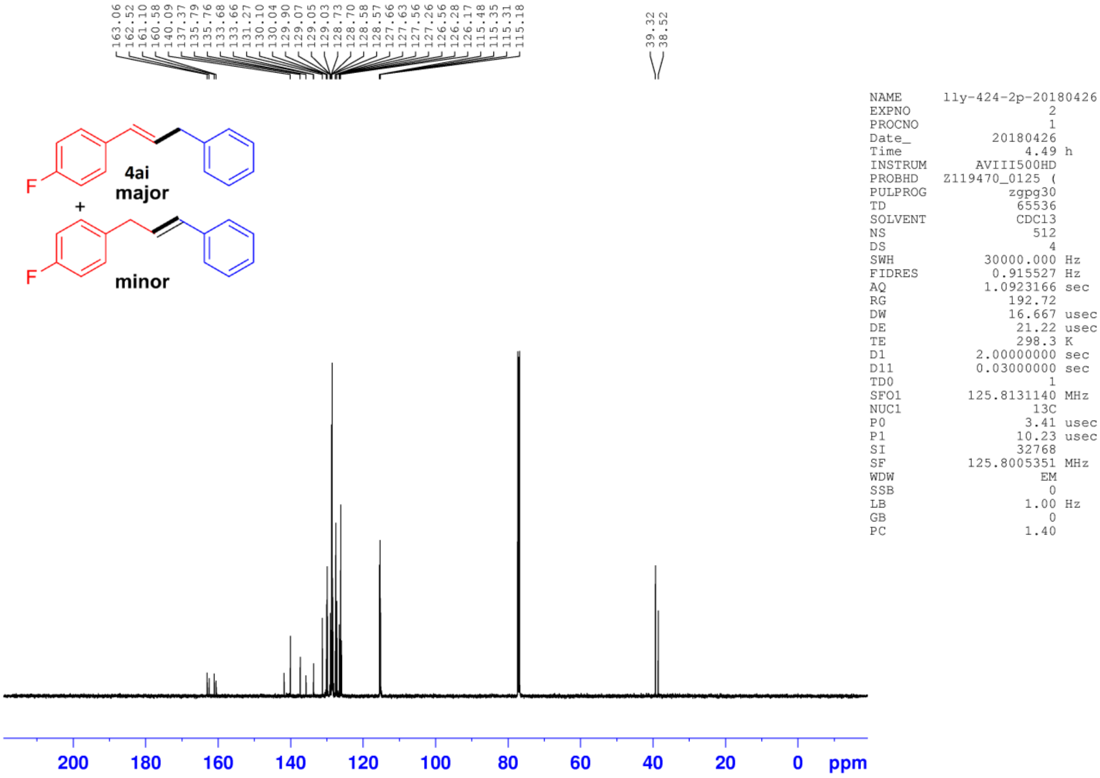

Supplementary Figure 19. ^{13}C NMR spectra for compound **4ai**

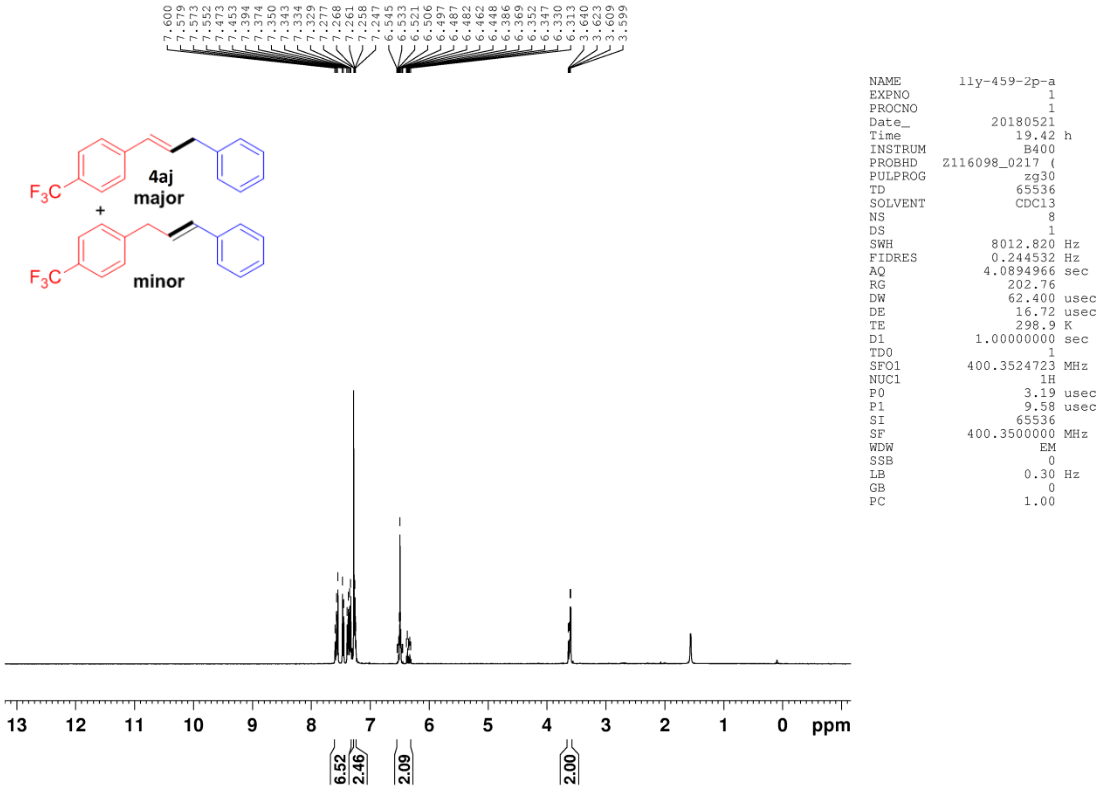

Supplementary Figure 20. ^{1}H NMR spectra for compound **4aj**

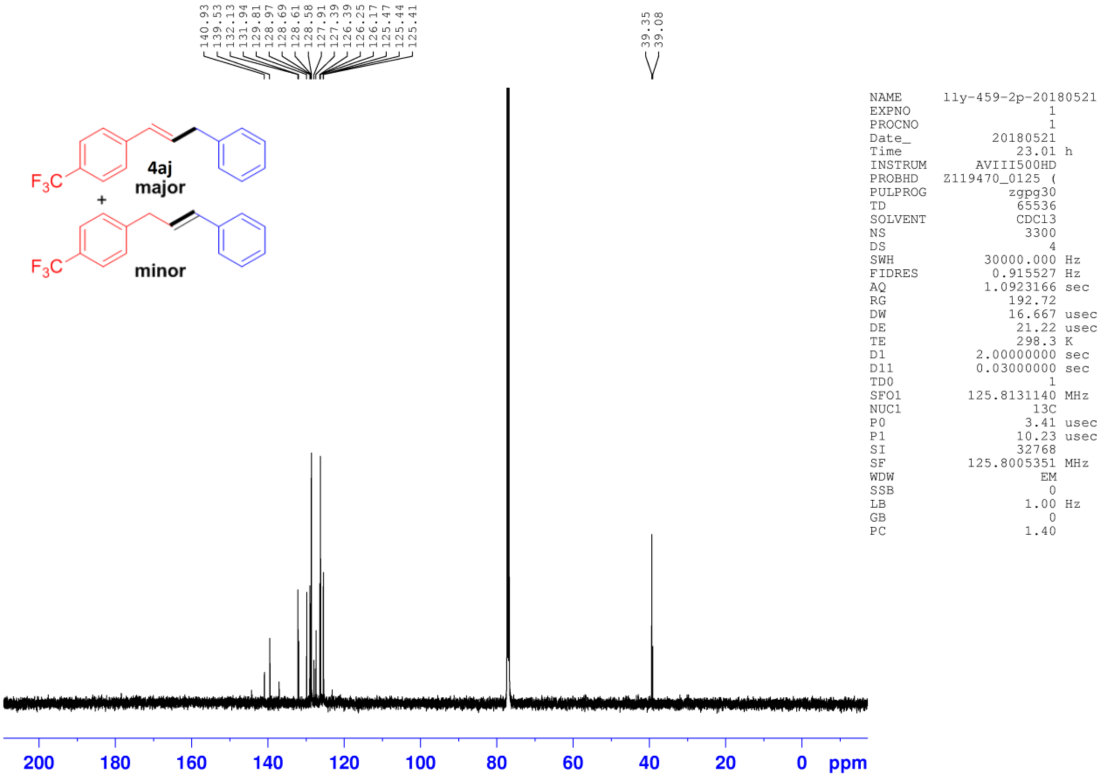

Supplementary Figure 21. ^{13}C NMR spectra for compound **4aj**

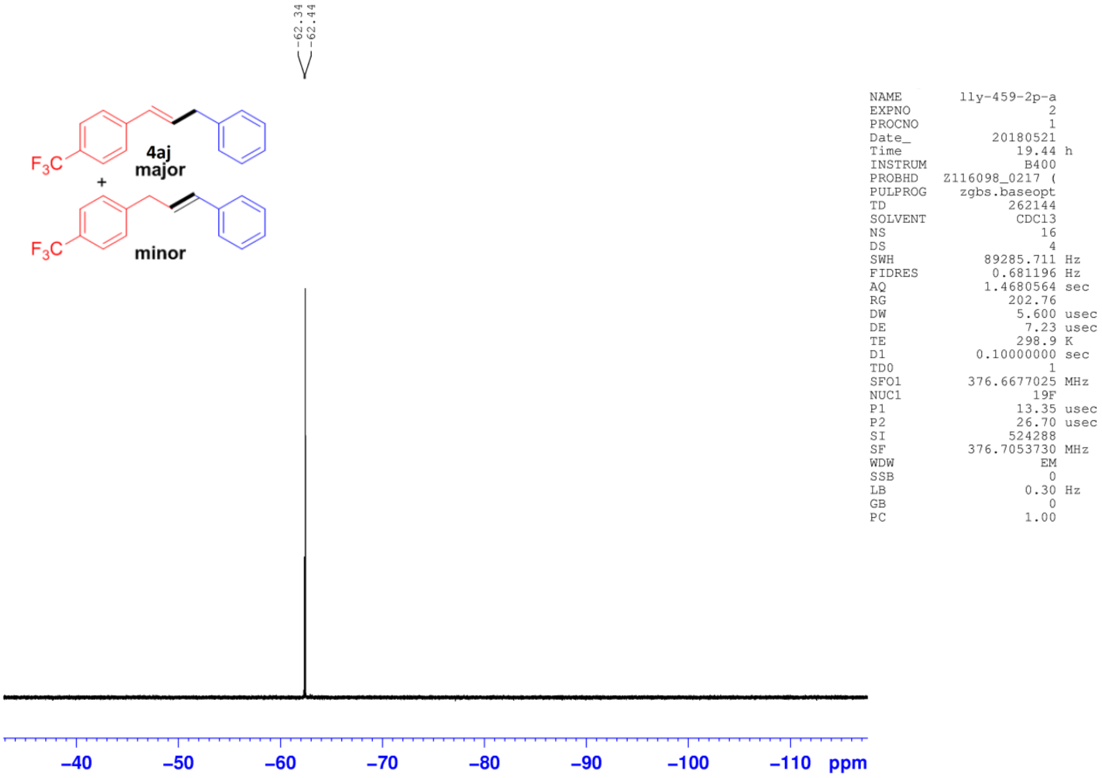

Supplementary Figure 22. ^{19}F NMR spectra for compound **4aj**

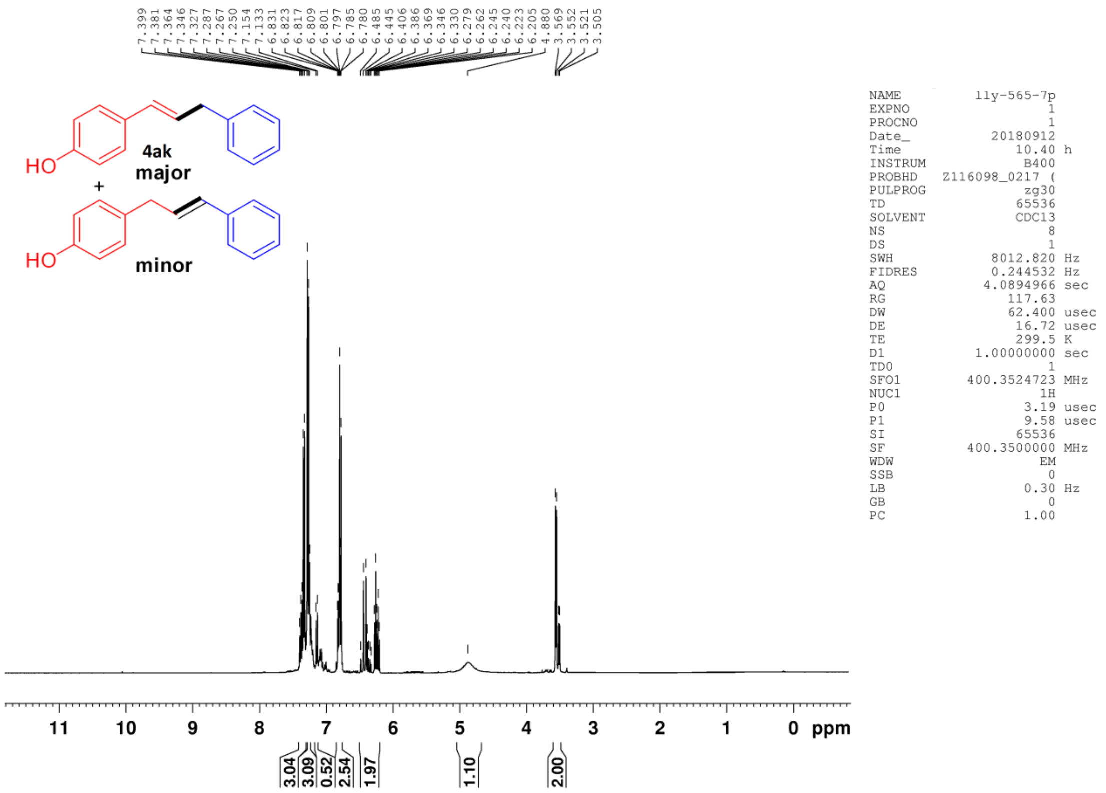

Supplementary Figure 23. ^{1}H NMR spectra for compound **4ak**

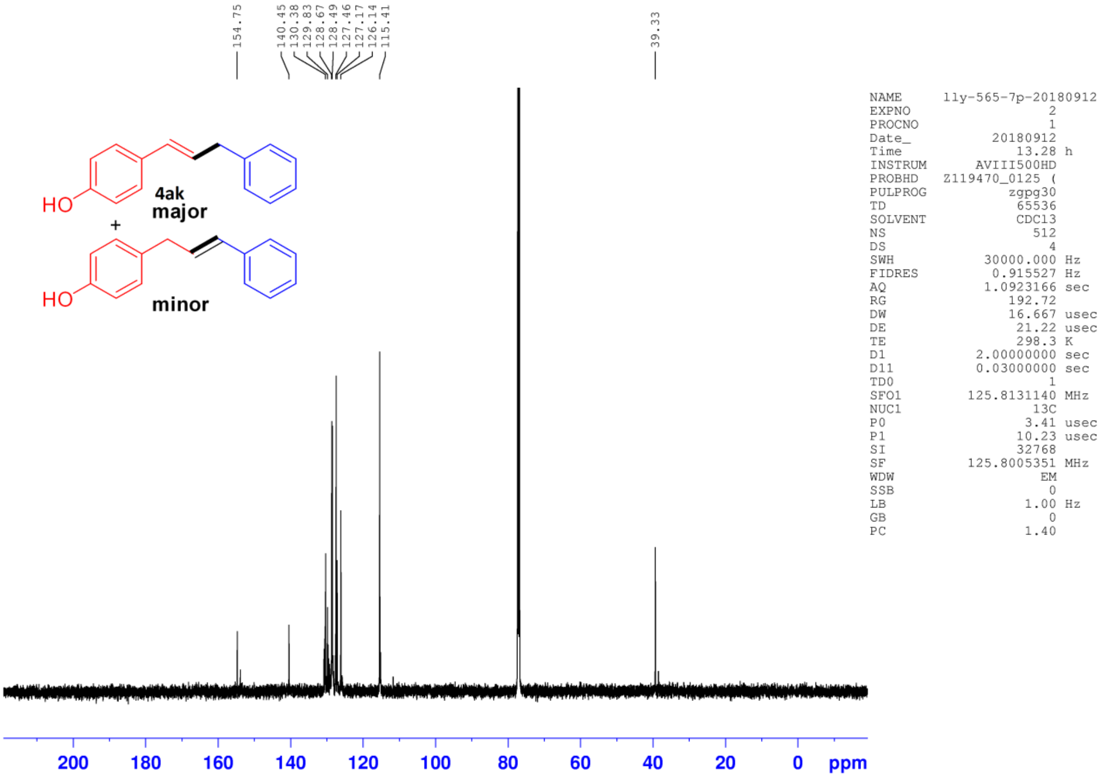

Supplementary Figure 24. ^{13}C NMR spectra for compound **4ak**

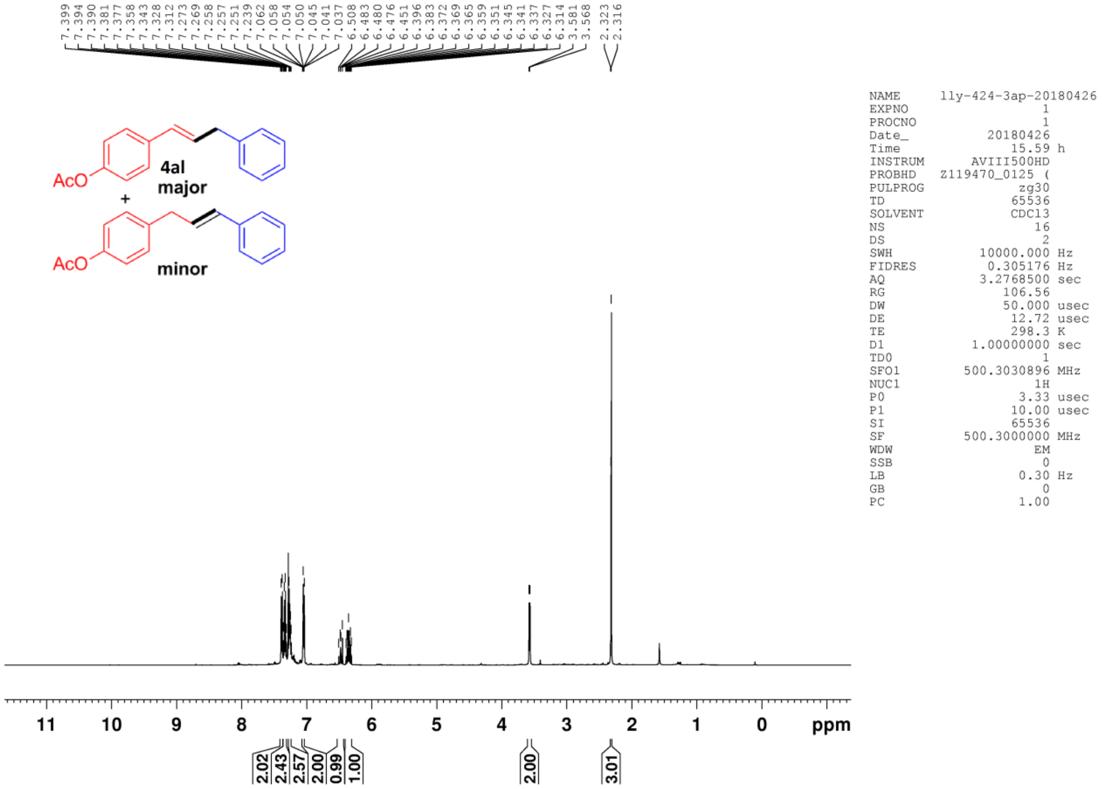

Supplementary Figure 25. ^{1}H NMR spectra for compound **4al**

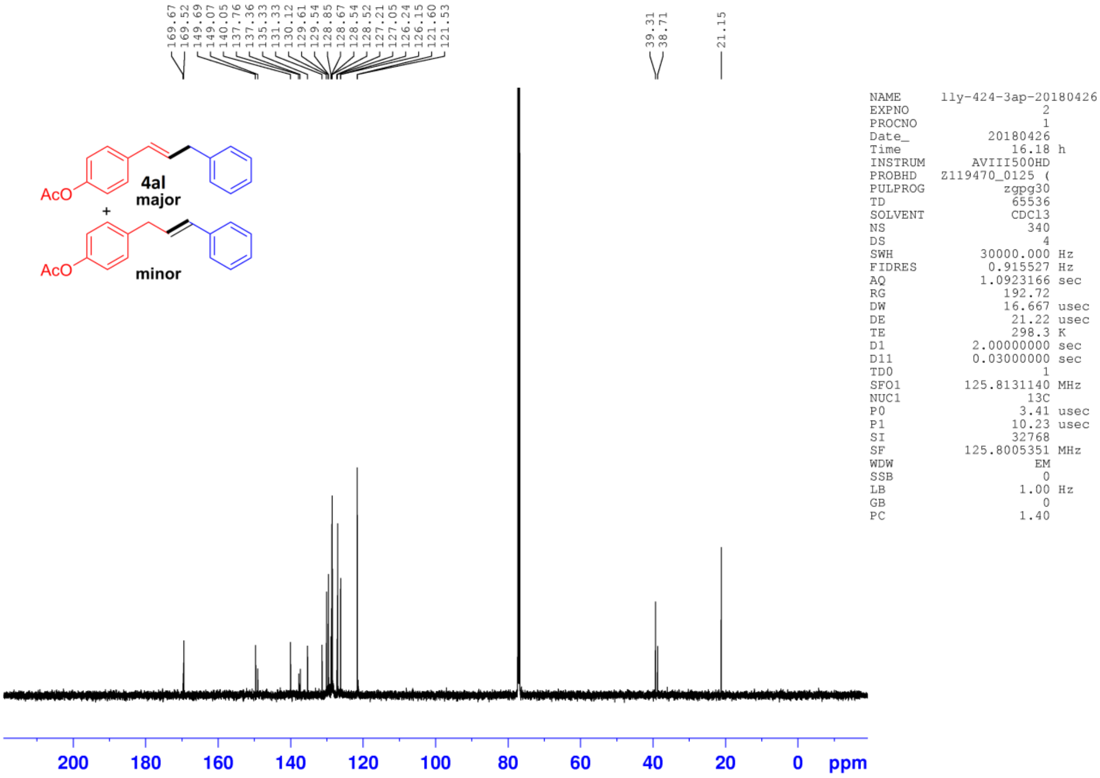

Supplementary Figure 26. ^{13}C NMR spectra for compound **4al**

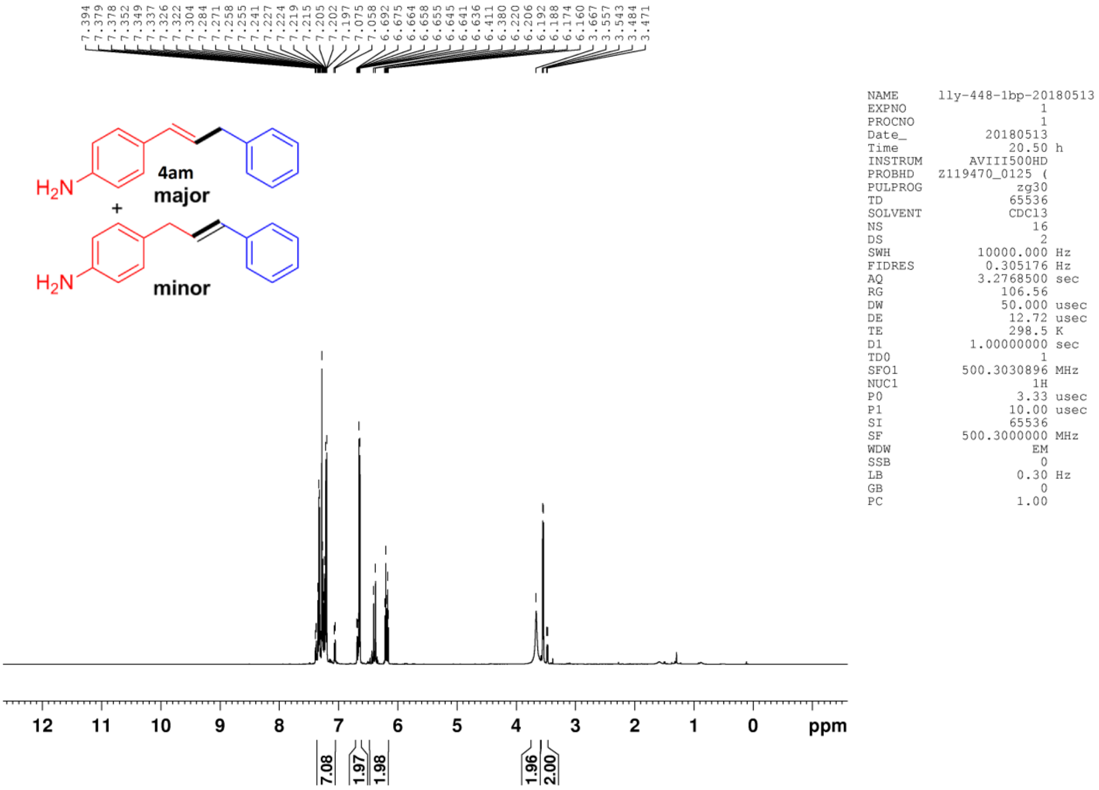

Supplementary Figure 27. ^{1}H NMR spectra for compound **4am**

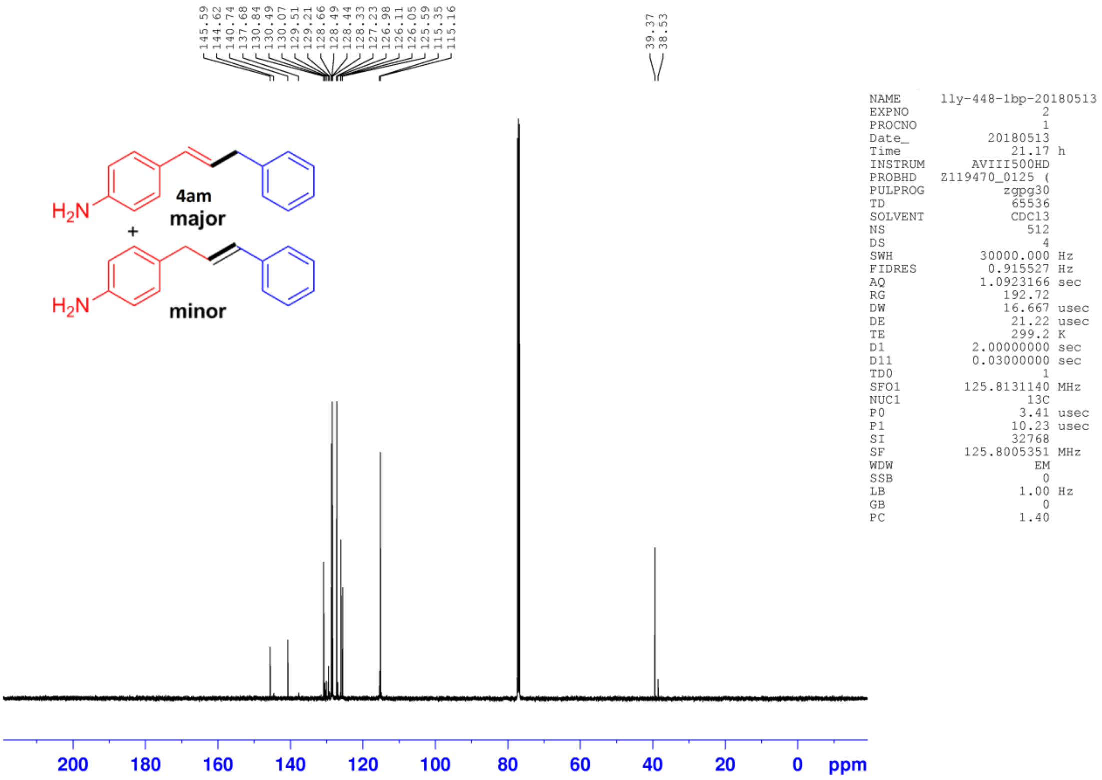

Supplementary Figure 28. ^{13}C NMR spectra for compound **4am**

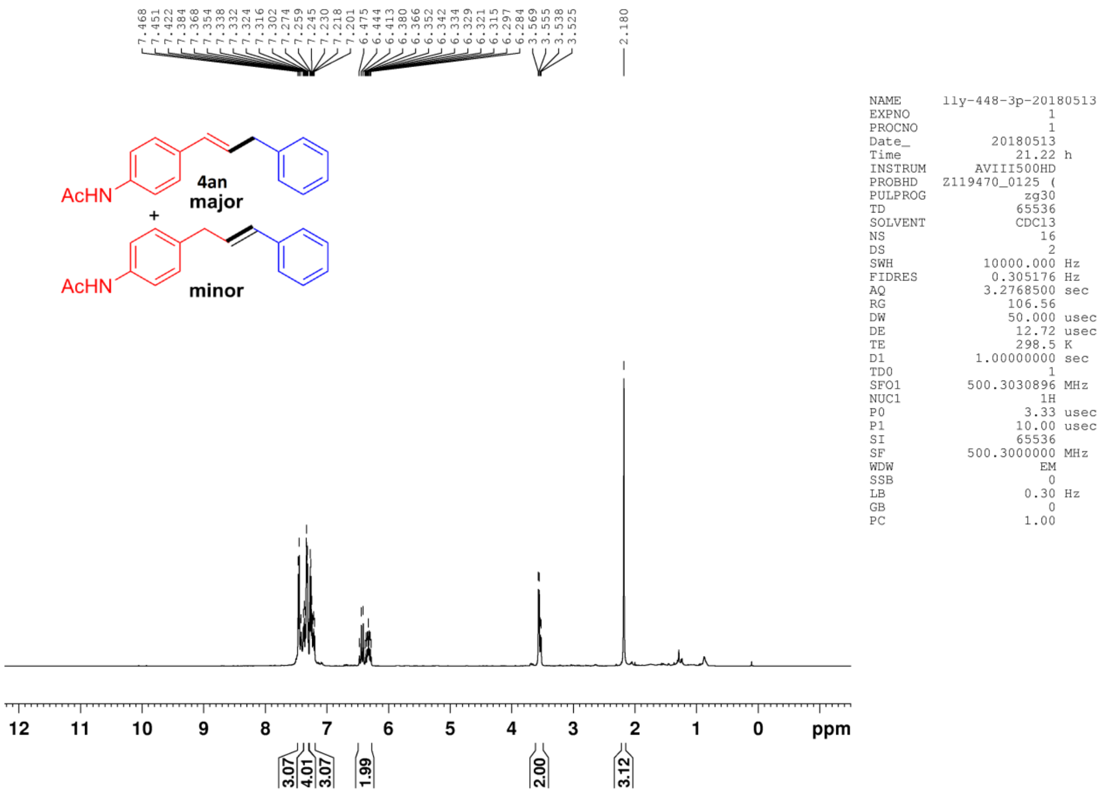

Supplementary Figure 29. ^{1}H NMR spectra for compound **4an**

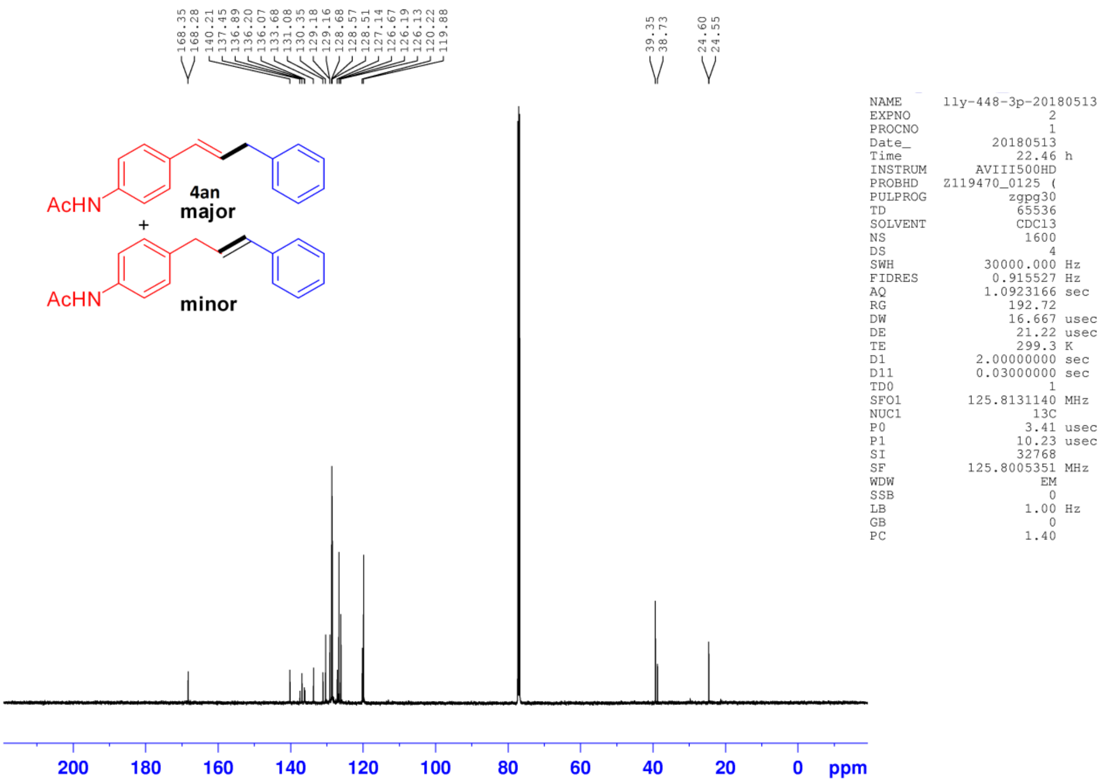

Supplementary Figure 30. ^{13}C NMR spectra for compound **4an**

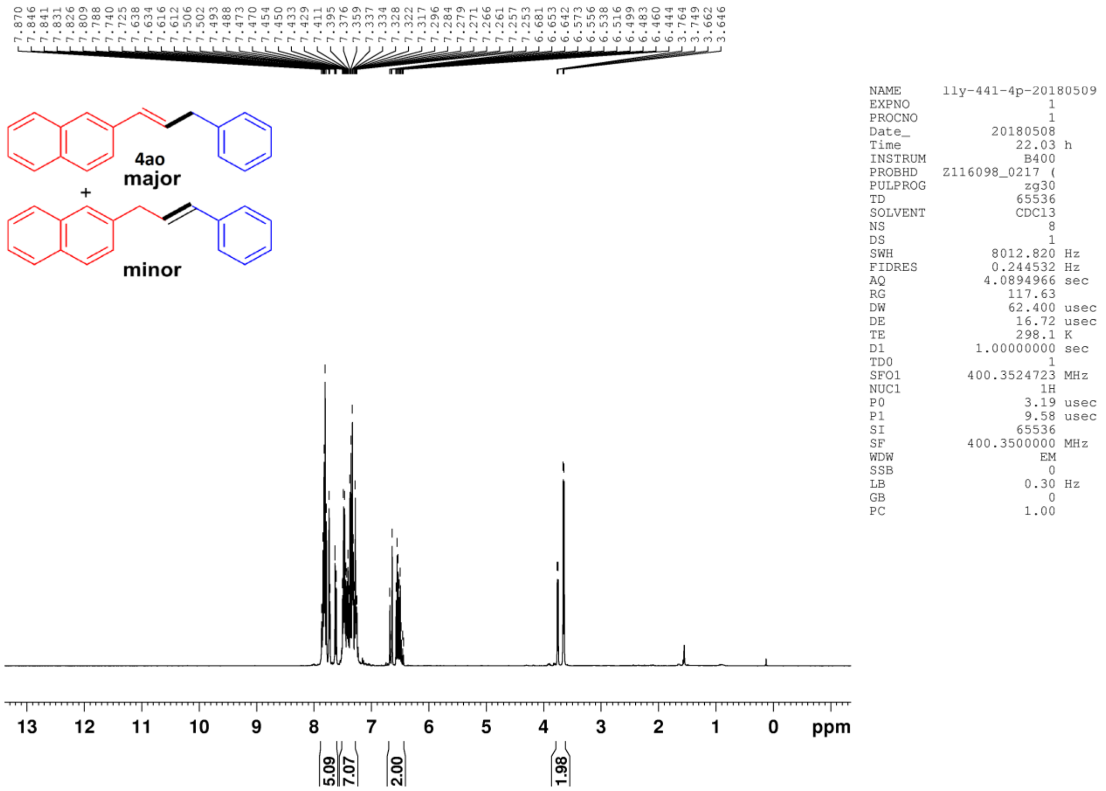

Supplementary Figure 31. ^{1}H NMR spectra for compound **4ao**

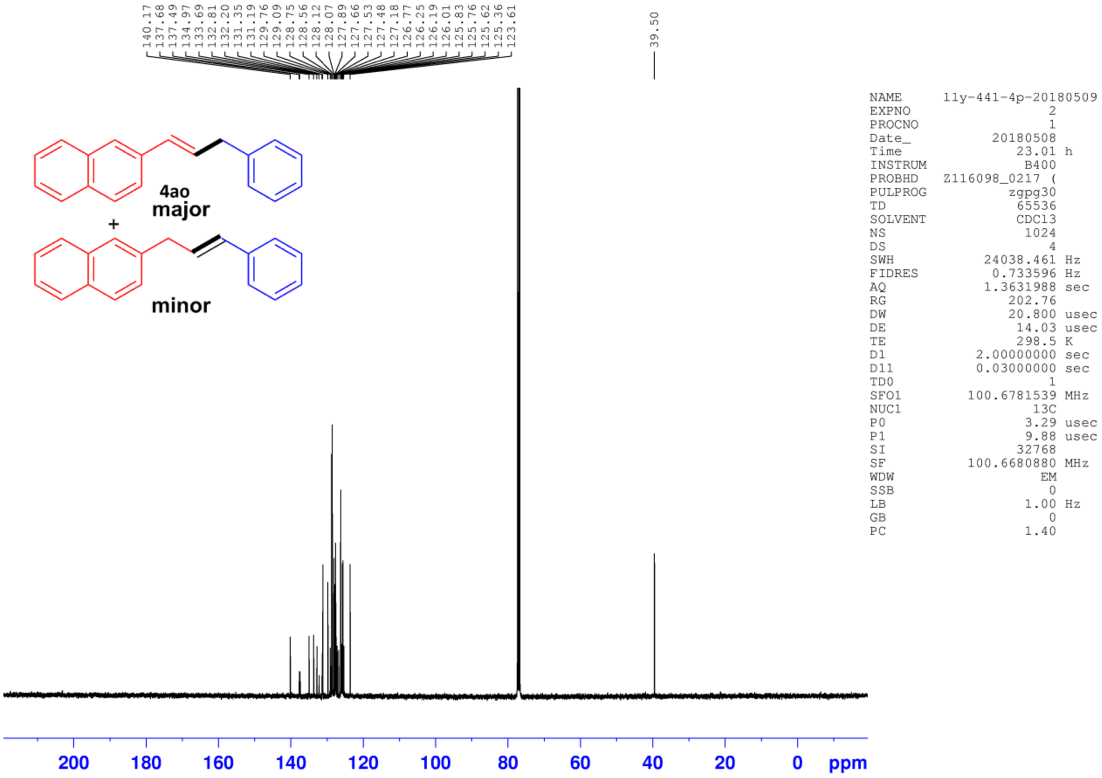

Supplementary Figure 32. ^{13}C NMR spectra for compound **4ao**

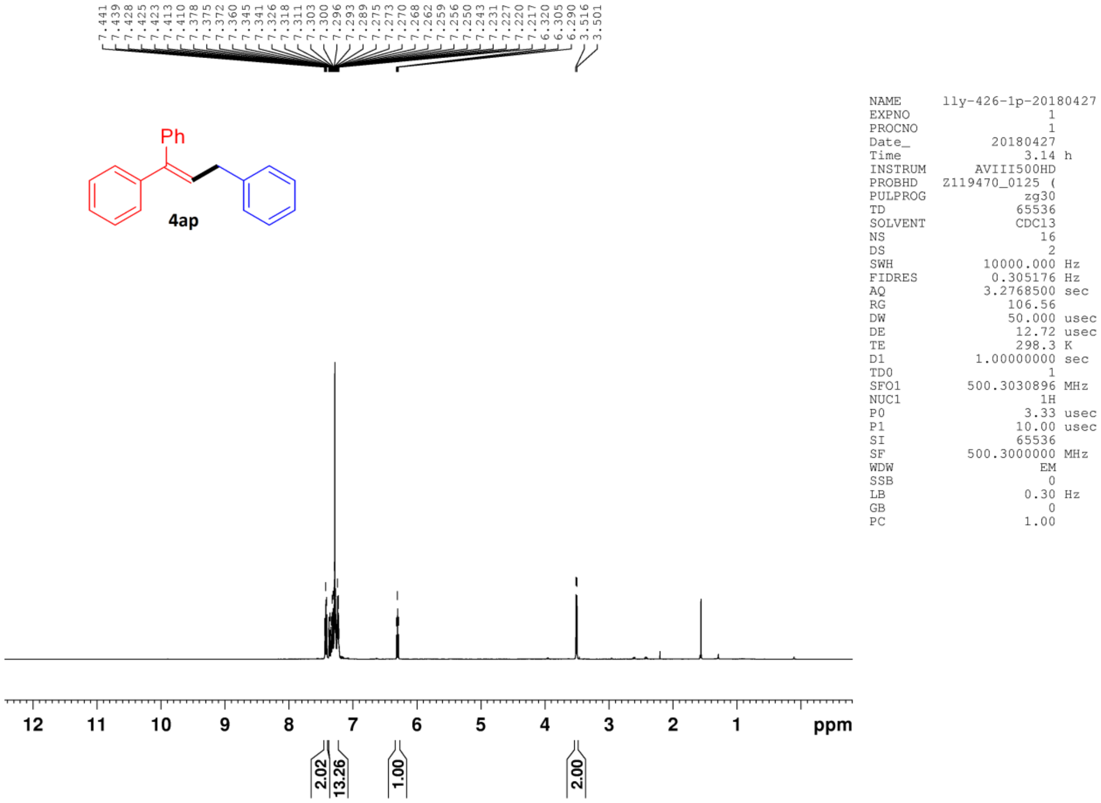

Supplementary Figure 33. ^{13}C NMR spectra for compound **4ap**

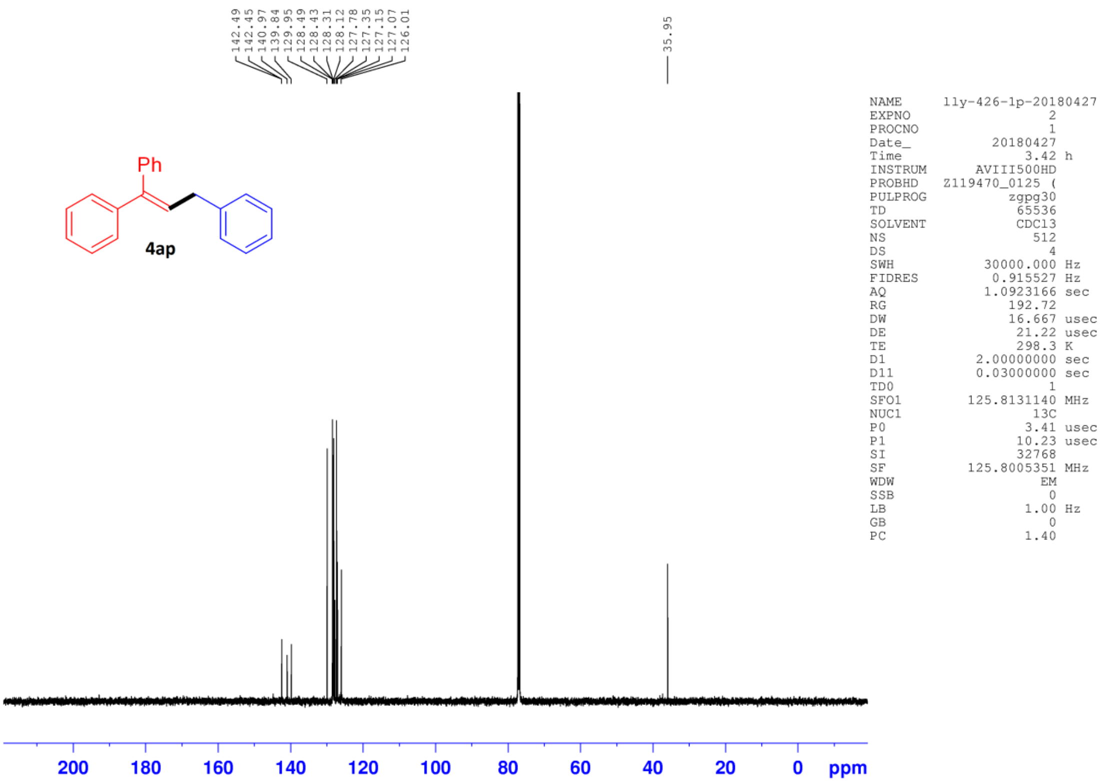

Supplementary Figure 34. ^{13}C NMR spectra for compound **4ap**

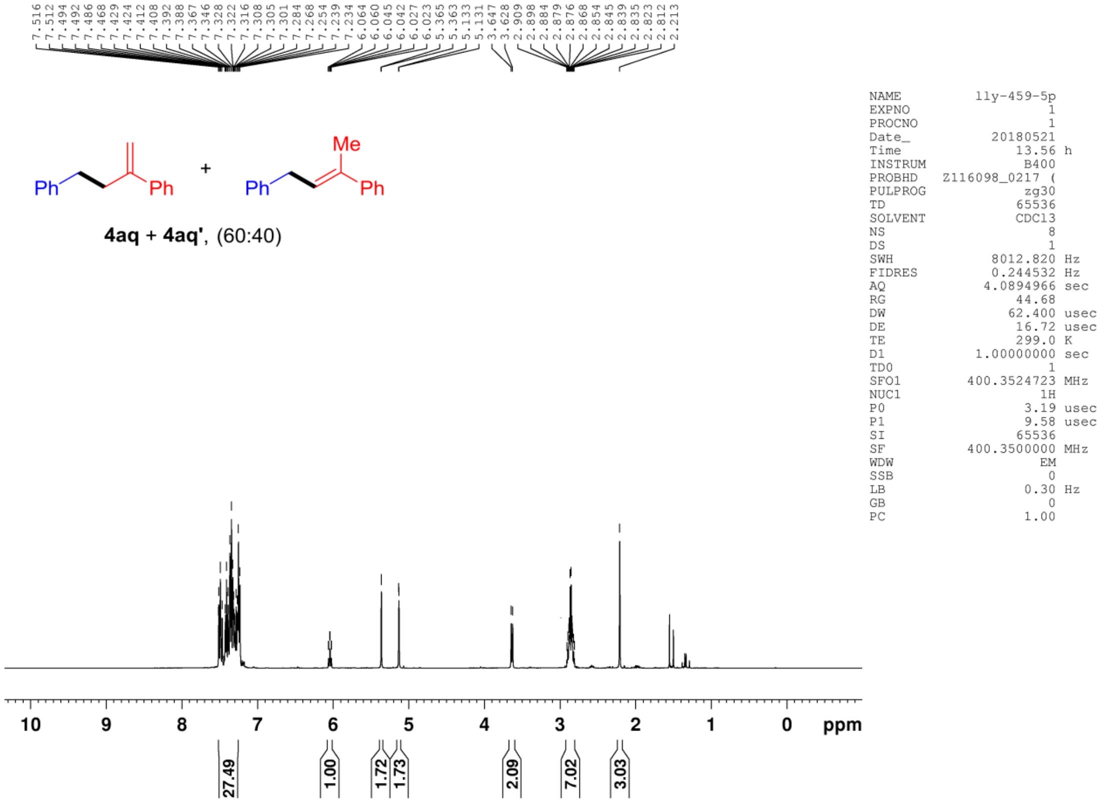

Supplementary Figure 35. ^{1}H NMR spectra for compound **4aq** and **4aq’**

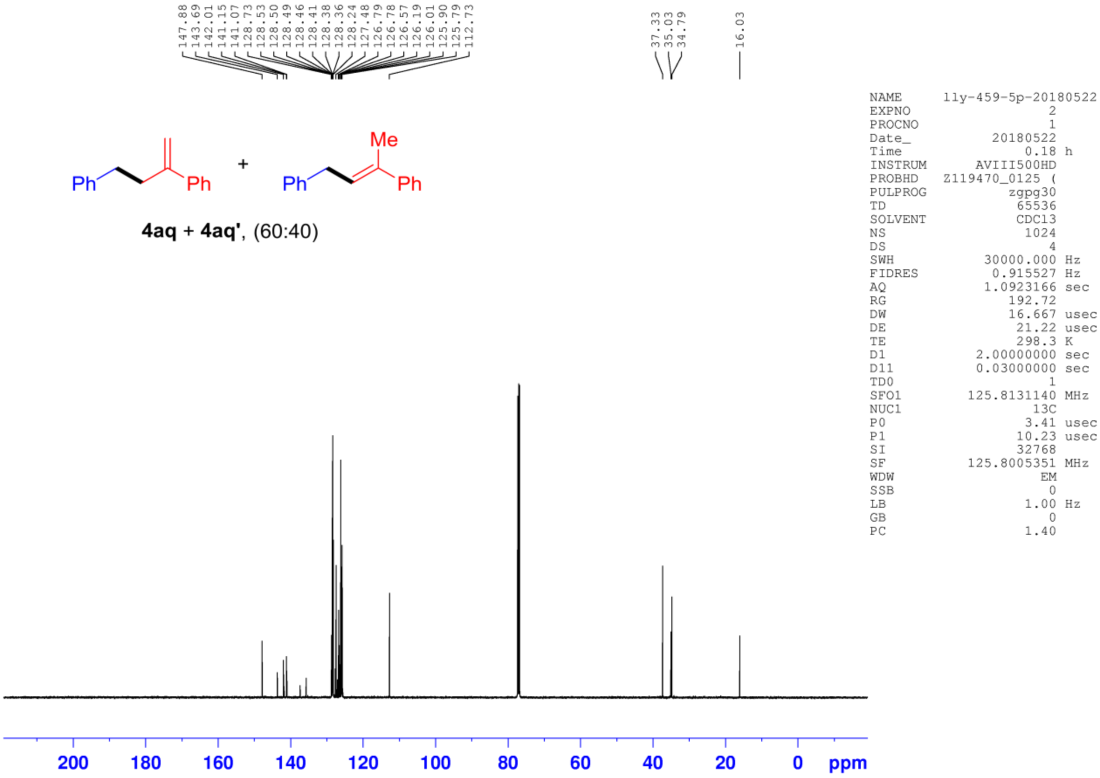

Supplementary Figure 36. ^{13}C NMR spectra for compound **4aq** and **4aq'**

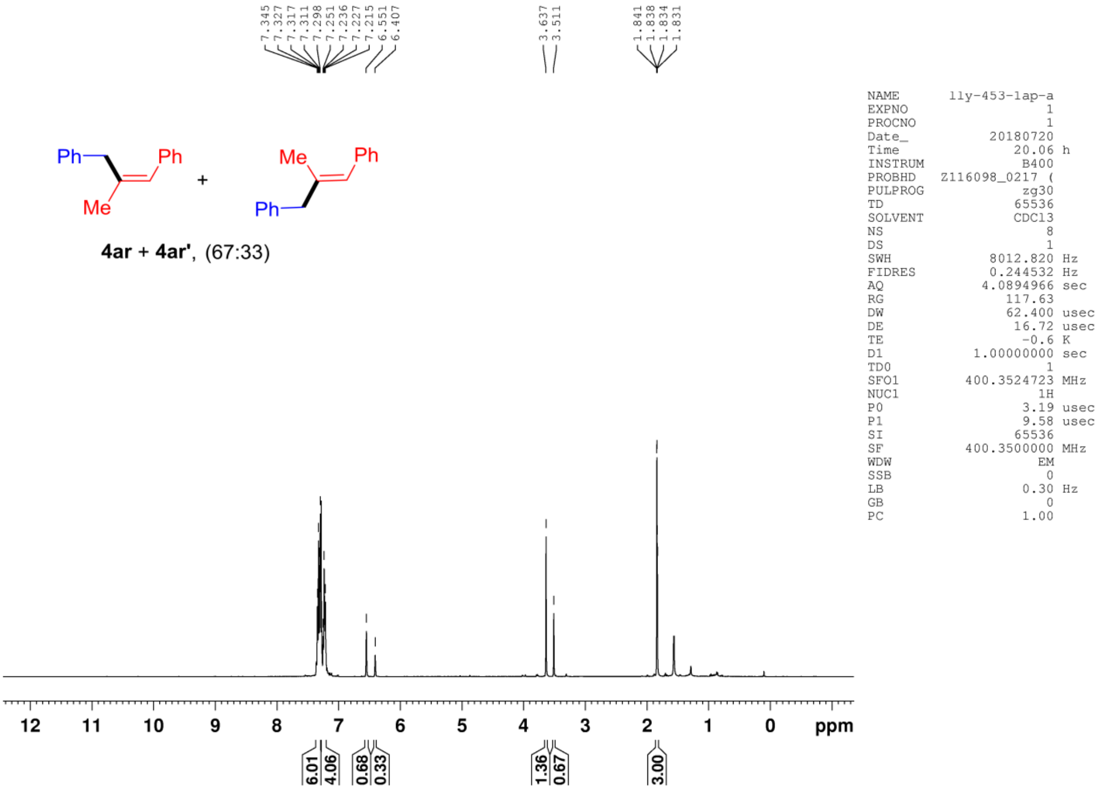

Supplementary Figure 37. ^{1}H NMR spectra for compound **4ar** and **4ar'**

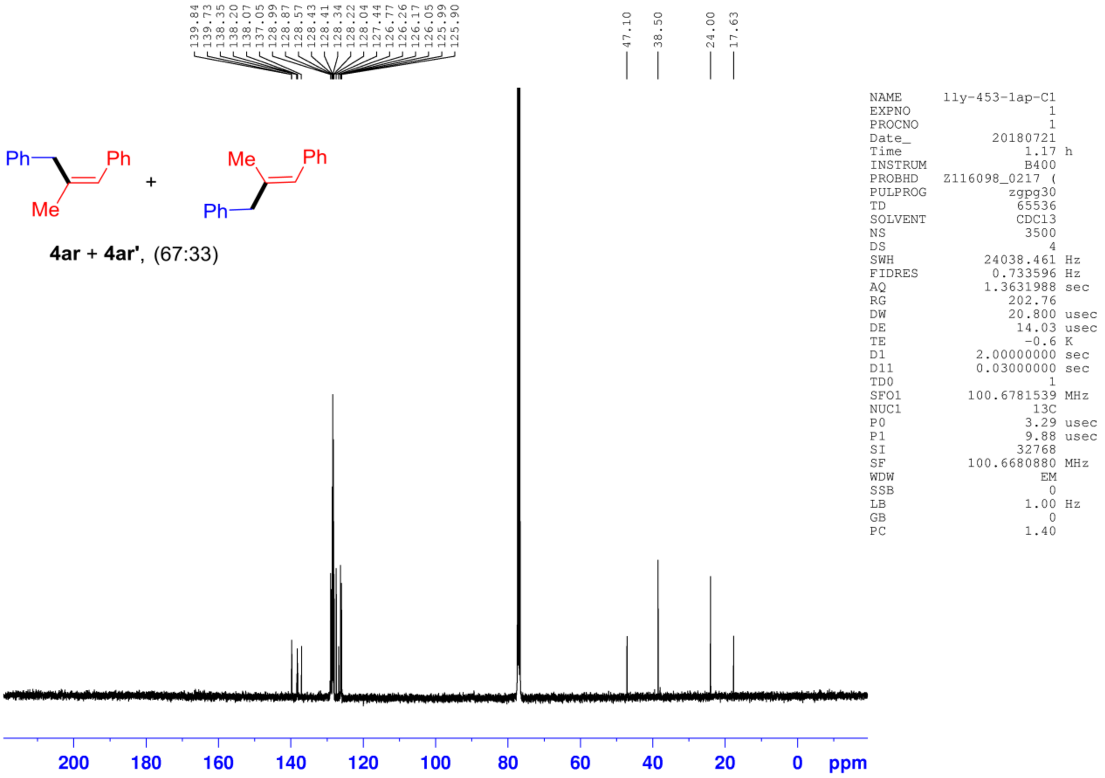

Supplementary Figure 38. ^{13}C NMR spectra for compound **4ar** and **4ar’**

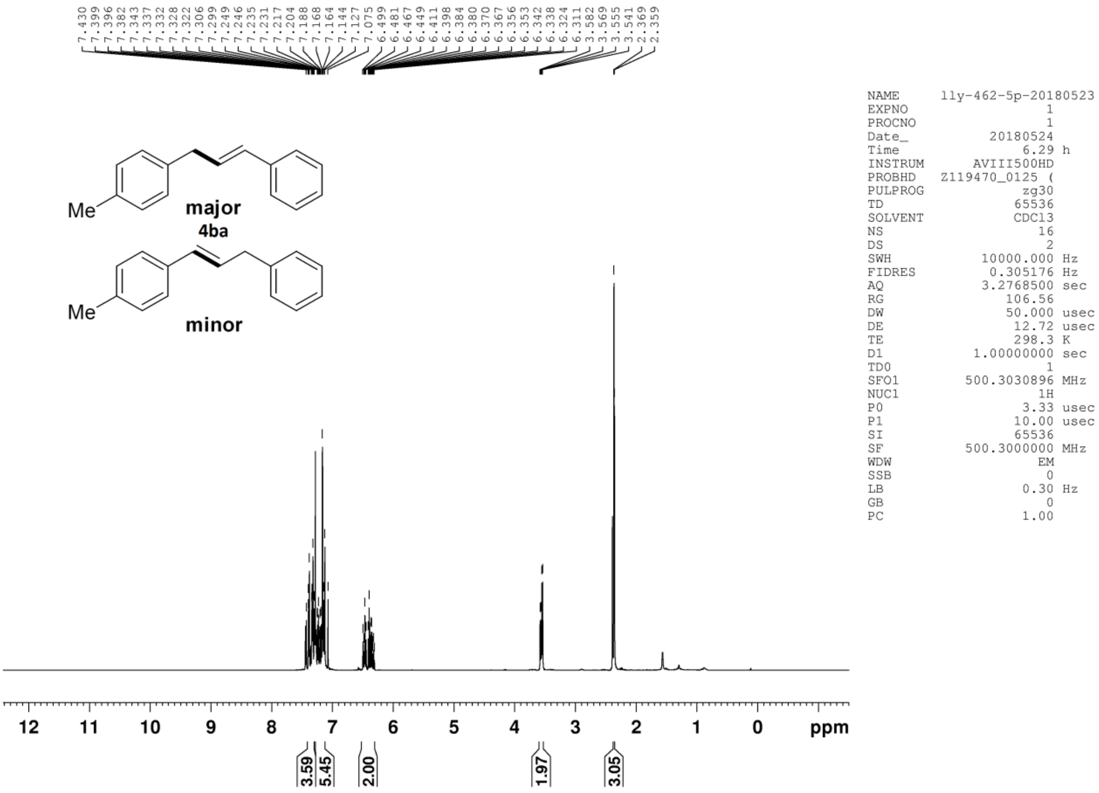

Supplementary Figure 39. ^{1}H NMR spectra for compound **4ba**

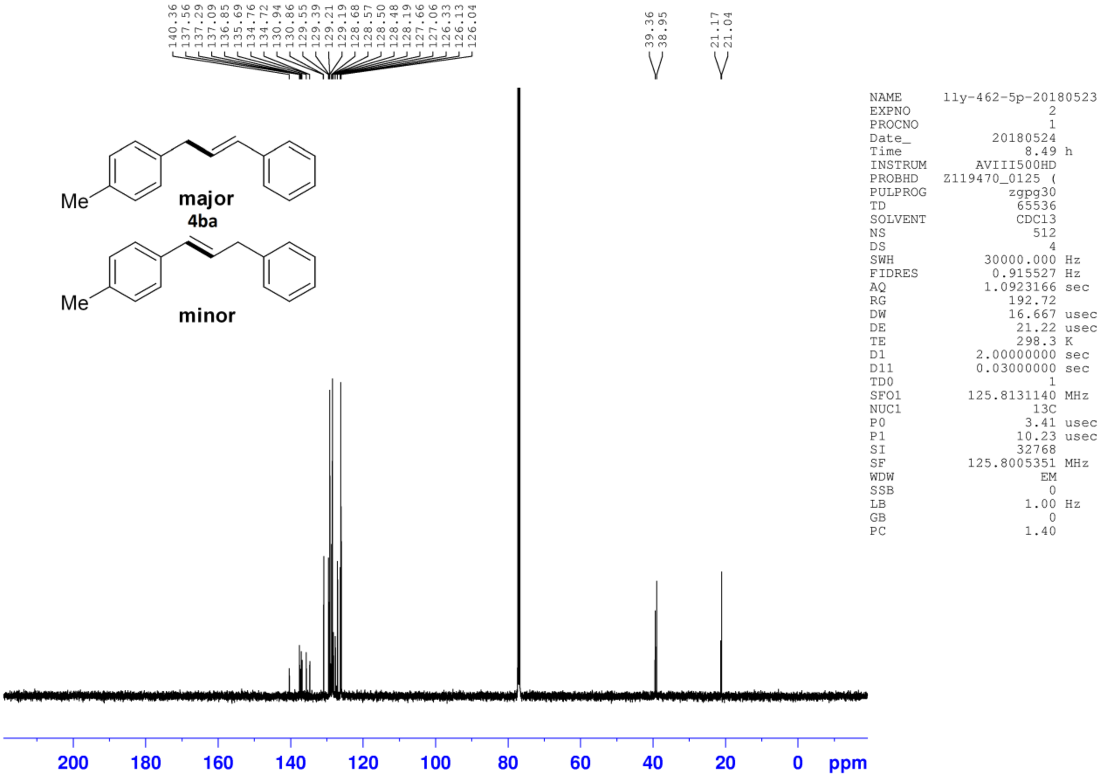

Supplementary Figure 40. ^{13}C NMR spectra for compound **4ba**

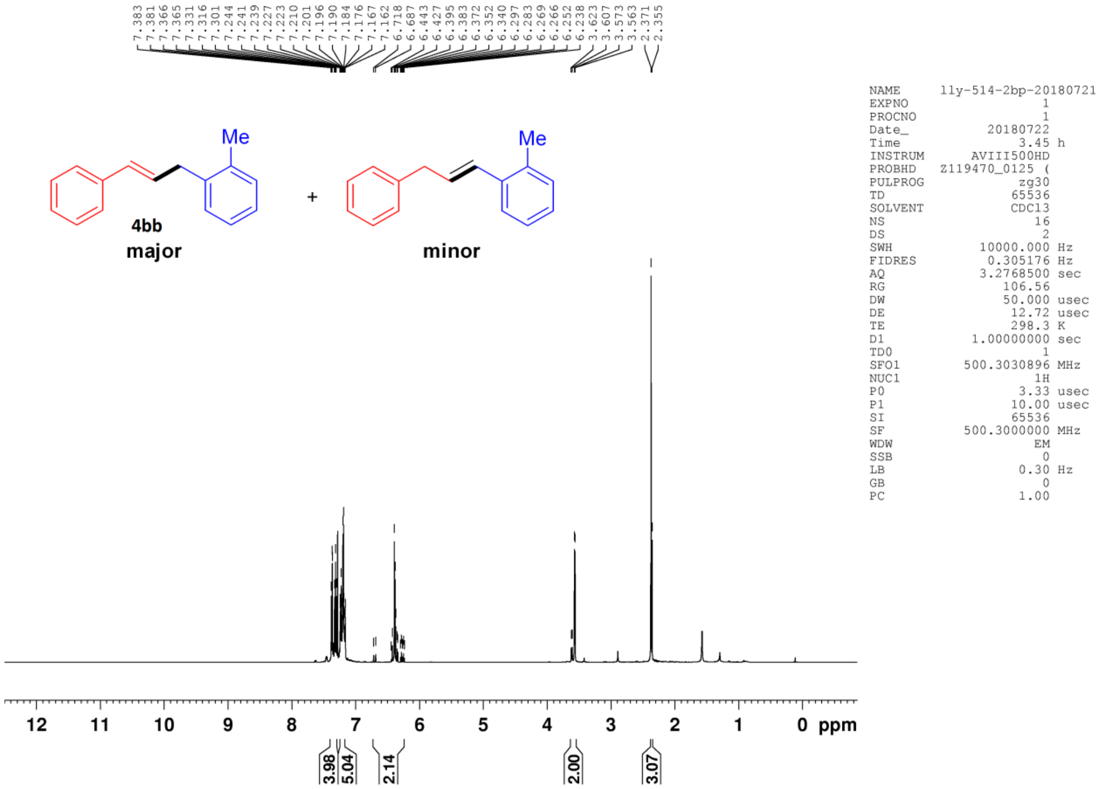

Supplementary Figure 41. ^{1}H NMR spectra for compound **4bb**

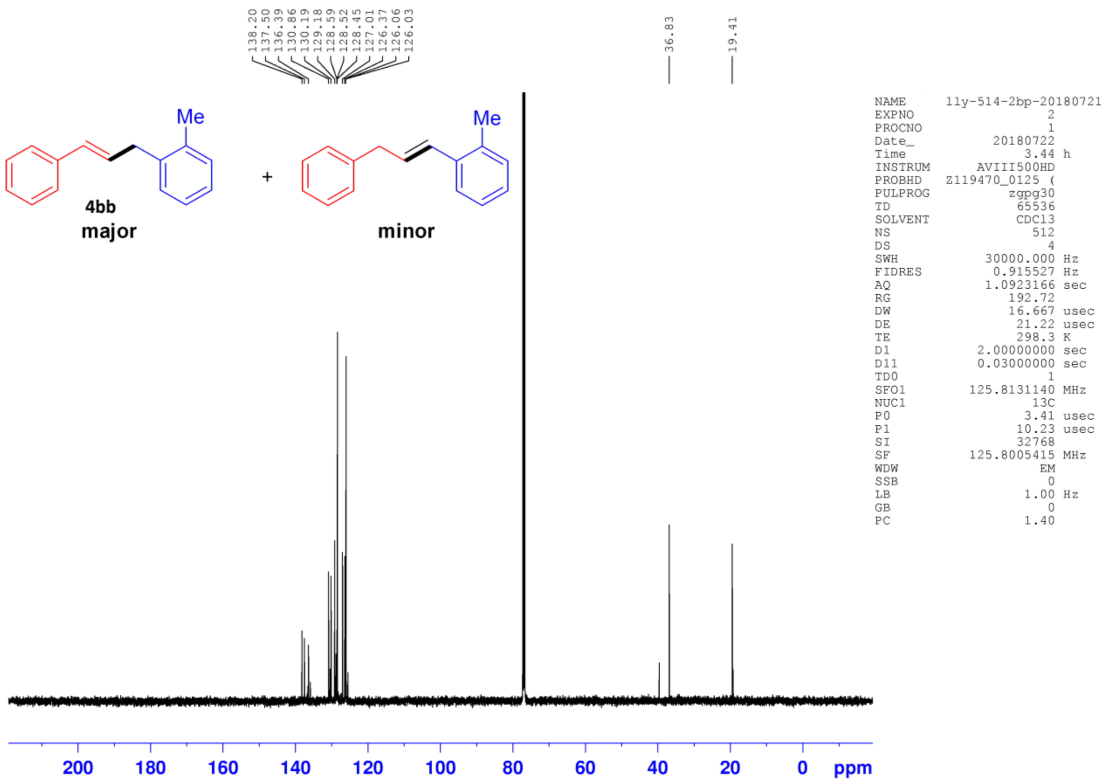

Supplementary Figure 42. ^{13}C NMR spectra for compound **4bb**

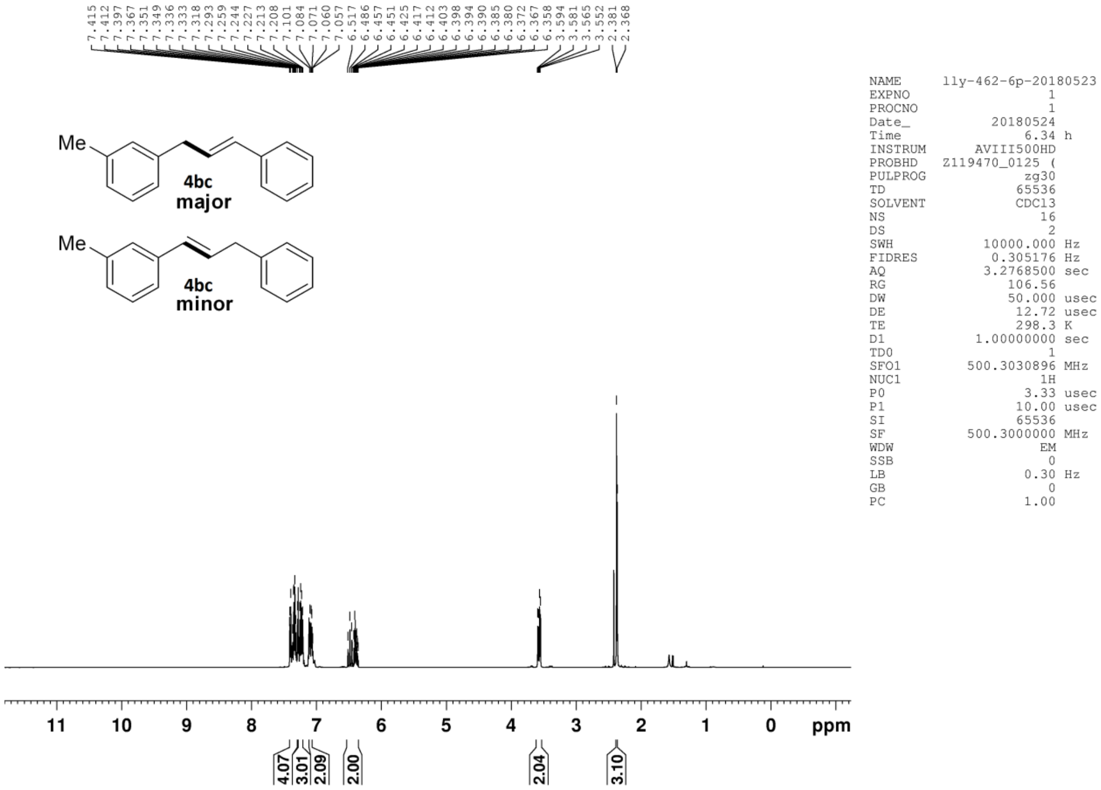

Supplementary Figure 43. ^{1}H NMR spectra for compound **4bc**

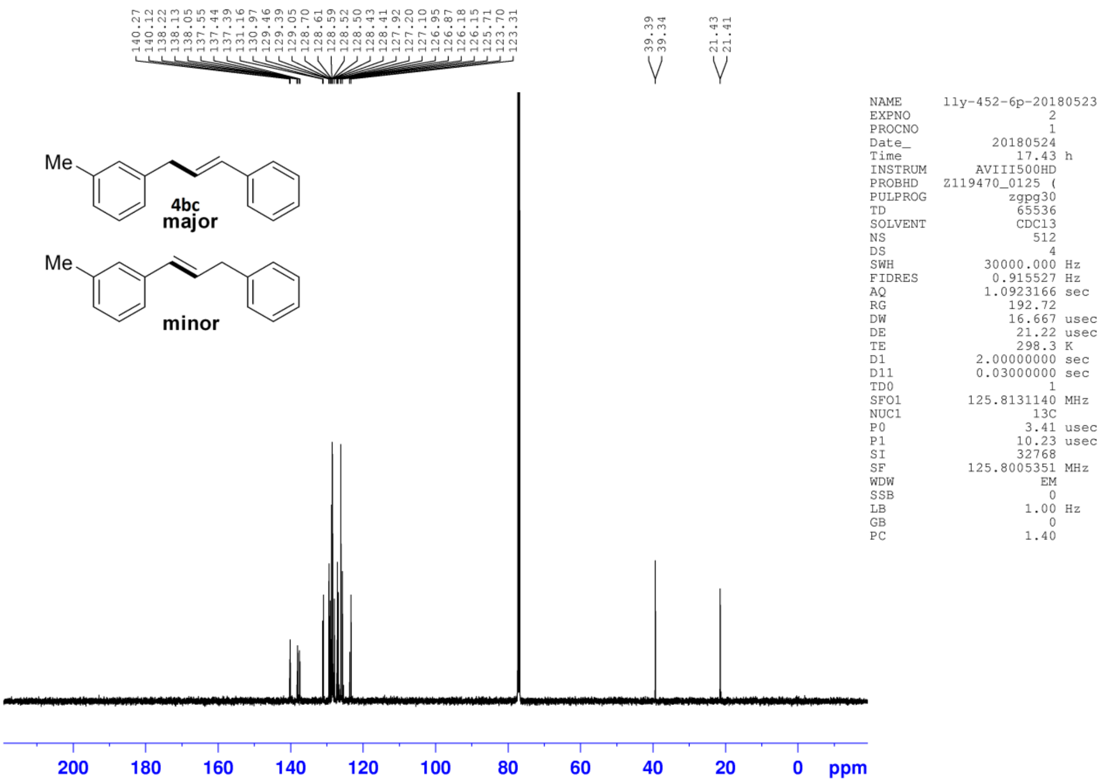

Supplementary Figure 44. ^{13}C NMR spectra for compound **4bc**

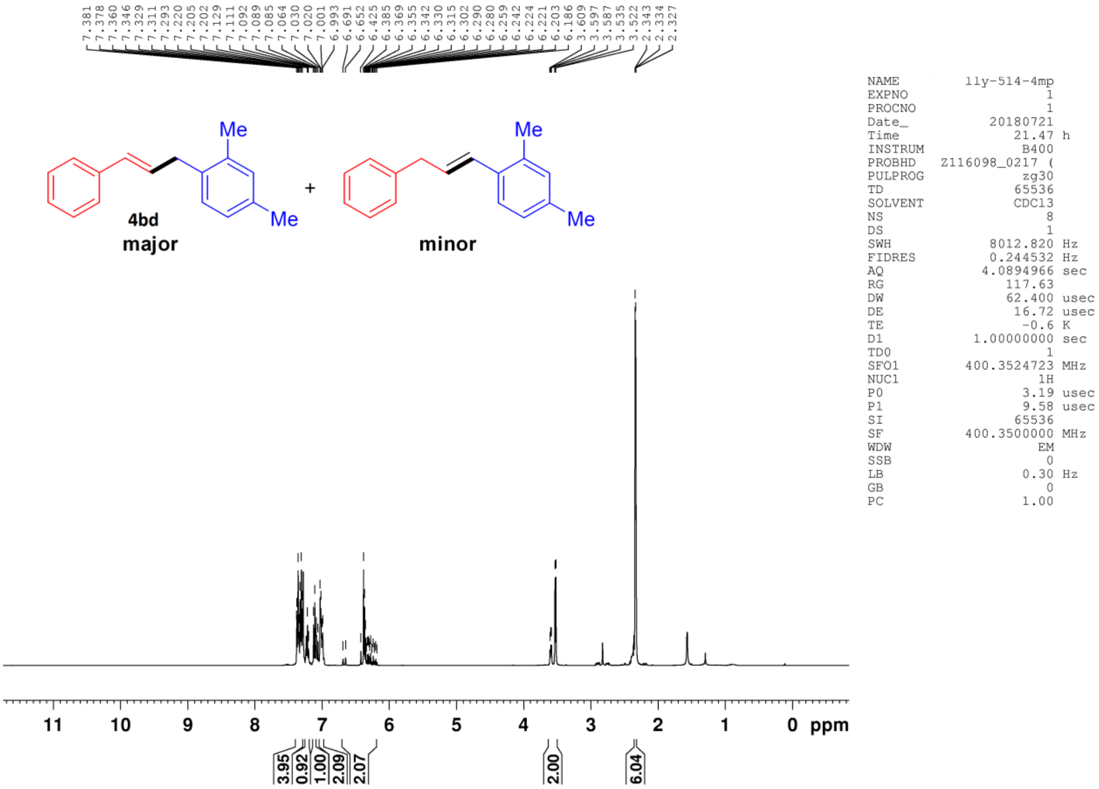

Supplementary Figure 45. ^{1}H NMR spectra for compound **4bd**

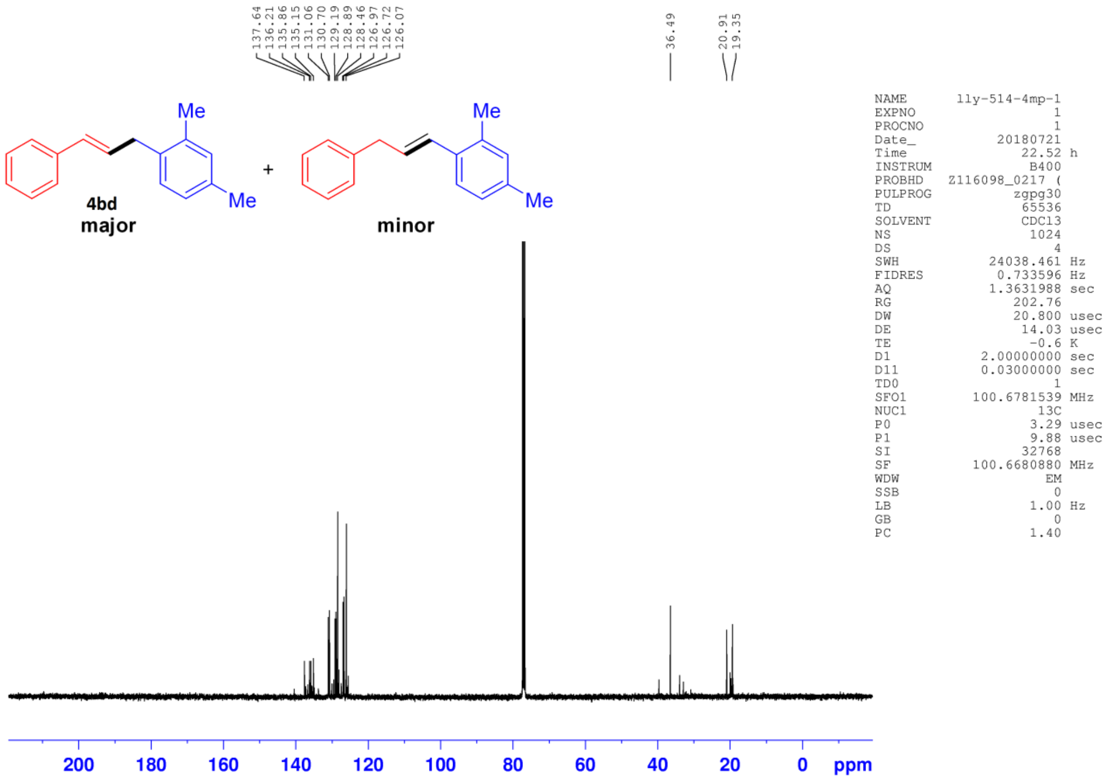

Supplementary Figure 46. ^{13}C NMR spectra for compound **4bd**

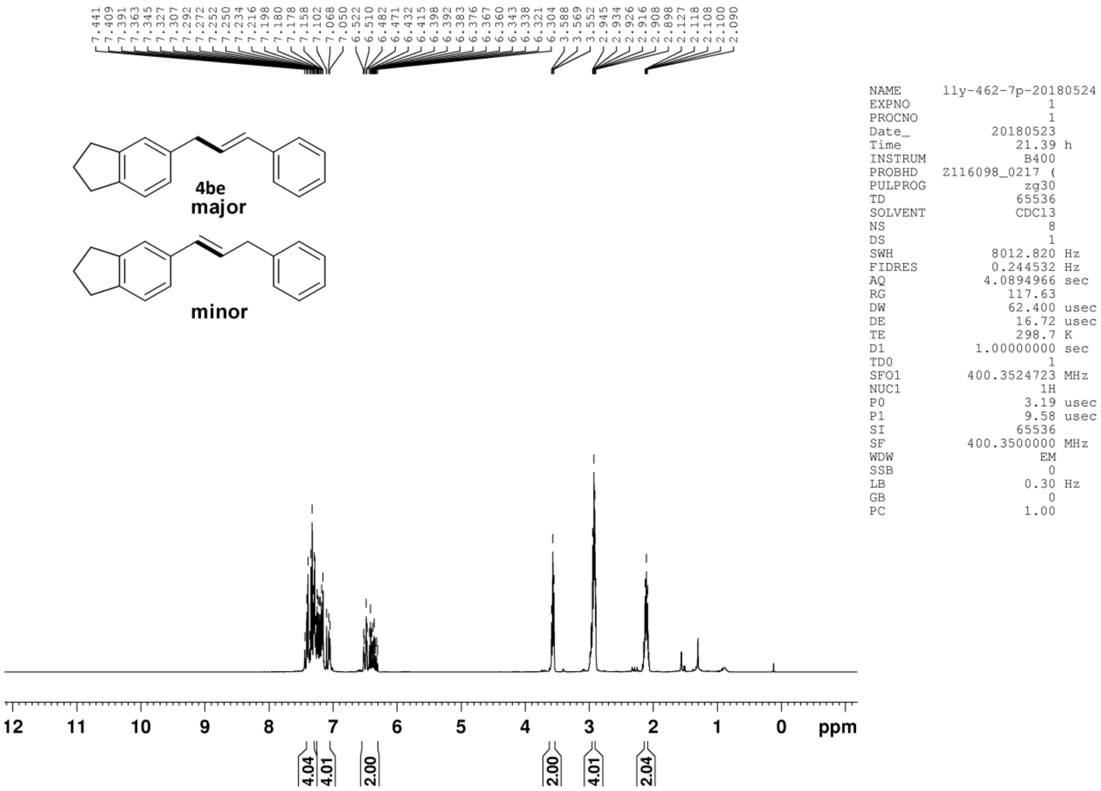

Supplementary Figure 47. ^{1}H NMR spectra for compound **4be**

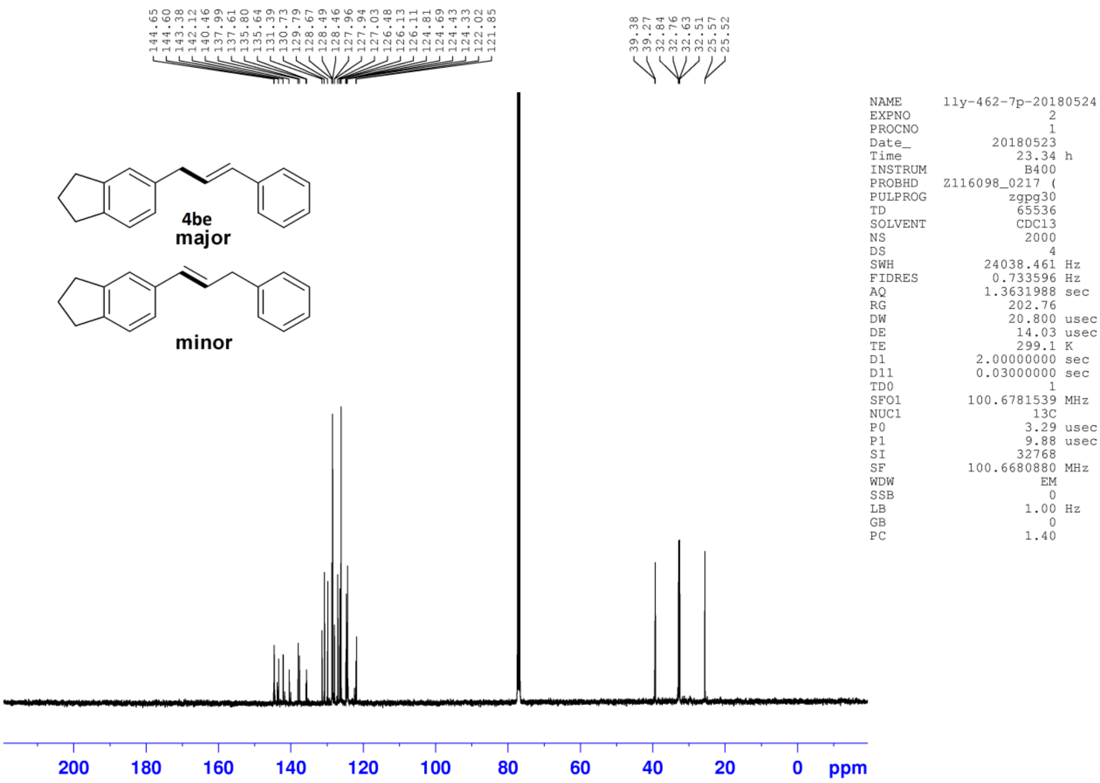

Supplementary Figure 48. ^{13}C NMR spectra for compound **4be**

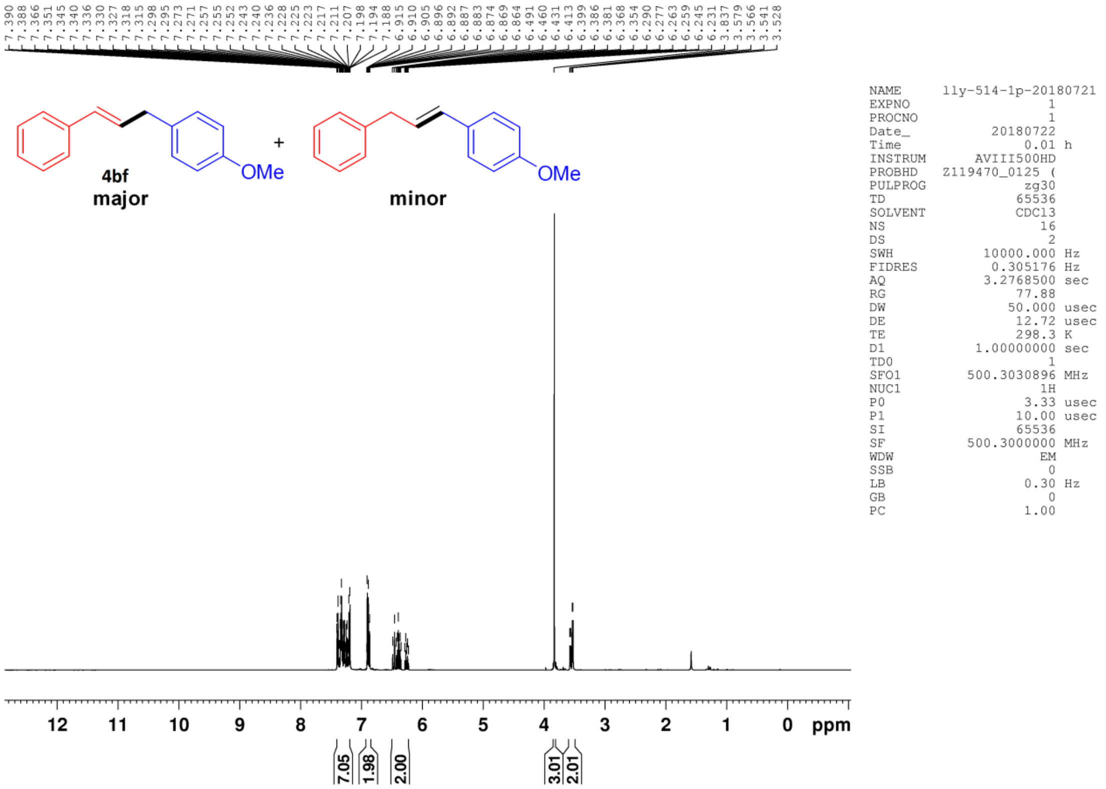

Supplementary Figure 49. ^{1}H NMR spectra for compound **4bf**

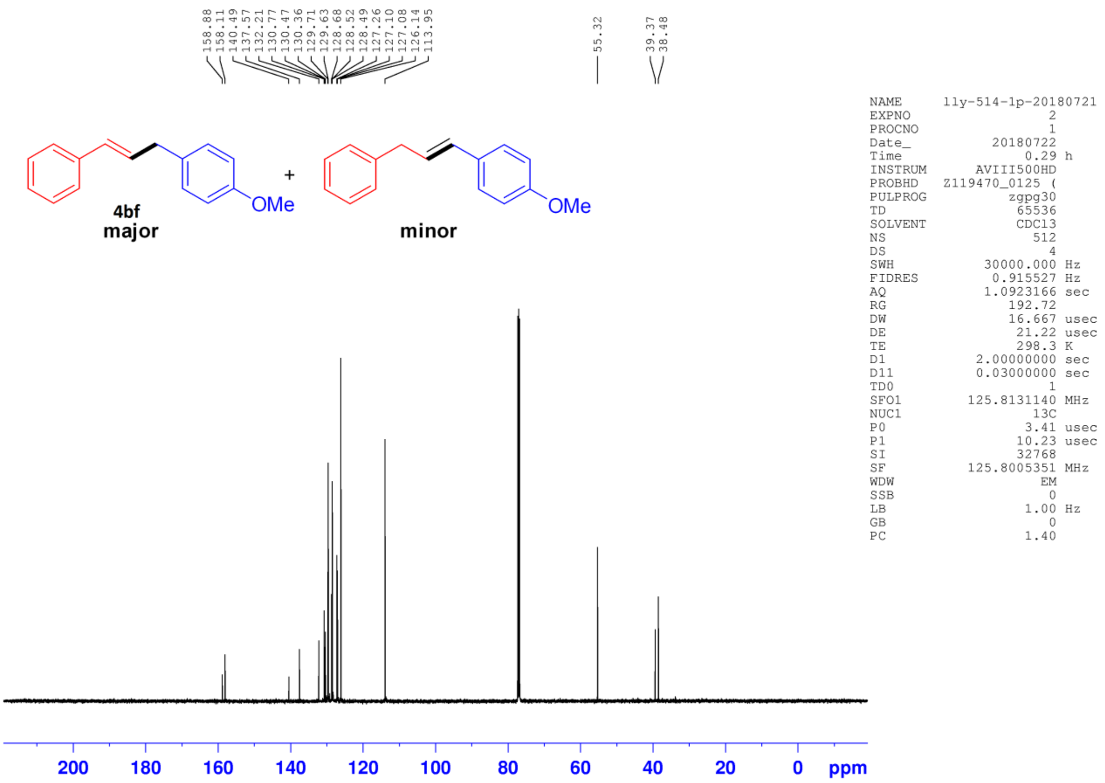

Supplementary Figure 50. ^{13}C NMR spectra for compound **4bf**

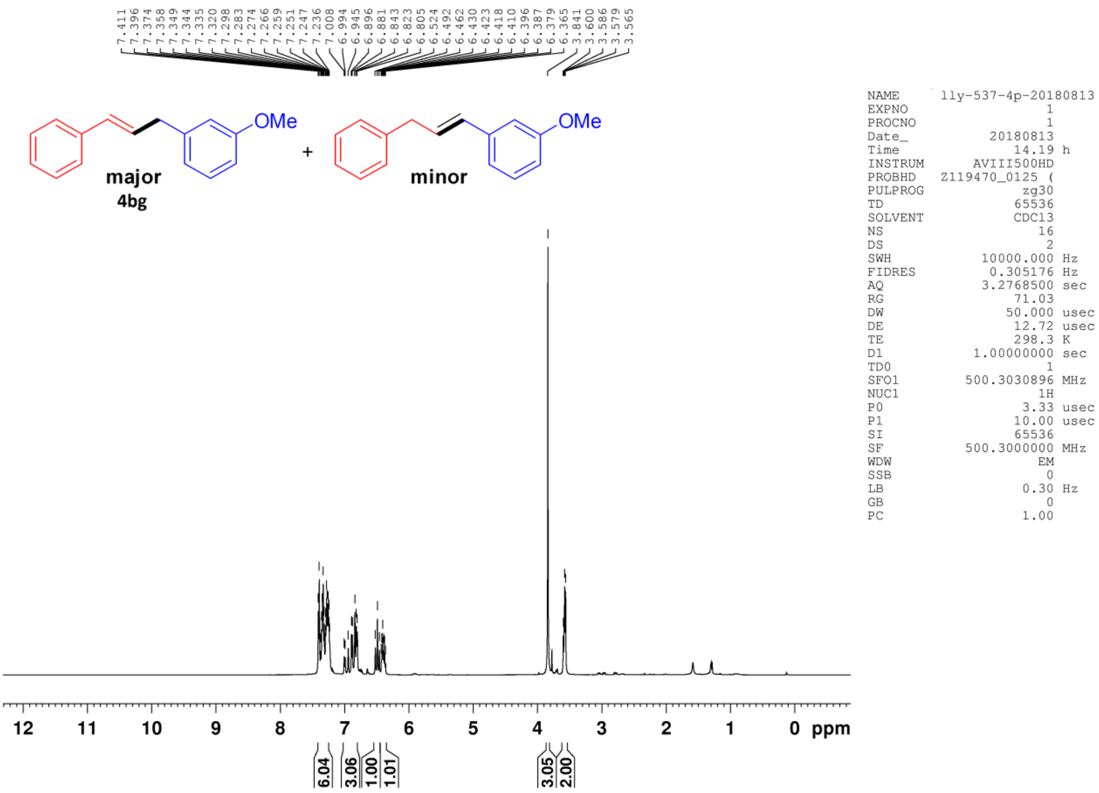

Supplementary Figure 51. ^{1}H NMR spectra for compound **4bg**

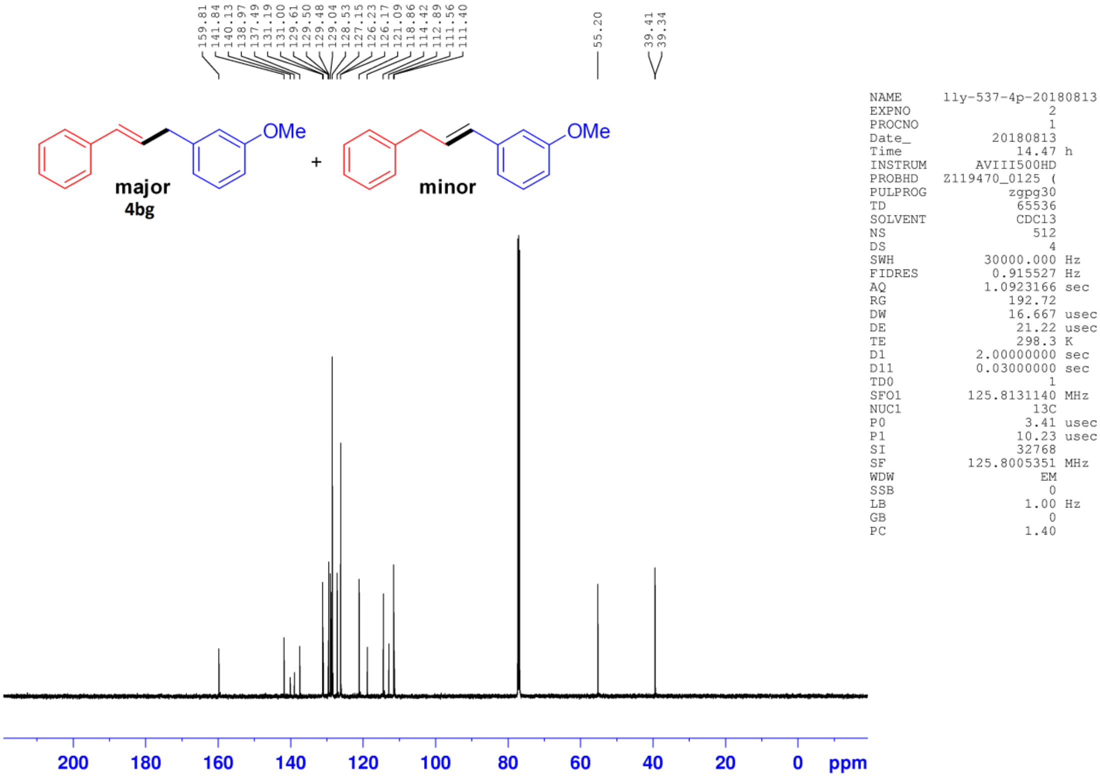

Supplementary Figure 52. ^{13}C NMR spectra for compound **4bg**

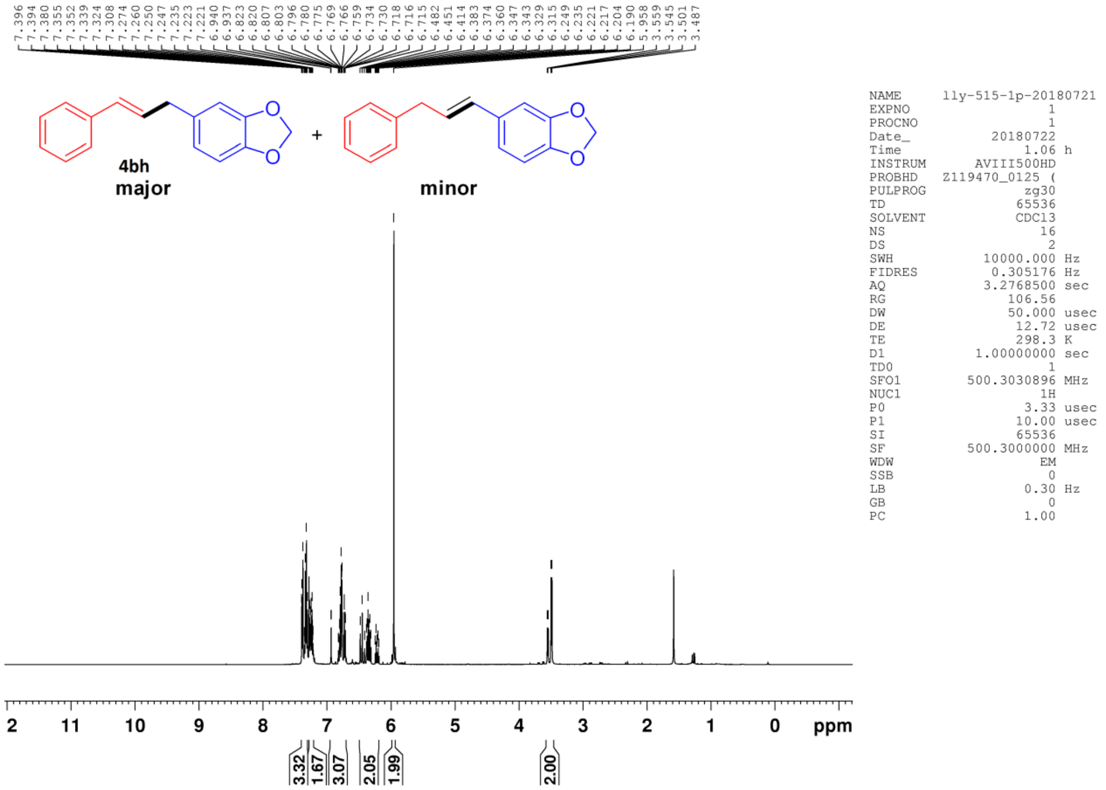

Supplementary Figure 53. ^{1}H NMR spectra for compound **4bh**

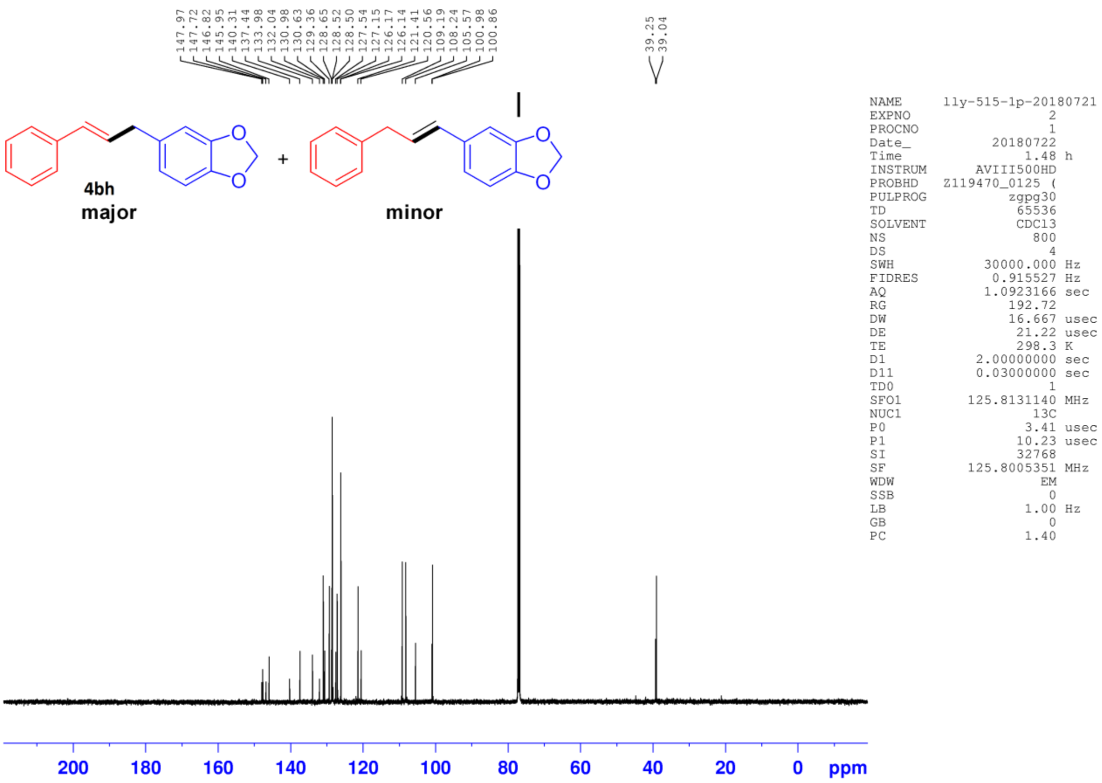

Supplementary Figure 54. ^{13}C NMR spectra for compound **4bh**

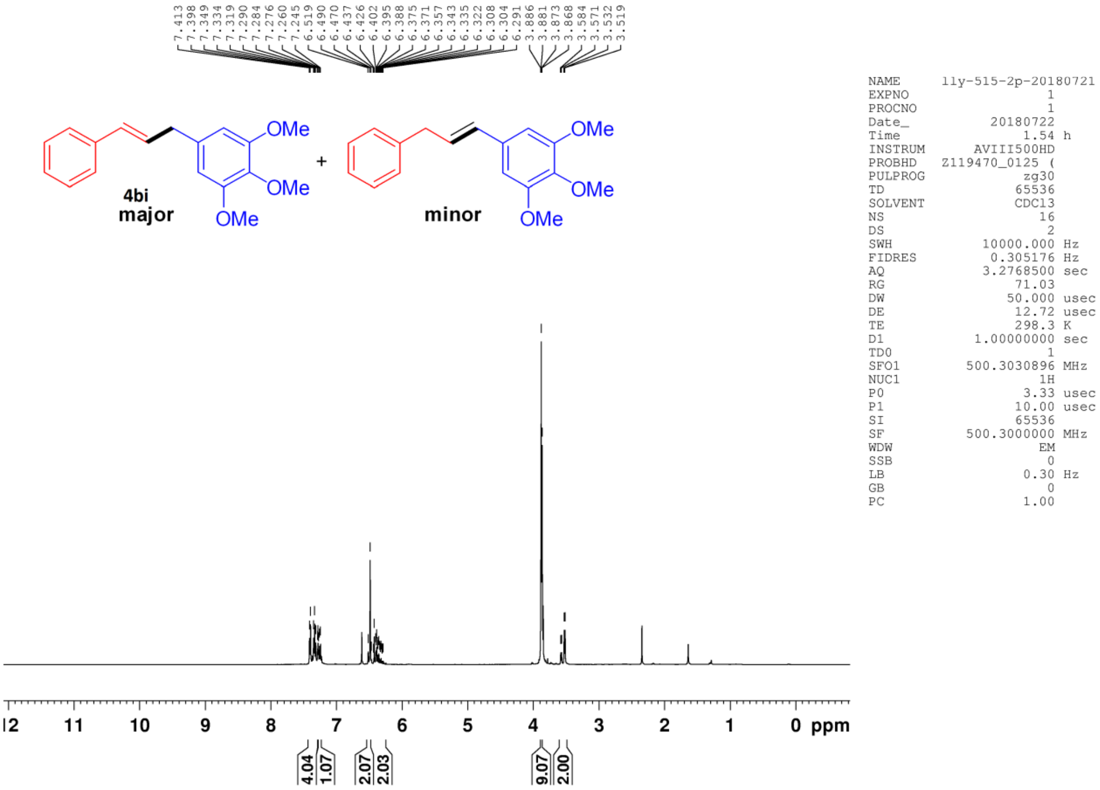

Supplementary Figure 55. ^{1}H NMR spectra for compound **4bi**

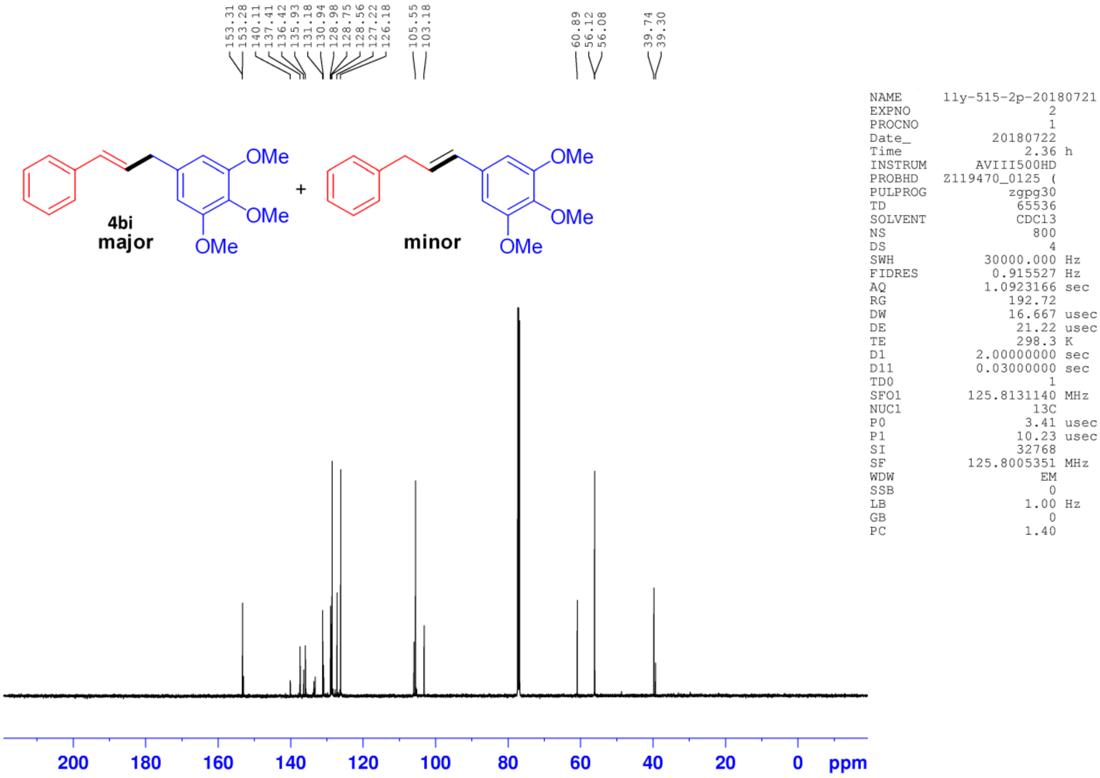

Supplementary Figure 56. ^{13}C NMR spectra for compound **4bi**

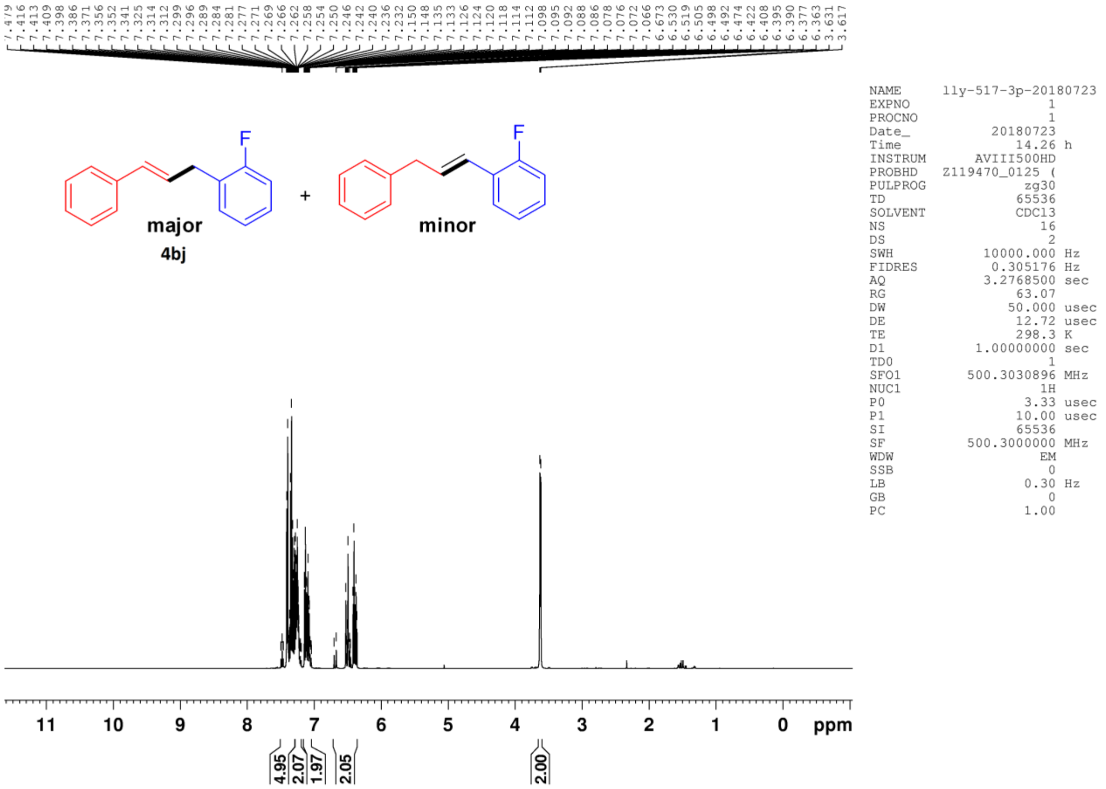

Supplementary Figure 57. ^{1}H NMR spectra for compound **4bj**

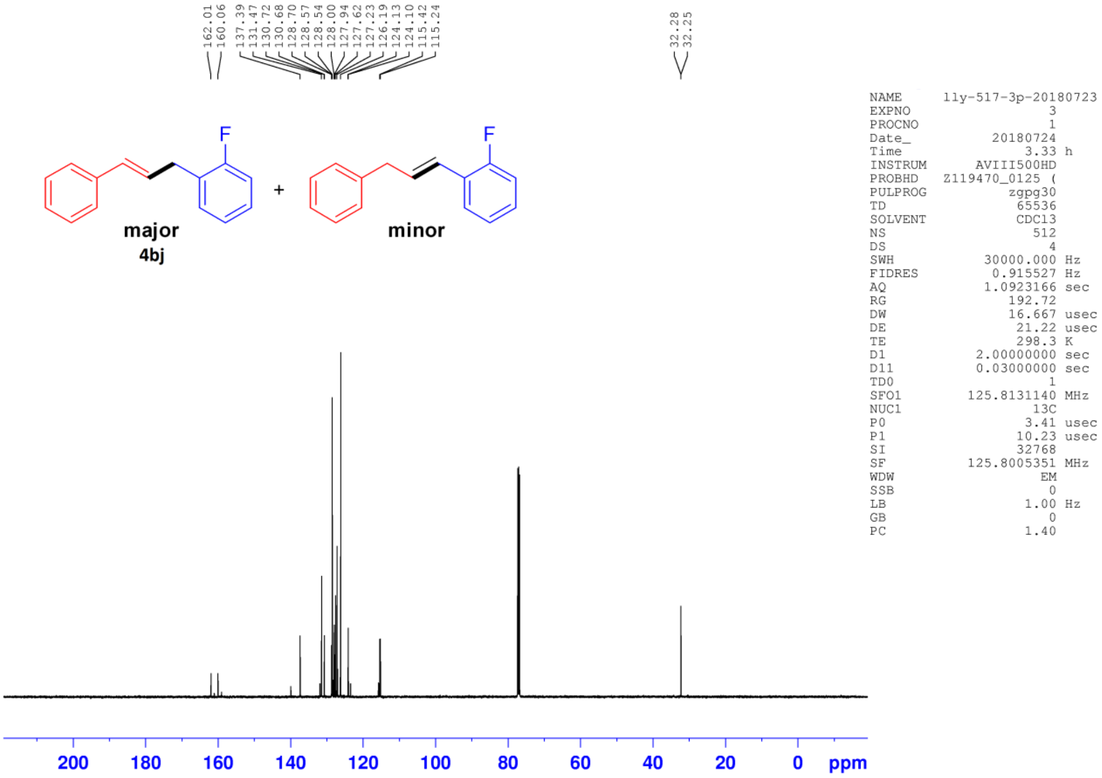

Supplementary Figure 58. ^{13}C NMR spectra for compound **4bj**

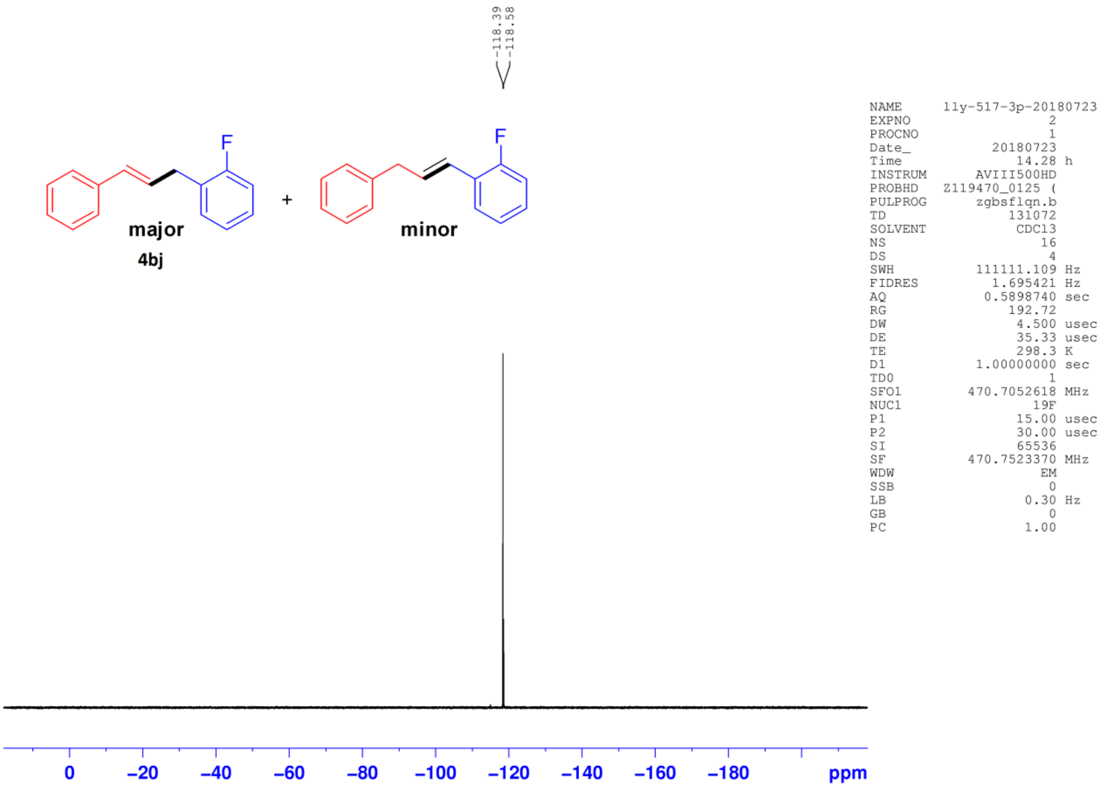

Supplementary Figure 59. ^{19}F NMR spectra for compound **4bj**

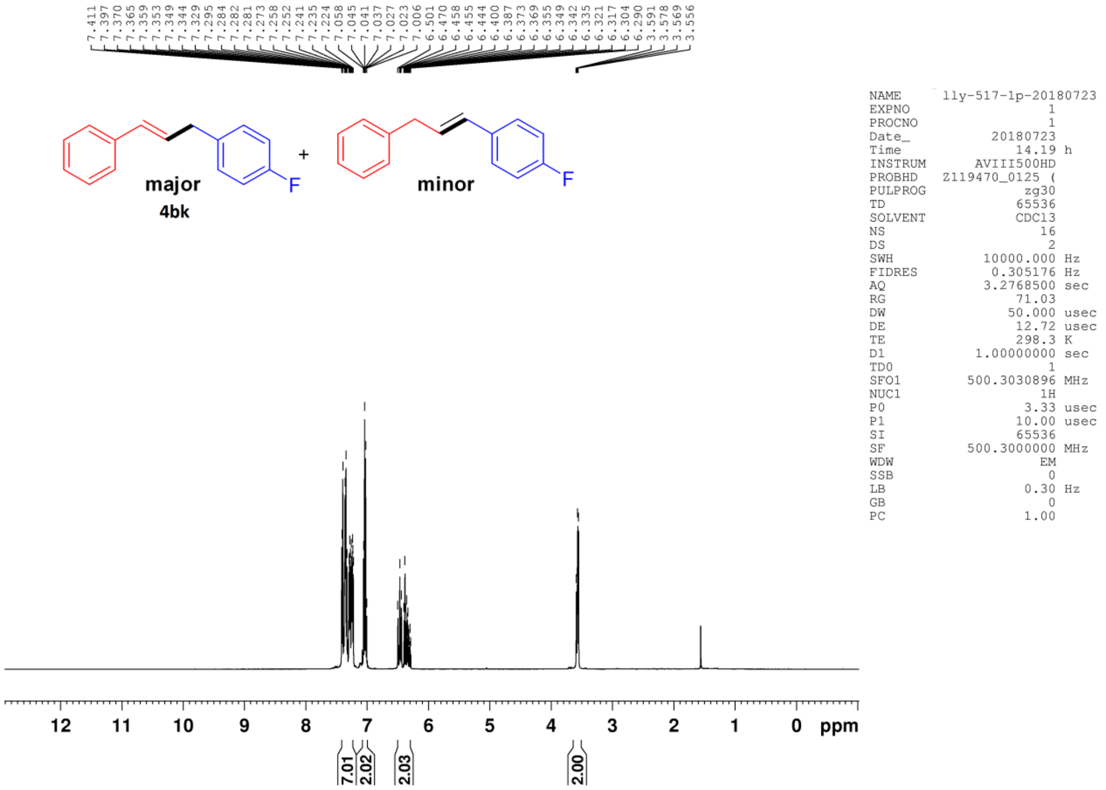

Supplementary Figure 60. ^{1}H NMR spectra for compound **4bk**

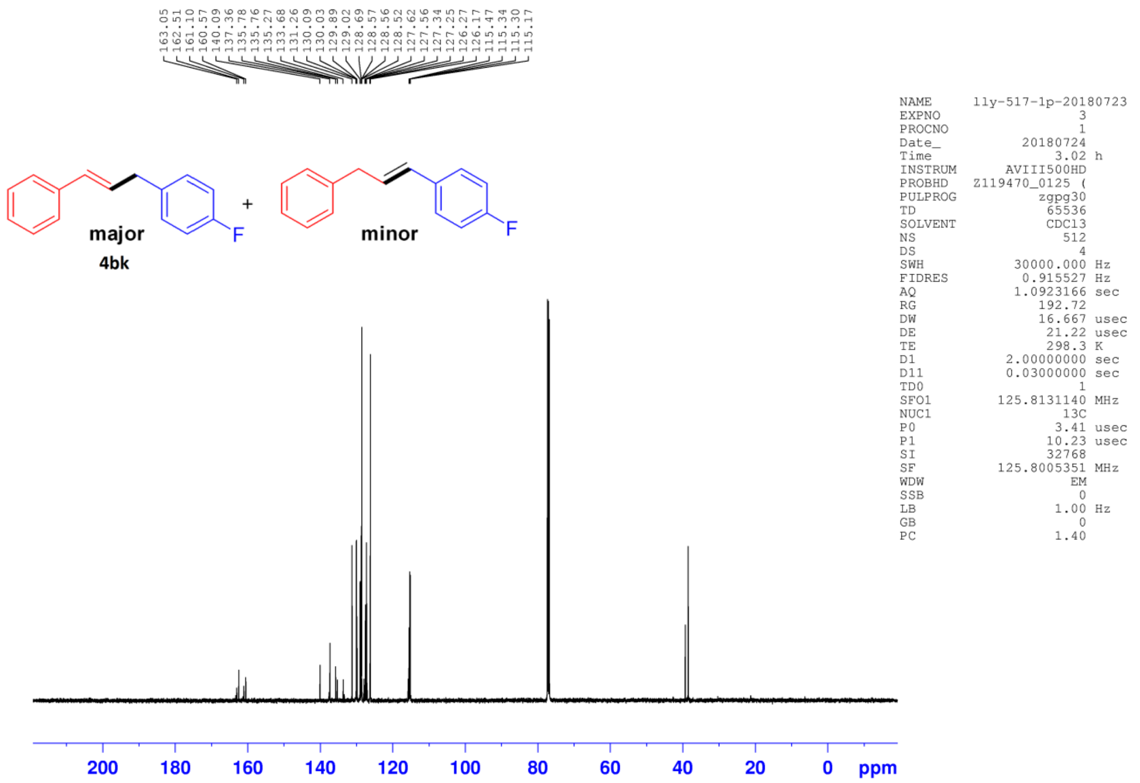

Supplementary Figure 61. ^{13}C NMR spectra for compound **4bk**

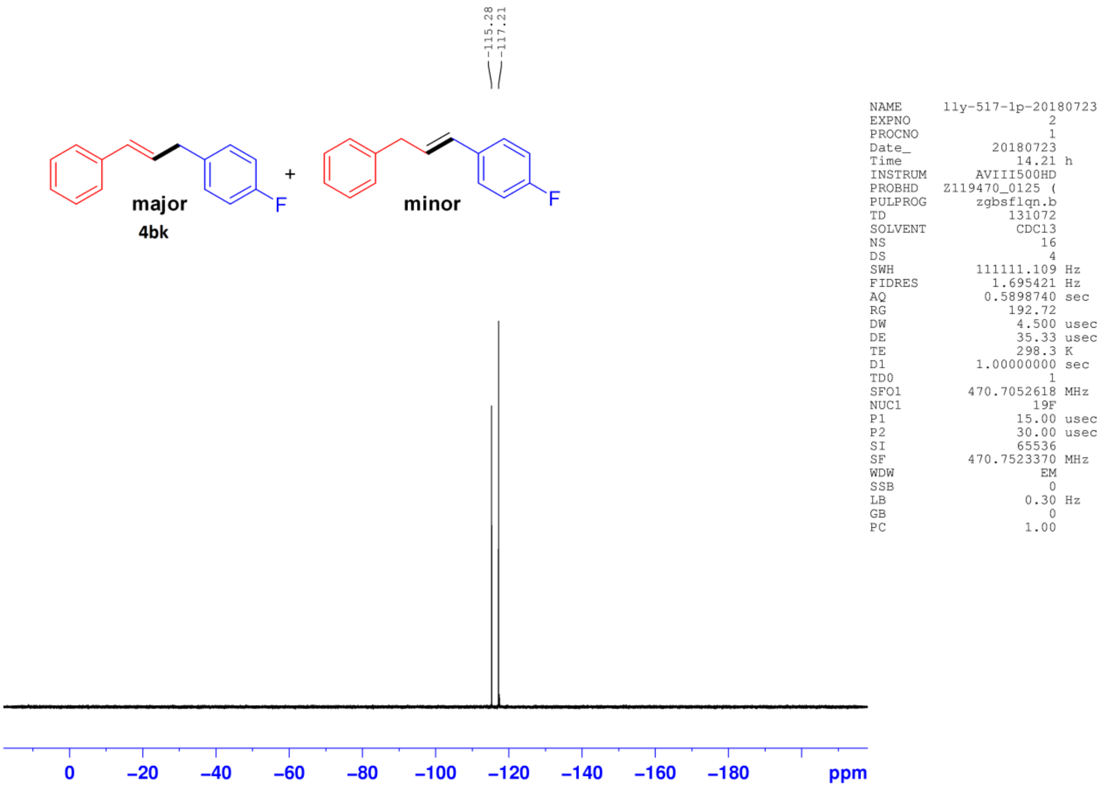

Supplementary Figure 62. ^{19}F NMR spectra for compound **4bk**

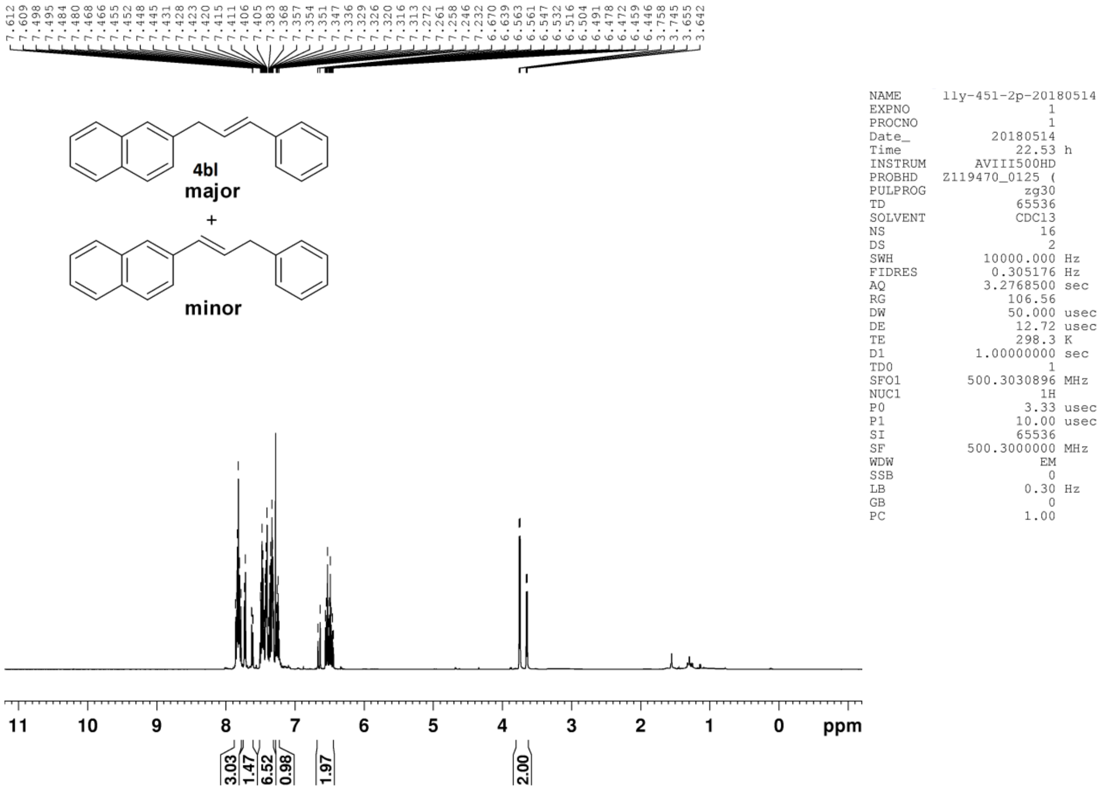

Supplementary Figure 63. ^{1}H NMR spectra for compound **4bl**

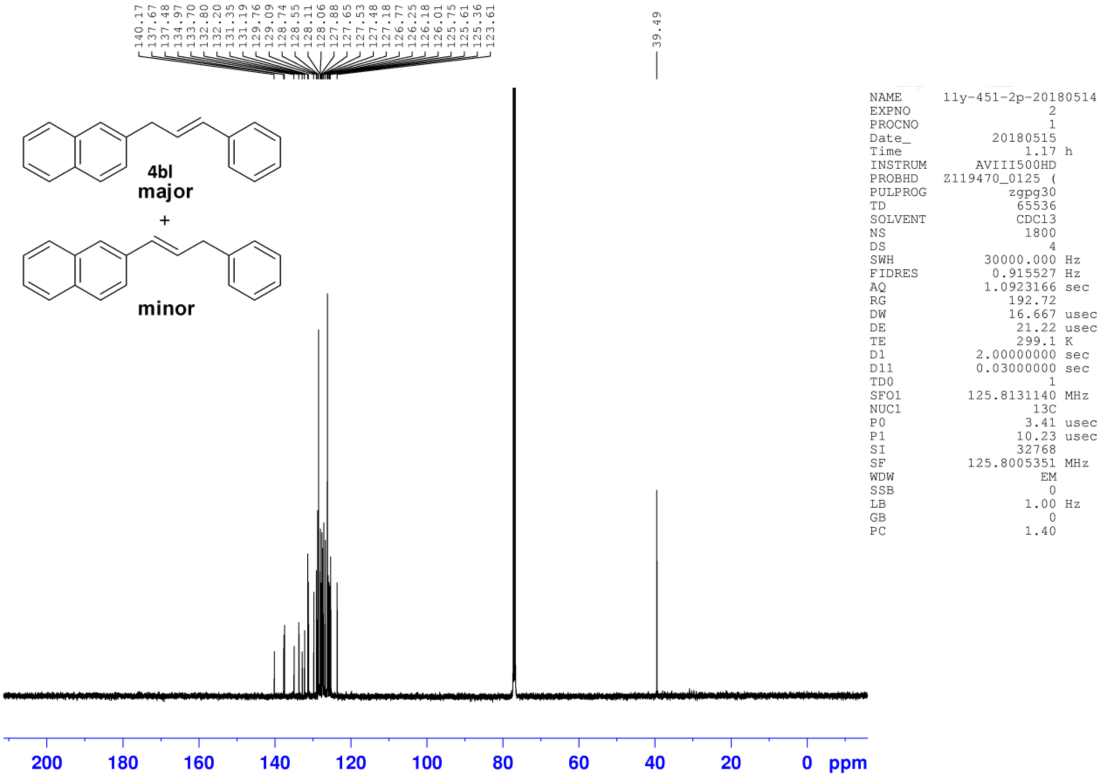

Supplementary Figure 64. ^{13}C NMR spectra for compound **4bl**

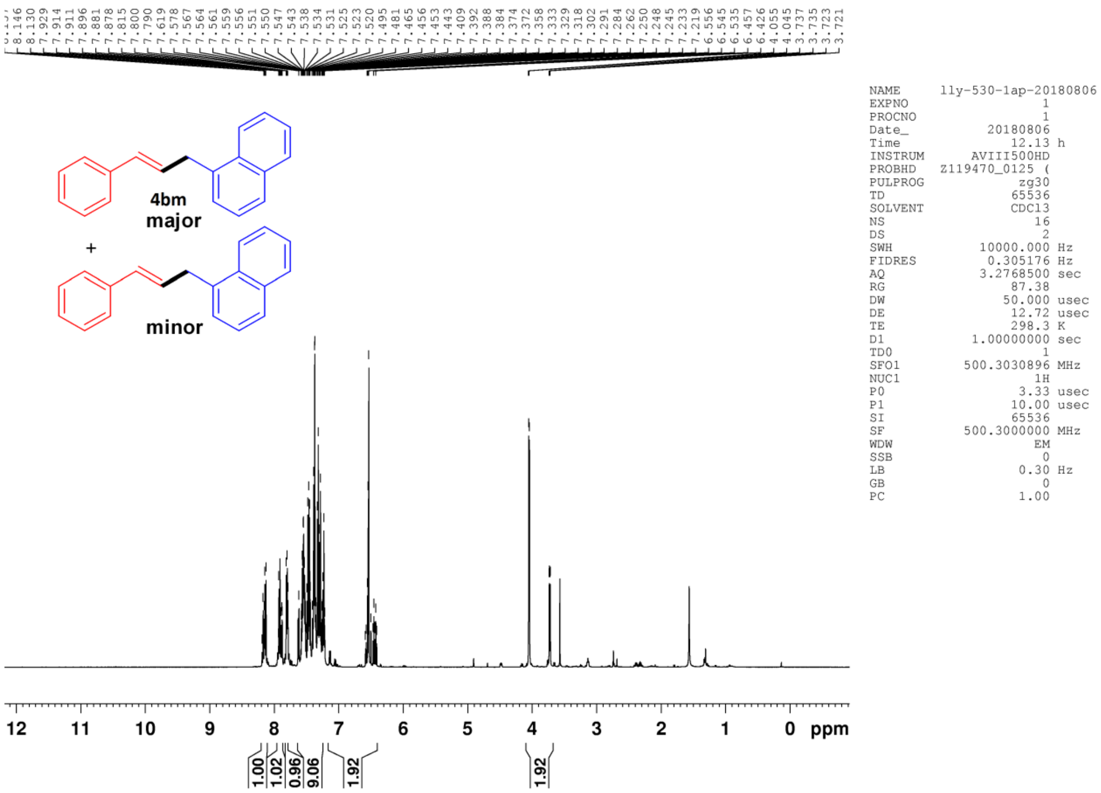

Supplementary Figure 65. ^{1}H NMR spectra for compound **4bm**

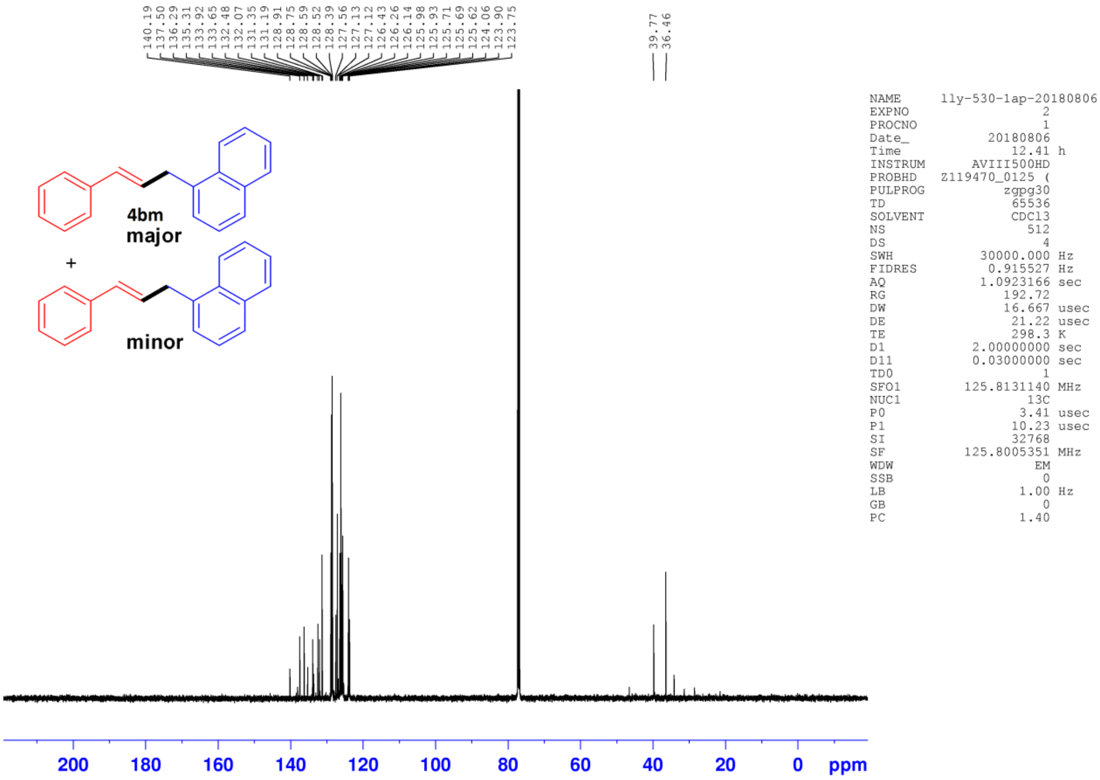

Supplementary Figure 66. ^{13}C NMR spectra for compound **4bm**

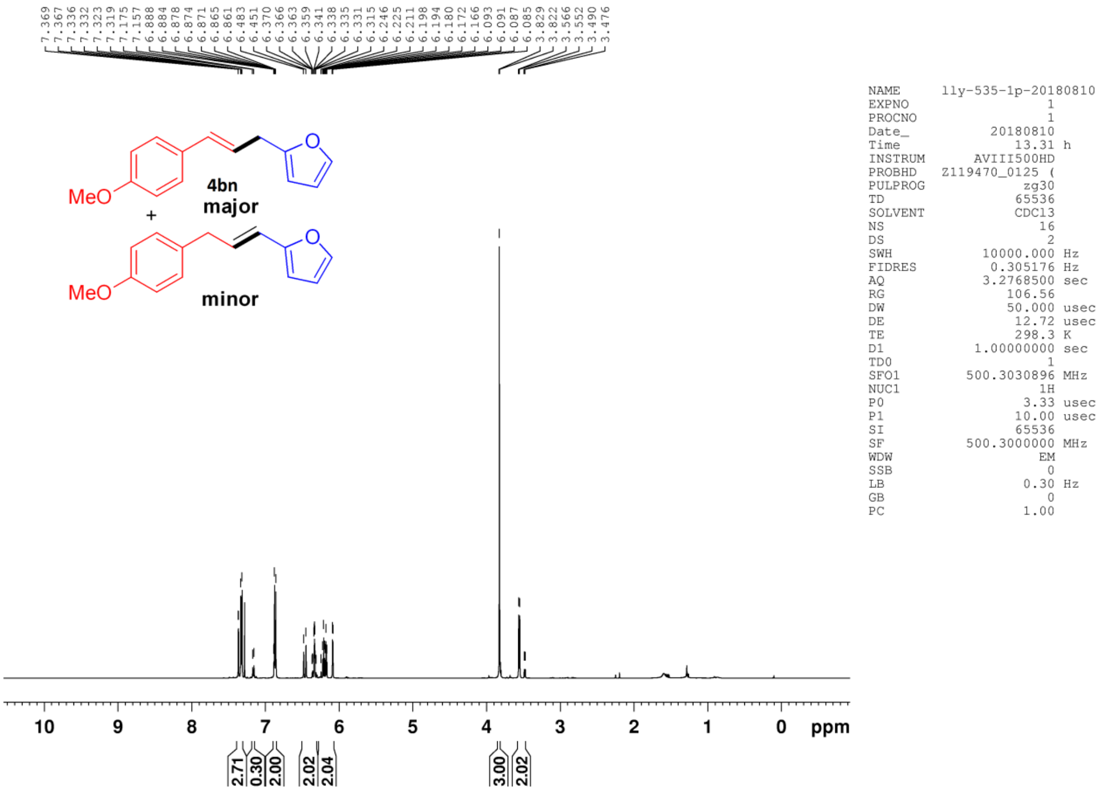

Supplementary Figure 67. ^{1}H NMR spectra for compound **4bn**

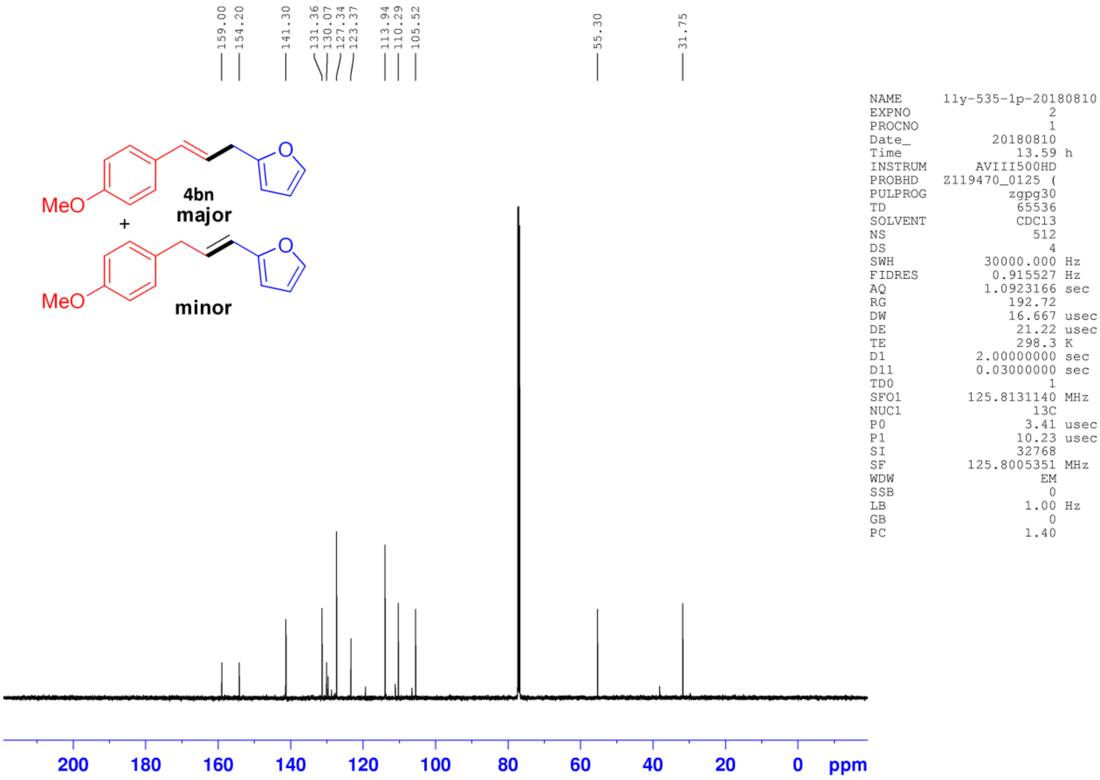

Supplementary Figure 68. ^{13}C NMR spectra for compound **4bn**

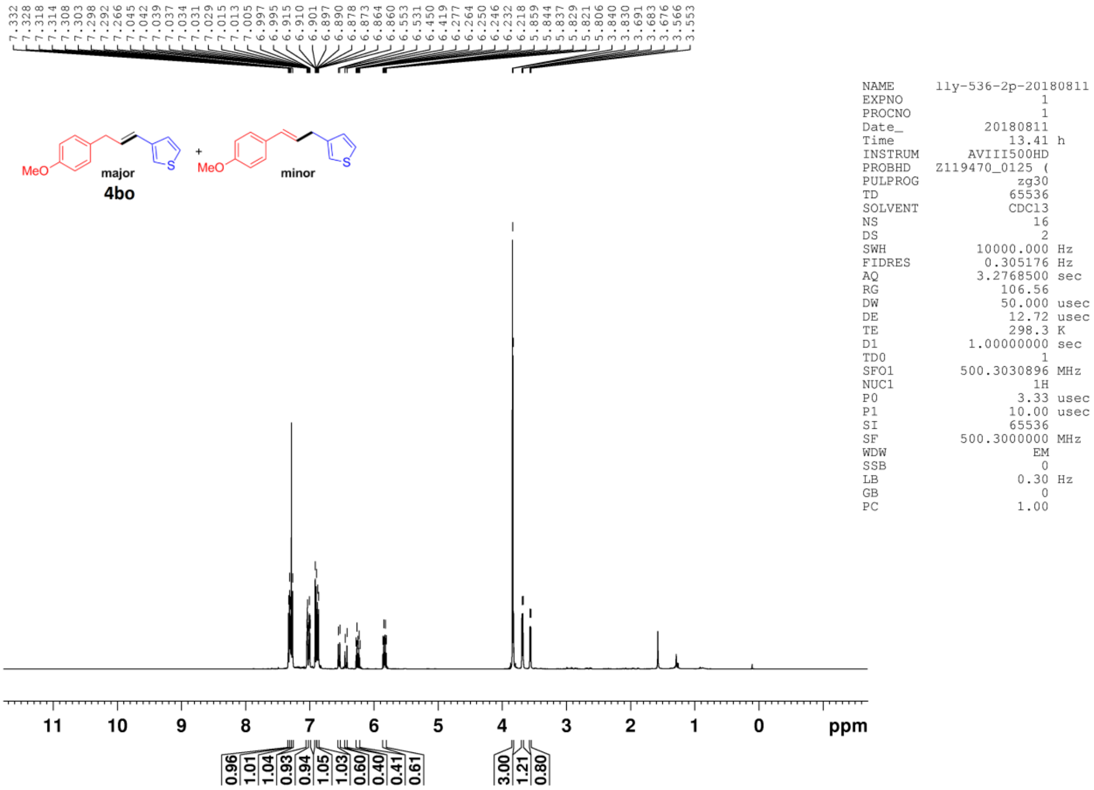

Supplementary Figure 69. ^{1}H NMR spectra for compound **4bo**

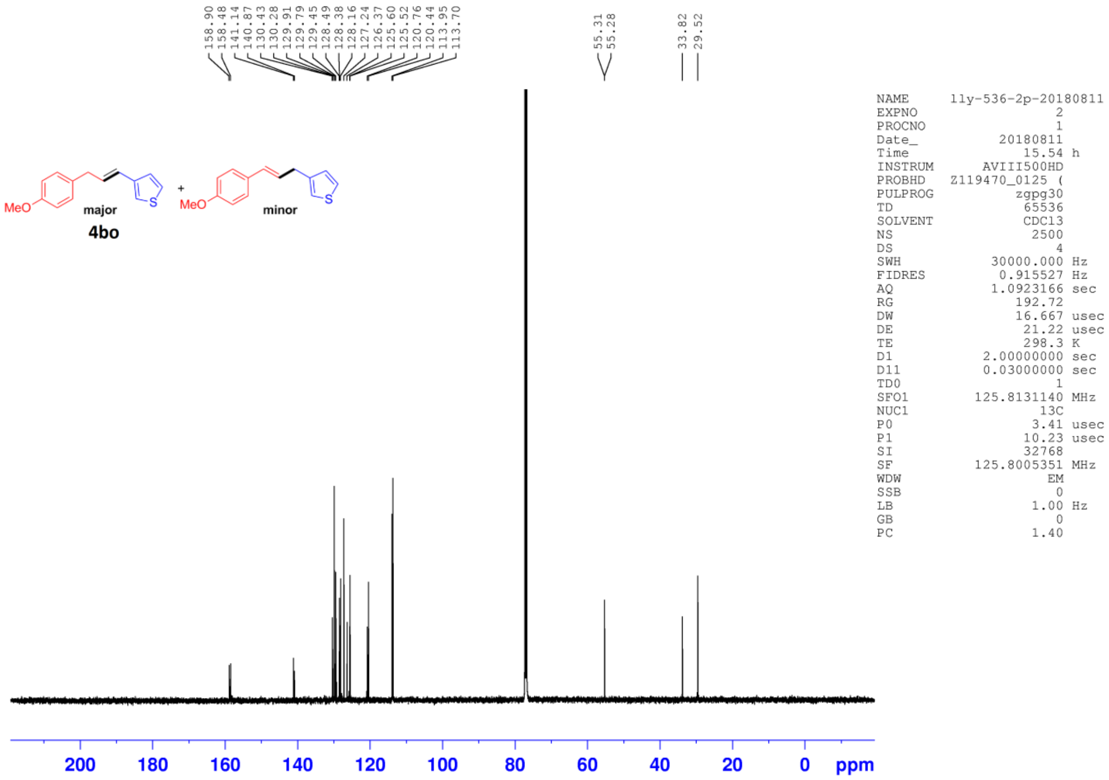

Supplementary Figure 70. ^{13}C NMR spectra for compound **4bo**

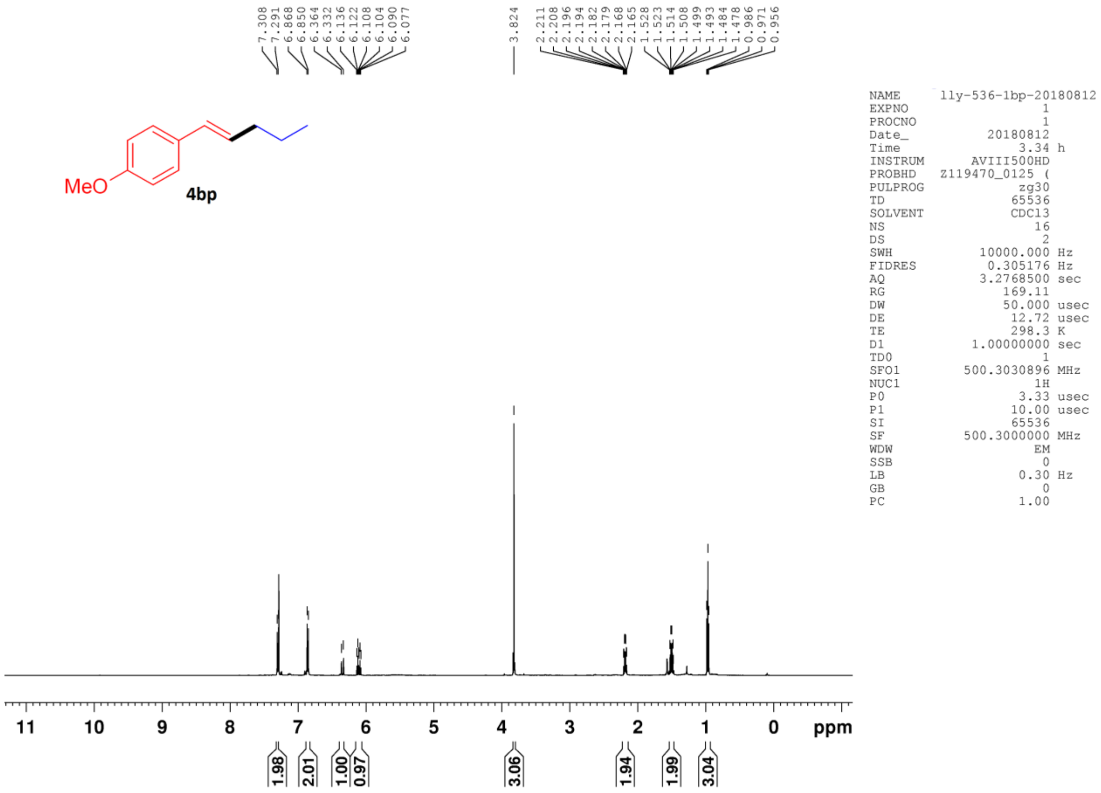

Supplementary Figure 71. ^{1}H NMR spectra for compound **4bp**

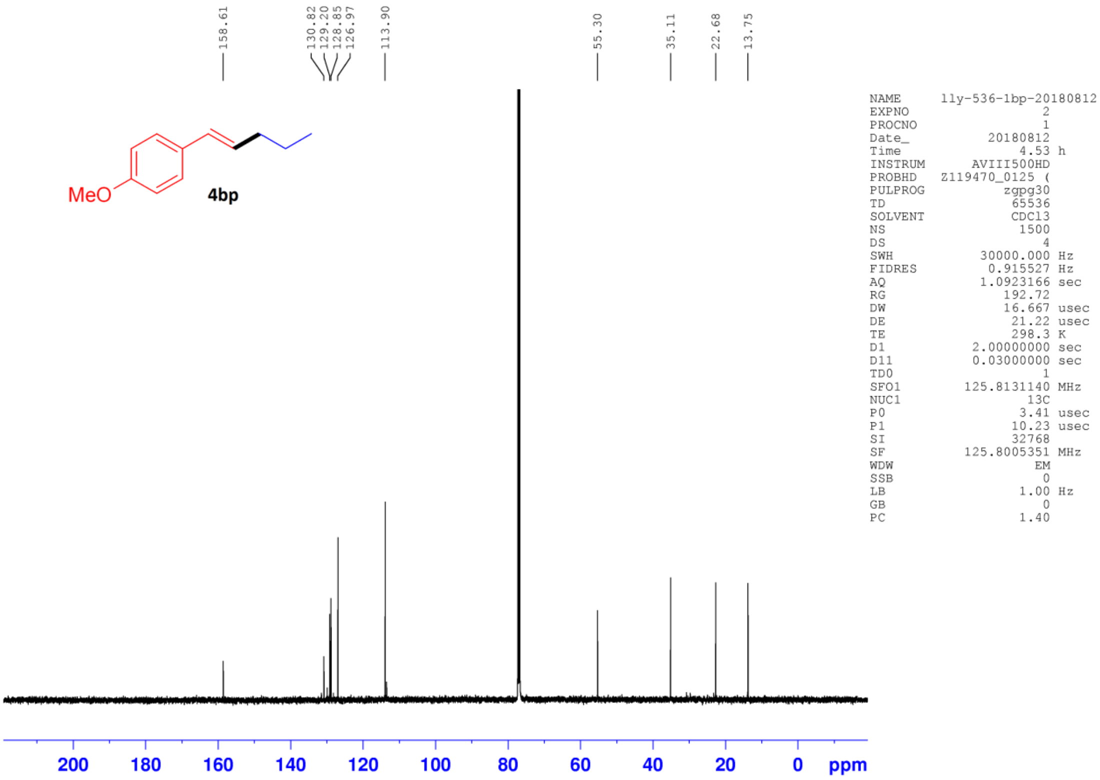

Supplementary Figure 72. ^{13}C NMR spectra for compound **4bp**

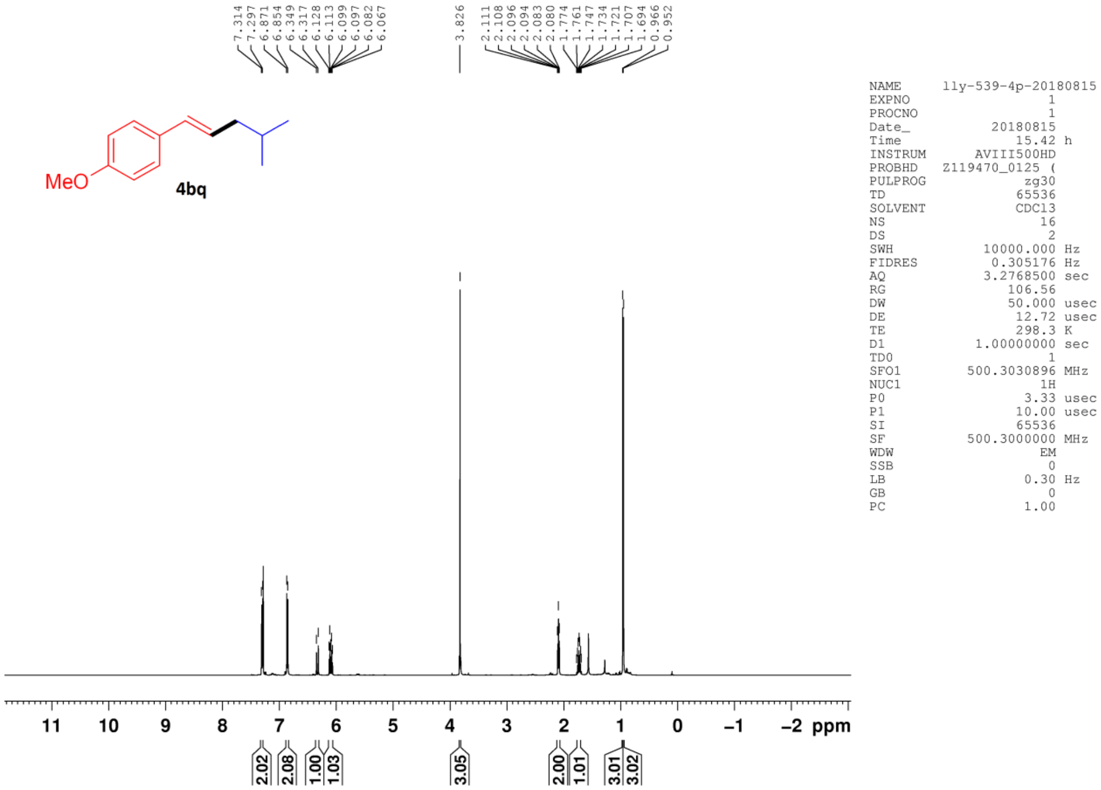

Supplementary Figure 73. ^{1}H NMR spectra for compound **4bq**

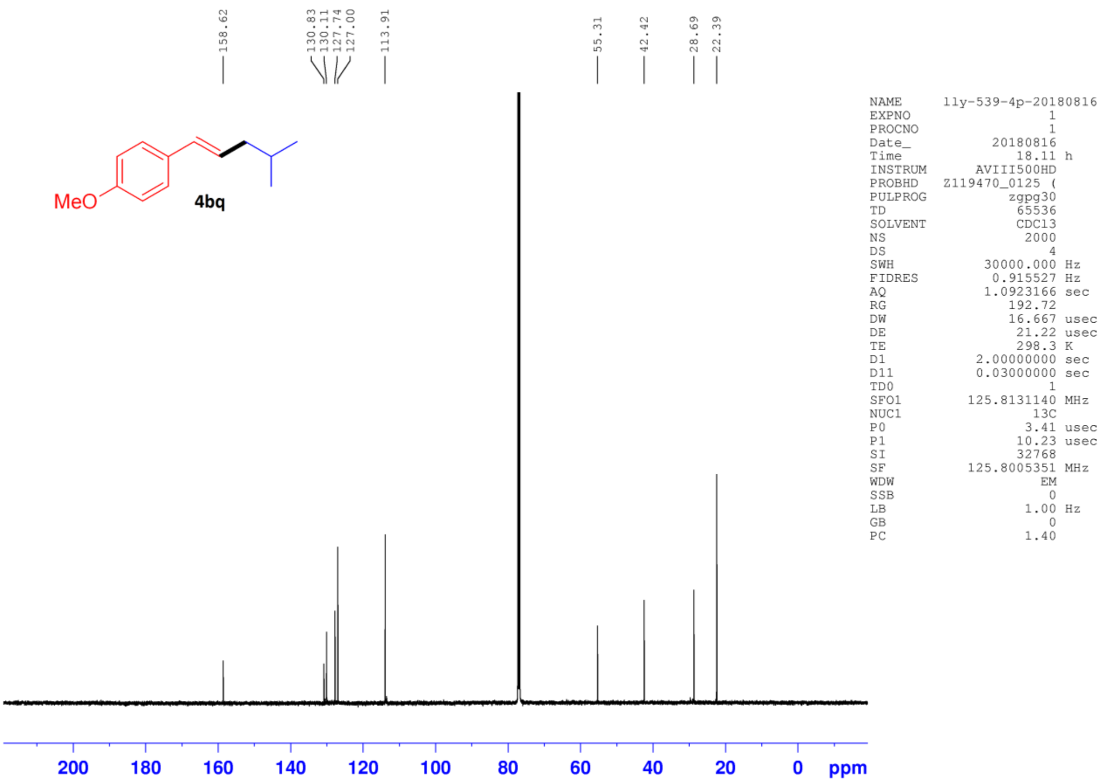

Supplementary Figure 74. ^{13}C NMR spectra for compound **4bq**

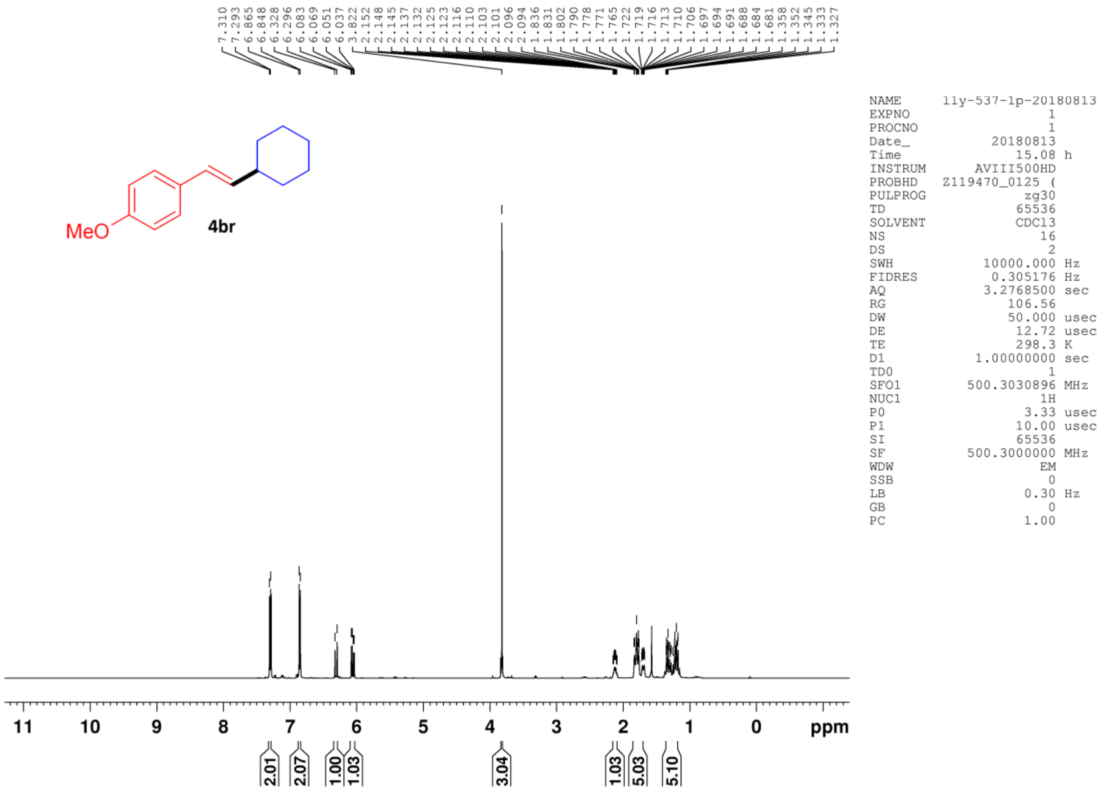

Supplementary Figure 75. ^{1}H NMR spectra for compound **4br**

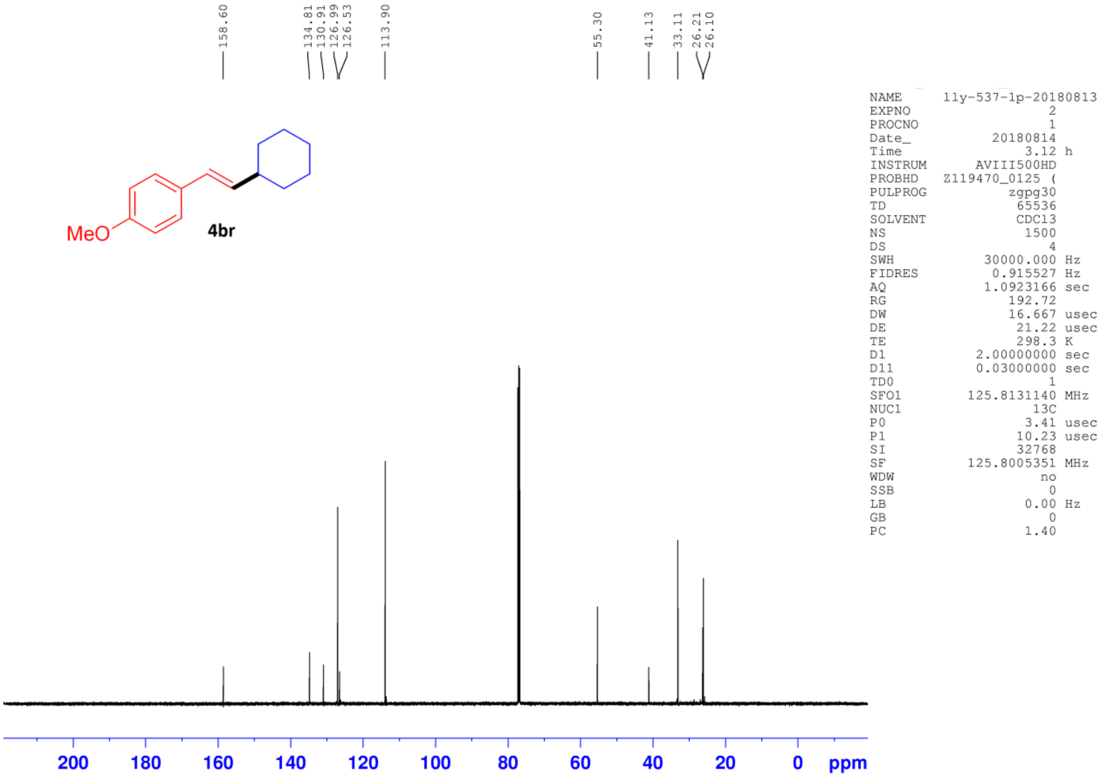

Supplementary Figure 76. ^{13}C NMR spectra for compound **4br**

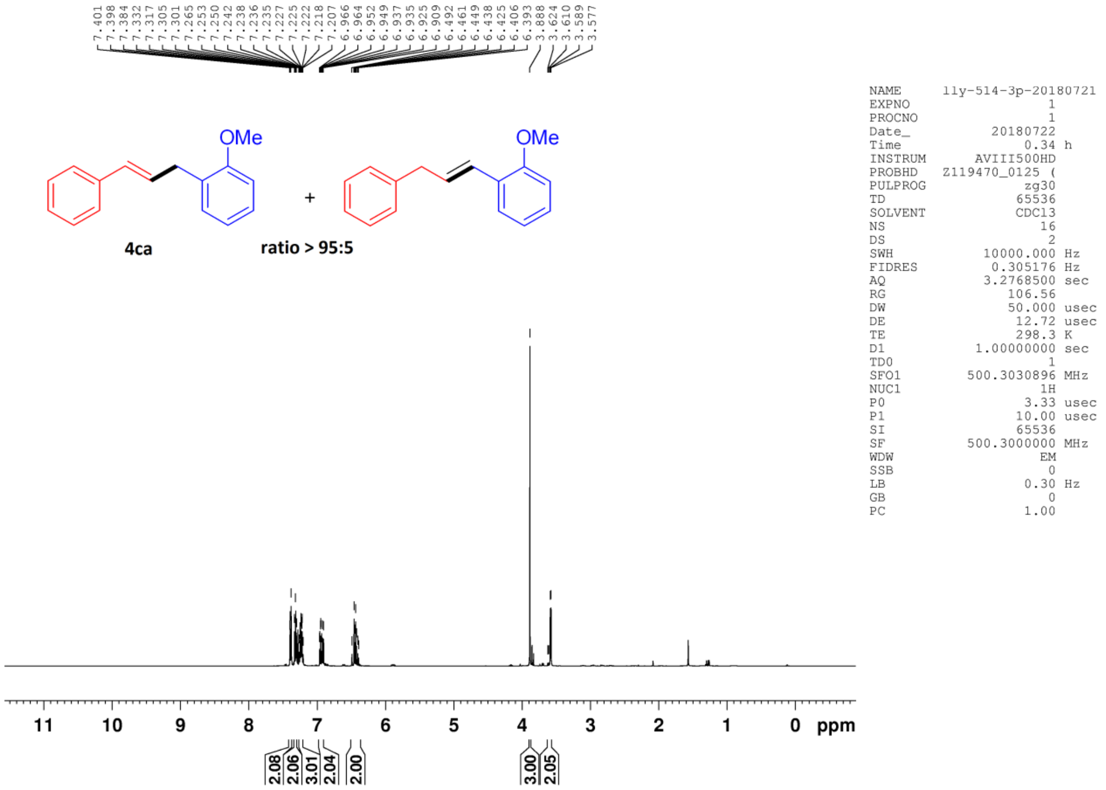

Supplementary Figure 77. ^{1}H NMR spectra for compound **4ca**

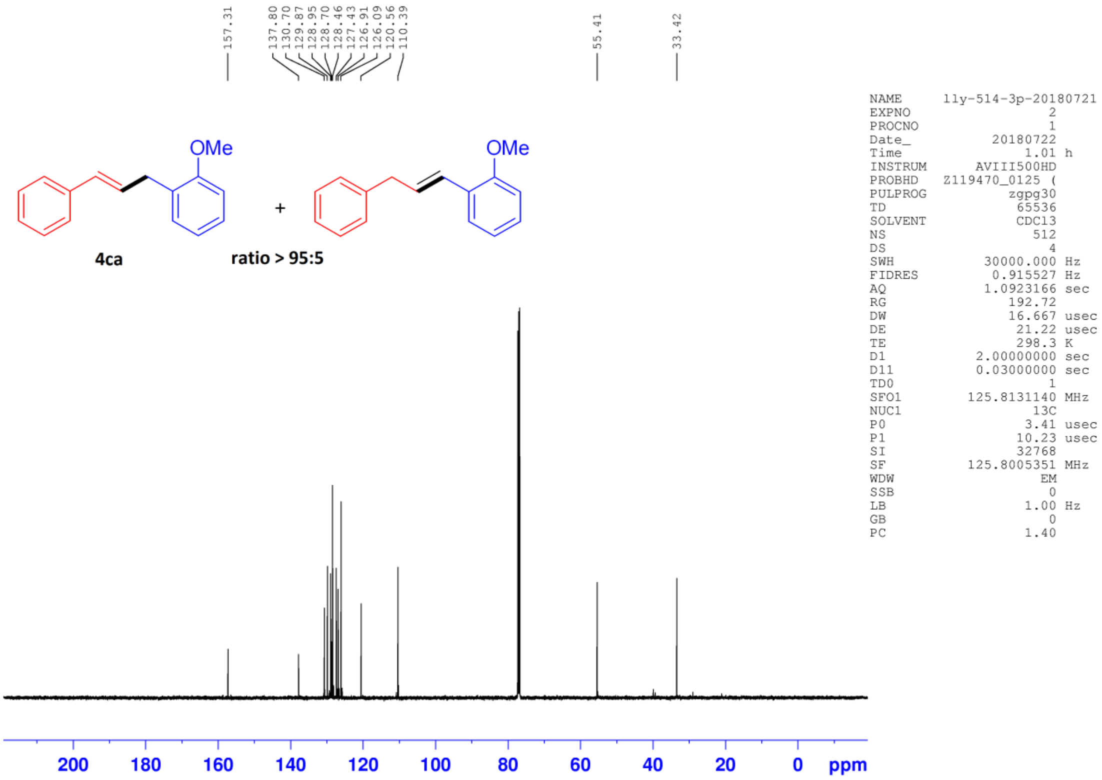

Supplementary Figure 78. ^{13}C NMR spectra for compound **4ca**

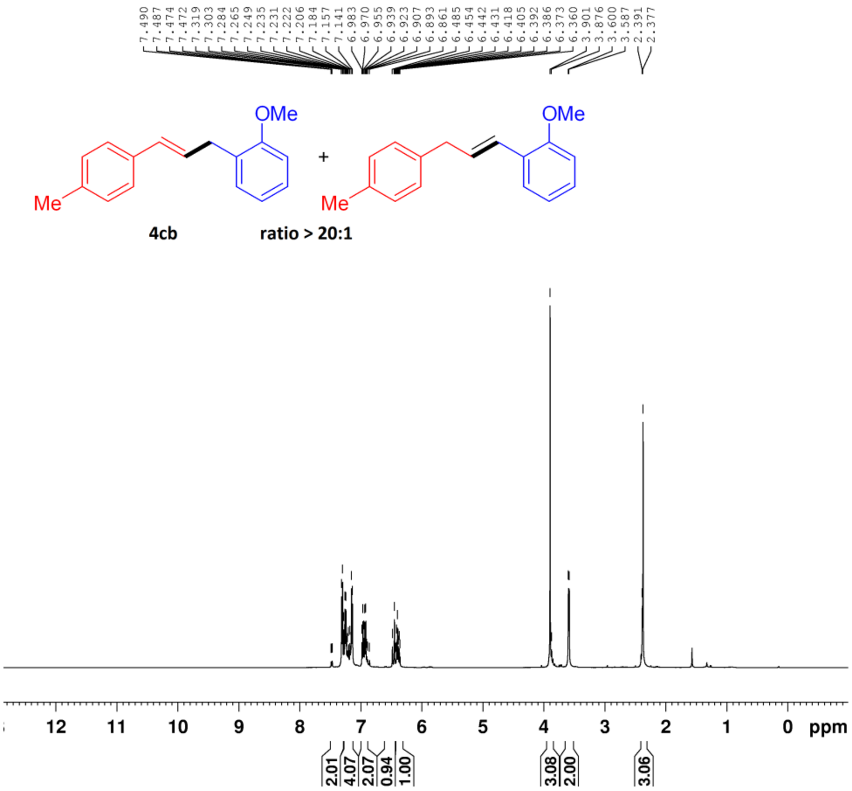


```
NAME       lly-528-3p-20180801
EXPNO                     1
PROCNO                    1
Date_              20180801
Time                  14.12 h
INSTRUM          AVIII500HD
PROBHD     Z119470_0125 (
PULPROG                zg30
TD                    65536
SOLVENT               CDCl3
NS                       16
DS                        2
SWH               10000.000 Hz
FIDRES             0.305176 Hz
AQ                3.2768500 sec
RG                    32.48
DW                   50.000 usec
DE                    12.72 usec
TE                    298.3 K
D1               1.00000000 sec
TD0                       1
SFO1            500.3030896 MHz
NUC1                     1H
P0                     3.33 usec
P1                    10.00 usec
SI                    65536
SF              500.3000000 MHz
WDW                      EM
SSB                       0
LB                     0.30 Hz
GB                        0
PC                     1.00
```

Supplementary Figure 79. ^{1}H NMR spectra for compound **4cb**

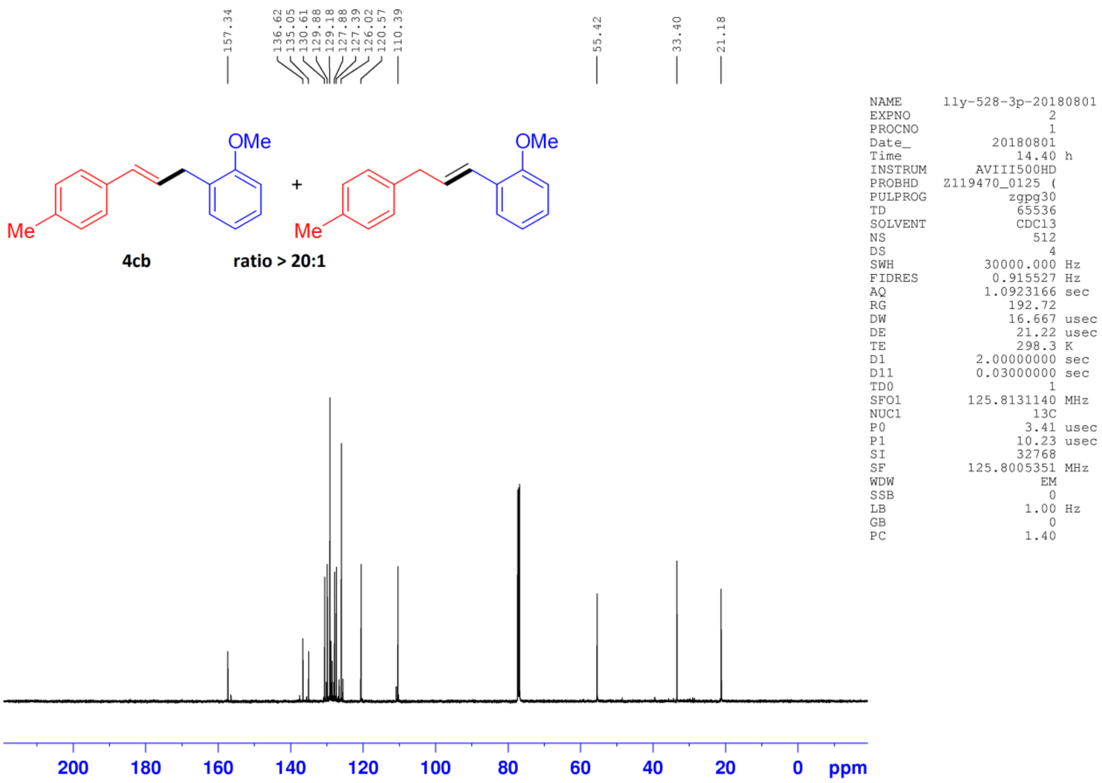

Supplementary Figure 80. ^{13}C NMR spectra for compound **4ca**

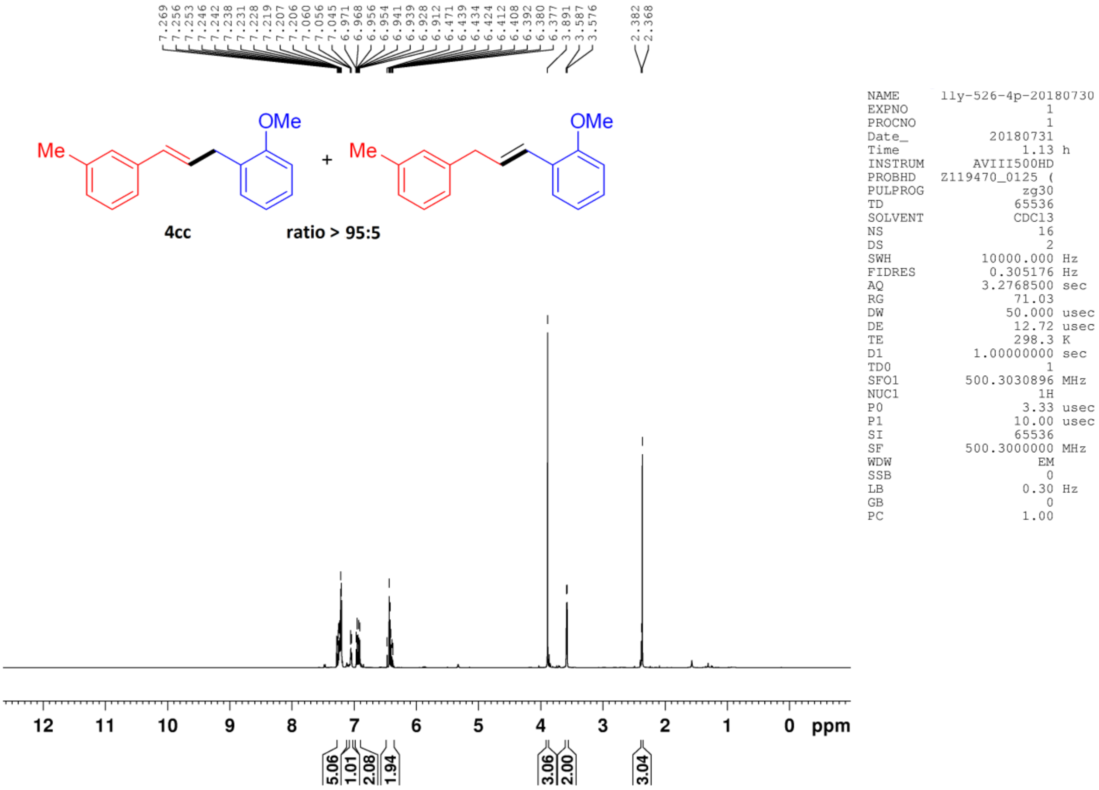

Supplementary Figure 81. ^{1}H NMR spectra for compound **4cc**

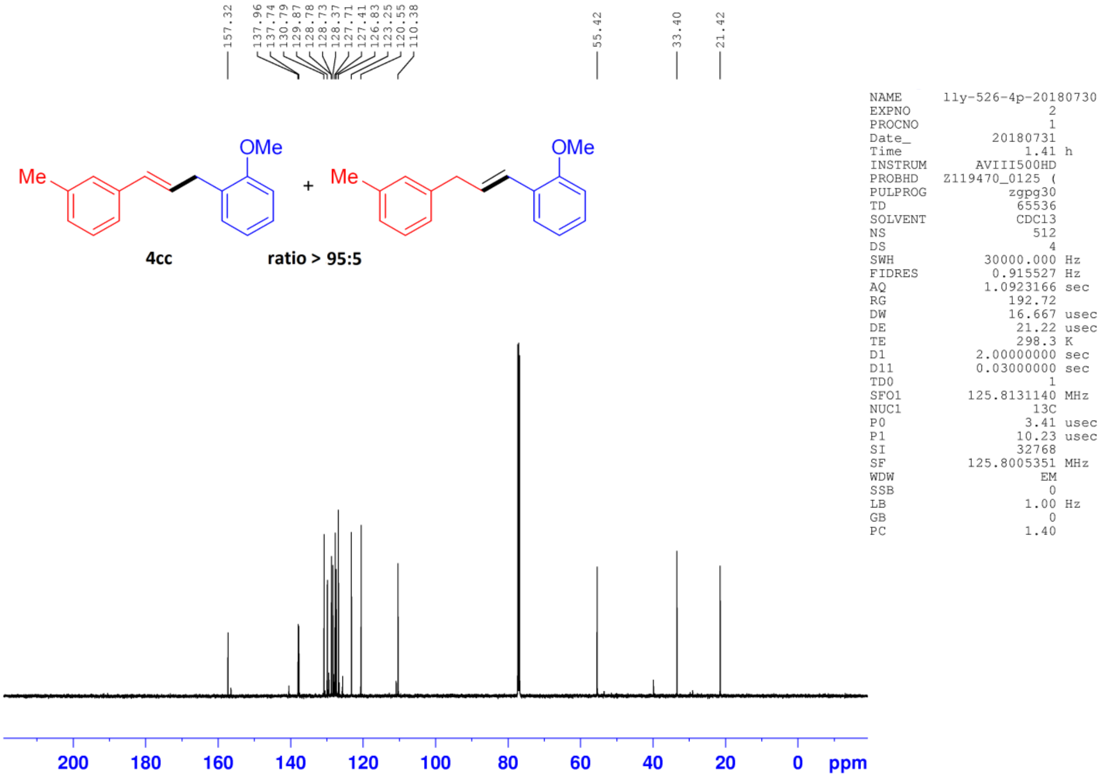

Supplementary Figure 82. ^{13}C NMR spectra for compound **4cc**

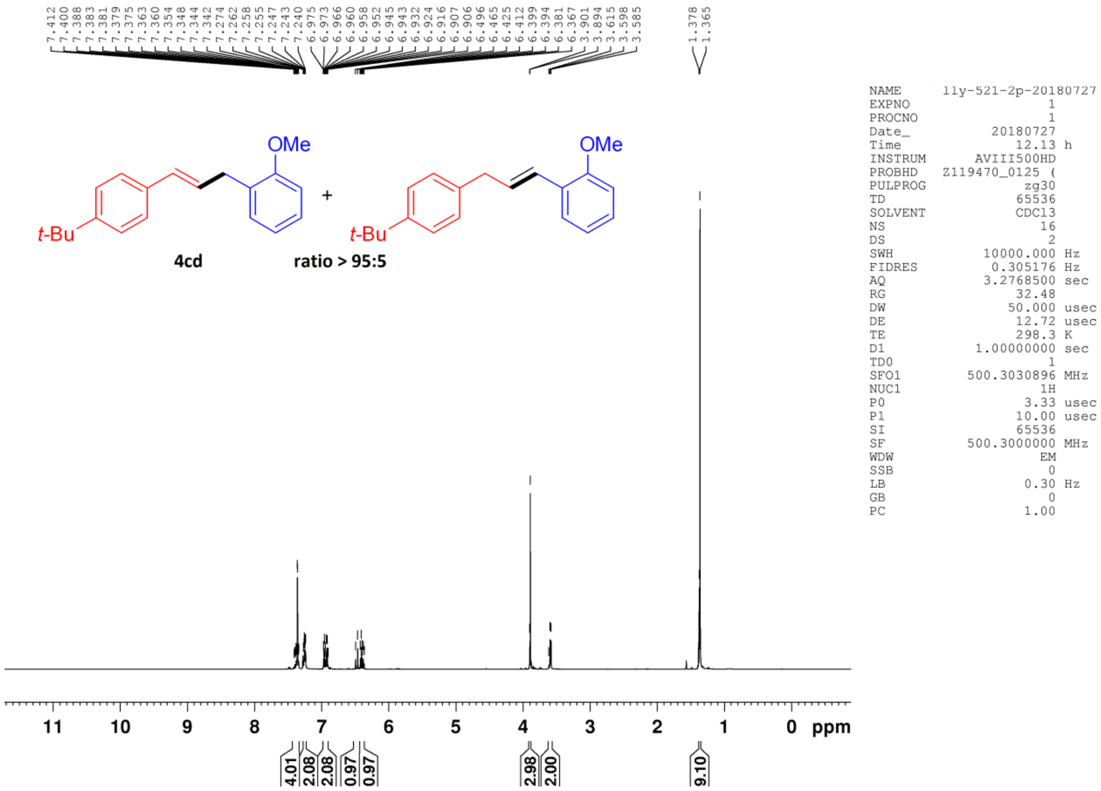

Supplementary Figure 83. ^{1}H NMR spectra for compound **4cd**

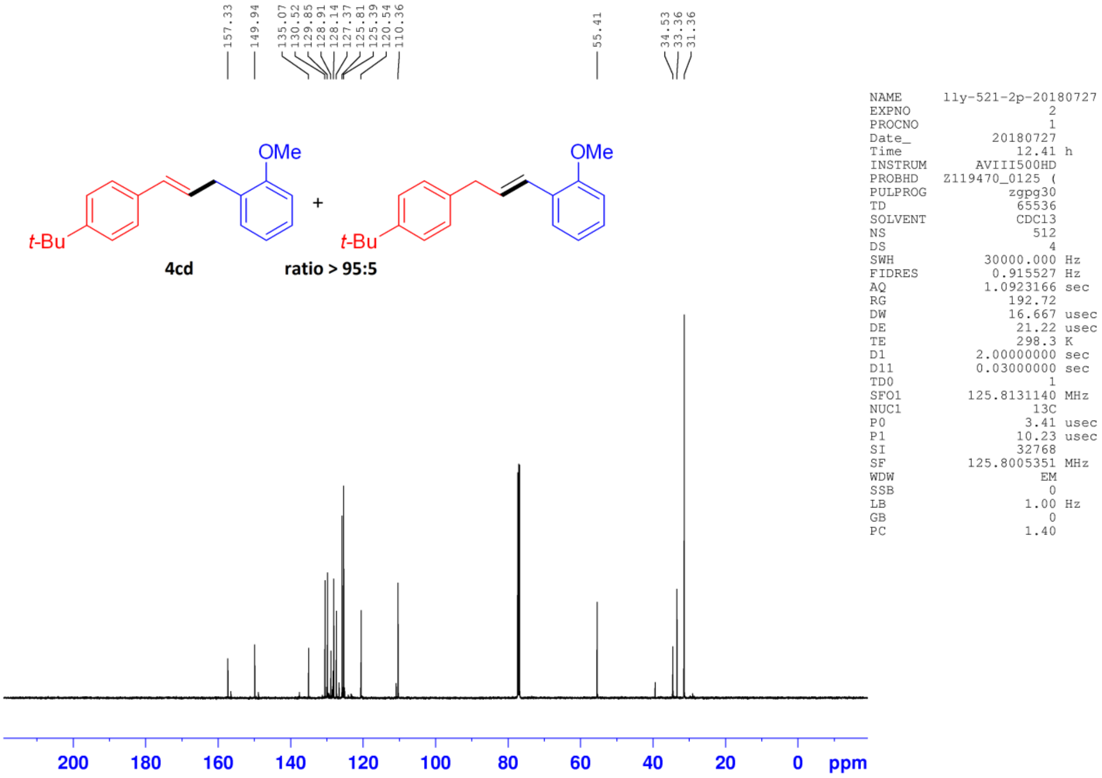

Supplementary Figure 84. ^{13}C NMR spectra for compound **4cd**

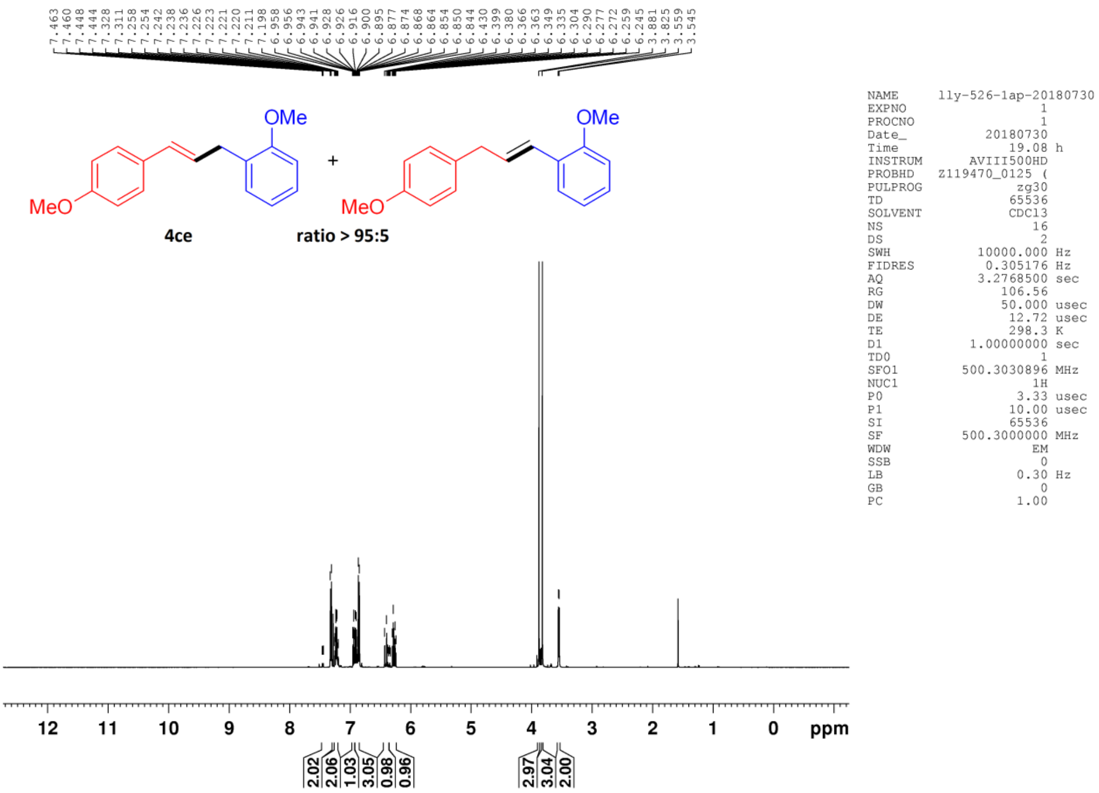

Supplementary Figure 85. ^{1}H NMR spectra for compound **4ce**

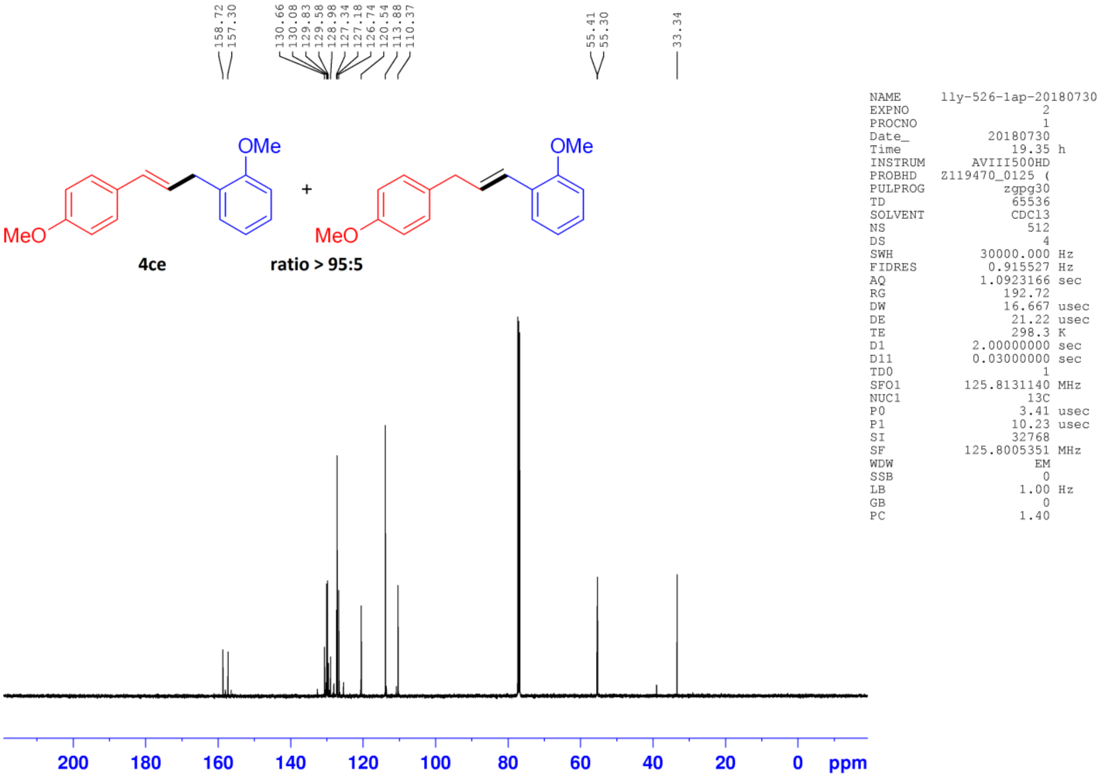

Supplementary Figure 86. ^{13}C NMR spectra for compound **4ce**

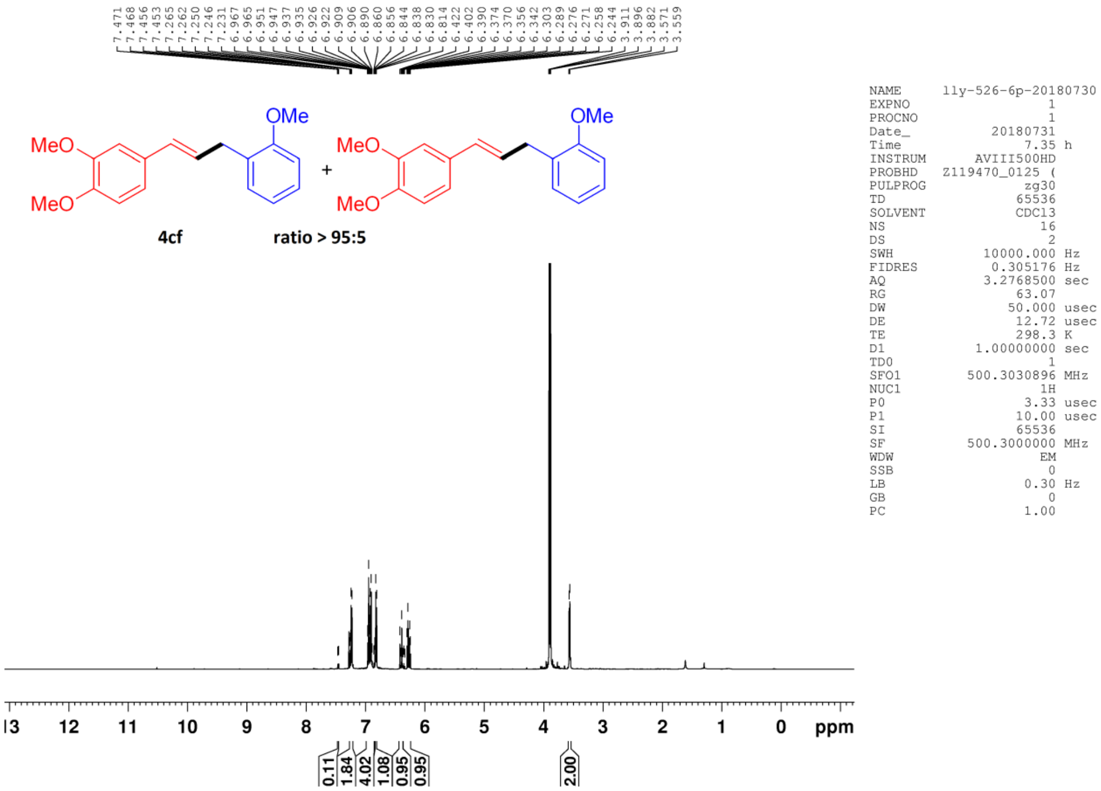

Supplementary Figure 87. ^{1}H NMR spectra for compound **4cf**

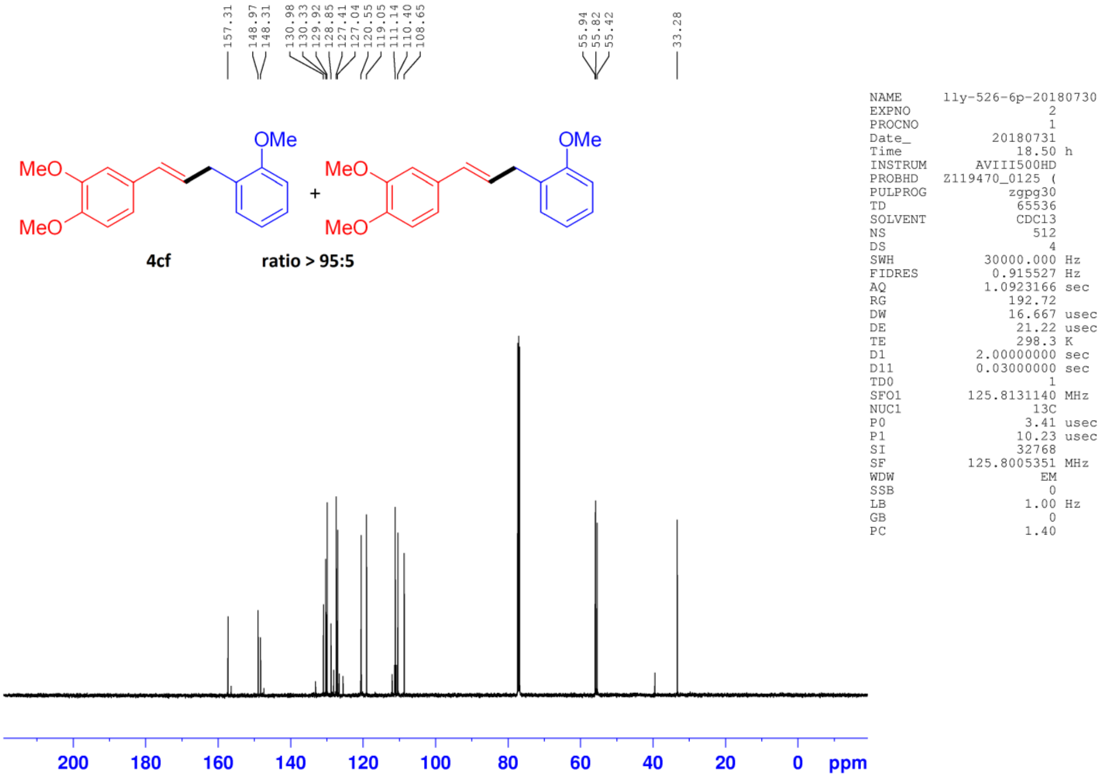

Supplementary Figure 88. ^{13}C NMR spectra for compound **4cf**

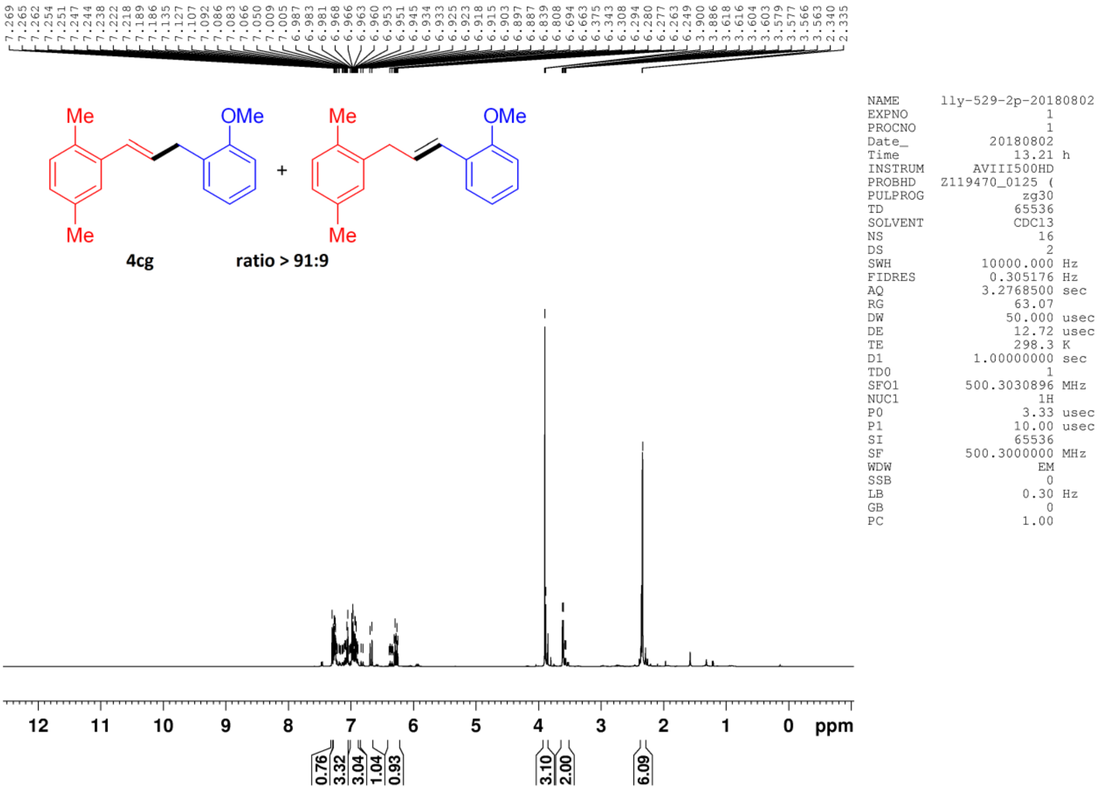

Supplementary Figure 89. ^{1}H NMR spectra for compound **4cg**

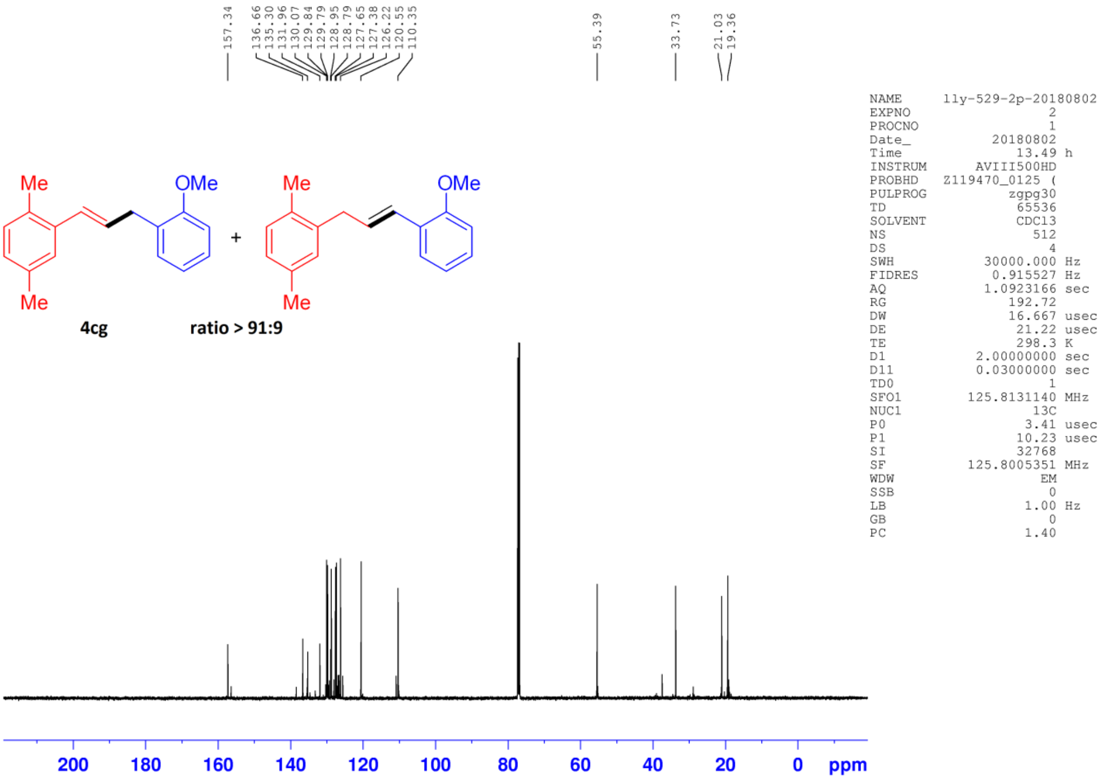

Supplementary Figure 90. ^{13}C NMR spectra for compound **4cg**

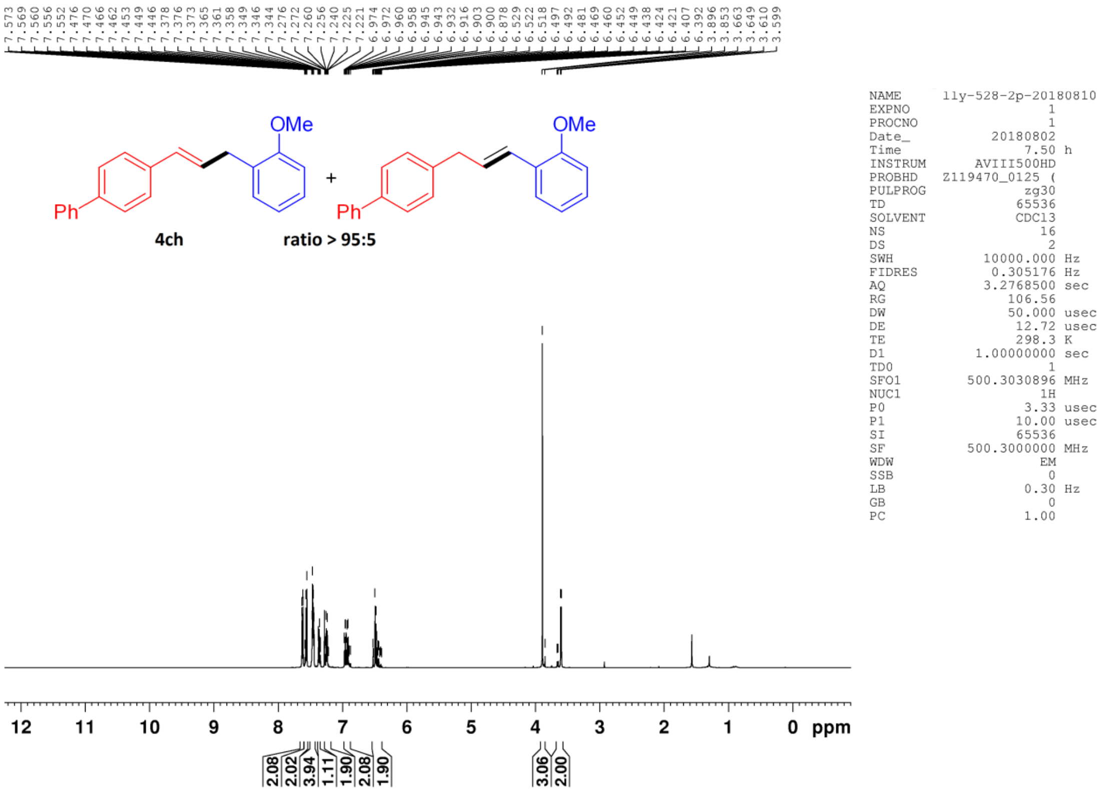

Supplementary Figure 91. ^{1}H NMR spectra for compound **4ch**

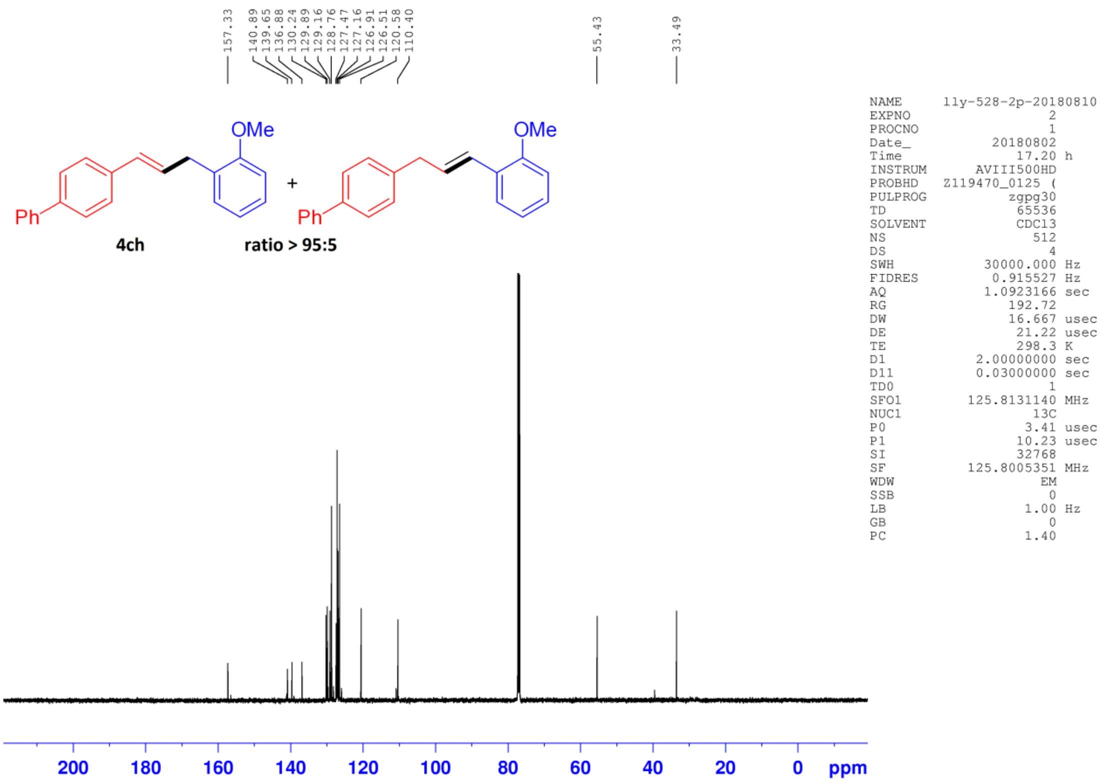

Supplementary Figure 92. ^{13}C NMR spectra for compound **4ch**

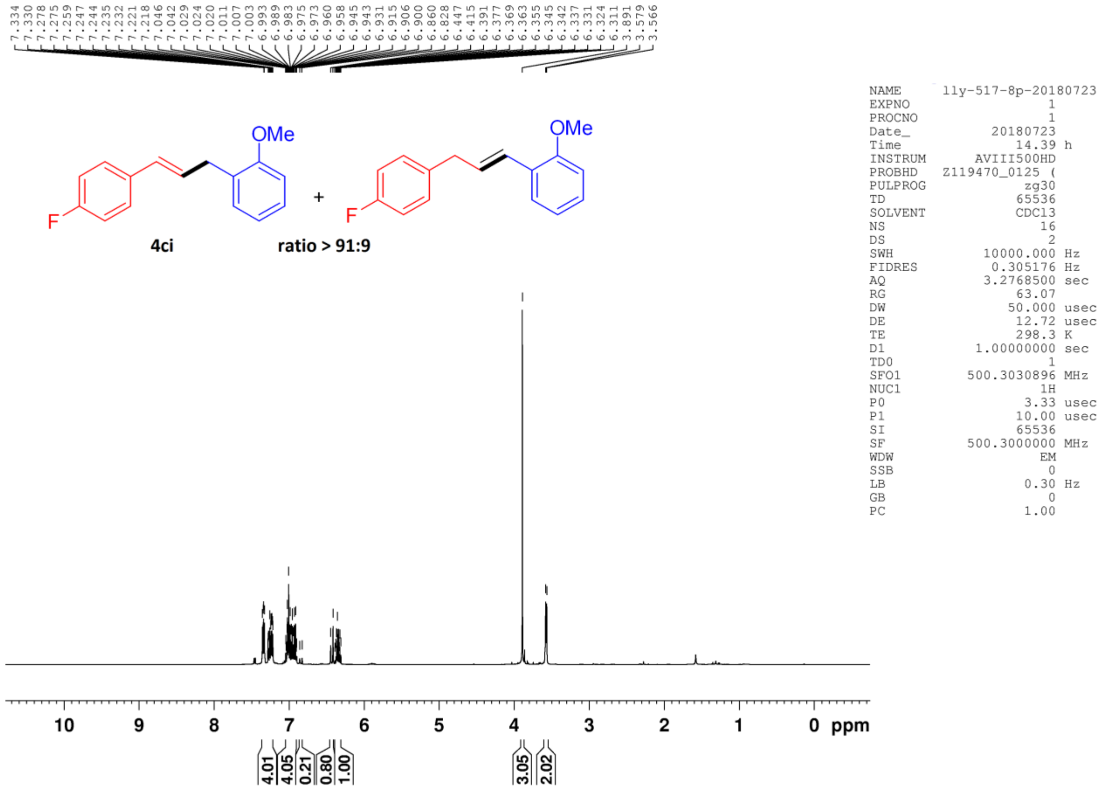

Supplementary Figure 93. ^{1}H NMR spectra for compound **4ci**

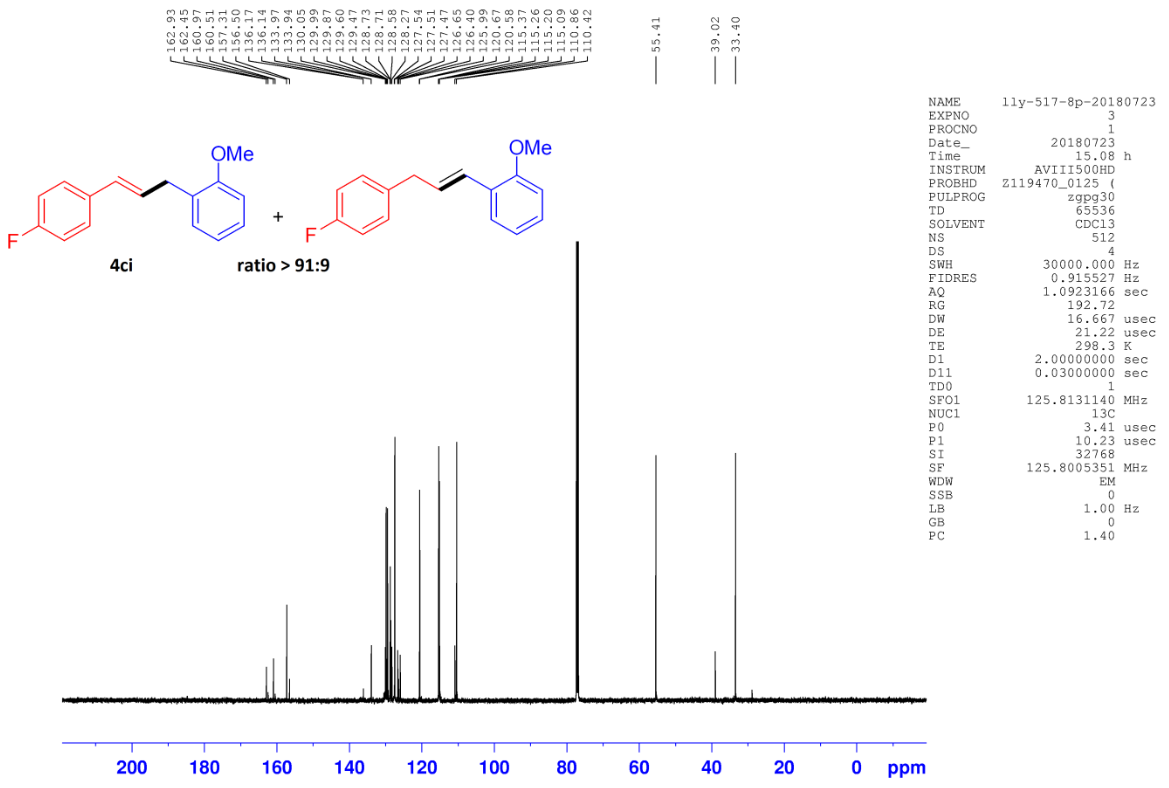

Supplementary Figure 94. ^{13}C NMR spectra for compound **4ci**

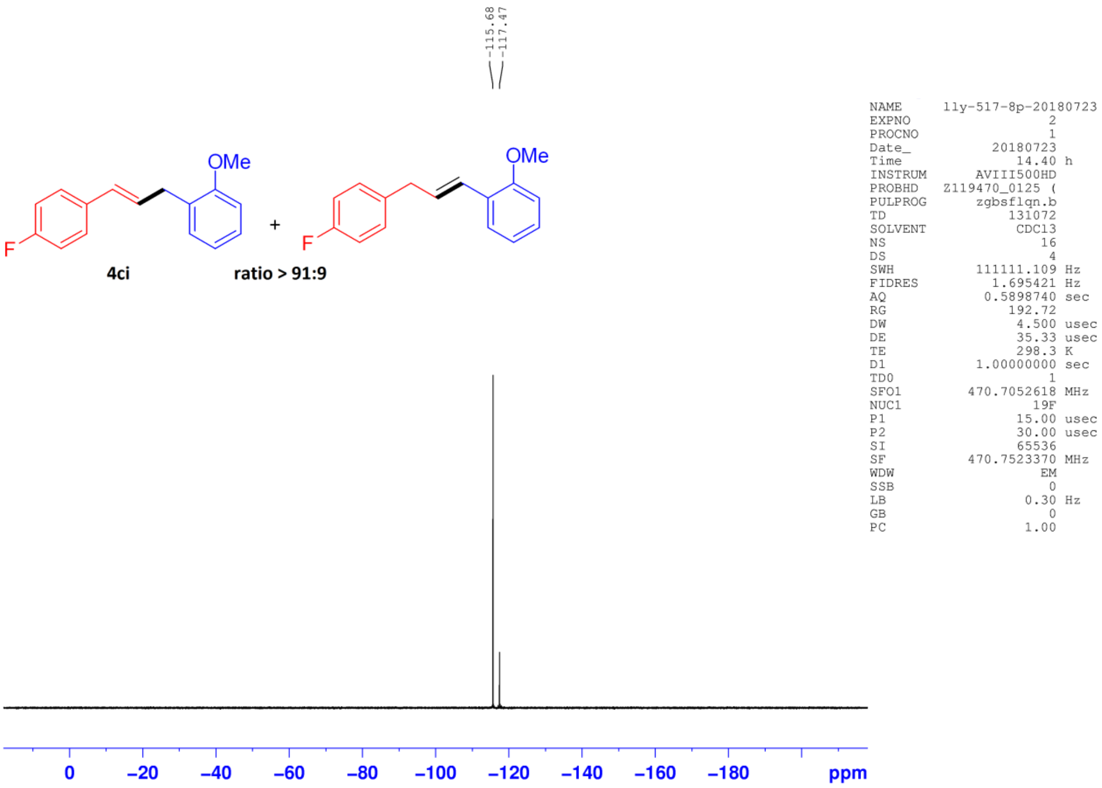

Supplementary Figure 95. ^{19}F NMR spectra for compound **4ci**

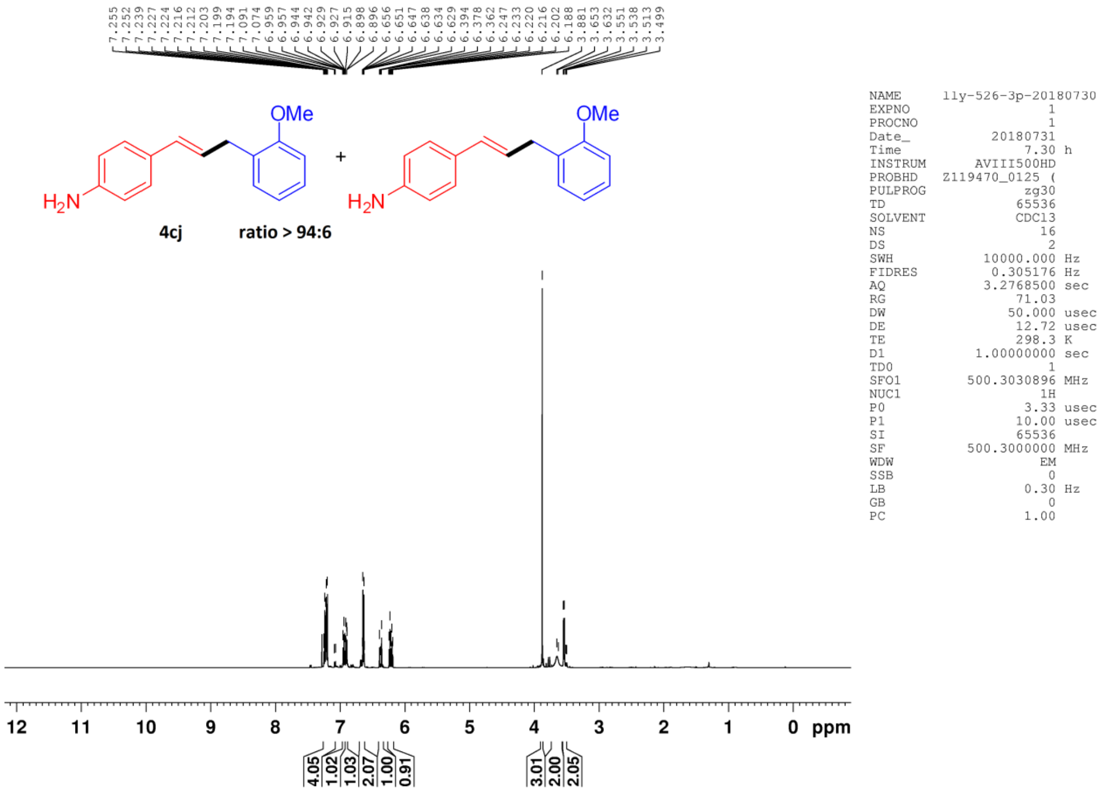

Supplementary Figure 96. ^{1}H NMR spectra for compound **4cj**

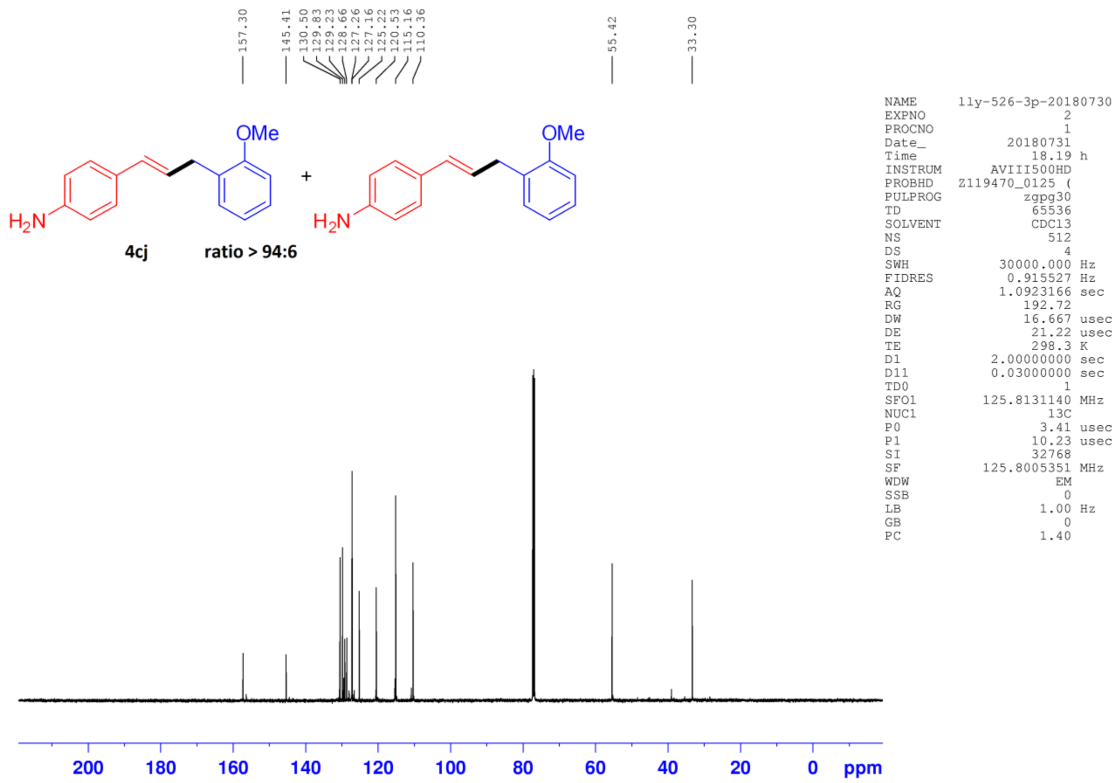

Supplementary Figure 97. ^{13}C NMR spectra for compound **4cj**

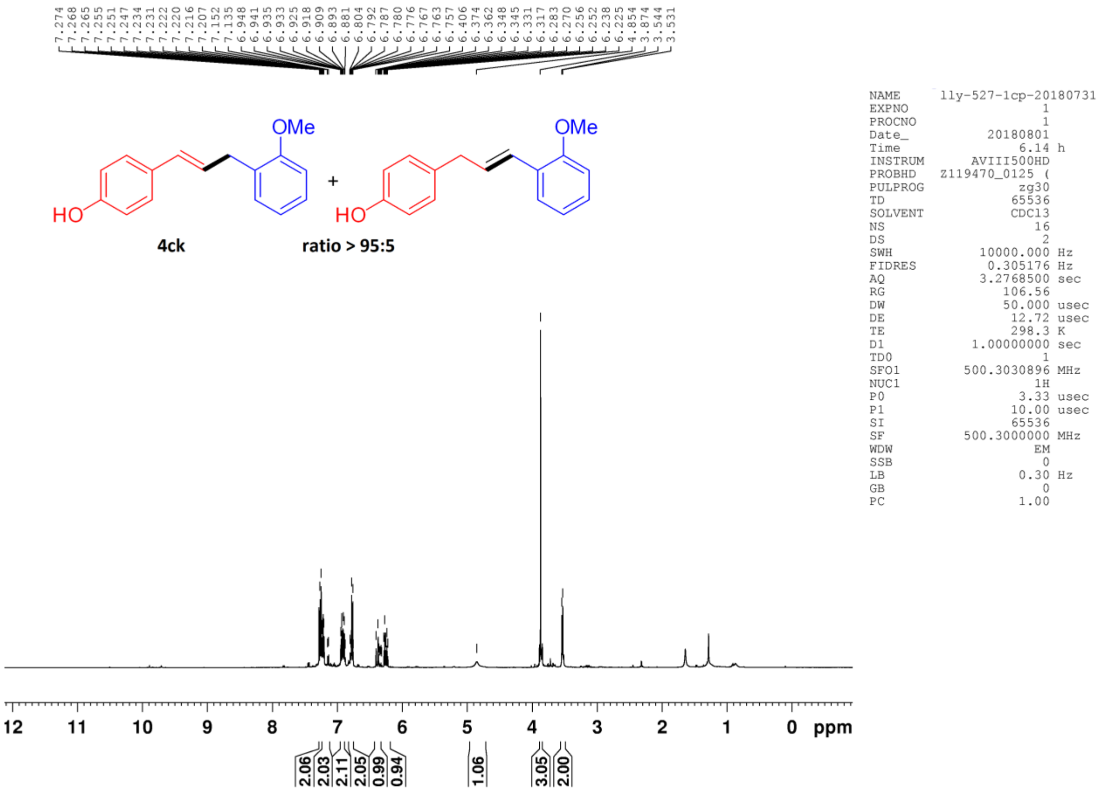

Supplementary Figure 98. ^{1}H NMR spectra for compound **4ck**

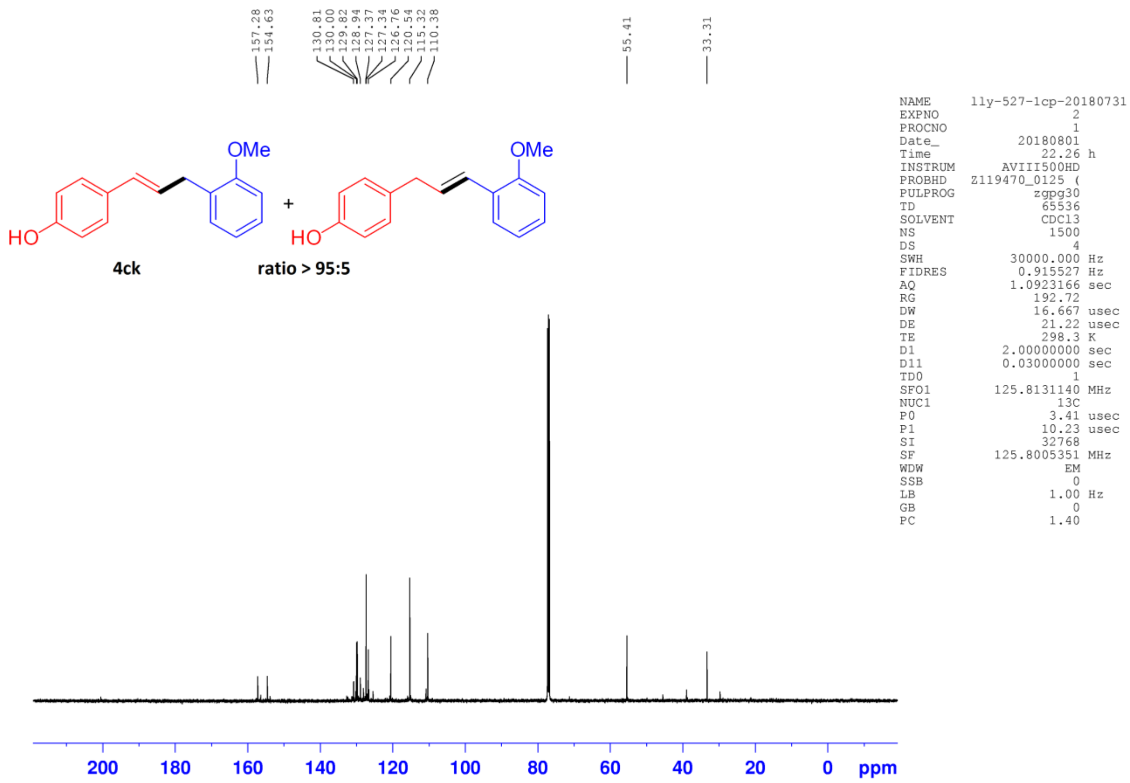

Supplementary Figure 99. ^{13}C NMR spectra for compound **4ck**

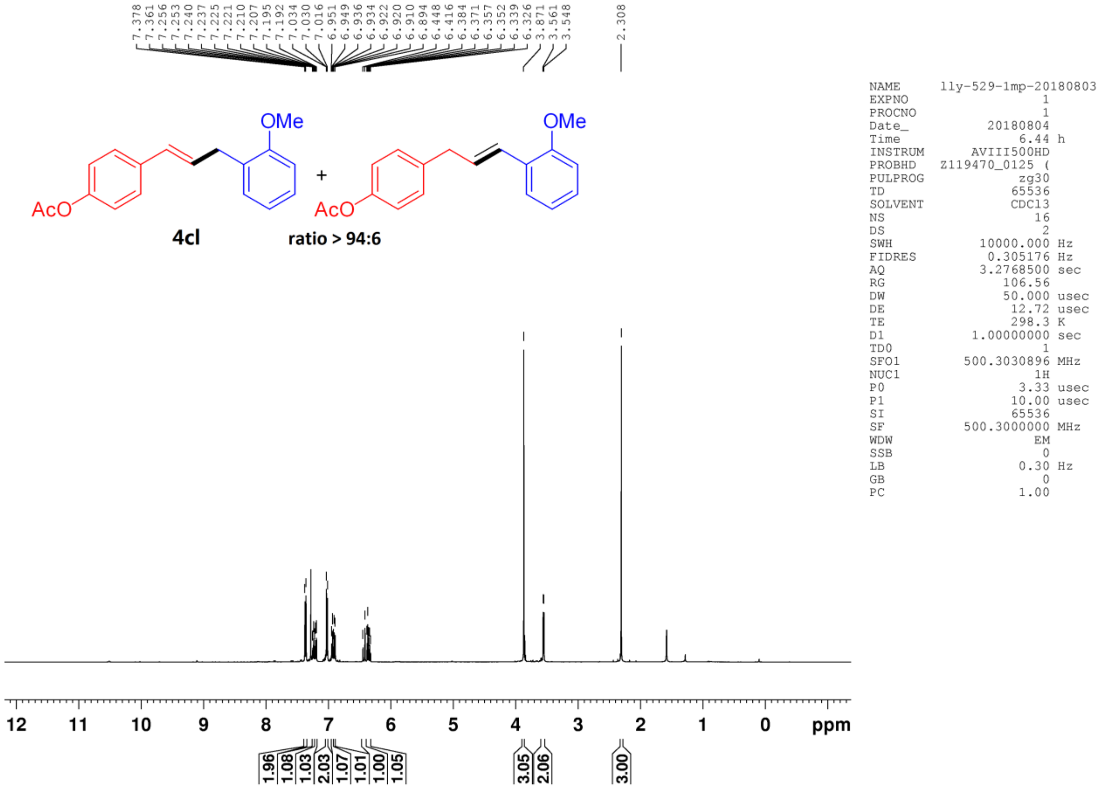

Supplementary Figure 100. ^{1}H NMR spectra for compound **4cl**

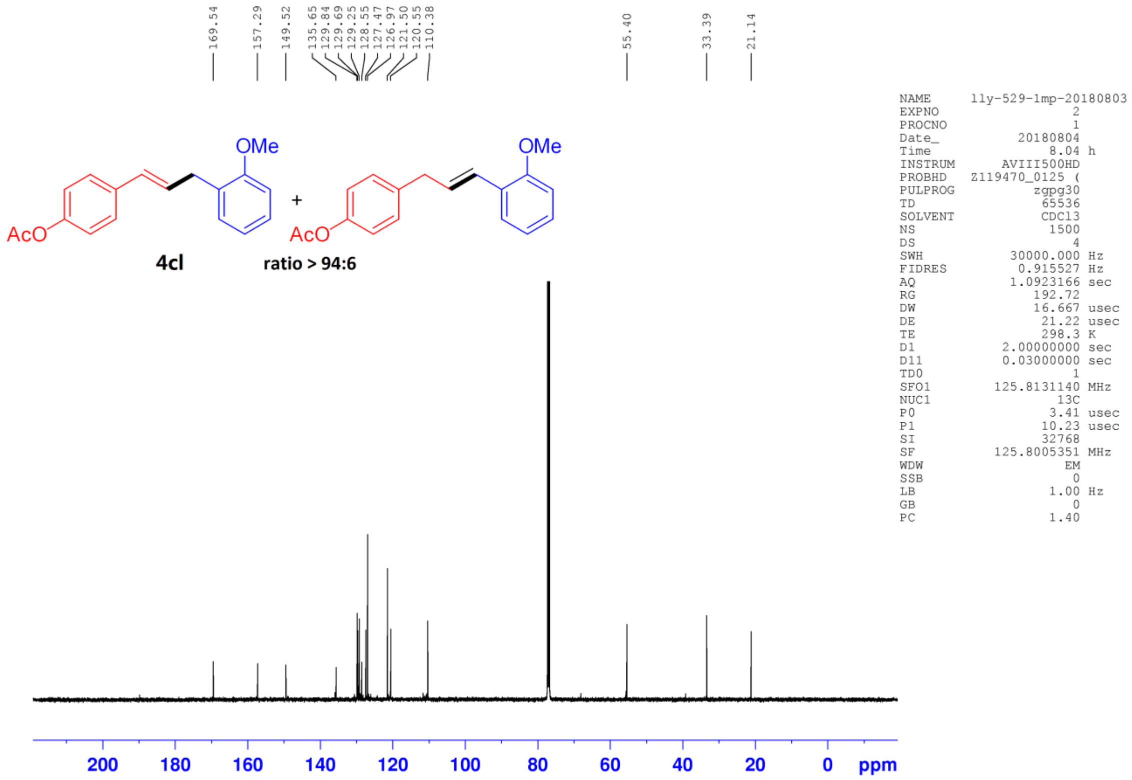

Supplementary Figure 101. ^{13}C NMR spectra for compound **4cl**

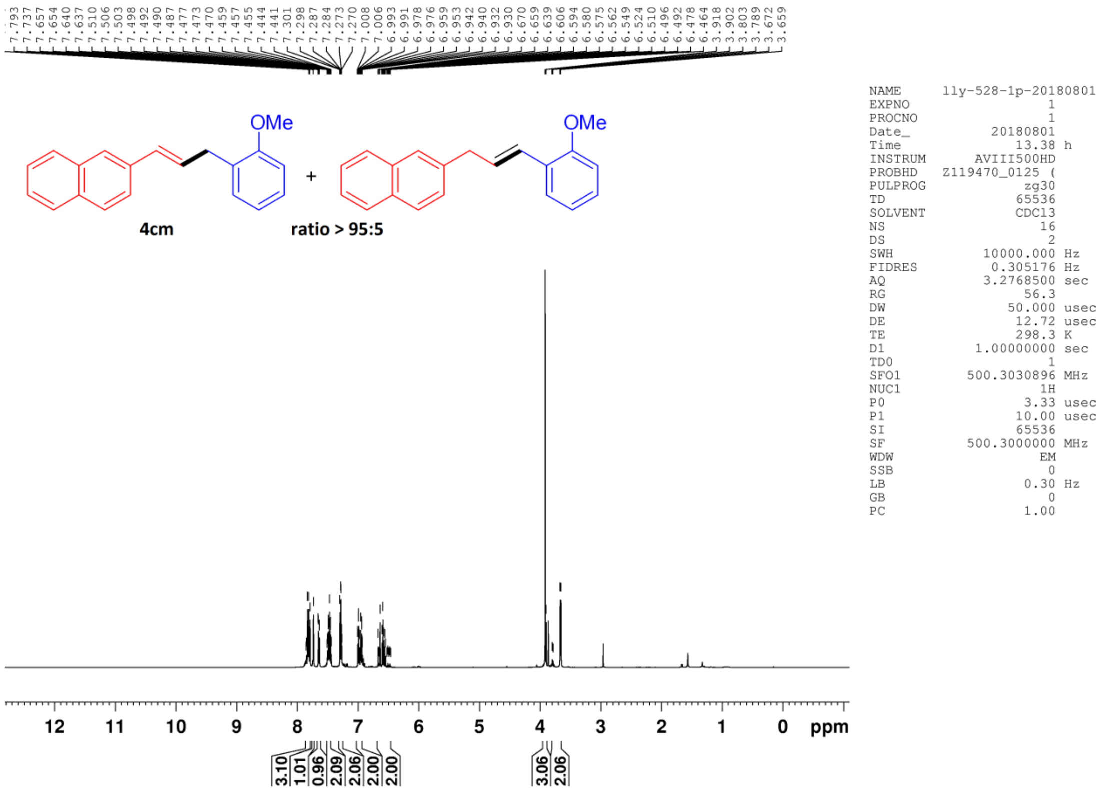

Supplementary Figure 102. ^{1}H NMR spectra for compound **4cm**

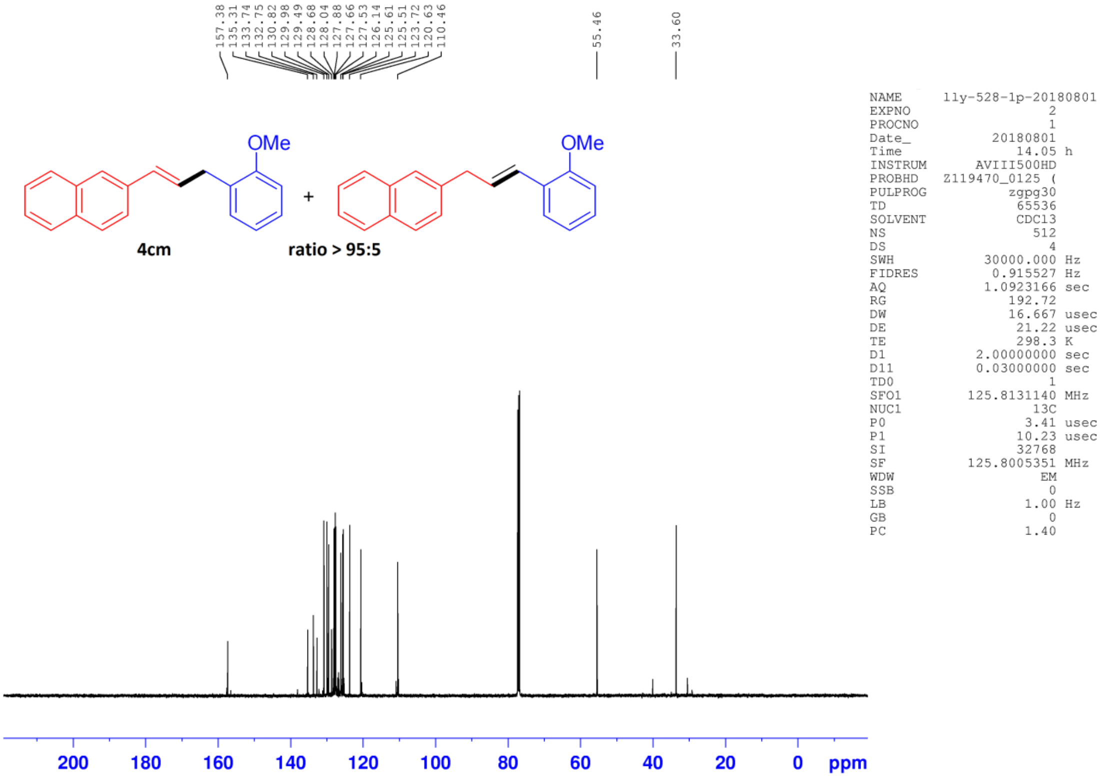

Supplementary Figure 103. ^{13}C NMR spectra for compound **4cm**

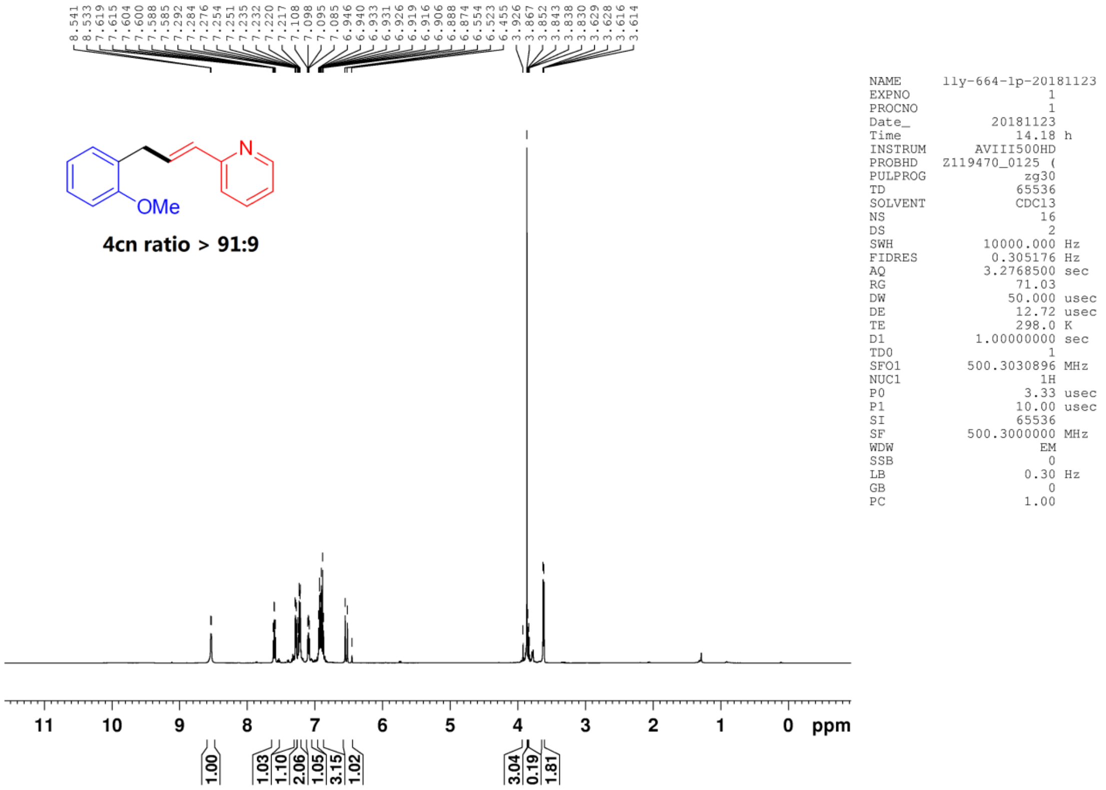

Supplementary Figure 104. ^{1}H NMR spectra for compound **4cn**

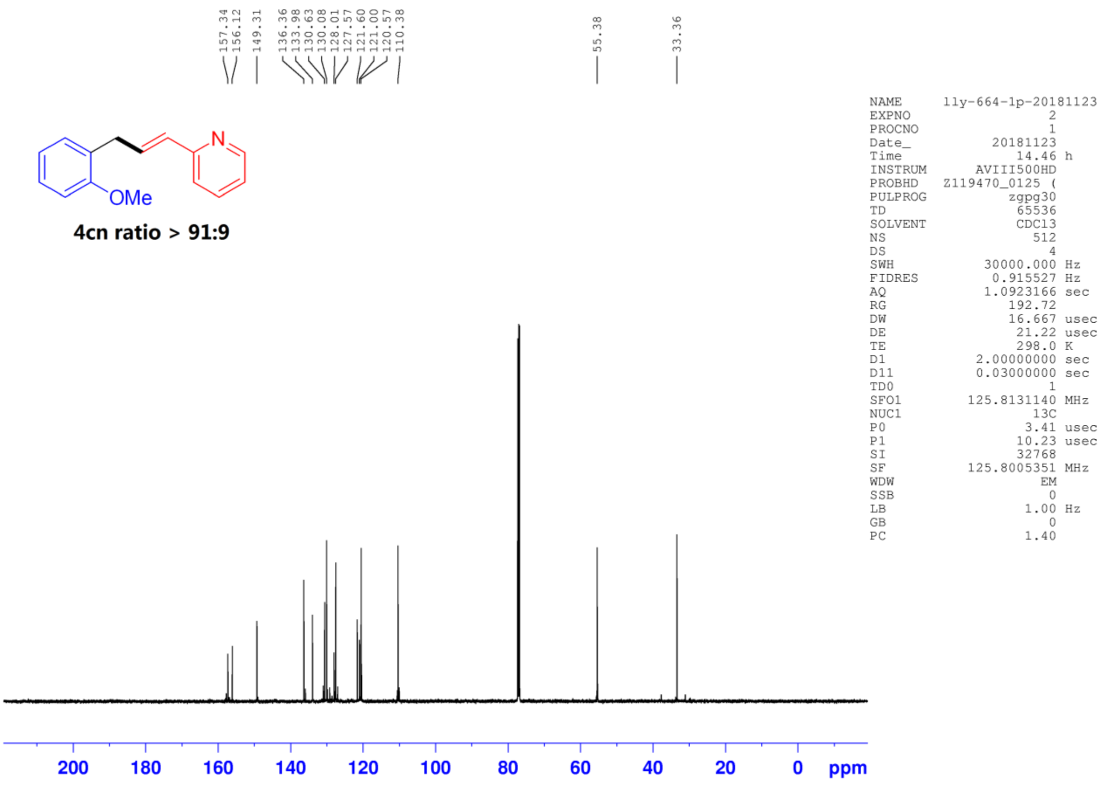

Supplementary Figure 105. ^{13}C NMR spectra for compound **4cn**

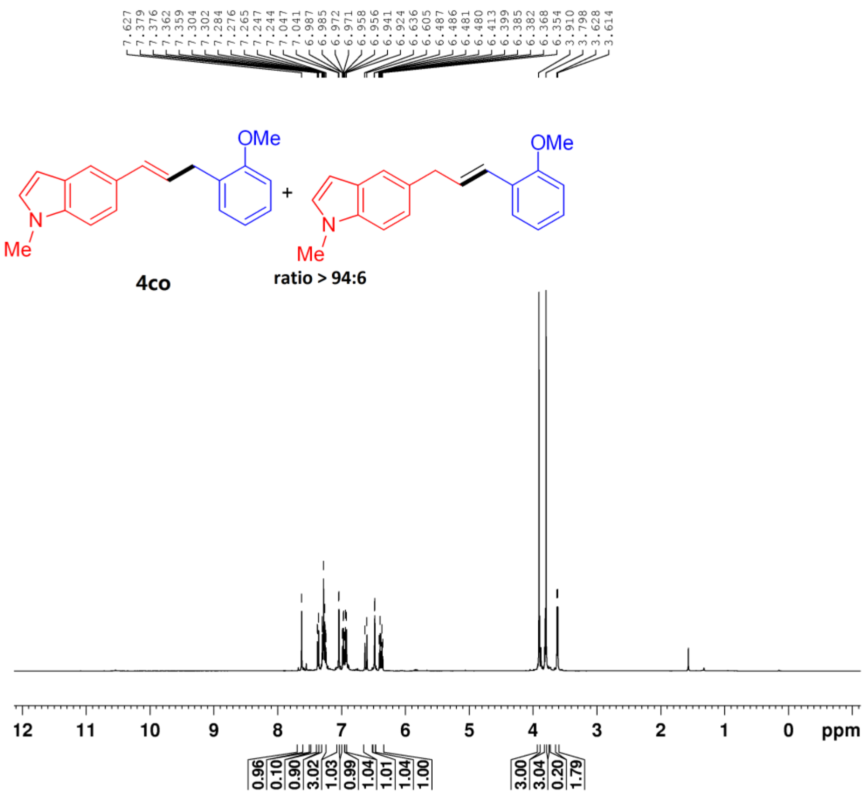

NAME	lly-535-1ap-20180811
EXPNO	1
PROCNO	1
Date_	20180811
Time	13.13 h
INSTRUM	AVIII500HD
PROBHD	Z119470_0125 (
PULPROG	zg30
TD	65536
SOLVENT	CDCl3
NS	16
DS	2
SWH	10000.000 Hz
FIDRES	0.305176 Hz
AQ	3.2768500 sec
RG	63.07
DW	50.000 usec
DE	12.72 usec
TE	298.3 K
D1	1.00000000 sec
TD0	1
SFO1	500.3030896 MHz
NUC1	1H
P0	3.33 usec
P1	10.00 usec
SI	65536
SF	500.3000000 MHz
WDW	EM
SSB	0
LB	0.30 Hz
GB	0
PC	1.00

Supplementary Figure 106. ^{1}H NMR spectra for compound **4co**

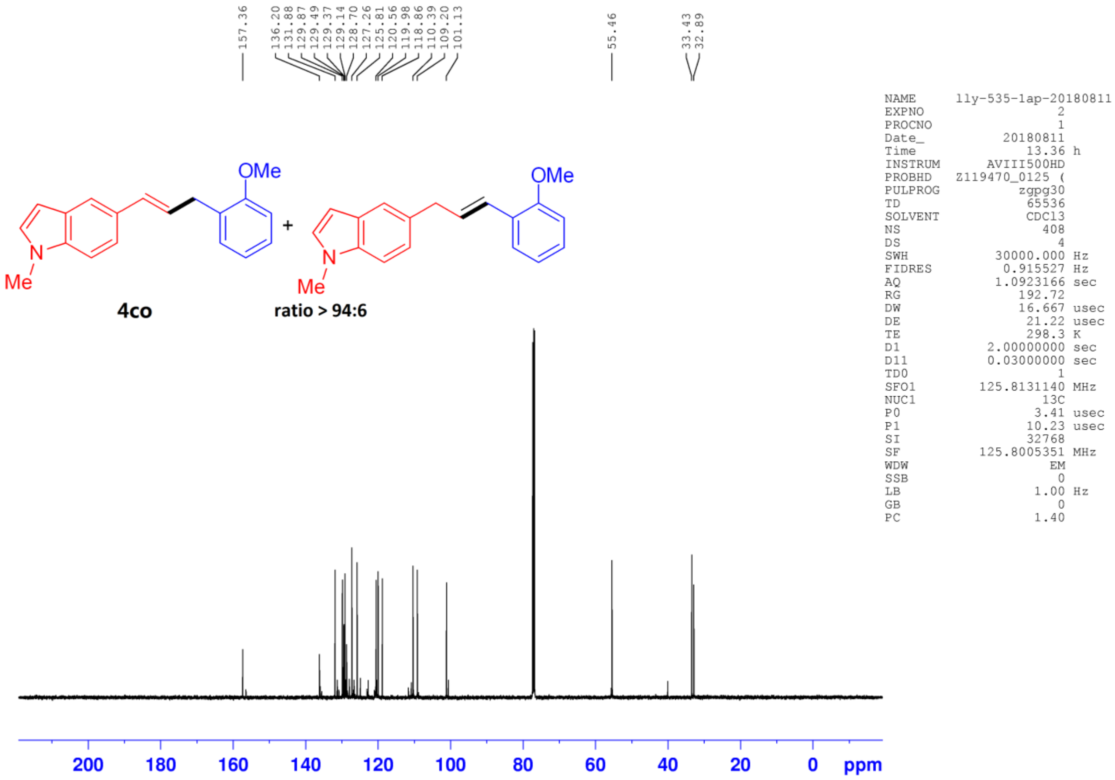

Supplementary Figure 107. ^{13}C NMR spectra for compound **4co**

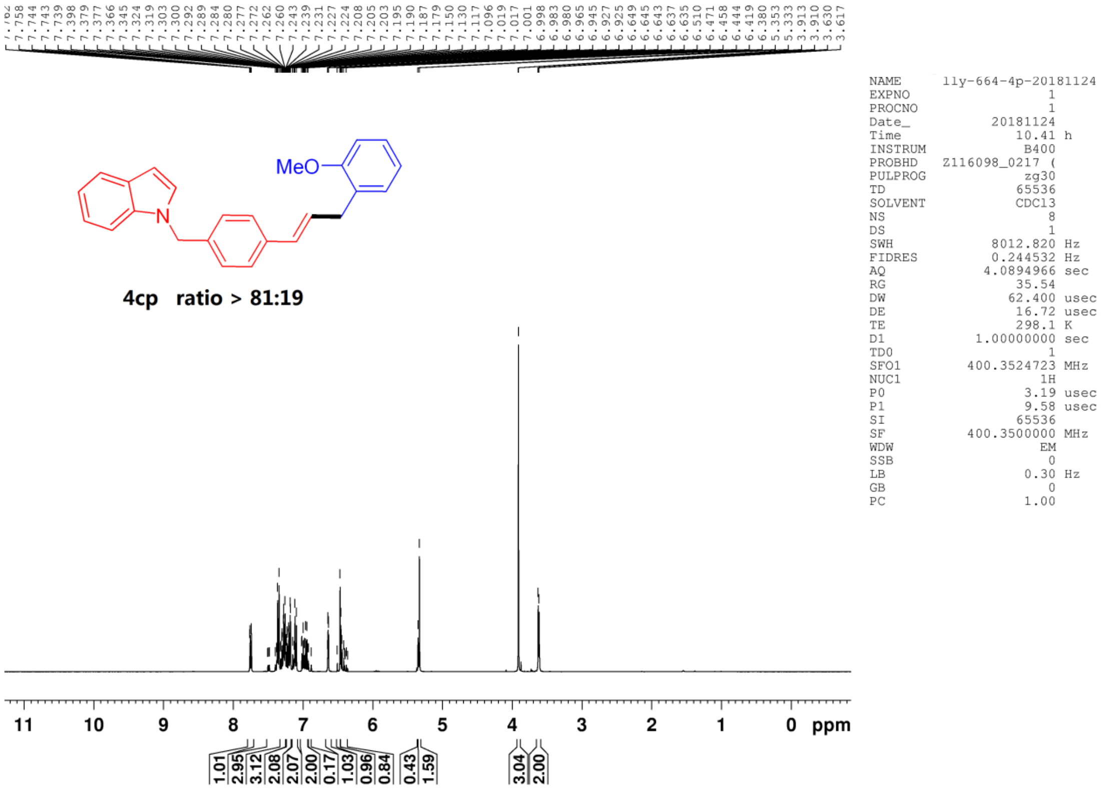

Supplementary Figure 108. ^{1}H NMR spectra for compound **4cp**

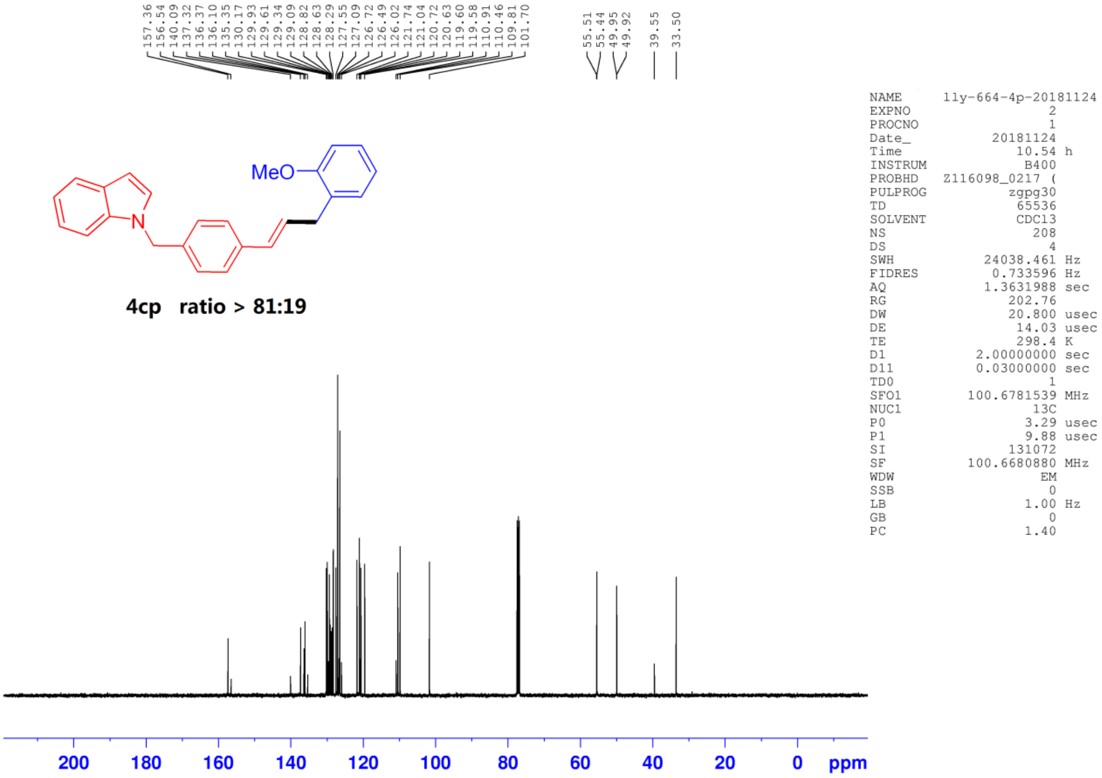

Supplementary Figure 109. ^{13}C NMR spectra for compound **4cp**

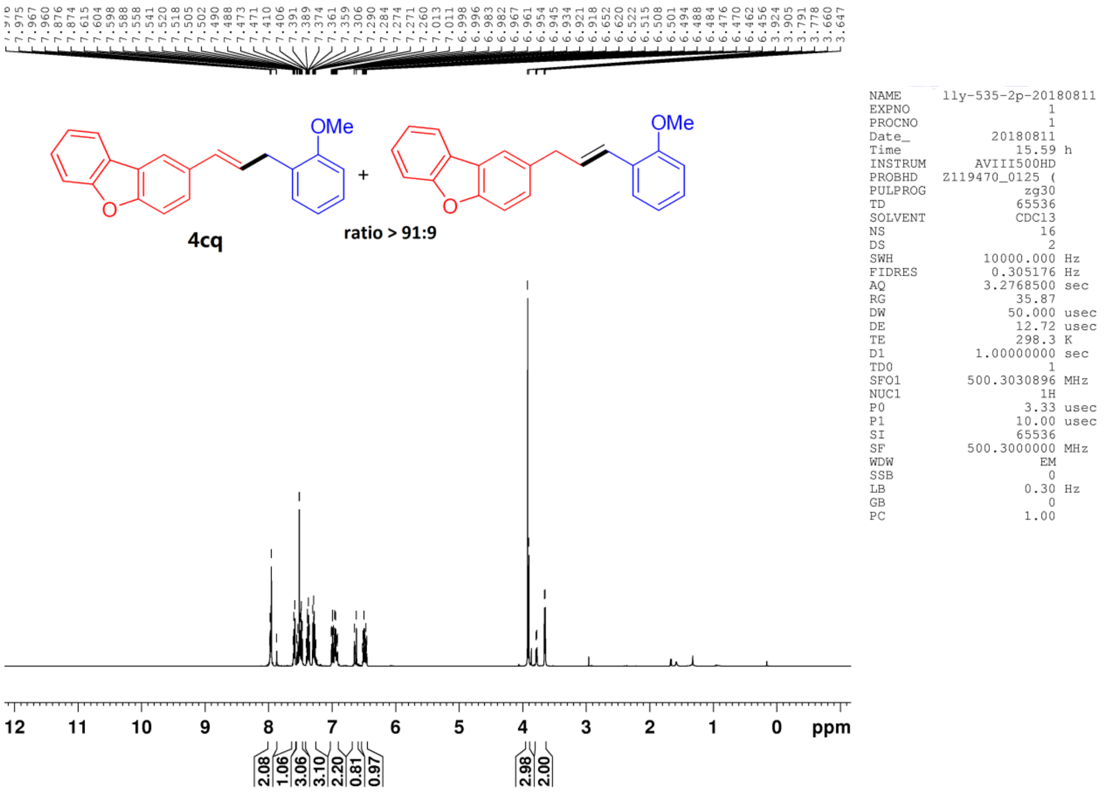

Supplementary Figure 110. ^{1}H NMR spectra for compound **4cq**

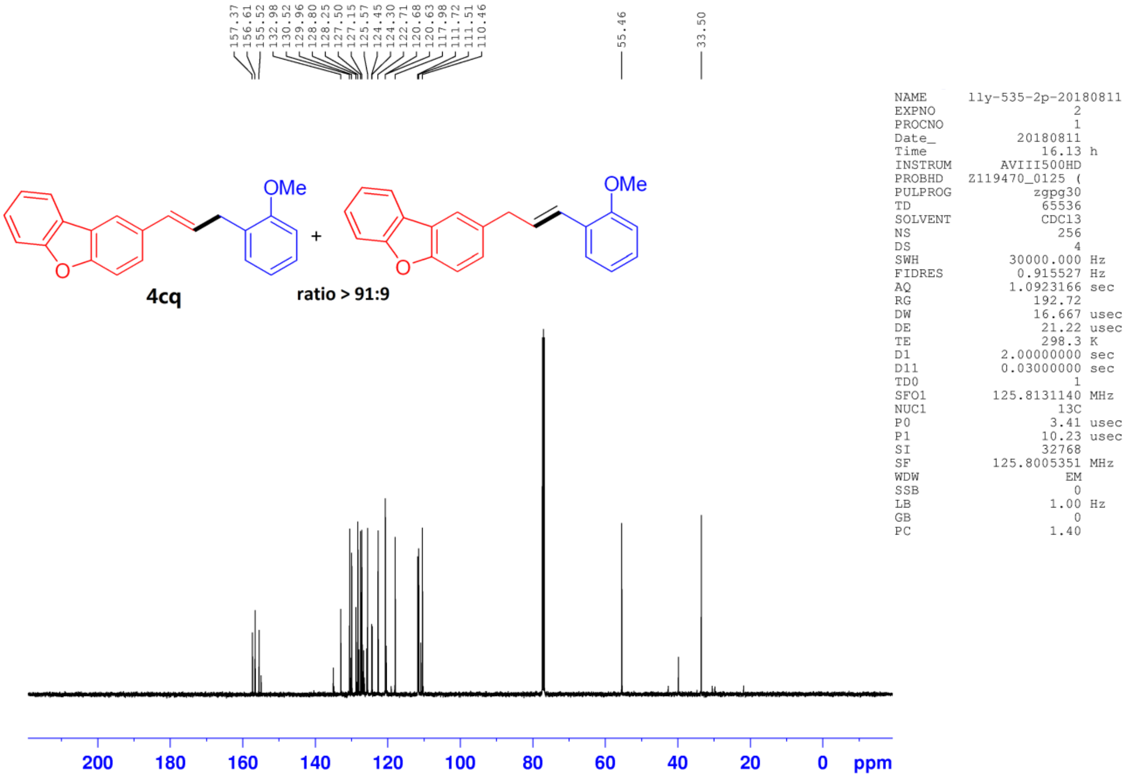

Supplementary Figure 111. ^{13}C NMR spectra for compound **4cq**

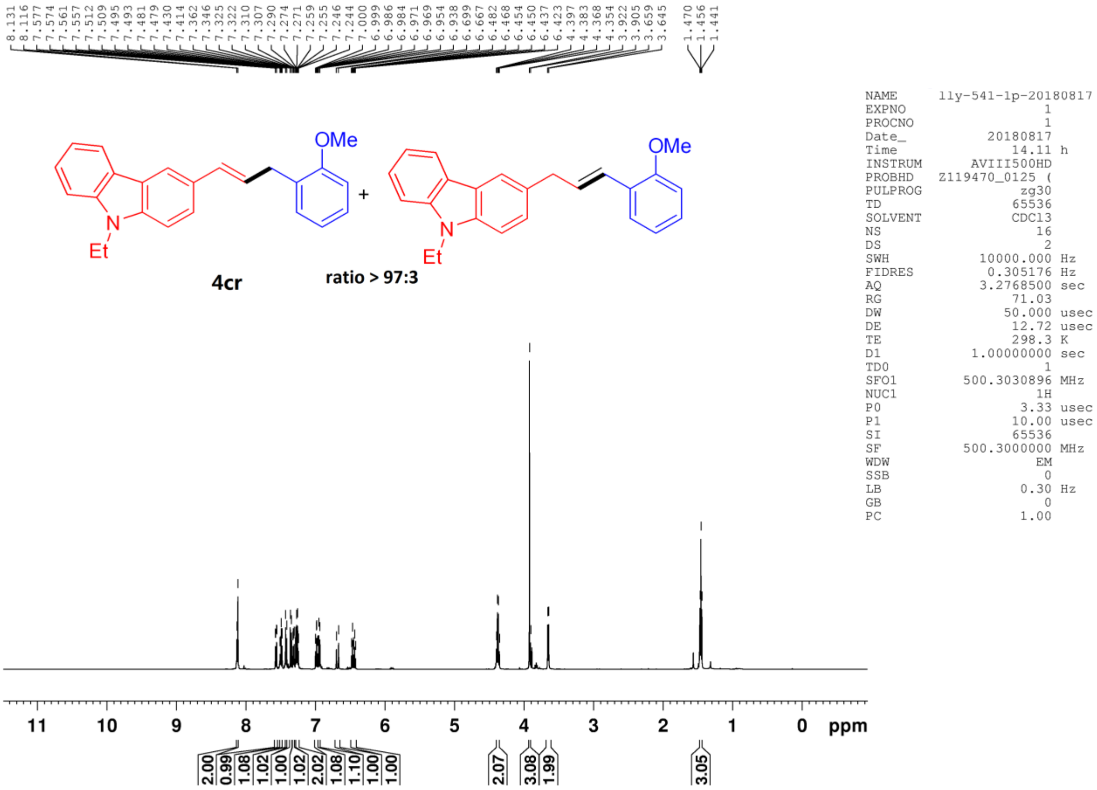

Supplementary Figure 112. ^{1}H NMR spectra for compound **4cr**

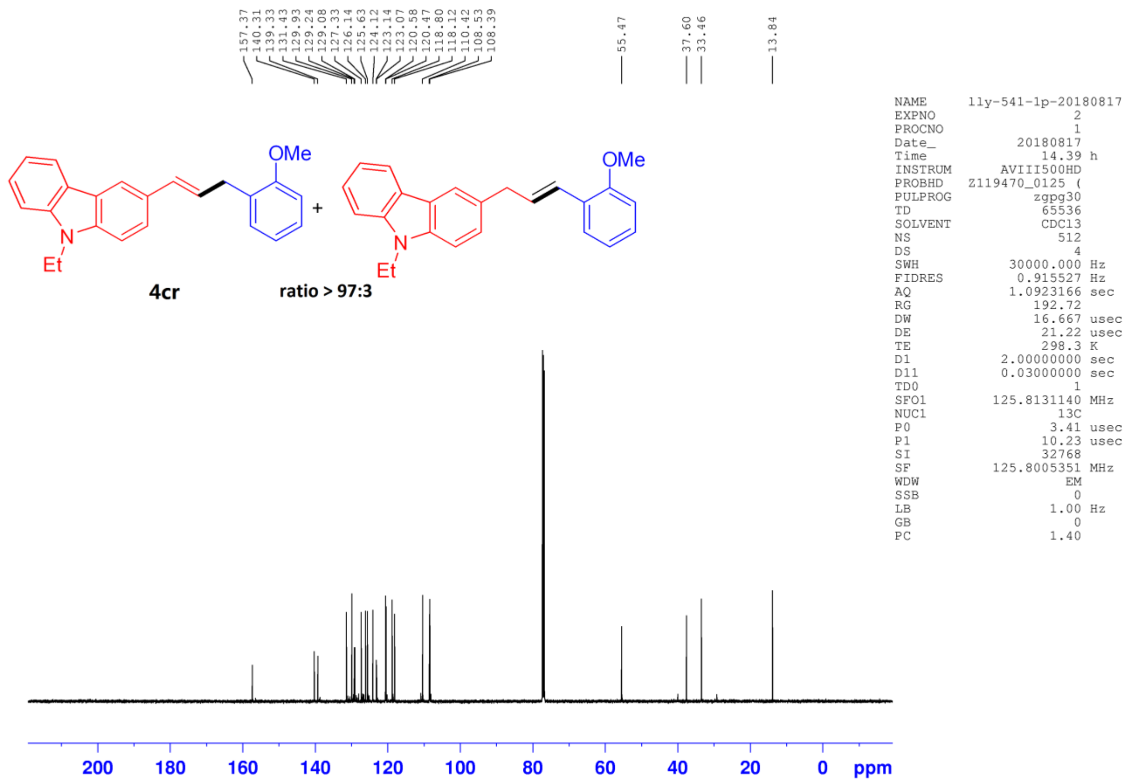

Supplementary Figure 113. ^{1}H NMR spectra for compound **4cr**

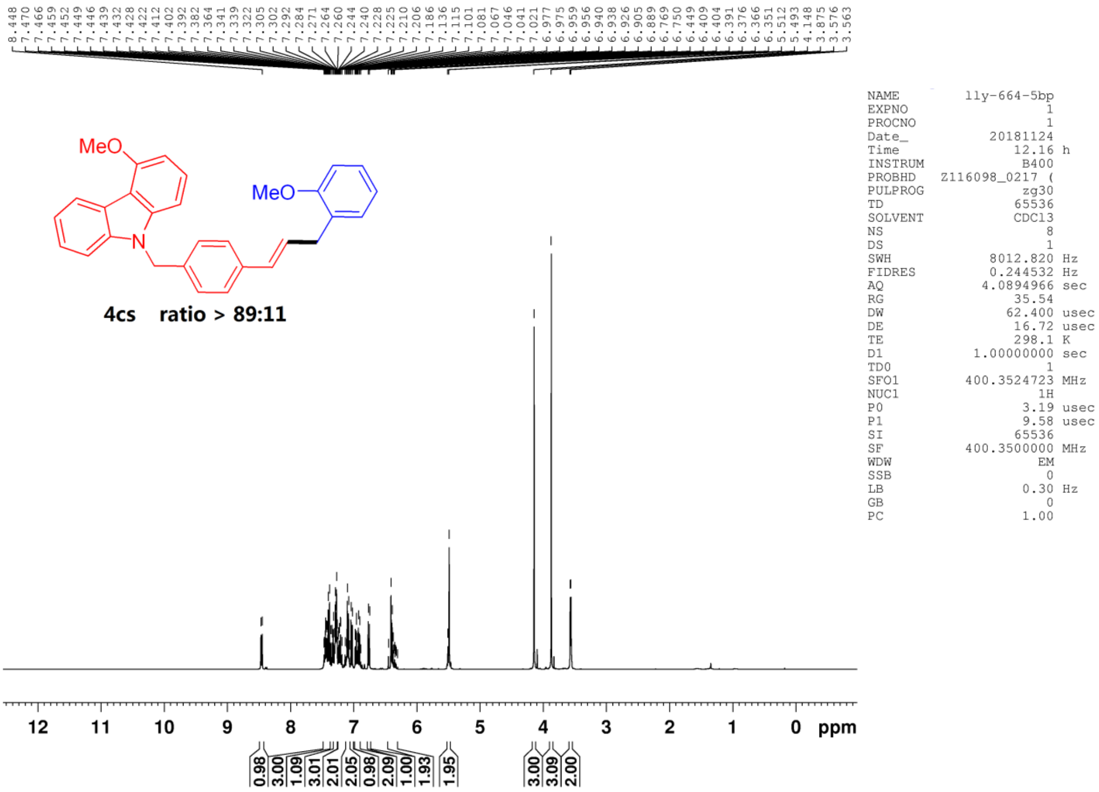

Supplementary Figure 114. ^{1}H NMR spectra for compound **4cs**

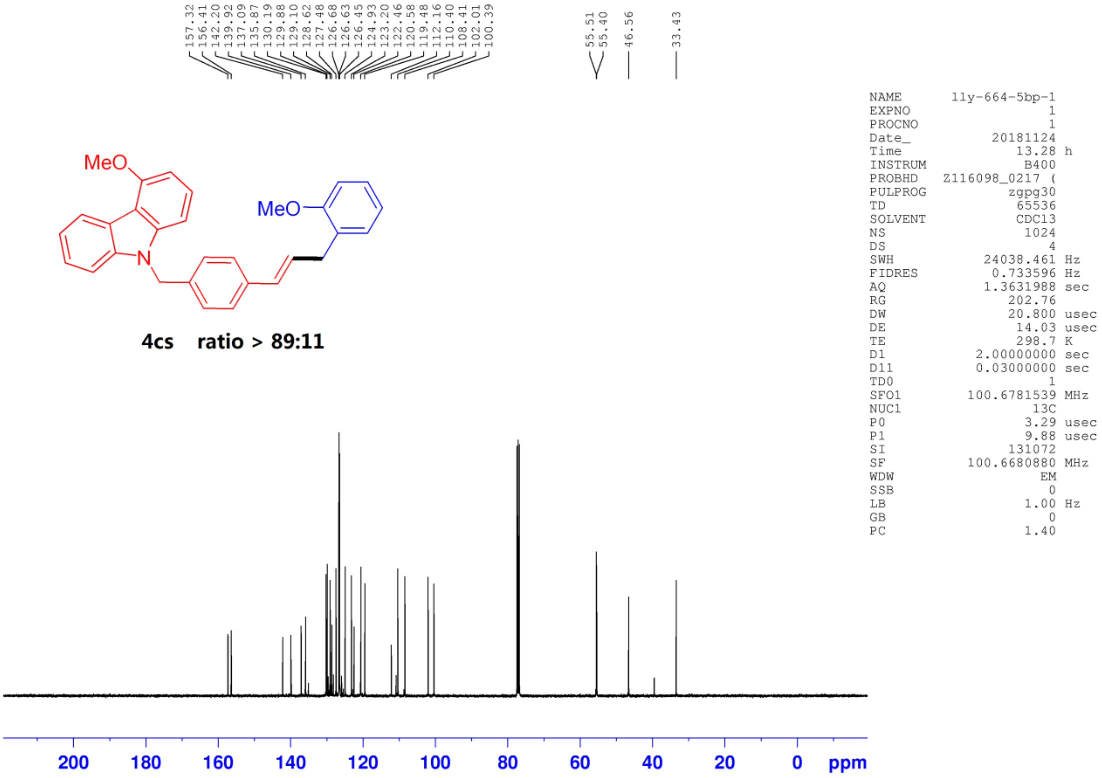

Supplementary Figure 115. ^{13}C NMR spectra for compound **4cs**

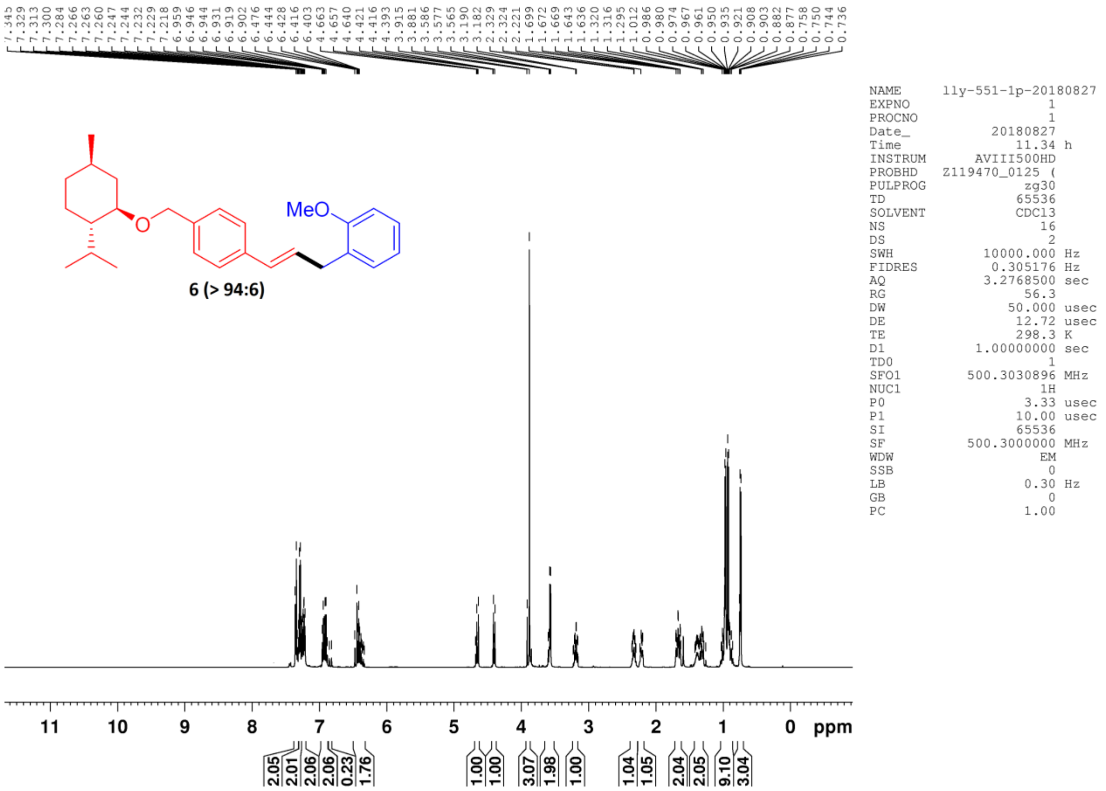

Supplementary Figure 116. ^{1}H NMR spectra for compound **6**

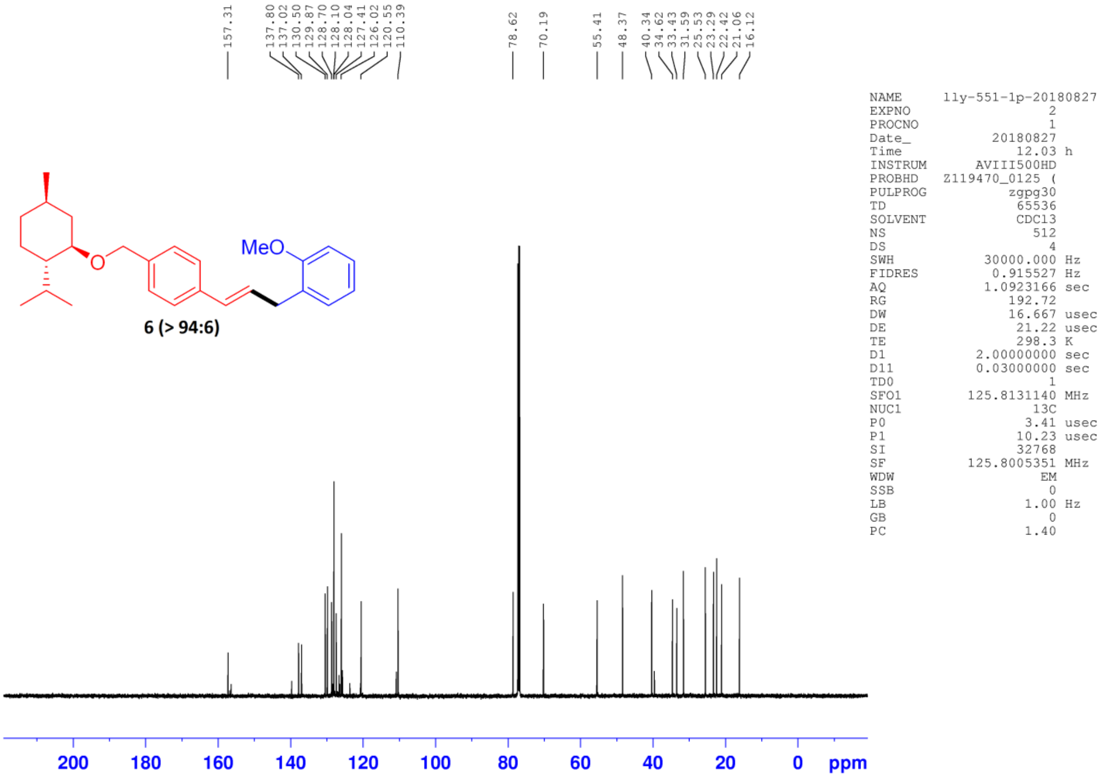

Supplementary Figure 117. ^{13}C NMR spectra for compound **6**

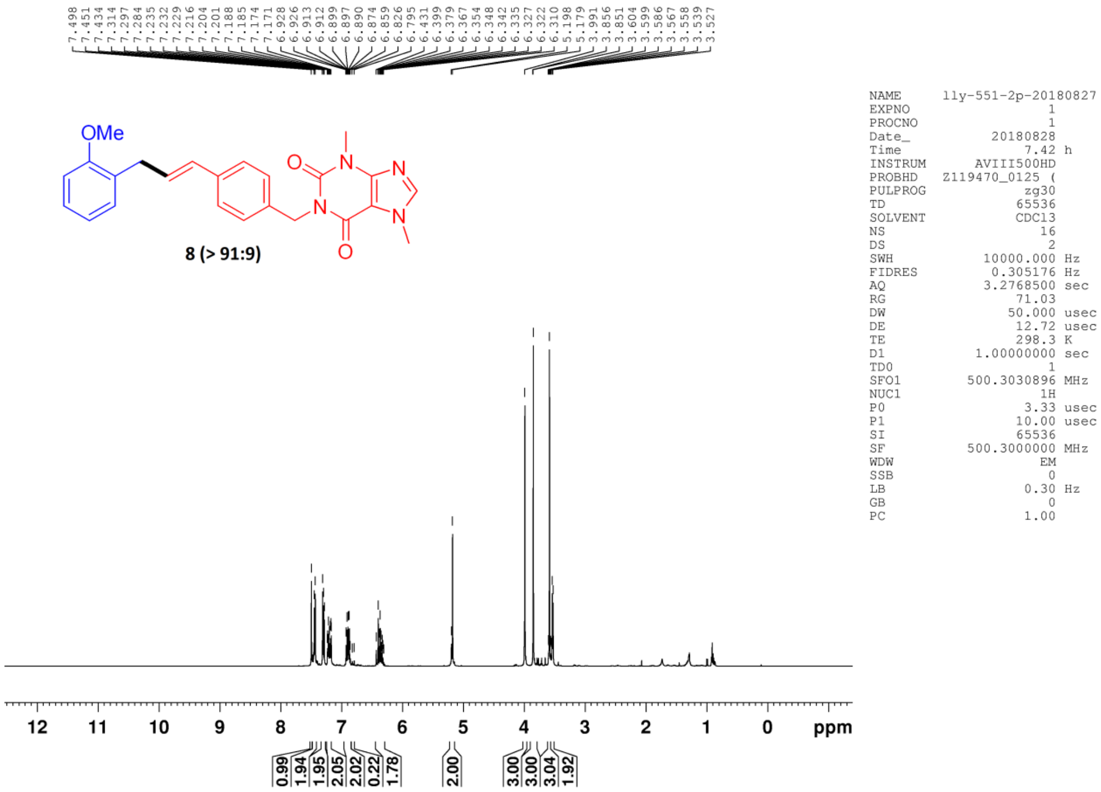

Supplementary Figure 118. ^{1}H NMR spectra for compound **8**

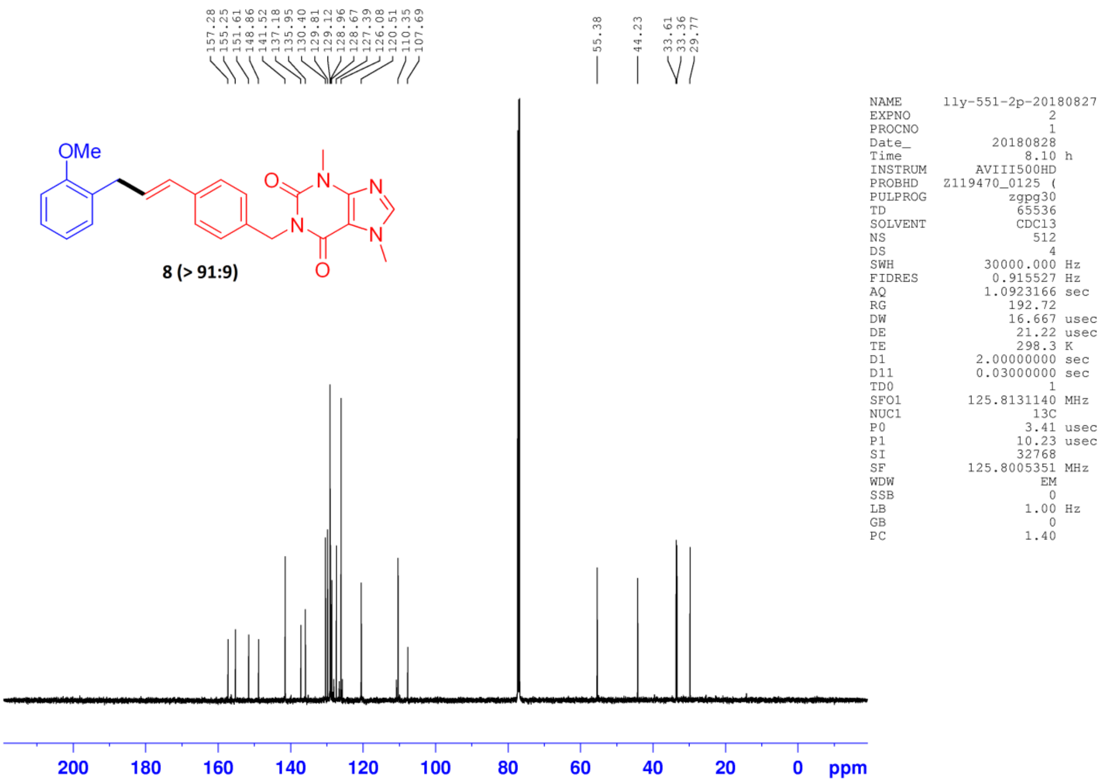

Supplementary Figure 119. ^{13}C NMR spectra for compound **8**

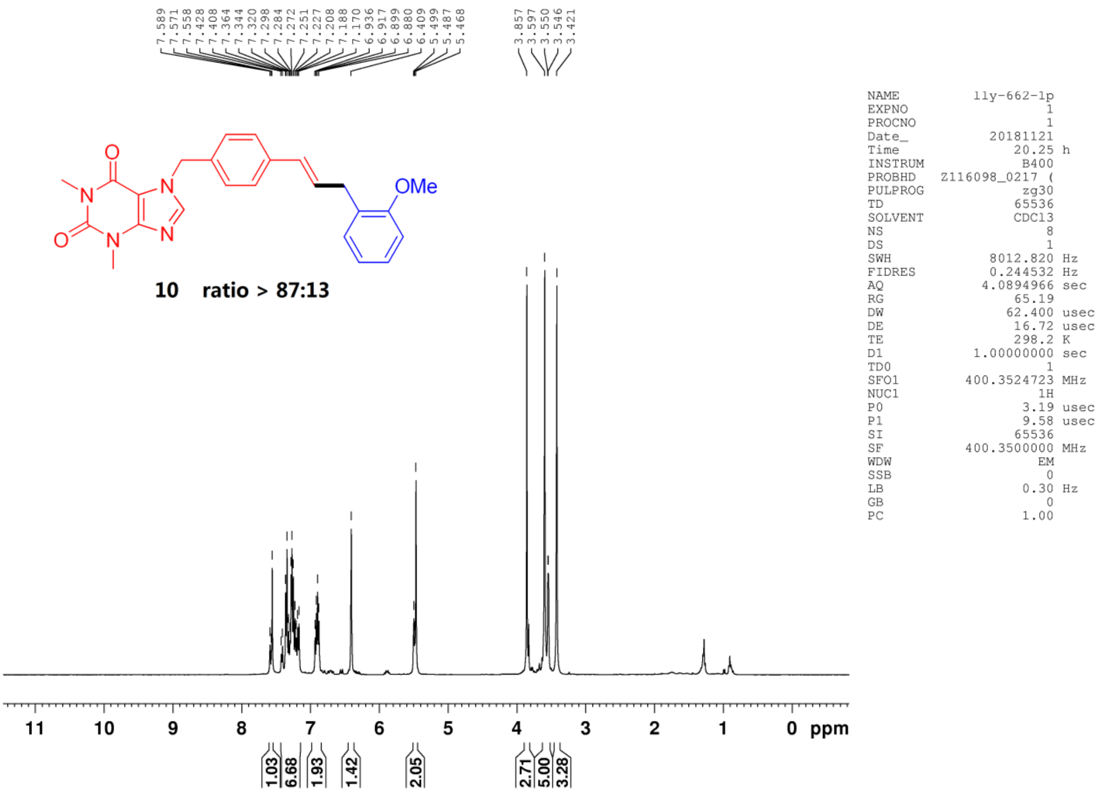

Supplementary Figure 120. ^{1}H NMR spectra for compound **10**

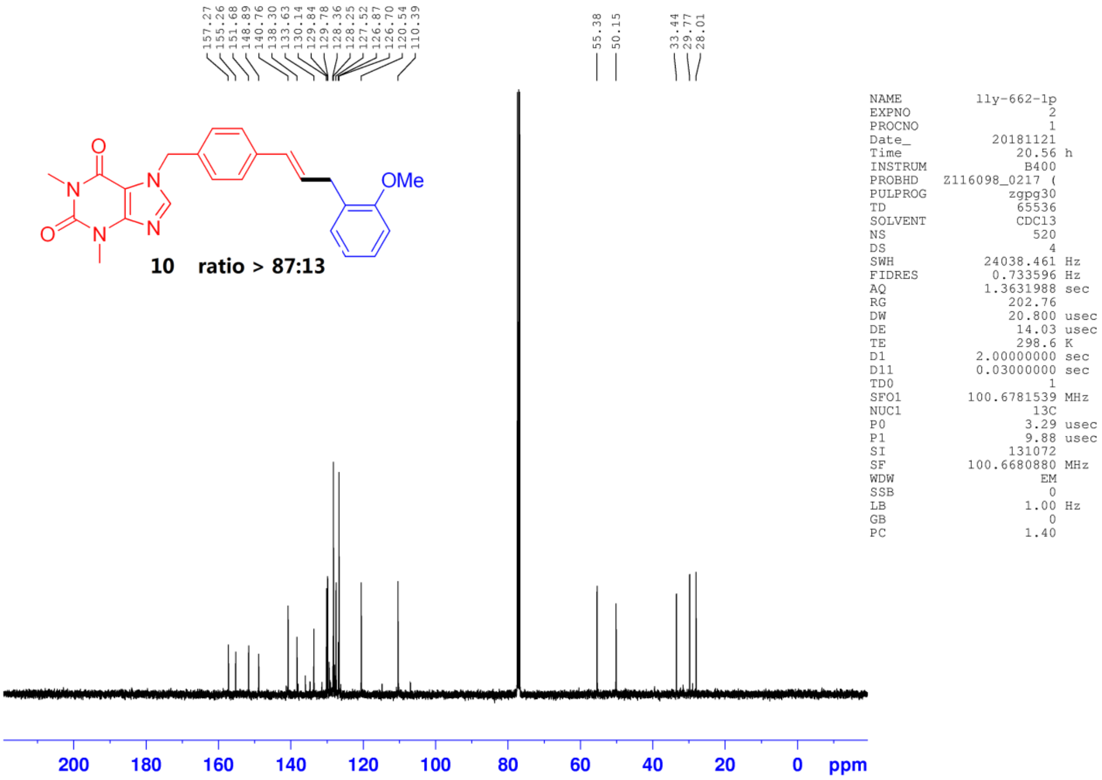

Supplementary Figure 121. ^{13}C NMR spectra for compound **10**

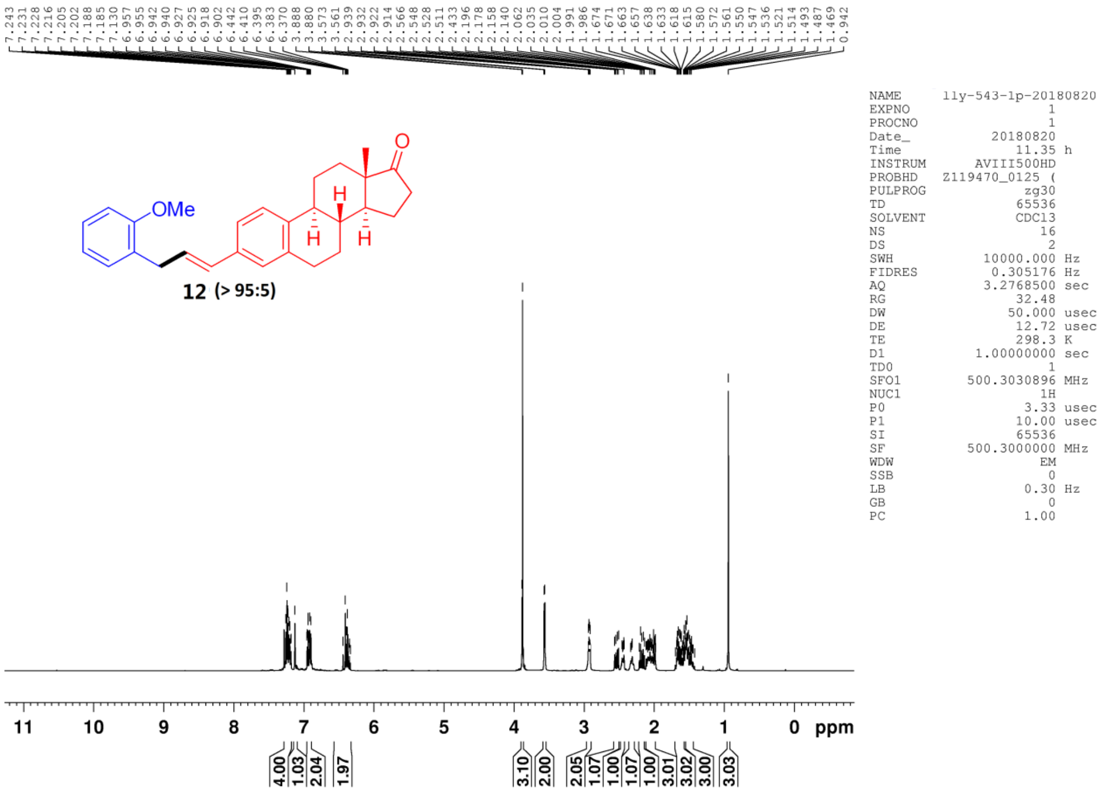

Supplementary Figure 122. ^{1}H NMR spectra for compound **12**

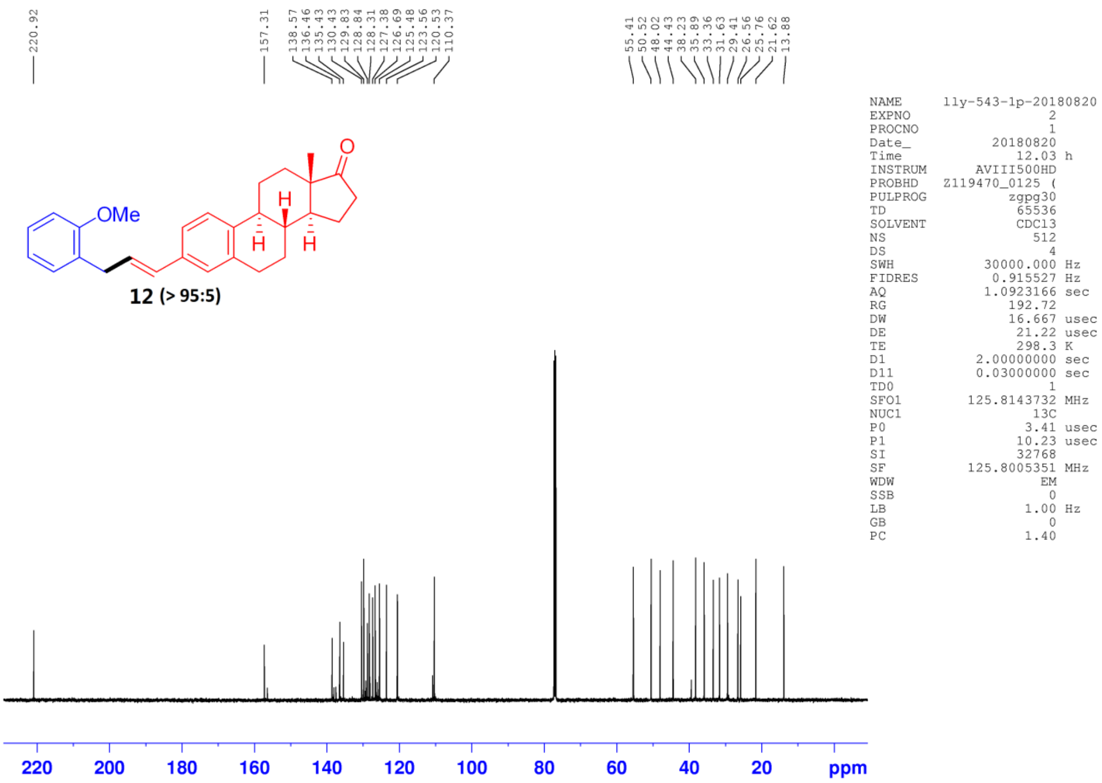

Supplementary Figure 123. ^{13}C NMR spectra for compound **12**

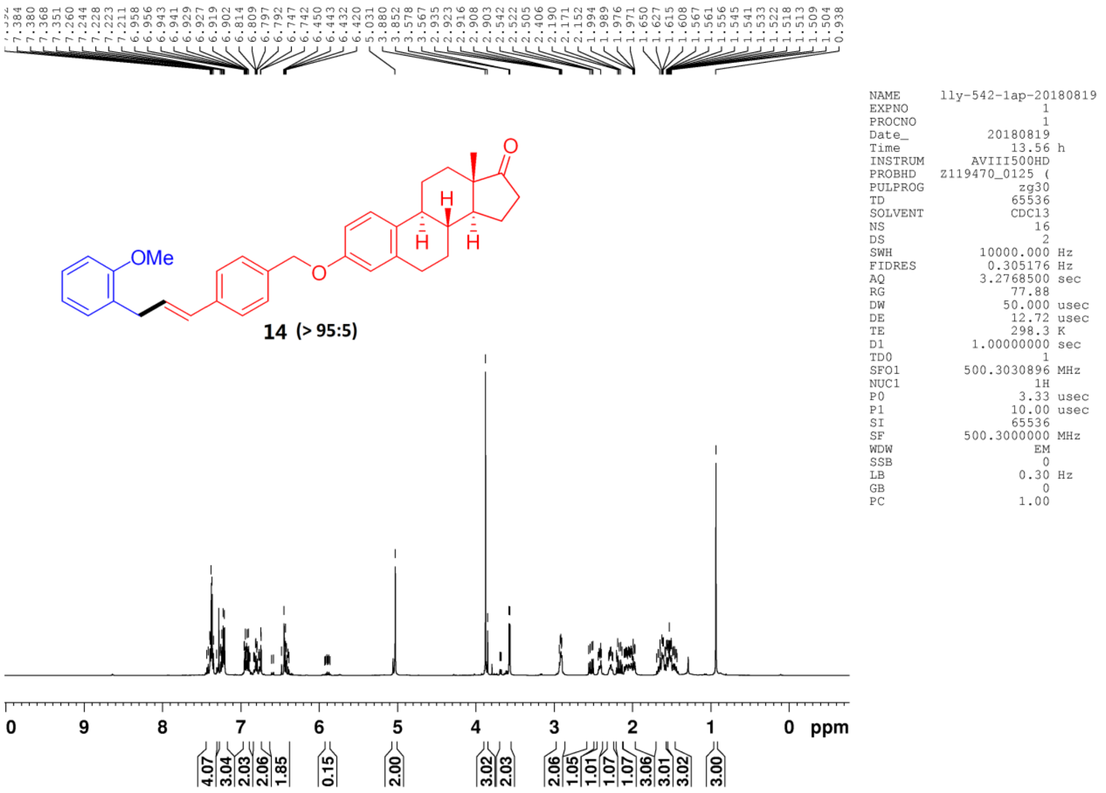

Supplementary Figure 124. ^{1}H NMR spectra for compound **14**

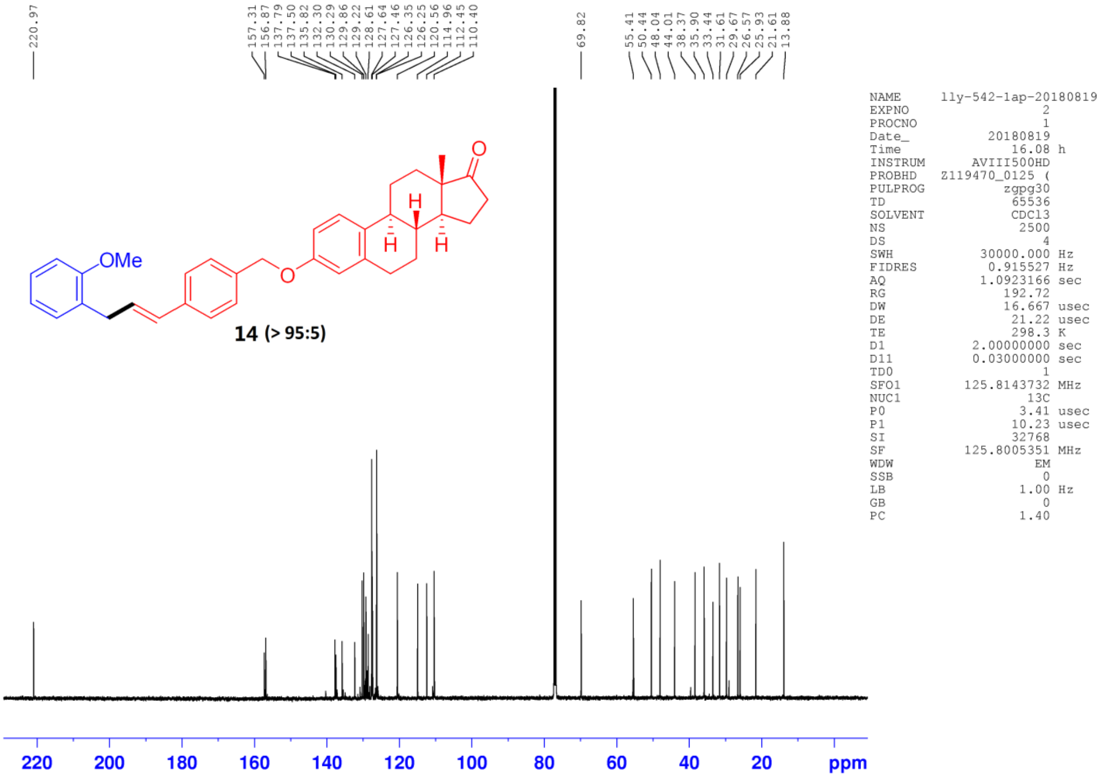

Supplementary Figure 125. ^{13}C NMR spectra for compound **14**

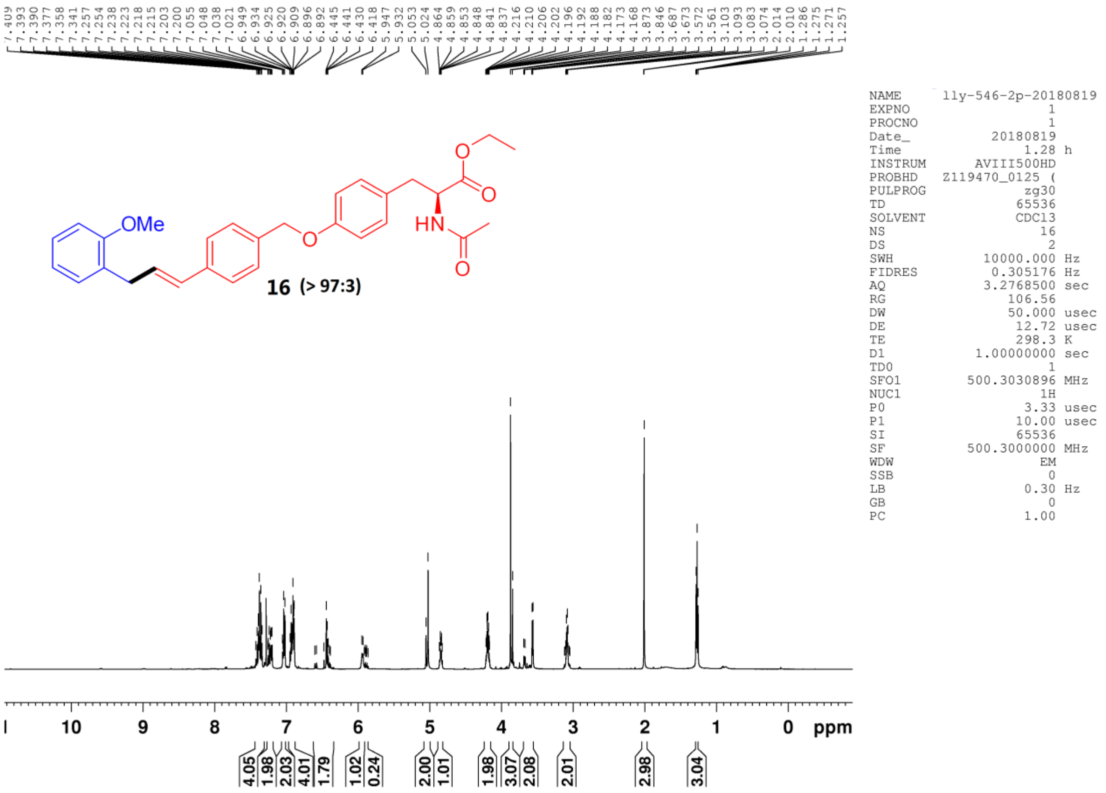

Supplementary Figure 126. ^{1}H NMR spectra for compound **16**

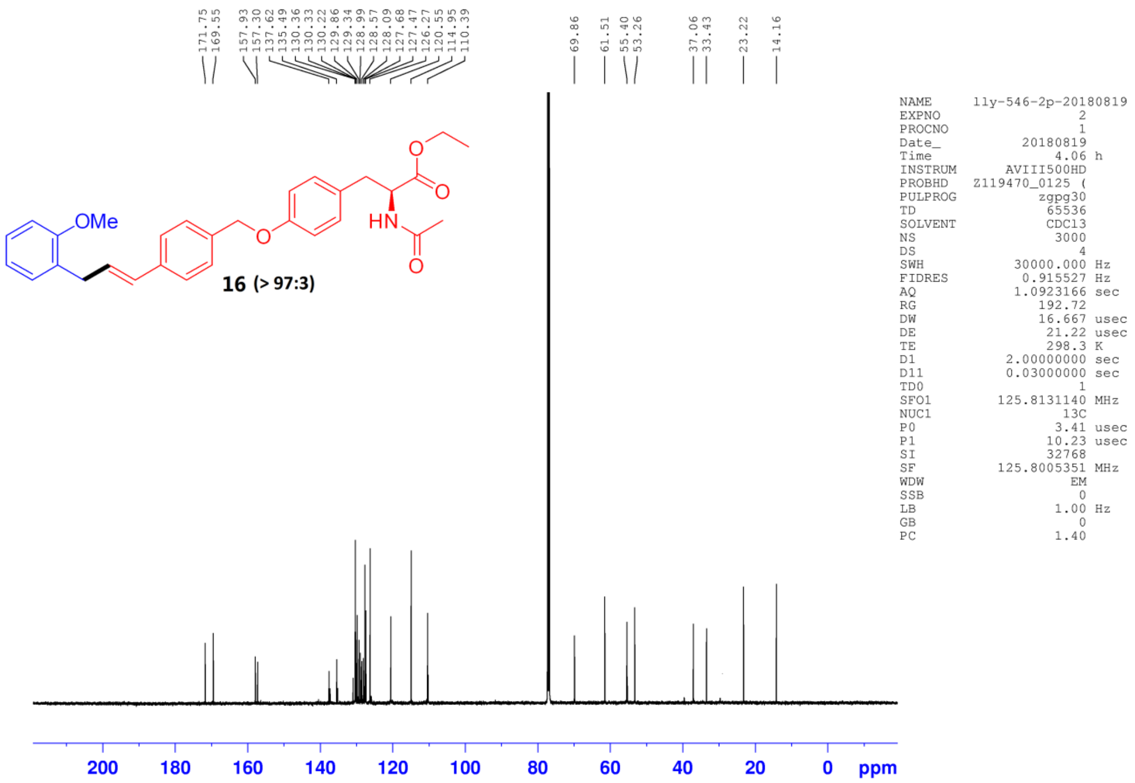

Supplementary Figure 127. ^{13}C NMR spectra for compound **16**

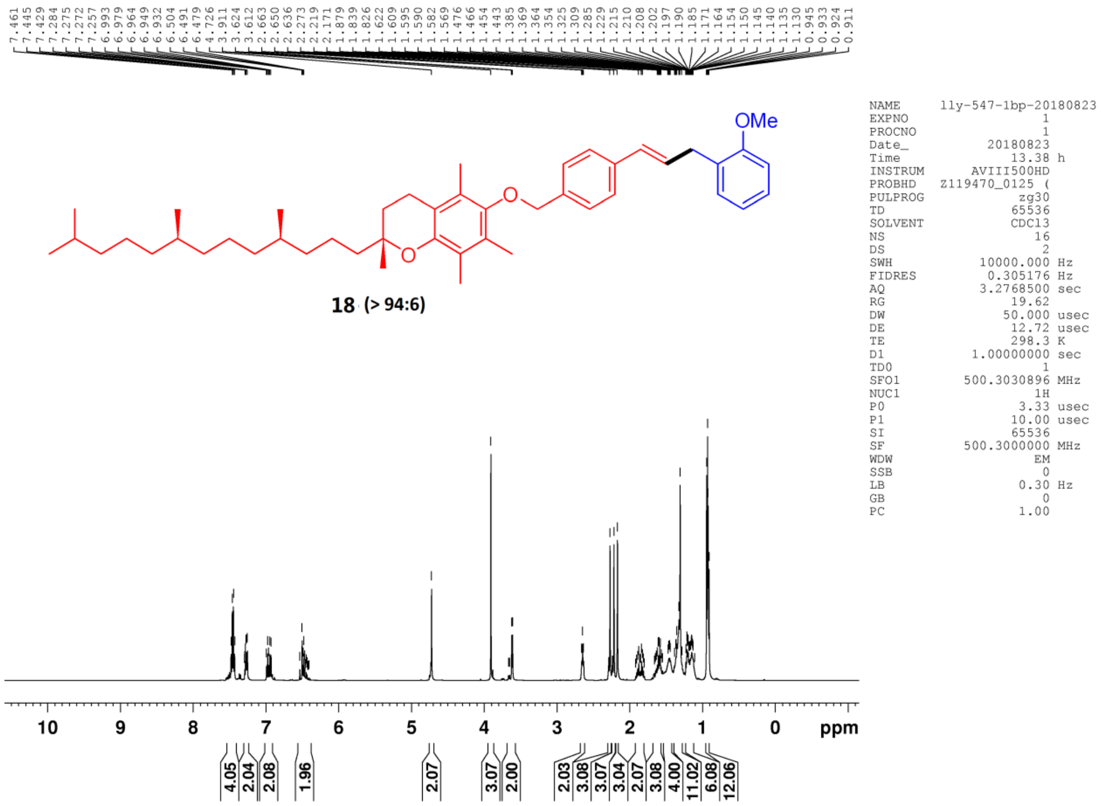

Supplementary Figure 128. ^{1}H NMR spectra for compound **18**

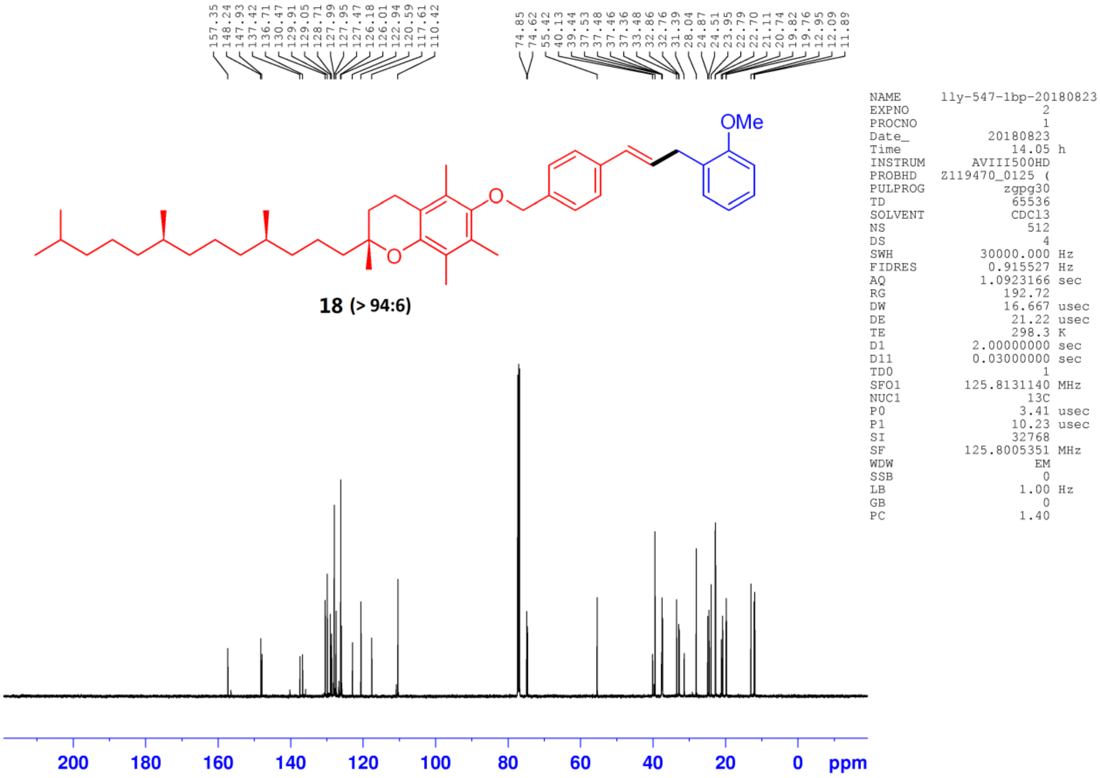

Supplementary Figure 129. ^{13}C NMR spectra for compound **18**

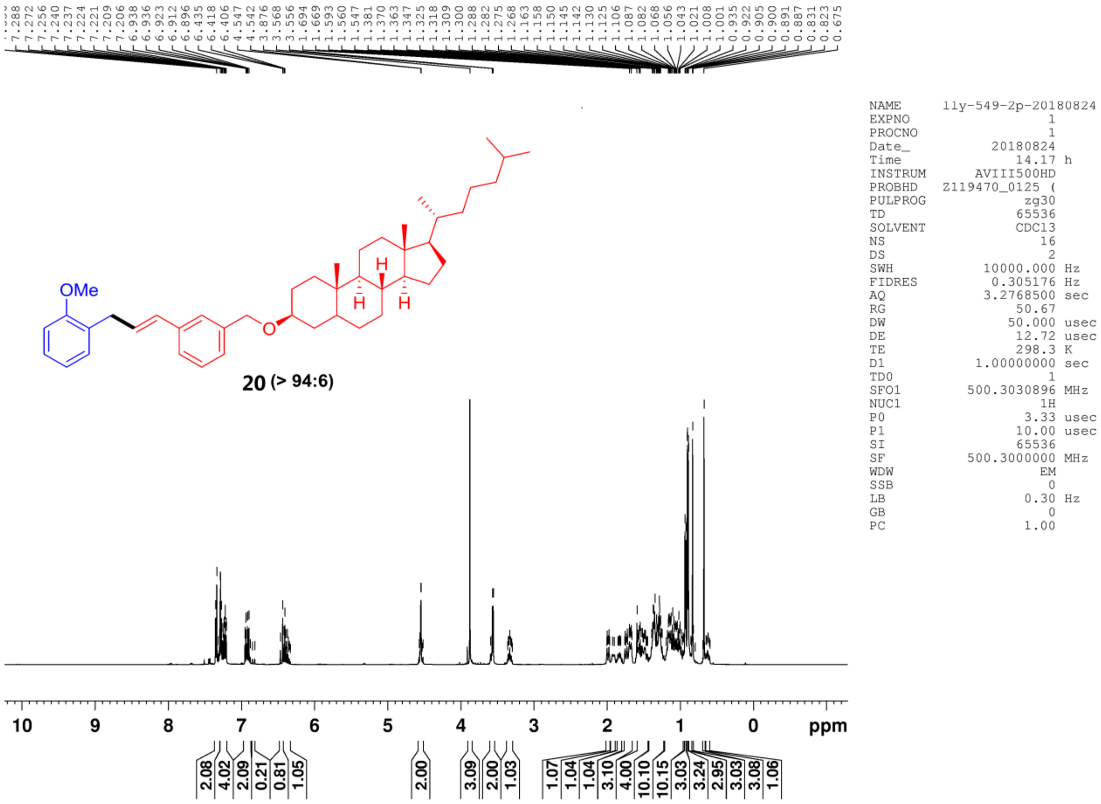

Supplementary Figure 130. ^{1}H NMR spectra for compound **20**

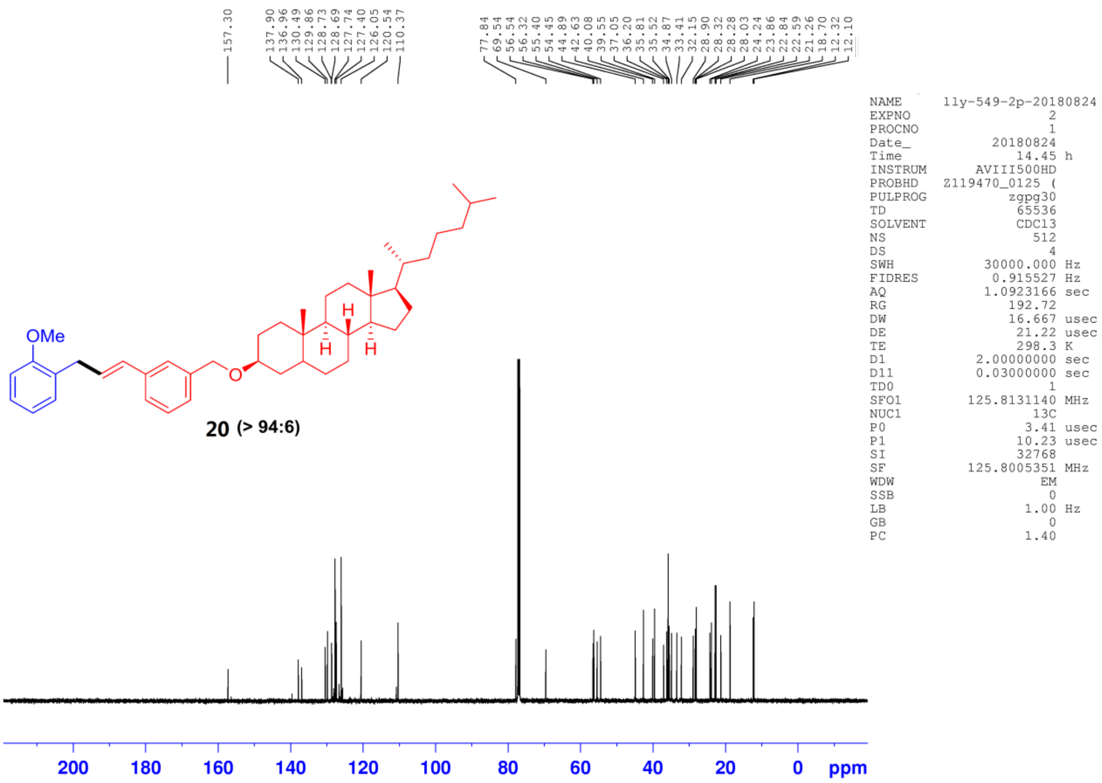

Supplementary Figure 131. ^{13}C NMR spectra for compound **20**

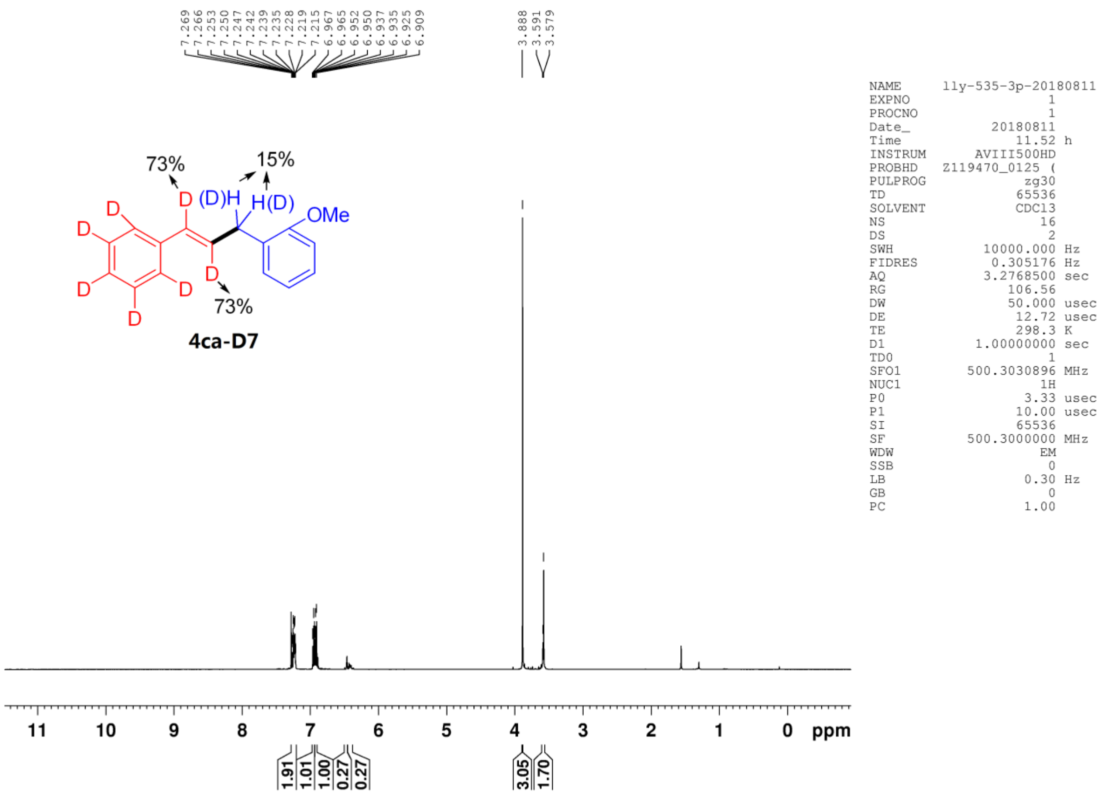

Supplementary Figure 132. ^{1}H NMR spectra for compound **4ca-D7**

Supplementary References

[1] Hamasaka, G., Sakurai, F. & Uozumi, Y. A palladium NNC-Pincer complex: an efficient catalyst for allylic arylation at parts per billion levels. *Chem. Commun.* **51**, 3886-3888 (2015).

[2] Yang, H., Yan, H., Sun, P., Zhu, Y., Lu, L., Liu, D., Rong, G. & Mao, J. Iron-catalyzed direct alkenylation of sp^3(C-H) bonds via decarboxylation of cinnamic acids under ligand-free conditions. *Green Chem.* **15**, 976-981 (2013).

[3] Jin, W., Wong, W.-T. & Law, G.-L. A simple and direct method for the palladium-catalyzed oxidative coupling of unactivated allylarenes with classic arenes. *Chem. Cat. Chem.* **6**, 1599-1603 (2014).

[4] Werner, E. W. & Sigman, M. S. Operationally simple and highly (*E*)-styrenyl-selective Heck reactions of electronically nonbiased olefins. *J. Am. Chem. Soc.* **133**, 9692-9695 (2011).

[5] Cai, Y., Benischke, A. D., Knochel, P. & Gosmini, C. Cobalt-catalyzed reductive cross-coupling between styryl and benzyl halides. *Chem.- Eur. J.* **23**, 250-253 (2017).

[6] Lei, C., Yip, Y. J. & Zhou, J. S. Nickel-catalyzed direct synthesis of aryl olefins from ketones and organoboron reagents under neutral conditions. *J. Am. Chem. Soc.* **139**, 6086-6089 (2017).

[7] Pérez-Aguilar, M. C. & Valdés, C. Olefination of carbonyl compounds through reductive coupling of alkenylboronic acids and tosylhydrazones. *Angew. Chem. Int. Ed.* **51**, 5953-5957 (2012).

[8] Zhou, B., Sato, H., Ilies, L. & Nakamura, E. Iron-catalyzed remote arylation of aliphatic C-H bond via 1,5-hydrogen shift. *ACS Catalysis* **8**, 8-11 (2018).

[9] Jia, X.-G., Guo, P., Duan, J. & Shu, X.-Z. Dual nickel and lewis acid catalysis for cross-electrophile coupling: the allylation of aryl halides with allylic alcohols. *Chem. Sci.* **9**, 640-645 (2018).

[10] Ricardo, C. L., Mo, X., McCubbin, J. A. & Hall, D. G. A surprising substituent effect provides a superior boronic acid catalyst for mild and metal-free direct Friedel-Crafts alkylations and prenylations of neutral arenes. *Chem.- Eur. J.* **21**, 4218-4223 (2015).

[11] Alacid, E. & Nájera, C. Palladium-catalyzed cross-coupling reactions of potassium alkenyltrifluoroborates with organic halides in aqueous media. *J. Org. Chem.* **74**, 2321-2327 (2009).

[12] Tao, J.-L., Yang, B. & Wang, Z.-X. Pincer-nickel-catalyzed allyl-aryl coupling between allyl methyl ethers and arylzinc chlorides. *J. Org. Chem.* **80**, 12627-12634 (2015).

[13] Chan, C.-K., Tsai, Y.-L. & Chang, M.-Y. $Bi(OTf)_3$ catalyzed disproportionation reaction of cinnamyl alcohols. *Tetrahedron* **73**, 3368-3376 (2017).

[14] Mino, T., Kogure, T., Abe, T., Koizumi, T., Fujita, T. & Sakamoto, M. Palladium-catalyzed allylic arylation of allylic ethers with arylboronic acids using hydrazone ligands. *Eur. J. Org. Chem.* 1501-1505 (2013).

[15] Onodera, G., Imajima, H., Yamanashi, M., Nishibayashi, Y., Hidai, M. & Uemura, S. Ruthenium-catalyzed allylation of aromatic compounds and allylic ether formation. *Organometallics* **23**, 5841-5848 (2004).

[16] Yoshimitsu, T., Arano, Y. & Nagaoka, H. Radical α-C-H hydroxyalkylation of ethers and acetal. *J. Org. Chem.* **70**, 2342-2345 (2005).

[17] Li, G., Wu, L., Lv, G., Liu, H., Fu, Q., Zhang, X. & Tang, Z. Alkyl transfer from C-C cleavage: replacing the nitro group of nitro-olefins. *Chem. Commun.* **50**, 6246-6248 (2014).

[18] Wang, G.-Z., Shang, R., Cheng, W.-M. & Fu, Y. Irradiation-induced Heck reaction of unactivated alkyl halides at room temperature. *J. Am. Chem. Soc.* **139**, 18307-18312 (2017).

[19] Nazari, S. H., Bourdeau, J. E., Talley, M. R., Valdivia-Berroeta, G. A., Smith, S. J. & Michaelis, D. J. Nickel-catalyzed Suzuki cross couplings with unprotected allylic alcohols enabled by bidentate *N*-heterocyclic carbene (NHC)/phosphine ligands. *ACS Catalysis* **8**, 86-89 (2018).

[20] Friscourt, F., Fahrni, C. J. & Boons, G.-J. Fluorogenic strain-promoted alkyne–diazo cycloadditions. *Chem.-Eur. J.* **21**, 13996-14001 (2015)

[21] Barluenga, J., Quiñones, N., Tomás-Gamasa, M. & Cabal, M.-P. Intermolecular metal-free cyclopropanation of alkenes using tosylhydrazones. *Eur. J. Org. Chem.* 2312-2317 (2012).

[22] Lv, L., Qiu, Z., Li, J., Liu, M. & Li, C.-J. N_2H_4 as traceless mediator for homo- and cross- aryl coupling. *Nat. Commun.* **9**, 4739 (2018).
